# Derivation, internal validation, and recalibration of a cardiovascular risk score for Latin America and the Caribbean (Globorisk-LAC): A pooled analysis of cohort studies

**DOI:** 10.1016/j.lana.2022.100258

**Published:** 2022-04-23

**Authors:** Cohorts Consortium of Latin America and the Caribbean (CC-LAC), Rodrigo M Carrillo-Larco, Dalia Stern, Ian R Hambleton, Paulo Lotufo, Mariachiara Di Cesare, Anselm Hennis, Catterina Ferreccio, Vilma Irazola, Pablo Perel, Edward W Gregg, J Jaime Miranda, Majid Ezzati, Goodarz Danaei, Carlos A Aguilar-Salinas, Ramón Alvarez-Váz, Marselle B Amadio, Cecilia Baccino, Claudia Bambs, João Luiz Bastos, Gloria Beckles, Antonio Bernabe-Ortiz, Carla DO Bernardo, Katia V Bloch, Juan E Blümel, Jose G Boggia, Pollyanna K Borges, Miguel Bravo, Gilbert Brenes-Camacho, Horacio A Carbajal, Paola Casas-Vasquez, Maria S Castillo Rascon, Blanca H Ceballos, Veronica Colpani, Jackie A Cooper, Sandra Cortes, Adrian Cortes-Valencia, Roberto S Cunha, Eleonora d'Orsi, William H Dow, Walter G Espeche, Flavio D Fuchs, Sandra C Fuchs, Suely GA Gimeno, Donaji Gomez-Velasco, David A Gonzalez-Chica, Clicerio Gonzalez-Villalpando, María-Elena Gonzalez-Villalpando, Gonzalo Grazioli, Ricardo O Guerra, Laura Gutierrez, Fernando L Herkenhoff, Andrea RVR Horimoto, Andrea Huidobro, Elard Koch, Martin Lajous, Maria Fernanda Lima-Costa, Ruy Lopez-Ridaura, Alvaro CC Maciel, Gladys E Maestre, Betty S Manrique-Espinoza, Larissa P Marques, Jesus D Melgarejo, Luis J Mena, Jose G Mill, Leila B Moreira, Oscar M Muñoz, Lariane M Ono, Karen Oppermann, Pedro J Ortiz, Karina M Paiva, Sergio V Peixoto, Alexandre C Pereira, Karen G Peres, Marco A Peres, Paula Ramírez-Palacios, Cassiano R Rech, Berenice Rivera-Paredez, Nohora I Rodriguez, Rosalba Rojas-Martinez, Luis Rosero-Bixby, Adolfo Rubinstein, Alvaro Ruiz-Morales, Martin R Salazar, Aaron Salinas-Rodriguez, Jorge Salmerón, Ramon A Sanchez, Nelson AS Silva, Thiago LN Silva, Liam Smeeth, Poli M Spritzer, Fiorella Tartaglione, Jorge Tartaglione, Tania Tello, Rafael Velázquez-Cruz

**Affiliations:** 1 Imperial College London, UK; 2 National Institute of Public Health, Mexico; 3 The University of the West Indies, Barbados; 4 University of São Paulo, Brazil; 5 Middlesex University, UK; 6 Pan American Health Organization, USA; 7 Pontificia Universidad Católica de Chile, Advanced Centre for Chronic Diseases ACCDiS, Chile; 8 Institute for Clinical Effectiveness and Health Policy, Argentina; 9 London School of Hygiene and Tropical Medicine, UK; 10 Universidad Peruana Cayetano Heredia, Peru; 11 Harvard TH Chan School of Public Health, USA; 12 Instituto Nacional de Ciencias Médicas y Nutrición, Mexico; 13 Universidad de la Republica, Uruguay; 14 Centro Universitario Senac Santo Amaro, Brazil; 15 Pontificia Universidad Católica de Chile, Chile; 16 Universidade Federal de Santa Catarina, Brazil; 17 Centers for Disease Control and Prevention, USA; 18 Universidad Peruana Cayetano Heredia, Peru; 19 The University of Adelaide, Australia; 20 Universidade Federal do Rio de Janeiro (UFRJ), Brazil; 21 Universidad de Chile, Chile; 22 Universidad de la República, Uruguay; 23 Universidade Estadual de Ponta Grossa, Brazil; 24 MELISA Institute, Chile; 25 Universidad de Costa Rica, Costa Rica; 26 Universidad Nacional de la Plata, Argentina; 27 Universidad Nacional de Misiones, Argentina; 28 Hospital Dr Ramon Madariaga, Argentina; 29 Federal University of Rio Grande do Sul, Brazil; 30 Queen Mary University of London, UK; 31 National Institute of Public Health, Mexico; 32 Federal University of Espírito Santo, Brazil; 33 University of California, Berkeley, USA; 34 Universidade Federal do Rio Grande do Sul, Brazil; 35 Universidade Federal de São Paulo, Brazil; 36 Instituto Nacional de Salud Pública, México; 37 Centro de Estudios en Diabetes A.C., México; 38 Hospital Churruca Visca, Argentina; 39 Federal University of Rio Grande do Norte, Brazil; 40 Institute for Clinical Effectiveness and Health Policy, Argentina; 41 University of São Paulo, Brazil; 42 Universidad Católica del Maule, Chile; 43 Harvard T.H. Chan School of Public Health, USA; 44 Oswaldo Cruz Foundation, Brazil; 45 University of Texas Rio Grande Valley, USA; 46 University of Leuven, Belgium; 47 Universidad Politécnica de Sinaloa, México; 48 Pontificia Universidad Javeriana, Hospital Universitario San Ignacio, Colombia; 49 Universidade Federal do Paraná, Brazil; 50 Passo Fundo University, Brazil; 51 NDRIS/NDCS Duke-NUS Medical School, Singapore; 52 IMSS Epidemiology and Health Services Research Unit, Mexico; 53 National Autonomous University of Mexico, Mexico; 54 Clinica de Marly, Colombia; 55 Instituto Nacional de Salud Pública, México; 56 Pontificia Universidad Javeriana, Colombia; 57 London School of Hygiene & Tropical Medicine, UK; 58 National Institute of Genomic Medicine (INMEGEN), Mexico

**Keywords:** Risk prediction, Primary prevention, Global health, Cardiovascular diseases, Latin America and the Caribbean, Predicción de riesgo, Prevención primaria, Salud global

## Abstract

**Background:**

Risk stratification is a cornerstone of cardiovascular disease (CVD) prevention and a main strategy proposed to achieve global goals of reducing premature CVD deaths. There are no cardiovascular risk scores based on data from Latin America and the Caribbean (LAC) and it is unknown how well risk scores based on European and North American cohorts represent true risk among LAC populations.

**Methods:**

We developed a CVD (including coronary heart disease and stroke) risk score for fatal/non-fatal events using pooled data from 9 prospective cohorts with 21,378 participants and 1,202 events. We developed laboratory-based (systolic blood pressure, total cholesterol, diabetes, and smoking), and office-based (body mass index replaced total cholesterol and diabetes) models. We used Cox proportional hazards and held back a subset of participants to internally validate our models by estimating Harrell's C-statistic and calibration slopes.

**Findings:**

The C-statistic for the laboratory-based model was 72% (70–74%), the calibration slope was 0.994 (0.934–1.055) among men and 0.852 (0.761–0.942) among women; for the office-based model the C-statistic was 71% (69–72%) and the calibration slope was 1.028 (0.980–1.076) among men and 0.811 (0.663–0.958) among women. In the pooled sample, using a 20% risk threshold, the laboratory-based model had sensitivity of 21.9% and specificity of 94.2%. Lowering the threshold to 10% increased sensitivity to 52.3% and reduced specificity to 78.7%.

**Interpretation:**

The cardiovascular risk score herein developed had adequate discrimination and calibration. The Globorisk-LAC would be more appropriate for LAC than the current global or regional risk scores. This work provides a tool to strengthen risk-based cardiovascular prevention in LAC.

**Funding:**

Wellcome Trust (214185/Z/18/Z)

## Abstract (es)

**Antecedentes:**

La estratificación de riesgo es piedra angular en la prevención cardiovascular y una estrategia fundamental para reducir la mortalidad prematura por enfermedades cardiovasculares. No existe un puntaje de riesgo (*risk score*) cardiovascular basado en datos de América Latina y el Caribe (ALC), y se desconoce qué tan bien los puntajes basados en cohortes de Europa y América del Norte representan el riesgo cardiovascular en población de ALC.

**Métodos:**

Desarrollamos un puntaje de riesgo para enfermedades cardiovasculares (enfermedad coronaria y *stroke*) fatales y no fatales utilizando 9 cohortes con 21,378 participantes y 1,202 desenlaces. Desarrollamos un puntaje con variables de laboratorio (presión arterial sistólica, colesterol total, diabetes y tabaquismo), y un puntaje con solo variables clínicas (índice de masa corporal reemplazó al colesterol total y diabetes). Utilizamos modelos de Cox y validamos internamente los modelos calculando la pendiente de calibración y el *Harrell's C-statistic*.

**Resultados:**

El *C-statistic* para el modelo con variables de laboratorio fue 72% (70–74%) y la pendiente de calibración fue 0.994 (0.934–0.958) en hombres y 0.852 (0.761–0.942) en mujeres; para el modelo con solo variables clínicas el *C-statistic* fue 71% (69–72%) y la pendiente de calibración fue 1.028 (0.980-1.076) en hombres y 0.811 (0.663–0.958) en mujeres. En la muestra estudiada, utilizando un punto de corte de 20% de riesgo, el modelo con variables de laboratorio tuvo una sensibilidad de 21.9% y especificidad de 94.2%; cambiando el punto de corte a 10% aumentó la sensibilidad a 52.3% y redujo la especificidad a 78.7%.

**Interpretación:**

El puntaje de riesgo cardiovascular aquí desarrollado tuvo adecuada discriminación y calibración. El Globorisk-LAC sería más apropiado para ALC en comparación a los puntajes de riesgo globales y regionales. Este trabajo ofrece una herramienta que fortalece la prevención cardiovascular basada en estratificación de riesgo para ALC.

**Research in context**

**Evidence before this study**

We conducted a systematic review to identify cardiovascular risk scores developed or recalibrated in Latin America and the Caribbean (LAC); after this published systematic review, we have updated the search periodically (last on June 8th 2021). At no time did we find any cardiovascular risk score developed or adapted specifically for LAC. There are, however, two global efforts. First, the Globorisk was developed and validated with many cohorts including some from low- and middle-income countries (one from Puerto Rico). The Globorisk delivered risk charts for 182 countries. Second, the 2019 World Health Organization Cardiovascular Disease Risk Charts developed and validated their risk score from several global cohorts; unfortunately, none of these were from LAC. They delivered risk charts at the sub-region level (i.e., not for each country). This suggests that LAC did not have a region-specific cardiovascular risk prediction score to guide primary cardiovascular prevention or to quantify the burden of high cardiovascular risk.

**Added value of this study**

We developed a risk prediction equation for primary prevention of cardiovascular diseases in LAC. We also recalibrated our models and delivered risk charts for 31 countries in LAC. This work adds value to the existing evidence by providing a cardiovascular risk prediction tool specific for LAC, a world region which was neglected from previous cardiovascular risk prediction endeavors.

**Implications of all the available evidence**

Evidence showed that there were no cardiovascular risk scores specific for populations in LAC. Global efforts did not include risk estimates from LAC or did not deliver risk prediction tools at the country level. In this work, we developed a risk score for primary prevention of cardiovascular diseases exclusively for LAC; we recalibrated the model and delivered risk charts for 31 LAC countries. This work, accounting for its limitations, has provided LAC with a cardiovascular risk prediction tool which can advance cardiovascular prevention in several ways. First, pending on further validations, these models could be incorporated into local and regional cardiovascular prevention guidelines and policies; in particular, it could be adopted by the HEARTS technical package (a set of technical documents by the World Health Organization with strategies to improve cardiovascular health). Second, it could be used to quantify the burden of high cardiovascular risk in LAC; in addition, it could be used to quantify the treatment gap in LAC (i.e., high-risk people without pharmacological treatment, namely antihypertensive or lipid-lowering medication). Whether used in clinical medicine or public health for primary cardiovascular prevention, the new cardiovascular risk prediction equation for LAC will support this region to attain the 3.4 Sustainable Development Goal.

Alt-text: Unlabelled box

## Introduction

The burden of cardiovascular diseases (CVDs) in Latin America and the Caribbean (LAC) is large.^1^ While LAC countries have made good progress in implementing universal health coverage,^2^ they can focus on strengthening primary prevention of CVDs. Risk-stratification and risk-based prevention of CVDs have been deemed cost-effective across diverse populations.^3^, ^4^, ^5^ For an efficient risk-based prevention, a reliable risk score that applies to the target population should be used. However, most of the available cardiovascular risk scores^6^, ^7^, ^8^, ^9^ derived their coefficients from prospective studies conducted in high-income countries or in low-and-middle-income countries outside LAC.^10^^,^^11^ Therefore, these scores may not be applicable to LAC populations because of different socio-economic, behavioural, genetics and epidemiological profiles of patients and populations. Moreover, the ethnic composition of LAC would not be reflected in risk scores constructed for other world regions. Regional and local analysis of cardiovascular risk requires either recalibrating current risk scores to ensure that extrapolations are valid or developing a new cardiovascular risk score using data from the target population. Until recently, efforts to develop a cardiovascular risk score for LAC populations have been hampered by the small number of events in CVD cohorts from LAC.^12^ The only two global models that developed cardiovascular risk scores for LAC populations, Globorisk^7^ and the 2019 World Health Organization Cardiovascular Disease Risk Charts,^9^ borrowed information from high-income cohorts for the coefficients in their risk prediction algorithm and the latter only developed risk charts for geographic subregions within LAC (as opposed to countries).^7^^,^^9^ Therefore, there are currently no cardiovascular risk scores developed using data from prospective studies in LAC.

In this paper, leveraging on a unique data source pooled by the Cohorts Consortium of Latin America and the Caribbean (CC-LAC),^13^ we describe the development and internal validation of a cardiovascular risk score for LAC populations: Globorisk-LAC. We also provide risk charts for 31 countries in LAC by recalibrating the model to nationally representative data.

## Methods

This work adhered to the TRIPOD (Transparent reporting of a multivariable prediction model for individual prognosis or diagnosis) statement for reporting development and validation of prognostic models.^14^

### Cohorts

**Figure 1:**
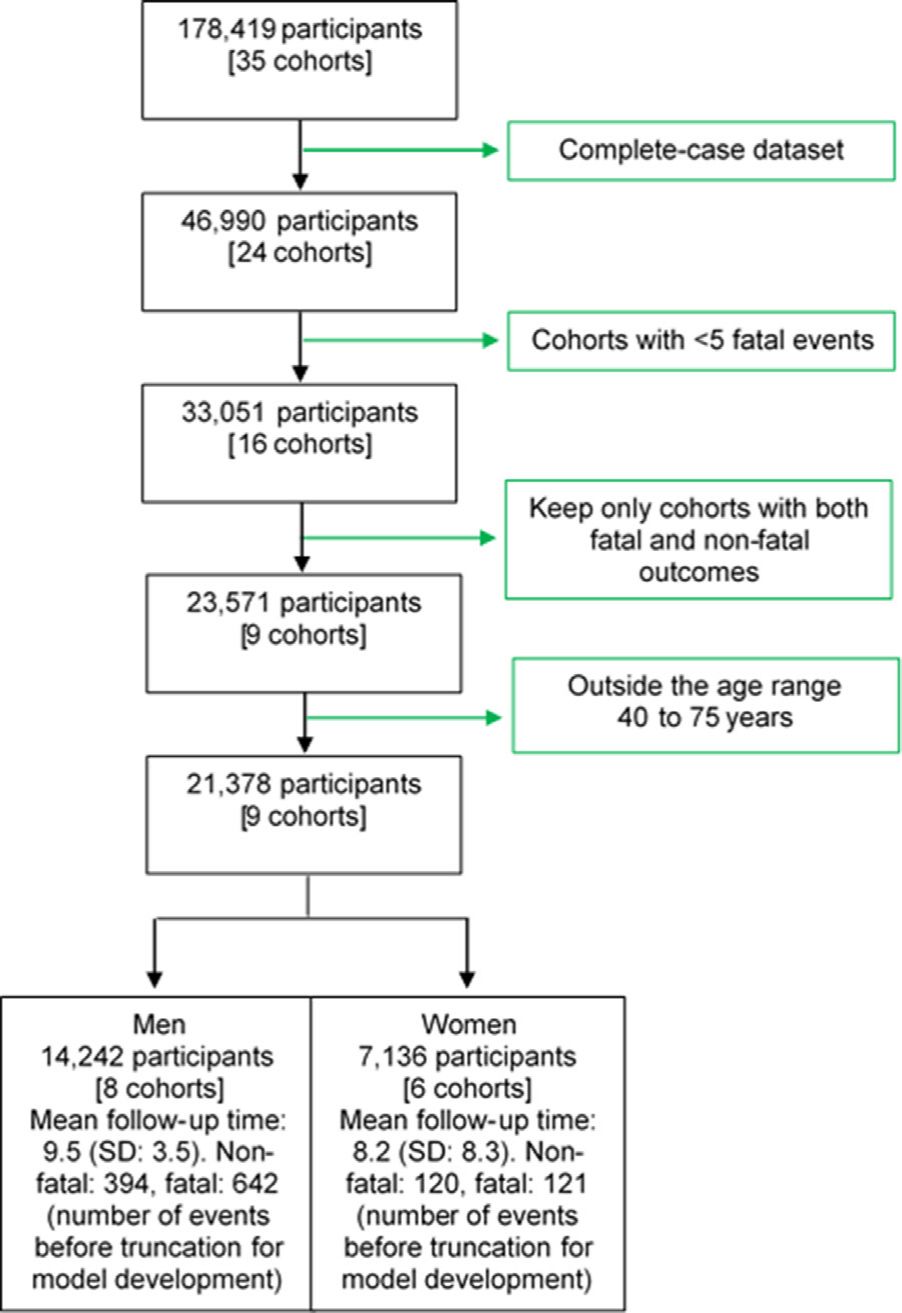
Flowchart of inclusion and exclusion of cohort participants. The original pooled dataset decreased by ∼75% (from 178,419 to 46,990 observations) mostly because a large cohort^18^ (∼115,000 people) had laboratory tests in a subsample of ∼10%. Supplementary Table1 shows summary statistics for each cohort included in the analysis

The CC-LAC is a network of health researchers and practitioners in LAC.^13^ Originally, we harmonized and pooled data from 35 CVD cohort studies identified through a systematic review and via our collaboration network. Participants in these cohorts were not recruited based on history of CVD (e.g., stroke survivors) or their high-risk status (e.g., smokers). Five cohorts included participants who attended a specific health centre^15^, ^16^, ^17^ or were members of a professional organization (e.g., The Mexican Teachers' Cohort^18^ and the Health Workers Cohort Study in Mexico^19^). The other 30 studies enrolled a random sample of the general population. In this paper, we used data from nine cohorts which met the eligibility criteria (Figure 1).

### Eligible participants

The original pooled dataset excluded participants who had self-reported history of CVDs at baseline; similarly, the original pooled dataset only included people whose cardiometabolic risk factors were within these plausible ranges: systolic blood pressure 70–270 mmHg; diastolic blood pressure 30–150 mmHg; body mass index 10–80 kg/m^2^; fasting glucose 2.5–30 mmol/l; and total cholesterol 1.75–20 mmol/l.^13^ Leveraging on this pooled dataset of prospective cohort studies in LAC, we conducted a complete-case analysis and excluded cohort studies with fewer than five fatal CVD events. Only cohort studies with information on both fatal and non-fatal cardiovascular outcomes, were included. The analysis was restricted to participants aged 40 to 75 years at baseline (Figure 1).

### Statistical analysis

#### Overview

We developed risk scores for fatal/non-fatal coronary heart disease (CHD) and stroke which we hereafter refer to as CVDs (*Supplementary Materials* p. 03). We developed a laboratory- and office-based model; the former included predictors measured in the laboratory (e.g., diabetes and total cholesterol) while the latter included only predictors that can be measured at the consultation with a physician (e.g., body mass index). The office-based model can be used in resource-poor communities where laboratories are not available.

#### Model development

To estimate the coefficients of all risk prediction equations (laboratory- and office-models), we used Cox proportional hazard regressions in which the baseline hazard was stratified by sex; also, age was the time scale in the Cox proportional hazard regression. We did not further stratify by cohort (as we did in the original Globorisk model^7^), because several cohorts had insufficient number of events to estimate the baseline hazard function. We truncated follow-up at 15 years, after which participants were administratively censored. All continuous predictors (systolic blood pressure, total cholesterol, and body mass index) were mean centred by sex. This model formulation allows country-specific recalibration with mean risk factor levels and age- and sex-specific CVD event rates, as we demonstrated in a previous global risk prediction model.^7^^,^^8^

#### Predictors

We chose a parsimonious set of predictors to which many clinicians and public health scientists would have access. These were systolic blood pressure (mmHg), total serum cholesterol (mmol/l), diabetes (no/yes, including aware and unaware: fasting glucose ≥126 mg/dl (7 mmol/l), self-reported diagnosis or treatment for diabetes), current smoker (no/yes), and body mass index (kg/m^2^). Body mass index, instead of total cholesterol and diabetes, was included in the office-based model (Table 1). All predictors were evaluated at baseline only (i.e., change in time was not analysed).

**Table 1:** Predictors included in the laboratory- and office based Globorisk-LAC models.

Laboratory-based model	Office-based model
Systolic blood pressure	Systolic blood pressure
Interaction – systolic blood pressure and age	Interaction – systolic blood pressure and age
Total cholesterol	Interaction – systolic blood pressure and sex
Diabetes (yes or no)	Body mass index
Interaction – diabetes and sex (female)	Current smoker (yes o nor)
Current smoker (yes o nor)	Interaction – current smoker and sex (female)
Interaction – current smoker and sex (female)	

Systolic blood pressure in mmHg; body mass index in kg/m^2^. The interactions refer to multiplicative interactions whereby the cardiometabolic risk factor was multiplied by sex (0=men and 1=women).

In the laboratory-based model we also included interaction terms between sex and diabetes as well as sex and smoking based on prior evidence.^20^^,^^21^ In the office-based model, the diabetes-sex interaction was not included, we instead included the interaction between systolic blood pressure and sex because the model including the latter yielded better discrimination and calibration. Furthermore, we included interactions between systolic blood pressure and age because evidence suggested that hazard ratios for cardiovascular risk factors on cardiovascular outcomes decrease with age and including this term improved prediction.^10^^,^^22^^,^^23^ In contrast, interaction terms between age and total cholesterol and smoking were not included as they did not improve discrimination or calibration. We tested several functional forms for the selected predictors, including the natural logarithm, quadratic, and interactions between predictors (e.g., systolic blood pressure and smoking). These alternative models did not substantially improve discrimination and calibration.

#### Internal validation

We used a 5-fold internal validation process. The pooled dataset (i.e., including all cohorts) was randomly split into five groups with virtually equal number of observations. First, we estimated the model coefficients in all but one group (i.e., we used four of the five groups to run the Cox model). Second, we estimated discrimination and calibration when the model was applied to the withheld group (i.e., we used the remaining group only), after recalibrating the model by replacing the baseline hazard and mean risk factor levels with those observed in the withheld group. This process was repeated until all groups were used for model internal validation.

We evaluated discrimination using Harrell's C-statistic, which assesses whether the risk prediction equation assigns higher risk to participants who experience the outcome sooner. We evaluated model calibration by comparing, separately for each sex, average predicted risk within quintiles with the observed 10-year risk (Kaplan-Meier estimator). We fitted a linear regression to the calibration plot to quantify the calibration slope by sex. The linear regression had the predicted risk as dependent variable and the observed risk as independent variable. A slope above one would suggest that observed risk was lower than the predicted risk; conversely, a slope below one would suggest that the observed risk was higher than the predicted risk. A slope of 1 would suggest perfect agreement between average predicted and observed risks.

#### Recalibration and country-specific risk charts

To recalibrate the model for each country we followed a similar procedure as in the Globorisk model.^7^^,^^8^ Briefly, we used (1) coefficients from the risk prediction model herein developed (i.e., linear predictors); (2) mean risk factor levels from global modelling analyses for each 5-year age-group by sex^24^, ^25^, ^26^, ^27^, ^28^; and (3) the baseline hazard for fatal/non-fatal CVDs that were estimated by dividing CHD and stroke death rates from 2010 by region-age-sex specific case fatality rates estimated for LAC.^29^ To generate risk charts, we calculated the 10-year CVD risk for a number of pre-specified risk factor profiles (e.g., a 40–49 year old woman in Guatemala who does not smoke, has diabetes and a specific level of systolic blood pressure, body mass index, and total cholesterol). Further details and a working example are provided in Supplementary Materials p. 04–06.

#### Comparison with other risk prediction equations

We compared our risk prediction equations with the Globorisk, because it was validated in cohorts from both high-income and middle-income countries,^7^^,^^8^ unlike other models that were developed and validated in particular populations mostly in high-income countries.^30^^,^^31^

First, we recalibrated Globorisk to our pooled cohort data by resetting the mean risk factor levels and baseline hazard to those observed in our dataset separately for men and women. Afterwards, we compared the predicted risks using the Globorisk against the observed risks by quintiles of predicted risk as well as across cells in the risk charts for the six most populous countries in the three main sub-regions in LAC (Caribbean, Central and South America). In the latter analysis, we quantified the differences in predicted risk between Globorisk and Globorisk-LAC and estimated the proportion of discordant pairs. i.e., risk factor profiles that were classified as low risk (<20%) with one model but as high-risk (≥20%) with the other, or vice-versa. With the original version of the Globorisk model recalibrated to our study population, we computed the categorical Net Reclassification Improvement (NRI) index for the two thresholds (10% and 20% predicted risk).

Second, we computed the absolute cardiovascular risk with the 2019 WHO Cardiovascular Disease Risk Charts^9^ using the Stata package developed by the authors^32^ and compared the predicted risk against the observed risk in our data to assess calibration. Of note, the Stata package did not allow us to recalibrate this model to our study population but instead uses country-specific baseline risk and average risk factor levels. For comparison purposes, we also applied the original Globorisk model without recalibration to our study population. In both cases (non-recalibrated 2019 WHO and original Globorisk), the baseline year was set at 2017.

### Role of the funding source

The funder of the study had no role in study design, data collation, data analysis, results interpretation or writing of the manuscript. RMC-L and GD had full access to all the data and had final responsibility for the decision to submit for publication.

## Results

We analysed data from 21,378 participants (14,242 men and 7136 women; Figure 1). Women had higher body mass index (28.7 kg/m^2^ vs 26.1 kg/m^2^), and higher diabetes prevalence (11.5% vs 9.2%); conversely, men were more likely to be smokers (39.1% vs 20.9%; Table 2). During a mean follow-up of 8.5 years, we observed 461 first non-fatal events and 741 fatal events not preceded by a non-fatal event (incidence rate of composite outcome = 6.6 (95% Confidence Interval (95% CI): 6.3–7.0) per 1000 person-year).

**Table 2:** Characteristics of the study population at baseline.

	Overall [*n* = 21,378]	Men [*n* = 14,242]	Women [*n* = 7,136]
Baseline age (years)	54.7 (8.1)	54.4 (7.5)	55.4 (9.1)
Body mass index (kg/m^2^)	27.0 (5.0)	26.1 (4.5)	28.7 (5.6)
Systolic blood pressure (mmHg)	134 (22.9)	134 (22.4)	133 (23.9)
Total cholesterol (mmol/l)	5.3 (1.1)	5.3 (1.1)	5.3 (1.2)
Diabetes Mellitus (%)	10.0	9.2	11.5
Smoker (yes, %)	33.0	39.1	20.9

Numeric variables are summarized with mean and standard deviation. Smoker refers to current smoker versus non-smoker. Diabetes includes self-reported or fasting plasma glucose ≥126 mg/dl. All comparisons between men and women were significant at p<0.001; numeric variables (age, body mass index, systolic blood pressure and total cholesterol) were compared with t-tests and categorical variables (diabetes and smoking) with chi-2 tests.

In the laboratory-based model, higher SBP, higher total cholesterol, diabetes and smoking were strongly associated with CVD risk. In the office-based model, higher SBP and smoking were strongly associated with CVD risk. In both models, the association between SBP and cardiovascular events decreased with age (Table 3). At 63 years of age (mean age at event in the pooled cohort population), the hazard ratios herein computed were generally consistent and between 3% higher to 12% lower than that of the original Globorisk model (Table 3).

**Table 3:** Coefficients (log hazard ratio and 95% confidence intervals) from the sex-stratified proportional hazard regressions for laboratory- and office-based models for fatal/nonfatal CHD or stroke (CC-LAC cohorts, *N* = 21,378 and 1202 events).

Predictors (unit/reference group)	Globorisk-LAC		Original Globorisk	
	Laboratory-based model	HR	Laboratory-based model	HR
SBP (per 10 mmHg)	0.4189 (0.2562; 0.5815)	1.227	0.3070	1.176
Interaction between SBP and age (per 10 mmHg for 1 year)	-0.0034 (-0.0058; -0.0009)		-0.0023	
Total cholesterol (per 1 mmol/l)	0.1203 (0.0743; 0.1662)	1.128	0.6149	1.197
Interaction between total cholesterol and age (per 1 mmol/l for 1 year)			-0.0069	
Diabetes	0.6691 (0.5080; 0.8303)	1.952	1.4753	1.904
Interaction between diabetes and age			-0.0132	
Interaction between diabetes and sex (female)	0.1024 (-0.2857; 0.5825)	1.108	0.4051	1.499
Smoker (current)	0.3268 (0.2014; 0.4521)	1.387	1.8467	1.575
Interaction between smoker and age			-0.0221	
Interaction between smoker and sex (female)	0.1469 (-0.2887; 0.5825)	1.158	0.3254	1.385
	**Office-based model**		**Office-based model**	
SBP (per 10 mmHg)	0.4377 (0.2725; 0.6030)	1.243	0.3037	1.187
Interaction between SBP and age (per 10 mmHg for 1 year)	-0.0035 (-0.0061; -0.0010)		-0.0021	
Body mass index (per 5 kg/m^2^)	0.0495 (-0.0160; 0.1151)	1.051	0.3245	1.145
Interaction between body mass index and age (per 5 kg/m^2^ for 1 year)			-0.0030	
Smoker (current)	0.3083 (0.1816; 0.4350)	1.361	1.7951	1.554
Interaction between smoker and age			-0.0215	
Interaction between smoker and sex (female)	0.1843 (-0.2518; 0.6203)	1.202	0.3528	1.423
Interaction between systolic blood pressure (per 10 mmHg) and sex (female)	0.0069 (-0.0505; 0.0643)	1.007		

SBP=systolic blood pressure; HR=hazard ratios. Blank cells because the Globorisk-LAC model did not include those age interactions. The Cox regression model included age as the time scale; age was not centred in the regression models. Therefore, HR for age interactions was computed at age 63, which was the mean age at event. The coefficients of HR for 2019 WHO Cardiovascular Disease Risk Charts^9^ were not included in the table because these were reported by sex unlike those herein shown which were for both men and women.

**Figure 2:**
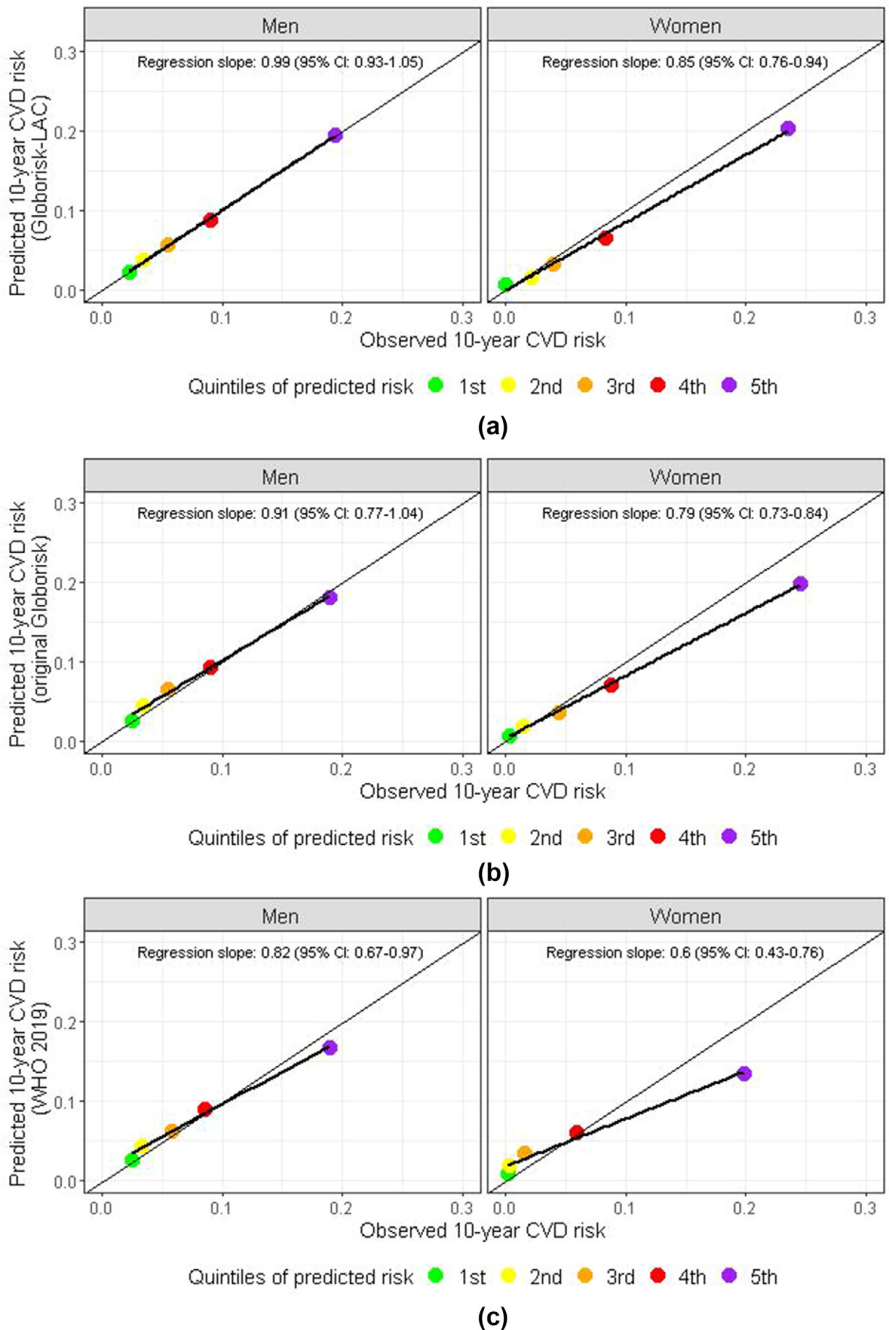
Calibration plots for the 10-year risk of fatal/non-fatal CHD or stroke for laboratory-based models: (A) Globorisk-LAC, (B) original Globorisk and (C) 2019 WHO Cardiovascular Disease Risk Charts. The reported regressions slopes represent the coefficient and 95% confidence interval of a univariate linear in which the dependent (y) variable was the predicted risk and the independent (x) variable was the observed risk. To compute the absolute risk with the 2019 WHO Cardiovascular Risk Charts we used the Stata package developed by the authors; the diabetes indicator we used was total diabetes (unaware plus aware).

The Harrell's C-statistic for the laboratory-based model was 72% (95% CI: 70–74%) and the calibration regression slope was 0.852 (95% CI: 0.761–0.942) among women and 0.994 (95% CI: 0.934–1.055) among men, suggesting 15% underestimation of 10-year risk in women and reasonable agreement in men (Table 4 and Figure 2A). At a threshold of 20% risk, the sensitivity was 21.9% and specificity was 94.2%. For a threshold of 10%, sensitivity was 52.3% and specificity 78.7%. As an example, the predicted 10-year risk of CVD for a 60-year-old woman who is a smoker and has diabetes, a systolic blood pressure of 140 mmHg and total cholesterol of 5 mmol/l, ranged from 10% in Chile to 42% in Guyana (median=23%, p25=18%, p75=27%) (Supplementary Figure 2). A man with the same profile would have a 10-year predicted risk ranging from 15% in Chile to 46% in Guyana (median=28%, p25=22%, p75=32%). The 10-year predicted risk was consistently higher in men, except in two countries (Bolivia and Paraguay). The full set of risk charts are presented in Supplementary Figure 4 (laboratory-based) and Supplementary Figure 5 (office-based).

**Table 4:** Discrimination (Harrell's c-statistic) and calibration (regression coefficient for quintiles of predicted versus observed risk) for 5-fold internal validation for fatal/non-fatal CHD or stroke.

Iteration	C-statistic (95% CI)	Calibration regression slope (95% CI)	
		Men	Women
**Laboratory-based**			
Iteration 1	71% (67–75%)	1.020 (0.826–1.214)	0.406 (0.217–0.596)
Iteration 2	73% (69–77%)	0.973 (0.838–1.109)	1.371 (0.672–2.070)
Iteration 3	73% (69–76%)	0.890 (0.742–1.039)	0.840 (0.610–1.070)
Iteration 4	74% (70–78%)	1.078 (0.548–1.608)	0.559 (0.371–0.747)
Iteration 5	69% (64–73%)	1.067 (0.782–1. 523)	0.747 (0.588–0.907)
All observations	72% (70–74%)	0.994 (0.934–1.055)	0.852 (0.761–0.942)
**Office-based**			
Iteration 1	70% (66–74%)	0.985 (0.795–1.175)	0.389 (0.258–0.520)
Iteration 2	72% (68–76%)	0.994 (0.783–1.205)	0.963 (0.259–1.667)
Iteration 3	70% (66–74%)	0.969 (0.772–1.167)	0.782 (0.228–1.335)
Iteration 4	73% (68–77%)	0.920 (0.795–1.045)	0.539 (0.518–0.559)
Iteration 5	68% (64–72%)	1.130 (0.953–1.308)	0.798 (0.511–1.084)
All observations	71% (69–72%)	1.028 (0.980–1.076)	0.811 (0.663–0.958)

The Cox proportional hazard model to derive the coefficients was conducted in all but partition X (X in 1, 2, 3, 4, 5), and the Harrell's C-statistic (95% confidence interval) as well as the calibration regression slopes (95% confidence interval) were computed in partition X alone after recalibrating (i.e. replacing the baseline hazard and mean risk factor levels).

The Harrell's C-statistic for the office-based model was 71% (95% CI: 69–72%), and the calibration regression slope was 0.811 (95% CI: 0.663–0.958) among women and 1.028 (95% CI: 0.980–1.076) in men, suggesting underestimation of the observed risk among women and reasonable agreement in men (Table 4 and Supplementary Figure 1A). At a threshold of 20% risk, sensitivity was 19.4% and specificity 94.9%; for a threshold of 10%, sensitivity was 49.6% and specificity, 78.5%.

The predicted risk using Globorisk-LAC was closer to the observed risk (Figure 2A) compared with the original Globorisk model after recalibration to the pooled study population (Figure 2B). The latter underestimated the observed risk by 9% in men and by 21% in women. The original Globorisk office-base model underestimated the risk by 11% in men and 28% in women (Supplementary Figure 1B).

When examining the non-recalibrated models, the predicted risk using the Globorisk-LAC was closer to the observed risk compared to the 2019 WHO Cardiovascular Disease Risk Charts (Figure 2**C**) which underestimated the risk by 18% among men and 40% among women; the office-based model of the 2019 WHO Cardiovascular Disease Risk Charts underestimated the risk by 14% in men and 46% in women (Supplementary Figure 1C). The non-recalibrated original laboratory-based Globorisk model overestimated the risk by 23% in men and 17% in women (Supplementary Figure 3A) while the office-based model overestimated it by 18% in men and 14% in women (Supplementary Figure 3B).

Across 6560 possible risk factor profiles (i.e., cells in risk charts) and using a 20% risk threshold, discrepancies in high-risk status between Globorisk and Globorisk-LAC were small. The proportion of discordant risk factor profiles among men ranged from 3% in Mexico to 5% in Guatemala and Haiti. Among women, the same proportion ranged from 7% in Cuba and Mexico to 10% in Guatemala (Supplementary Table 2). When the risk threshold was set at 10%, the proportions of discordant points were generally smaller compared to those calculated using a 20% threshold.

Regarding the NRI metrics at a 20% predicted risk threshold, both the laboratory- and office-based Globorisk-LAC models correctly classified more high-risk individuals than the original Globorisk models recalibrated to our study population. At a 10% predicted risk threshold, the Globorisk-LAC models did not substantially reclassify more high-risk individuals than the original Globorisk model recalibrate to our population (Supplementary Table 3).

## Discussion

We developed laboratory- and office-based cardiovascular risk prediction equations for populations in LAC using data from local cohort studies and provided risk charts for the 31 countries in the region. The decision on whether to use the laboratory-based or office-based model should be mostly informed by the availability of laboratory resources. We delivered a pragmatic tool to support primary cardiovascular prevention in LAC and to facilitate achieving the 3.4 Sustainable Development Goal^33^ by targeting people at high cardiovascular risk. In internal validations, our models showed acceptable discrimination and calibration metrics. The new model had reasonable discrimination and calibration and performed slightly better than our own previous global model^7^ especially among men. At a threshold of 10% for 10-year risk, the model had appropriate sensitivity and specificity profile to detect individuals with high cardiovascular risk in the pooled sample.

For both the laboratory- and office-based models, the sensitivity substantially increased (from ∼20% to ∼51%) when the 10-year predicted risk threshold changed from 20% to 10%. This suggests that lowering the 10-year predicted risk threshold would lead to detecting more cases, i.e., true positives. The same threshold change led to a smaller decline in specificity (from ∼94% to ∼79%). In other words, when assessing cardiovascular risk among 100 people who will not have a cardiovascular event, using a threshold of 20% would incorrectly classify five people as high-risk compared with 21 people when using a 10% threshold. This may have pragmatic implications because with a 10% threshold, more people would require additional resources, e.g., counselling or treatment. Defining the best threshold to define high cardiovascular risk, whether 7.5%, 10% or 20% of predicted 10-year cardiovascular risk, would depend on the capacity of the health system to provide adequate care for those individuals who are correctly identified at high cardiovascular risk while avoiding unnecessary burden of testing on individuals who are incorrectly classified as high-risk.

The calibration plots showed non-optimal performance in women in the highest quintile of predicted risk. Risk factors not included in our model could explain this finding. For example, hormone replacement therapy may have a positive impact in the model calibration.^34^ In addition, sex-differences in access to treatment for the prevention or management of cardiometabolic risk factors^35^ could also explain this finding.

The application of our laboratory-based model in a clinical vignette showed large differences between the countries with the lowest and highest predicted risk. This is, probably, a consequence of the underlying risk factor distribution and rates of cardiovascular diseases in these populations. For example, the mean total cholesterol in men was higher in Guyana (5.0 mmol/l) than in Chile (4.6 mmol/l);^36^ similarly, the mean SBP was higher in men in Guyana (124 mmHg) than in Chile (120 mmHg).^25^ More importantly, the age-standardized cardiovascular mortality rate in Guyana is 3.5 times the rate in Chile (447 vs 126 per 100,000).^37^

Previous efforts in LAC to study or recalibrate cardiovascular risk scores were limited by a small number of outcome events and were conducted in one or few countries;^12^ also, the coefficients from those models were derived from non-LAC cohorts limiting their extrapolation to populations in LAC. The previous two global endeavours to develop CVD risk scores: the Globorisk^7^^,^^8^ and the 2019 World Health Organization Cardiovascular Disease Risk Charts^9^ share the latter limitation, i.e., using coefficients from cohorts conducted in other regions.

We benefited from the largest cohort data pooling project in LAC,^13^ overcoming many of the limitations faced by individual cohorts trying to assess, and possibly recalibrate, cardiovascular risk prediction equations in LAC.^12^ We used standard methods to develop the risk prediction coefficients^6^^,^^30^^,^^31^^,^^38^ and combined these coefficients with population-based estimates on mean levels of cardio-metabolic risk factors for 31 countries in LAC and the best evidence on CVD event rates. We used a Cox Proportional Hazard model with age as the time scale, which allows recalibration using age-sex-specific CVD rates from national sources or global estimates. We included interactions with age, which prevents overestimation in older ages. We developed an office-based model with reasonable discrimination and calibration using body mass index. Finally, to generate risk charts, we recalibrated the risk prediction equation for each country, using contemporary data on both cardiometabolic risk factor levels and CVD rates. The only parameters taken from the cohorts were the *proportional* associations (i.e., log hazard ratios) between risk factors and CVD rates, which we would not expect to have changed substantially overtime.

Limitations of this work should be acknowledged. First, we did not include some predictors with strong association with CVDs because data on these factors are not routinely available in cohort studies and population health surveys. For example, we did not include non-HDL- or LDL-cholesterol because data on these biomarkers were limited; had we used these biomarkers instead of total cholesterol, the sample size would have been reduced. However, the fact that our model included total cholesterol instead of HDL- or LDL-cholesterol would improve the uptake of our model in rural or resource-limited settings where laboratories may only have resources to measure total cholesterol. This rationale was also fallowed by the Globorisk and the 2019 WHO Cardiovascular Disease Risk Charts. Other risk scores have also included predictors regarding treatment (e.g., antihypertensive treatment), but compliance with treatment varies greatly across populations and there may be other indications for antihypertensive medications, making these data unreliable for risk prediction in this setting. Second, in the analysis we included ∼25% of the original sample. This is because some pooled cohorts did not have data on the predictors of interest. For example, a Mexican cohort of ∼115,000 people only collected blood biomarkers (e.g., total cholesterol) in a subsample of ∼10%.^18^ Third, we could not conduct an external validation because saving data from a few cohorts just for external validation would have reduced the number of events used for model estimation. Future work, and other cohorts in LAC, could independently validate our model. Fourth, for comparison purposes with the two previous global cardiovascular risk models we used the 2019 WHO Cardiovascular Disease Risk Charts and the original Globorisk model, but we could not recalibrate the former to our study population. To make the comparisons fair, we provided another set of results for the original Globorisk model without recalibration. These comparisons showed that the global models performed well but could over/underestimate risk by more than 10%.

Health systems need to identify individuals who are at high cardiovascular risk to focus their limited resources on more efficient primary prevention and treatment allocation. A reliable risk stratification tool to identify people at high risk of CVDs is key to achieve the Sustainable Development Goal 3.4 in LAC.^33^ However, available cardiovascular risk prediction equations that use coefficients from other populations could provide biased risk predictions in LAC. While countries in LAC transition to universal health coverage, monitoring the proportion of high-risk individuals with and without access to treatment is crucial to measure progress toward the World Health Organization's target of treating at least 50% of people aged ≥40 years with cardiovascular risk ≥30%.^39^ The Globorisk-LAC model provides a new tool to monitor the number of people at high-cardiovascular risk and the treatment gap; that is, the proportion of people at high cardiovascular risk not receiving treatment.

## Contributors

RMC-L, JJM and ME conceived the CC-LAC with support from all members in the steering committee. RMC-L, GD, JJM and ME conceived this study. RMC-L harmonized the dataset and conducted the analysis with support from GD. RMC-L and GD drafted the first version of the manuscript. All authors provided critical input and approved the final version.

## Data sharing

Data cannot be shared outside the Cohorts Consortium of Latin America and the Caribbean (CC-LAC). Analysis codes are available as supplementary files. The original Globorisk models and the Globorisk-LAC models are available in a R package. Please, go to this repository for further instructions on how to install and use: www.globorisk.org/code Please, refer to the scientific publications for further details about these cardiovascular risk prediction equations.

**^†^Cohorts Consortium of Latin America and the Caribbean (CC-LAC)**

Pooled Analysis and Writing

Rodrigo M Carrillo-Larco (Imperial College London, UK); Dalia Stern (National Institute of Public Health, Mexico); Ian R Hambleton (The University of the West Indies, Barbados); Paulo Lotufo (University of São Paulo, Brazil); Mariachiara Di Cesare (University of Essex, UK); Anselm Hennis (Pan American Health Organization, USA); Catterina Ferreccio (Pontificia Universidad Católica de Chile, Advanced Centre for Chronic Diseases ACCDiS, Chile); Vilma Irazola (Institute for Clinical Effectiveness and Health Policy, Argentina); Pablo Perel (London School of Hygiene and Tropical Medicine, UK); Edward W Gregg (Imperial College London, UK); J Jaime Miranda (Universidad Peruana Cayetano Heredia, Peru); Majid Ezzati (Imperial College London, UK); Goodarz Danaei (Harvard TH Chan School of Public Health, USA)

Country and Regional Data (* equal contribution; listed alphabetically by surname)

Carlos A Aguilar-Salinas (Instituto Nacional de Ciencias Médicas y Nutrición, Mexico)*; Ramón Alvarez-Váz (Universidad de la Republica, Uruguay)*; Marselle B Amadio (Centro Universitario Senac Santo Amaro, Brazil)*; Cecilia Baccino (Universidad de la Republica, Uruguay)*; Claudia Bambs (Pontificia Universidad Católica de Chile, Chile)*; João Luiz Bastos (Universidade Federal de Santa Catarina, Brazil)*; Gloria Beckles (Centers for Disease Control and Prevention, USA)*; Antonio Bernabe-Ortiz (Universidad Peruana Cayetano Heredia, Peru)*; Carla DO Bernardo (The University of Adelaide, Australia)*; Katia V Bloch (Universidade Federal do Rio de Janeiro (UFRJ), Brazil)*; Juan E Blümel (Universidad de Chile, Chile)*; Jose G Boggia (Universidad de la República, Uruguay)*; Pollyanna K Borges (Universidade Estadual de Ponta Grossa, Brazil)*; Miguel Bravo (MELISA Institute, Chile)*; Gilbert Brenes-Camacho (Universidad de Costa Rica, Costa Rica)*; Horacio A Carbajal (Universidad Nacional de la Plata, Argentina)*; Paola Casas-Vasquez (Universidad Peruana Cayetano Heredia, Peru)*; Maria S Castillo Rascon (Universidad Nacional de Misiones, Argentina)*; Blanca H Ceballos (Hospital Dr Ramon Madariaga, Argentina)*; Veronica Colpani (Federal University of Rio Grande do Sul, Brazil)*; Jackie A Cooper (Queen Mary University of London, UK)*; Sandra Cortes (Pontificia Universidad Católica de Chile, Chile)*; Adrian Cortes-Valencia (National Institute of Public Health, Mexico)*; Roberto S Cunha (Federal University of Espírito Santo, Brazil)*; Eleonora d'Orsi (Universidade Federal de Santa Catarina, Brazil)*; William H Dow (University of California, Berkeley, USA)*; Walter G Espeche (Universidad Nacional de la Plata, Argentina)*; Flavio D Fuchs (Universidade Federal do Rio Grande do Sul, Brazil)*; Sandra C Fuchs (Universidade Federal do Rio Grande do Sul, Brazil)*; Suely GA Gimeno (Universidade Federal de São Paulo, Brazil)*; Donaji Gomez-Velasco (Instituto Nacional de Ciencias Médicas y Nutrición, Mexico)*; David A Gonzalez-Chica (The University of Adelaide, Australia)*; Clicerio Gonzalez-Villalpando (Instituto Nacional de Salud Pública, México)*; María-Elena Gonzalez-Villalpando (Centro de Estudios en Diabetes A.C., México)*; Gonzalo Grazioli (Hospital Churruca Visca, Argentina)*; Ricardo O Guerra (Federal University of Rio Grande do Norte, Brazil)*; Laura Gutierrez (Institute for Clinical Effectiveness and Health Policy, Argentina)*; Fernando L Herkenhoff (Federal University of Espírito Santo, Brazil)*; Andrea RVR Horimoto (University of São Paulo, Brazil)*; Andrea Huidobro (Universidad Católica del Maule, Chile)*; Elard Koch (MELISA Institute, Chile)*; Martin Lajous (Harvard T.H. Chan School of Public Health, USA; National Institute of Public Health, Mexico)*; Maria Fernanda Lima-Costa (Oswaldo Cruz Foundation, Brazil)*; Ruy Lopez-Ridaura (National Institute of Public Health, Mexico)*; Alvaro CC Maciel (Federal University of Rio Grande do Norte, Brazil)*; Gladys E Maestre (University of Texas Rio Grande Valley, USA)*; Betty S Manrique-Espinoza (National Institute of Public Health, Mexico)*; Larissa P Marques (Universidade Federal de Santa Catarina, Brazil)*; Jesus D Melgarejo (University of Leuven, Belgium)*; Luis J Mena (Universidad Politécnica de Sinaloa, México)*; Jose G Mill (Federal University of Espírito Santo, Brazil)*; Leila B Moreira (Universidade Federal do Rio Grande do Sul, Brazil)*; Oscar M Muñoz (Pontificia Universidad Javeriana, Hospital Universitario San Ignacio, Colombia)*; Lariane M Ono (Universidade Federal do Paraná, Brazil)*; Karen Oppermann (Passo Fundo University, Brazil)*; Pedro J Ortiz (Universidad Peruana Cayetano Heredia, Peru)*; Karina M Paiva (Universidade Federal de Santa Catarina, Brazil)*; Sergio V Peixoto (Oswaldo Cruz Foundation, Brazil)*; Alexandre C Pereira (University of São Paulo, Brazil)*; Karen G Peres (NDRIS/NDCS Duke-NUS Medical School, Singapore)*; Marco A Peres (NDRIS/NDCS Duke-NUS Medical School, Singapore)*; Paula Ramírez-Palacios (IMSS Epidemiology and Health Services Research Unit, Mexico)*; Cassiano R Rech (Universidade Federal de Santa Catarina, Brazil)*; Berenice Rivera-Paredez (National Autonomous University of Mexico, Mexico)*; Nohora I Rodriguez (Clinica de Marly, Colombia)*; Rosalba Rojas-Martinez (Instituto Nacional de Salud Pública, México)*; Luis Rosero-Bixby (Universidad de Costa Rica, Costa Rica)*; Adolfo Rubinstein (Institute for Clinical Effectiveness and Health Policy, Argentina)*; Alvaro J Ruiz (Pontificia Universidad Javeriana, Colombia)*; Martin R Salazar (Universidad Nacional de la Plata, Argentina)*; Aaron Salinas-Rodriguez (National Institute of Public Health, Mexico)*; Jorge Salmerón (National Autonomous University of Mexico, Mexico)*; Ramon A Sanchez (Universidad Nacional de Misiones, Argentina)*; Nelson AS Silva (Universidade Federal do Rio de Janeiro (UFRJ), Brazil)*; Thiago LN Silva (Universidade Federal do Rio de Janeiro (UFRJ), Brazil)*; Liam Smeeth (London School of Hygiene & Tropical Medicine, UK)*; Poli M Spritzer (Federal University of Rio Grande do Sul, Brazil)*; Fiorella Tartaglione (Hospital Churruca Visca, Argentina)*; Jorge Tartaglione (Hospital Churruca Visca, Argentina)*; Tania Tello (Universidad Peruana Cayetano Heredia, Peru)*; Rafael Velázquez-Cruz (National Institute of Genomic Medicine (INMEGEN), Mexico)*

## Declaration of interests

ME reports a charitable grant from the AstraZeneca Young Health Programme, and personal fees from Prudential, outside the submitted work. All other authors declare no competing interests.

## Appendix Supplementary materials

Image, application 1

Image, application 2

Image, application 3
