# Supplementary material for: Derivation, internal validation, and recalibration of a cardiovascular risk score for Latin America and the Caribbean (Globorisk-LAC): A pooled analysis of cohort studies
Source: Lancet Reg Health Am. 2022 Apr 23;9:100258. doi: 10.1016/j.lana.2022.100258 (PMC9107390; doi:10.1016/j.lana.2022.100258)
Supplement: Supplementary file 1 [file mmc1.docx]

**Globorisk-LAC: Derivation, internal validation, and recalibration of a cardiovascular risk score for Latin America and the Caribbean: a pooled analysis of cohort studies**

**Corresponding Author**

Goodarz Danaei

Bernard Lown Associate Professor of Cardiovascular Health

Harvard T.H. Chan School of Public Health

Address: 677 Huntington Avenue, Building 1, 11th Floor, Room 1107, Boston, MA 02115

Email: [gdanaei@hsph.harvard.edu](mailto:gdanaei@hsph.harvard.edu)

[Expanded Methods 3](#_Toc93636054)

[Cardiovascular fatal outcomes 3](#_Toc93636055)

[Recalibration process 4](#_Toc93636056)

[Supplementary Table 1: Characteristics of cohorts included in the analysis. 6](#_Toc93636057)

[Supplementary Figure 1: Calibration plots for the (A) fatal/non-fatal events office-based Globorisk-LAC model, (B) fatal/non-fatal events office-based Globorisk model and (C) 2019 WHO Cardiovascular Risk Charts. 7](#_Toc93636058)

[Supplementary Table 1: Percentage (%) of discordant points between Globorisk-LAC and original Globorisk model at a 20%/10% 10-year predicted risk threshold. 9](#_Toc93636059)

[References 10](#_Toc93636060)

[Supplementary Figure 2: 10-year predicted cardiovascular risk per country based on the Globorisk-LAC model for a men/women who is smoker and has diabetes, a systolic blood pressure of 140 mmHg and total cholesterol of 5 mmol/L. 11](#_Toc93636061)

[Supplementary Figure 3: Calibration plots for (A) fatal/non-fatal events laboratory-based non-recalibrate original Globorisk model and (B) fatal/non-fatal events office-based non-recalibrated original Globorisk model. 12](#_Toc93636062)

[Supplementary Table 3: Net reclassification improvement index 13](#_Toc93636063)

# **Expanded Methods**

## **Cardiovascular fatal outcomes**

Cohorts herein analysed provided causes of death based on strong ascertainment methods, these included death certificates and adjudication.^1^ When ICD-10 codes were available, fatal cardiovascular events included: I21.2; I21.9 (or I21); I24.8; I25.5; I60.9 (or I60); I61.5; I61.9 (or I61); I62.0 (or I62); I62.9; I63.3 (or I63); I63.5; I63.9; I64; I67.8; I67.9. When ICD-9 codes were provided, we included: 410; 430; 431; 436. If the outcomes were recorded as free text, we included: fatal stroke (haemorrhagic, ischaemic or not specified), fatal myocardial infarction (heart attack), sudden death or acute cardiac death, cardiovascular death, and non-specified coronary disease. Equivalent conditions were included for non-fatal outcomes.

#

# **Recalibration process**


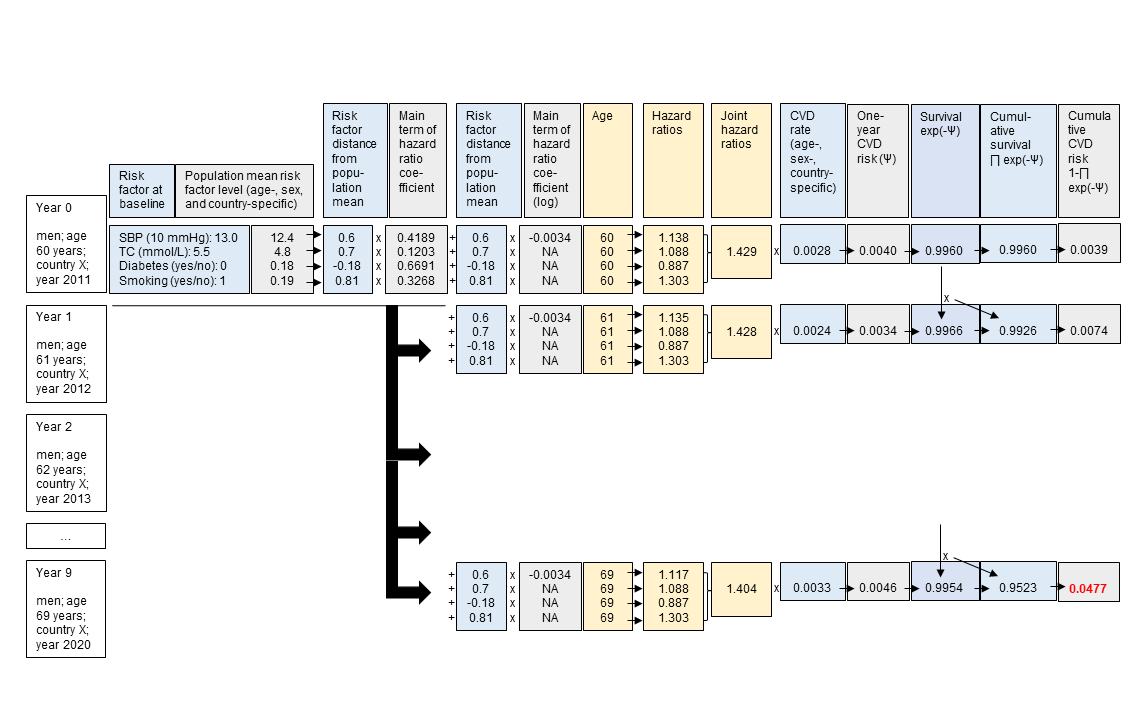


With a clinical vignette we detail the recalibration process. There is a man, 60 years of age, in 2011 (year 0). His systolic blood pressure is 130 mmHg; his total cholesterol is 5.5 mmol/L; he does not have diabetes; and he currently smokes. In his country, age and sex group, the mean systolic blood pressure is 124 mmHg; the mean total cholesterol is 4.8 mmol/L; the prevalence of diabetes is 18%; and the prevalence of smoking is 19%. These mean estimates at the population level are available from population-based surveys in each country.

First, we calculate the difference between this patient’s cardiometabolic profile and the mean in his country, age and sex group. For example, we subtract 12.0 from 12.4. For systolic blood pressure, his distance from the population mean is 0.6. We repeat the same calculation for all predictors in the model.

Second, the distance from the mean in the population is multiplied by the main coefficient of the corresponding risk factor. For systolic blood pressure for example, we multiply 0.6 (the distance) by 0.4189, which is the main coefficient from the Cox regression model for systolic blood pressure (Table 3 in main manuscript). The same process is repeated for each predictor.

Third, the distance from the mean in the population is multiplied by the time interaction term and by age. For systolic blood pressure for example, we multiply 0.6 (distance) by -0.0034 (age interaction for systolic blood pressure; Table 3 in main manuscript) and by 60 (age at year 0). In the Globorisk-LAC laboratory-based model we do not have age interactions for the other cardiometabolic risk factors, thus these are not available (NA).

Fourth, we sum the products from the second and third steps; this would be the log hazard ratio for each predictor in the model and should be exponentiated to calculate the hazard ratio. Fifth, we multiply all the hazard ratios (for systolic blood pressure x total cholesterol x diabetes x smoking) to compute the joint hazard ratio.

Sixth, the joint hazard ratio is multiplied by the cardiovascular disease (CVD) rates in the population (country-, sex-, and age-specific). This product gives the one-year cardiovascular risk.

Seventh, we calculate the survival as the exponential function of the negative value of the one-year CVD risk.

Eight, we computer the cumulative survival. For year 0, the cumulative survival is the same as the survival (step #7). For the following years, the cumulative survival is the product between the survival in year T and the survival in year T-1.

Ninth, the cumulative CVD risk is calculated as one minus the cumulative survival. The cumulative CVD risk in the last year (year 9) is the absolute 10-year predicted cardiovascular risk for this subject. In this working example, the absolute 10-year predicted cardiovascular risk was 0.0477 or 4.7% of having a CVD in a 10-year period.

This process was repeated for each country. This working example is made for the laboratory-based model, though the same process applies to the office-based model. Except for the coefficients which are those shown in Table 3 in the main manuscript, all other numbers in this working examples were made up for illustrative purposes only. This process and figure were adapted from previous publications.^[[1]](#footnote-1),^^[[2]](#footnote-2)^

# **Supplementary Table 1:** Characteristics of cohorts included in the analysis.

| **Cohort** | **Country** | **Baseline year** | **Sample size** | **Age** | **SBP** | **TC** | **BMI** | **Diabetes** | **Smoking** |
| --- | --- | --- | --- | --- | --- | --- | --- | --- | --- |
| Revista Argentina de Cardiología. 2008;76(5):347-351. | Argentina | 1997 | 1257 | 46.9 | 135.5 | 5.6 | 29.1 | 78 | 527 |
| Am J Hypertens. 2014;27(8):1061-8. | Argentina | 1997 | 584 | 55.1 | 143.0 | 5.0 | 27.2 | 28 | 139 |
| BMJ Open. 2011;1(1):e000126. | Argentina | 2010 | 3401 | 56.0 | 130.7 | 5.3 | 29.4 | 398 | 894 |
| Maturitas. 2003;45(3):205-12. | Chile | 1991 | 1196 | 48.5 | 120.1 | 5.7 | 25.9 | 41 | 182 |
| BMC Public Health. 2016;16:122. | Chile | 2014 | 3072 | 56.7 | 132.3 | 5.0 | 29.8 | 485 | 793 |
| Revista Colombiana de Cardiología. 2014;21(4):202-212. | Colombia | 1984 | 778 | 54.9 | 142.0 | 6.1 | 27.2 | 43 | 66 |
| Puerto Rico Heart Health Program (PRHHP) | Puerto Rico | 1965 | 9550 | 54.4 | 132.2 | 5.2 | 25.1 | 782 | 4178 |
| Int J Epidemiol. 2005;34(6):1387-94. | Trinidad and Tobago | 1977 | 539 | 54.1 | 136.4 | 5.7 | 23.9 | 65 | 86 |
| Neuroepidemiology 2007;29:107–112. | Venezuela | 2000 | 1001 | 64.0 | 163.8 | 5.1 | 27.7 | 208 | 189 |

BMI: body mass index (kg/m^2^); SBP: systolic blood pressure (mmHg); TC: total cholesterol (mmol/L). Age, BMI, SBP and TC are shown as means. Sample size, diabetes and current smoking are shown as absolute numbers.

# **Supplementary Figure 1:** Calibration plots for the (A) fatal/non-fatal events office-based Globorisk-LAC model, (B) fatal/non-fatal events office-based Globorisk model and (C) 2019 WHO Cardiovascular Risk Charts.

**A**


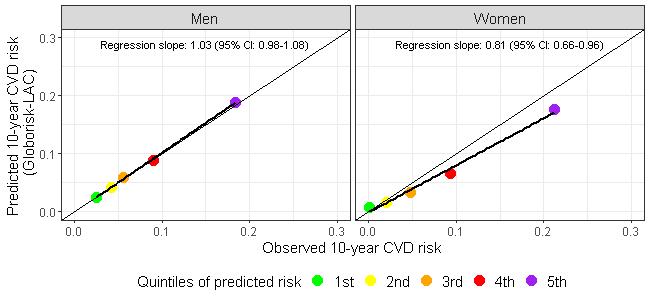


**B**


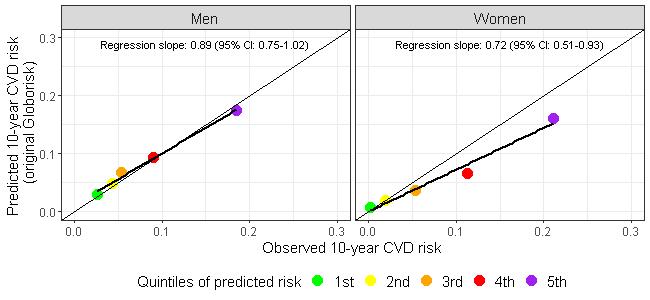


**C**


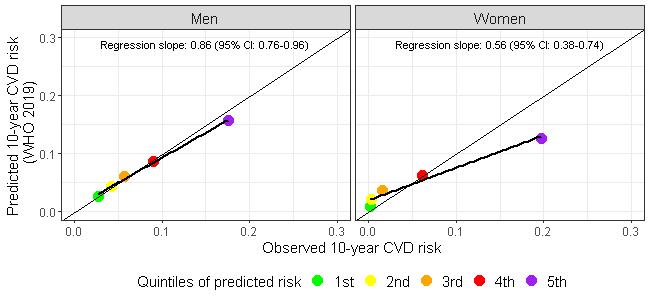


The numbers represent the beta coefficients and 95% confidence interval of a simple lineal regression in which the dependent (y) variable was the predicted risk and the independent (x) variable was the observed risk.

# **Supplementary Table 2:** Percentage (%) of discordant points between Globorisk-LAC and original Globorisk model at a 20%/10% 10-year predicted risk threshold.

| **Low risk with Globorisk-LAC but high risk with Globorisk** | | | | |
| --- | --- | --- | --- | --- |
| **Fatal/non-fatal cardiovascular events laboratory-based model** | | | | |
|  | **Men (%)** | **Women (%)** | **Diabetes (%)** | **Smoker (%)** |
| Brazil | 4/5 | 9/7 | 10/6 | 10/8 |
| Colombia | 4/3 | 9/5 | 9/3 | 10/5 |
| Cuba | 4/3 | 7/5 | 8/5 | 9/6 |
| Guatemala | 5/5 | 10/7 | 11/5 | 12/8 |
| Haiti | 5/3 | 9/5 | 9/2 | 11/5 |
| Mexico | ¾ | 7/7 | 9/6 | 9/8 |

| **High risk with Globorisk-LAC but low risk with Globorisk** | | | | |
| --- | --- | --- | --- | --- |
| **Fatal/non-fatal cardiovascular events laboratory-based model** | | | | |
|  | **Men (%)** | **Women (%)** | **Diabetes (%)** | **Smoker (%)** |
| Brazil | 5/2 | 1/2 | 3/1 | 3/0 |
| Colombia | 3/2 | 1/2 | 2/1 | 1/0 |
| Cuba | 3/2 | 1/2 | 2/1 | 1/0 |
| Guatemala | 4/2 | 1/2 | 2/1 | 2/0 |
| Haiti | 2/1 | 1/2 | 1/0 | 0/0 |
| Mexico | 4/3 | 1/2 | 2/1 | 2/0 |

Numbers on the left of the slash show the proportion for a 20% threshold and numbers on the right of the slash show the proportion for a 10% threshold.

Cardiovascular events refer to ischaemic heart disease and stroke. Interpretation: when we used the Globorisk-LAC coefficients and the original Globorisk coefficients to classify a risk factor profile (i.e., a cell in a risk chart), in Brazil, 4% were classified as low risk with the Globorisk-LAC but as high risk with the original Globorisk. See an example of the 2x2 table below.

| **Brazil** | **Globorisk <20%** | **Globorisk ≥20%** |
| --- | --- | --- |
| **Globorisk-LAC <20%** | 54% | 4% |
| **Globorisk-LAC ≥20%** | 5% | 37% |

# **References**

1. Cohorts Consortium of Latin America and the Caribbean (CC-LAC). Cohort profile: The Cohorts Consortium of Latin America and the Caribbean (CC-LAC). *International journal of epidemiology* 2020.

# **Supplementary Figure 2: 10-year predicted cardiovascular risk per country based on the Globorisk-LAC model for a men/women who is smoker and has diabetes, a systolic blood pressure of 140 mmHg and total cholesterol of 5 mmol/L.**


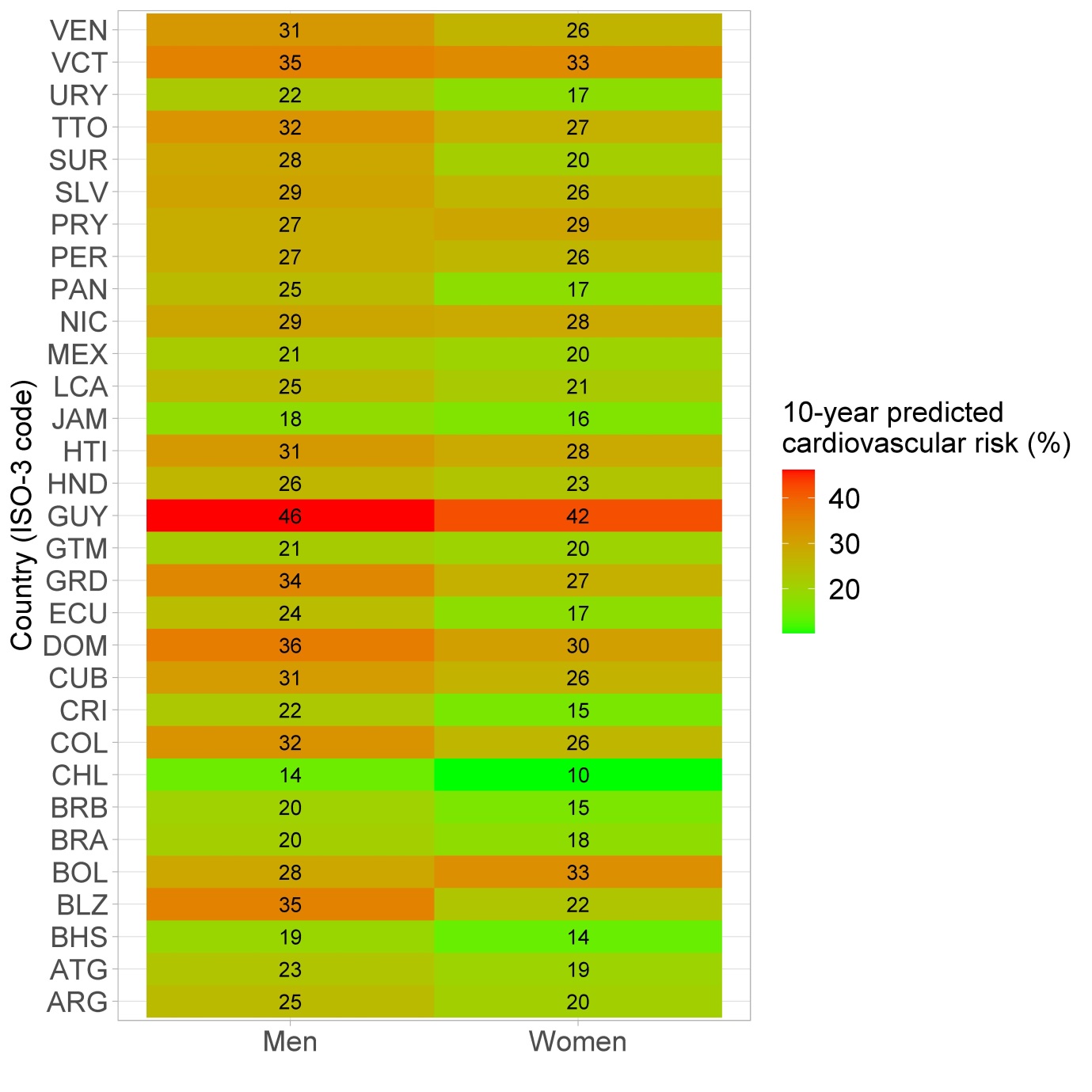


# **Supplementary Figure 3:** Calibration plots for (A) fatal/non-fatal events laboratory-based non-recalibrate original Globorisk model and (B) fatal/non-fatal events office-based non-recalibrated original Globorisk model.

**A**


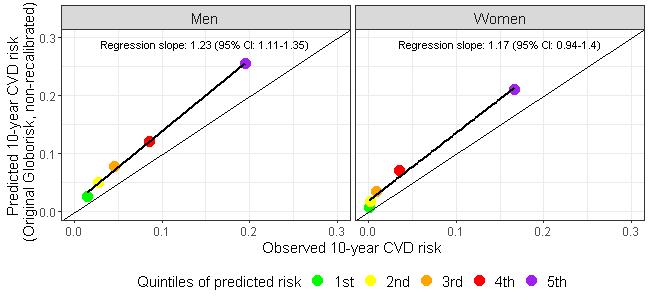


**B**


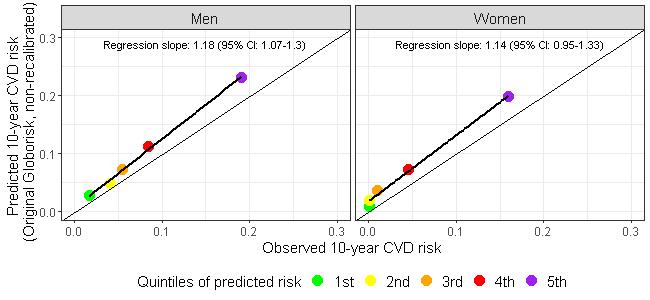


The numbers represent the beta coefficients and 95% confidence interval of a simple lineal regression in which the dependent (y) variable was the predicted risk and the independent (x) variable was the observed risk.

# **Supplementary Table 3: Net reclassification improvement index**

| **Laboratory-based models at 10% predicted cardiovascular risk threshold** | | | | |
| --- | --- | --- | --- | --- |
|  |  | Globorisk-LAC | |  |
| Outcome: absent | Original Globorisk | <10% | ≥10% | % reclassified |
|  | <10% | 13,251 | 210 | 2 |
|  | ≥10% | 563 | 3,530 | 14 |
| Outcome: present | <10% | 410 | 21 | 5 |
|  | ≥10% | 46 | 479 | 9 |
| Combined data | <10% | 13,661 | 231 | 2 |
|  | ≥10% | 609 | 4,009 | 13 |
| NRI (categorical): -0.60% (95% Confidence Interval: -0.23%-1.09%); p=0.486 | | | | |
| **Laboratory-based models at 20% predicted cardiovascular risk threshold** | | | | |
|  |  | Globorisk-LAC | |  |
| Outcome: absent | Original Globorisk | <20% | ≥20% | % reclassified |
|  | <20% | 16,437 | 219 | 1 |
|  | ≥20% | 104 | 794 | 12 |
| Outcome: present | <20% | 742 | 37 | 5 |
|  | ≥20% | 5 | 172 | 3 |
| Combined data | <20% | 17,179 | 256 | 1 |
|  | ≥20% | 109 | 966 | 10 |
| NRI (categorical) : 2.69% (95% Confidence Interval: 1.37%-4.02%); p<0.001 | | | | |
| **Office-based models at 10% predicted cardiovascular risk threshold** | | | | |
|  |  | Globorisk-LAC | |  |
| Outcome: absent | Original Globorisk | <10% | ≥10% | % reclassified |
|  | <10% | 13,358 | 237 | 1 |
|  | ≥10% | 420 | 3,539 | 11 |
| Outcome: present | <10% | 457 | 29 | 6 |
|  | ≥10% | 25 | 445 | 5 |
| Combined data | <10% | 13,815 | 266 | 2 |
|  | ≥10% | 445 | 3,984 | 10 |
| NRI (categorical): 1.46% (95% Confidence Interval: 0.00%-3.00%); p=0.062 | | | | |
| **Office-based models at 20% predicted cardiovascular risk threshold** | | | | |
|  |  | Globorisk-LAC | |  |
| Outcome: absent | Original Globorisk | <20% | ≥20% | % reclassified |
|  | <20% | 16,636 | 225 | 1 |
|  | ≥20% | 20 | 637 | 3 |
| Outcome: present | <20% | 768 | 41 | 5 |
|  | ≥20% | 3 | 144 | 2 |
| Combined data | <20% | 17,404 | 266 | 2 |
|  | ≥20% | 23 | 817 | 3 |
| NRI (categorical) : 2.81% (95% Confidence Interval: 1.46%-4.15%); p<0.001 | | | | |

Tables and Net Reclassification Improvement (NRI) estimates were computed with the reclassification function of the PredictABEL package in R. Interpretation: a categorical NRI of X% suggests that, in comparison to observations without a cardiovascular outcome, observations with a cardiovascular outcome were X% more likely to move from low to high predicted risk using the Globorisk-LAC versus the original Globorisk model.

1. Hajifathalian K, Ueda P, Lu Y, et al. A novel risk score to predict cardiovascular disease risk in national populations (Globorisk): a pooled analysis of prospective cohorts and health examination surveys. Lancet Diabetes & endocrinology 2015; 3(5): 339-55. [↑](#footnote-ref-1)
2. Ueda P, Woodward M, Lu Y, et al. Laboratory-based and office-based risk scores and charts to predict 10-year risk of cardiovascular disease in 182 countries: a pooled analysis of prospective cohorts and health surveys. Lancet Diabetes & endocrinology 2017; 5(3): 196-213. [↑](#footnote-ref-2)
