# Supplementary material for: Derivation, internal validation, and recalibration of a cardiovascular risk score for Latin America and the Caribbean (Globorisk-LAC): A pooled analysis of cohort studies
Source: Lancet Reg Health Am. 2022 Apr 23;9:100258. doi: 10.1016/j.lana.2022.100258 (PMC9107390; doi:10.1016/j.lana.2022.100258)
Supplement: Supplementary file 2 [file mmc2.pdf]

Antigua and Barbuda

Systolic Blood Pressur (mmHg) by Total cholteserol (mmol/L), for Women and Men, Non−diabetic and Diabetic, Non−smoker and Smoker, by Age.

Age 70−74

Sex	Diabetes	Smoking	SBP	3	4	5	6	7
Women	Non−diabetic	Non−smoker	180	16	17	20	22	24
Women	Non−diabetic	Non−smoker	160	11	13	14	16	18
Women	Non−diabetic	Non−smoker	140	8	9	11	12	13
Women	Non−diabetic	Non−smoker	120	6	7	8	9	10
Women	Non−diabetic	Smoker	180	24	27	30	33	36
Women	Non−diabetic	Smoker	160	18	20	22	25	28
Women	Non−diabetic	Smoker	140	13	15	17	19	21
Women	Non−diabetic	Smoker	120	10	11	12	14	15
Women	Diabetic	Non−smoker	180	31	34	38	42	45
Women	Diabetic	Non−smoker	160	24	26	29	32	35
Women	Diabetic	Non−smoker	140	18	20	22	24	27
Women	Diabetic	Non−smoker	120	13	15	16	18	20
Women	Diabetic	Smoker	180	45	49	54	58	62
Women	Diabetic	Smoker	160	35	39	43	46	51
Women	Diabetic	Smoker	140	27	30	33	36	40
Women	Diabetic	Smoker	120	20	23	25	28	31
Men	Non−diabetic	Non−smoker	180	25	27	30	33	37
Men	Non−diabetic	Non−smoker	160	18	20	23	25	28
Men	Non−diabetic	Non−smoker	140	14	15	17	19	21
Men	Non−diabetic	Non−smoker	120	10	11	13	14	16
Men	Non−diabetic	Smoker	180	32	36	39	43	47
Men	Non−diabetic	Smoker	160	25	27	30	34	37
Men	Non−diabetic	Smoker	140	18	21	23	26	28
Men	Non−diabetic	Smoker	120	14	15	17	19	21
Men	Diabetic	Non−smoker	180	43	46	51	55	59
Men	Diabetic	Non−smoker	160	33	36	40	44	48
Men	Diabetic	Non−smoker	140	25	28	31	34	38
Men	Diabetic	Non−smoker	120	19	21	23	26	29
Men	Diabetic	Smoker	180	54	58	62	67	71
Men	Diabetic	Smoker	160	43	47	51	55	59
Men	Diabetic	Smoker	140	33	37	40	44	48
Men	Diabetic	Smoker	120	25	28	31	34	38

Age 65−69

Sex	Diabetes	Smoking	SBP	3	4	5	6	7
Women	Non−diabetic	Non−smoker	180	12	13	15	17	19
Women	Non−diabetic	Non−smoker	160	8	9	10	12	13
Women	Non−diabetic	Non−smoker	140	6	6	7	8	9
Women	Non−diabetic	Non−smoker	120	4	4	5	6	6
Women	Non−diabetic	Smoker	180	18	21	23	25	28
Women	Non−diabetic	Smoker	160	13	15	17	18	21
Women	Non−diabetic	Smoker	140	9	10	12	13	15
Women	Non−diabetic	Smoker	120	6	7	8	9	10
Women	Diabetic	Non−smoker	180	24	27	30	33	36
Women	Diabetic	Non−smoker	160	17	19	22	24	27
Women	Diabetic	Non−smoker	140	12	14	16	17	20
Women	Diabetic	Non−smoker	120	9	10	11	12	14
Women	Diabetic	Smoker	180	36	40	43	47	52
Women	Diabetic	Smoker	160	27	30	33	36	40
Women	Diabetic	Smoker	140	19	22	24	27	30
Women	Diabetic	Smoker	120	14	16	17	19	22
Men	Non−diabetic	Non−smoker	180	19	21	24	27	29
Men	Non−diabetic	Non−smoker	160	14	15	17	19	22
Men	Non−diabetic	Non−smoker	140	10	11	12	14	15
Men	Non−diabetic	Non−smoker	120	7	8	9	10	11
Men	Non−diabetic	Smoker	180	26	29	32	35	38
Men	Non−diabetic	Smoker	160	19	21	23	26	29
Men	Non−diabetic	Smoker	140	13	15	17	19	21
Men	Non−diabetic	Smoker	120	9	11	12	13	15
Men	Diabetic	Non−smoker	180	34	38	42	45	50
Men	Diabetic	Non−smoker	160	25	28	31	34	38
Men	Diabetic	Non−smoker	140	18	21	23	26	28
Men	Diabetic	Non−smoker	120	13	15	17	19	21
Men	Diabetic	Smoker	180	44	48	53	57	61
Men	Diabetic	Smoker	160	34	37	41	44	49
Men	Diabetic	Smoker	140	25	28	30	34	37
Men	Diabetic	Smoker	120	18	20	22	25	28

Age 60−64

Sex	Diabetes	Smoking	SBP	3	4	5	6	7
Women	Non−diabetic	Non−smoker	180	10	12	13	15	17
Women	Non−diabetic	Non−smoker	160	7	8	9	10	11
Women	Non−diabetic	Non−smoker	140	5	5	6	7	8
Women	Non−diabetic	Non−smoker	120	3	3	4	5	5
Women	Non−diabetic	Smoker	180	17	19	21	23	26
Women	Non−diabetic	Smoker	160	11	13	14	16	18
Women	Non−diabetic	Smoker	140	8	9	10	11	12
Women	Non−diabetic	Smoker	120	5	6	7	7	8
Women	Diabetic	Non−smoker	180	22	24	27	30	33
Women	Diabetic	Non−smoker	160	15	17	19	21	24
Women	Diabetic	Non−smoker	140	10	12	13	15	16
Women	Diabetic	Non−smoker	120	7	8	9	10	11
Women	Diabetic	Smoker	180	33	36	40	44	48
Women	Diabetic	Smoker	160	23	26	29	32	35
Women	Diabetic	Smoker	140	16	18	20	23	25
Women	Diabetic	Smoker	120	11	13	14	16	18
Men	Non−diabetic	Non−smoker	180	16	18	20	23	25
Men	Non−diabetic	Non−smoker	160	11	13	14	16	18
Men	Non−diabetic	Non−smoker	140	8	9	10	11	12
Men	Non−diabetic	Non−smoker	120	5	6	7	7	8
Men	Non−diabetic	Smoker	180	22	25	27	30	33
Men	Non−diabetic	Smoker	160	15	17	19	22	24
Men	Non−diabetic	Smoker	140	11	12	13	15	17
Men	Non−diabetic	Smoker	120	7	8	9	10	12
Men	Diabetic	Non−smoker	180	30	33	36	40	44
Men	Diabetic	Non−smoker	160	21	24	26	29	32
Men	Diabetic	Non−smoker	140	15	17	18	21	23
Men	Diabetic	Non−smoker	120	10	11	13	14	16
Men	Diabetic	Smoker	180	39	43	47	51	55
Men	Diabetic	Smoker	160	28	31	35	38	42
Men	Diabetic	Smoker	140	20	22	25	28	31
Men	Diabetic	Smoker	120	14	16	17	19	22

Age 55−59

Sex	Diabetes	Smoking	SBP	3	4	5	6	7
Women	Non−diabetic	Non−smoker	180	11	12	14	15	17
Women	Non−diabetic	Non−smoker	160	7	8	9	10	12
Women	Non−diabetic	Non−smoker	140	5	5	6	7	8
Women	Non−diabetic	Non−smoker	120	3	3	4	4	5
Women	Non−diabetic	Smoker	180	17	19	21	24	26
Women	Non−diabetic	Smoker	160	11	13	14	16	18
Women	Non−diabetic	Smoker	140	7	8	10	11	12
Women	Non−diabetic	Smoker	120	5	5	6	7	8
Women	Diabetic	Non−smoker	180	23	25	28	31	34
Women	Diabetic	Non−smoker	160	15	17	19	21	24
Women	Diabetic	Non−smoker	140	10	11	13	14	16
Women	Diabetic	Non−smoker	120	7	7	8	9	11
Women	Diabetic	Smoker	180	34	37	41	45	49
Women	Diabetic	Smoker	160	24	26	29	32	35
Women	Diabetic	Smoker	140	16	18	20	22	25
Women	Diabetic	Smoker	120	11	12	13	15	17
Men	Non−diabetic	Non−smoker	180	15	17	19	22	24
Men	Non−diabetic	Non−smoker	160	10	11	13	15	16
Men	Non−diabetic	Non−smoker	140	7	8	8	10	11
Men	Non−diabetic	Non−smoker	120	4	5	5	6	7
Men	Non−diabetic	Smoker	180	21	23	26	29	32
Men	Non−diabetic	Smoker	160	14	16	18	20	22
Men	Non−diabetic	Smoker	140	9	10	12	13	15
Men	Non−diabetic	Smoker	120	6	7	8	9	10
Men	Diabetic	Non−smoker	180	28	31	34	38	42
Men	Diabetic	Non−smoker	160	19	22	24	27	30
Men	Diabetic	Non−smoker	140	13	15	16	18	20
Men	Diabetic	Non−smoker	120	9	10	11	12	14
Men	Diabetic	Smoker	180	37	41	44	49	53
Men	Diabetic	Smoker	160	26	29	32	35	39
Men	Diabetic	Smoker	140	18	20	22	25	27
Men	Diabetic	Smoker	120	12	13	15	17	19

Age 50−54

Sex	Diabetes	Smoking	SBP	3	4	5	6	7
Women	Non−diabetic	Non−smoker	180	11	13	14	16	18
Women	Non−diabetic	Non−smoker	160	7	8	9	10	12
Women	Non−diabetic	Non−smoker	140	4	5	6	7	7
Women	Non−diabetic	Non−smoker	120	3	3	3	4	5
Women	Non−diabetic	Smoker	180	18	20	22	25	28
Women	Non−diabetic	Smoker	160	12	13	15	16	18
Women	Non−diabetic	Smoker	140	7	8	9	11	12
Women	Non−diabetic	Smoker	120	5	5	6	7	7
Women	Diabetic	Non−smoker	180	23	26	29	32	35
Women	Diabetic	Non−smoker	160	15	17	19	22	24
Women	Diabetic	Non−smoker	140	10	11	13	14	16
Women	Diabetic	Non−smoker	120	6	7	8	9	10
Women	Diabetic	Smoker	180	35	39	42	46	50
Women	Diabetic	Smoker	160	24	26	29	32	36
Women	Diabetic	Smoker	140	16	18	20	22	24
Women	Diabetic	Smoker	120	10	11	13	14	16
Men	Non−diabetic	Non−smoker	180	14	16	17	20	22
Men	Non−diabetic	Non−smoker	160	9	10	11	13	14
Men	Non−diabetic	Non−smoker	140	6	6	7	8	9
Men	Non−diabetic	Non−smoker	120	3	4	4	5	6
Men	Non−diabetic	Smoker	180	19	21	24	26	29
Men	Non−diabetic	Smoker	160	12	14	15	17	19
Men	Non−diabetic	Smoker	140	8	9	10	11	13
Men	Non−diabetic	Smoker	120	5	5	6	7	8
Men	Diabetic	Non−smoker	180	26	29	32	35	38
Men	Diabetic	Non−smoker	160	17	19	21	24	26
Men	Diabetic	Non−smoker	140	11	12	14	16	17
Men	Diabetic	Non−smoker	120	7	8	9	10	11
Men	Diabetic	Smoker	180	34	37	41	45	49
Men	Diabetic	Smoker	160	23	26	28	31	35
Men	Diabetic	Smoker	140	15	17	19	21	23
Men	Diabetic	Smoker	120	10	11	12	14	15

Age 45−49

Sex	Diabetes	Smoking	SBP	3	4	5	6	7
Women	Non−diabetic	Non−smoker	180	12	13	15	17	19
Women	Non−diabetic	Non−smoker	160	7	8	9	10	12
Women	Non−diabetic	Non−smoker	140	4	5	6	6	7
Women	Non−diabetic	Non−smoker	120	2	3	3	4	4
Women	Non−diabetic	Smoker	180	18	21	23	25	28
Women	Non−diabetic	Smoker	160	12	13	15	16	18
Women	Non−diabetic	Smoker	140	7	8	9	10	11
Women	Non−diabetic	Smoker	120	4	5	5	6	7
Women	Diabetic	Non−smoker	180	24	27	30	33	36
Women	Diabetic	Non−smoker	160	15	17	19	21	24
Women	Diabetic	Non−smoker	140	10	11	12	14	15
Women	Diabetic	Non−smoker	120	6	7	7	8	9
Women	Diabetic	Smoker	180	36	39	43	47	51
Women	Diabetic	Smoker	160	24	26	29	32	36
Women	Diabetic	Smoker	140	15	17	19	21	24
Women	Diabetic	Smoker	120	9	11	12	13	15
Men	Non−diabetic	Non−smoker	180	11	13	14	16	18
Men	Non−diabetic	Non−smoker	160	7	8	9	10	11
Men	Non−diabetic	Non−smoker	140	4	5	5	6	7
Men	Non−diabetic	Non−smoker	120	2	3	3	4	4
Men	Non−diabetic	Smoker	180	16	17	20	22	24
Men	Non−diabetic	Smoker	160	10	11	12	14	16
Men	Non−diabetic	Smoker	140	6	7	8	9	10
Men	Non−diabetic	Smoker	120	3	4	5	5	6
Men	Diabetic	Non−smoker	180	21	24	27	29	33
Men	Diabetic	Non−smoker	160	14	15	17	19	21
Men	Diabetic	Non−smoker	140	8	9	11	12	13
Men	Diabetic	Non−smoker	120	5	6	7	7	8
Men	Diabetic	Smoker	180	29	32	35	38	42
Men	Diabetic	Smoker	160	18	21	23	26	28
Men	Diabetic	Smoker	140	12	13	15	16	18
Men	Diabetic	Smoker	120	7	8	9	10	12

Age 40−44

Sex	Diabetes	Smoking	SBP	3	4	5	6	7
Women	Non−diabetic	Non−smoker	180	13	14	16	18	20
Women	Non−diabetic	Non−smoker	160	8	8	10	11	12
Women	Non−diabetic	Non−smoker	140	4	5	6	6	7
Women	Non−diabetic	Non−smoker	120	2	3	3	4	4
Women	Non−diabetic	Smoker	180	20	22	24	27	30
Women	Non−diabetic	Smoker	160	12	14	15	17	19
Women	Non−diabetic	Smoker	140	7	8	9	10	12
Women	Non−diabetic	Smoker	120	4	5	5	6	7
Women	Diabetic	Non−smoker	180	26	28	32	35	38
Women	Diabetic	Non−smoker	160	16	18	20	22	25
Women	Diabetic	Non−smoker	140	10	11	12	14	15
Women	Diabetic	Non−smoker	120	6	6	7	8	9
Women	Diabetic	Smoker	180	38	42	46	50	54
Women	Diabetic	Smoker	160	25	27	30	34	37
Women	Diabetic	Smoker	140	15	17	19	21	24
Women	Diabetic	Smoker	120	9	10	12	13	15
Men	Non−diabetic	Non−smoker	180	7	8	9	11	12
Men	Non−diabetic	Non−smoker	160	4	5	5	6	7
Men	Non−diabetic	Non−smoker	140	2	3	3	4	4
Men	Non−diabetic	Non−smoker	120	1	1	2	2	2
Men	Non−diabetic	Smoker	180	10	11	13	14	16
Men	Non−diabetic	Smoker	160	6	7	8	9	10
Men	Non−diabetic	Smoker	140	3	4	4	5	6
Men	Non−diabetic	Smoker	120	2	2	2	3	3
Men	Diabetic	Non−smoker	180	14	16	18	20	22
Men	Diabetic	Non−smoker	160	9	10	11	12	14
Men	Diabetic	Non−smoker	140	5	6	6	7	8
Men	Diabetic	Non−smoker	120	3	3	4	4	5
Men	Diabetic	Smoker	180	19	22	24	27	30
Men	Diabetic	Smoker	160	12	13	15	17	19
Men	Diabetic	Smoker	140	7	8	9	10	11
Men	Diabetic	Smoker	120	4	5	5	6	7

Argentina

Systolic Blood Pressur (mmHg) by Age; Total cholteserol (mmol/L)

Women

Non−diabetic, Non−smoker

Age	SBP	3	4	5	6	7
70−74	180	22	25	27	30	34
	160	17	19	21	23	26
	140	12	14	15	17	19
	120	9	10	11	13	14
65−69	180	16	18	20	23	25
	160	12	13	15	16	18
	140	8	9	10	12	13
	120	6	6	7	8	9
60−64	180	12	14	16	17	19
	160	8	9	11	12	13
	140	6	6	7	8	9
	120	4	4	5	5	6
55−59	180	10	11	12	14	16
	160	6	7	8	9	10
	140	4	5	5	6	7
	120	3	3	3	4	4
50−54	180	8	9	10	12	13
	160	5	6	7	7	8
	140	3	4	4	5	5
	120	2	2	2	3	3
45−49	180	7	8	9	10	11
	160	4	5	5	6	7
	140	2	3	3	4	4
	120	1	1	2	2	2
40−44	180	6	7	8	9	10
	160	3	4	4	5	6
	140	2	2	2	3	3
	120	1	1	1	1	2

Non−diabetic, Smoker

Age	SBP	3	4	5	6	7
70−74	180	33	37	40	44	48
	160	25	28	31	34	38
	140	19	21	24	26	29
	120	14	16	18	20	22
65−69	180	25	28	31	34	38
	160	18	20	23	25	28
	140	13	15	16	18	21
	120	9	10	12	13	15
60−64	180	19	21	24	27	29
	160	13	15	17	19	21
	140	9	10	12	13	15
	120	6	7	8	9	10
55−59	180	16	17	20	22	24
	160	10	12	13	15	16
	140	7	8	9	10	11
	120	4	5	6	6	7
50−54	180	13	15	16	18	21
	160	8	9	11	12	13
	140	5	6	7	8	9
	120	3	4	4	5	5
45−49	180	11	12	14	16	17
	160	7	8	9	10	11
	140	4	5	5	6	7
	120	2	3	3	3	4
40−44	180	10	11	12	14	15
	160	6	6	7	8	9
	140	3	4	4	5	5
	120	2	2	2	3	3

Diabetic, Non−smoker

Age	SBP	3	4	5	6	7
70−74	180	42	46	50	55	59
	160	33	36	40	44	48
	140	25	28	31	34	37
	120	19	21	23	26	29
65−69	180	32	36	39	43	47
	160	24	27	29	33	36
	140	17	19	22	24	27
	120	12	14	16	17	20
60−64	180	25	28	31	34	38
	160	18	20	22	25	27
	140	12	14	15	17	19
	120	8	9	11	12	13
55−59	180	21	23	25	28	31
	160	14	16	17	19	22
	140	9	10	12	13	15
	120	6	7	8	9	10
50−54	180	17	19	22	24	27
	160	11	13	14	16	18
	140	7	8	9	10	12
	120	4	5	6	6	7
45−49	180	15	16	18	20	23
	160	9	10	12	13	15
	140	5	6	7	8	9
	120	3	4	4	5	5
40−44	180	13	14	16	18	20
	160	8	9	10	11	12
	140	4	5	6	6	7
	120	2	3	3	4	4

Diabetic, Smoker

Age	SBP	3	4	5	6	7
70−74	180	59	63	67	72	76
	160	47	51	56	60	65
	140	37	41	45	49	53
	120	28	31	35	38	42
65−69	180	47	51	55	60	64
	160	36	39	43	47	51
	140	27	29	33	36	40
	120	19	22	24	27	30
60−64	180	37	41	45	49	53
	160	27	30	33	37	40
	140	19	21	24	26	29
	120	13	15	17	19	21
55−59	180	31	34	38	41	45
	160	22	24	27	30	33
	140	15	16	18	20	23
	120	10	11	12	14	15
50−54	180	27	29	33	36	40
	160	18	20	22	24	27
	140	11	13	14	16	18
	120	7	8	9	10	12
45−49	180	23	25	28	31	34
	160	14	16	18	20	22
	140	9	10	11	13	14
	120	5	6	7	8	9
40−44	180	20	22	25	28	30
	160	12	14	15	17	19
	140	7	8	9	10	12
	120	4	5	5	6	7

Men

Non−diabetic, Non−smoker

Age	SBP	3	4	5	6	7
70−74	180	20	22	25	28	30
	160	15	17	19	21	23
	140	11	12	14	15	17
	120	8	9	10	11	13
65−69	180	18	20	22	25	27
	160	13	14	16	18	20
	140	9	10	11	13	14
	120	6	7	8	9	10
60−64	180	17	19	21	24	26
	160	12	13	15	17	19
	140	8	9	10	11	13
	120	5	6	7	8	9
55−59	180	16	18	20	23	25
	160	11	12	14	15	17
	140	7	8	9	10	11
	120	4	5	6	7	7
50−54	180	14	16	18	20	22
	160	9	10	12	13	15
	140	6	7	7	8	9
	120	4	4	5	5	6
45−49	180	12	13	15	17	19
	160	7	8	9	10	12
	140	4	5	6	6	7
	120	2	3	3	4	4
40−44	180	8	10	11	12	14
	160	5	6	6	7	8
	140	3	3	4	4	5
	120	1	2	2	2	3

Non−diabetic, Smoker

Age	SBP	3	4	5	6	7
70−74	180	27	30	33	36	40
	160	20	22	25	28	31
	140	15	17	19	21	23
	120	11	12	14	15	17
65−69	180	24	27	30	33	36
	160	17	19	22	24	27
	140	12	14	15	17	19
	120	9	10	11	12	14
60−64	180	23	26	29	32	35
	160	16	18	20	22	25
	140	11	12	14	16	18
	120	7	8	10	11	12
55−59	180	22	24	27	30	33
	160	15	17	19	21	23
	140	10	11	12	14	16
	120	6	7	8	9	10
50−54	180	19	22	24	27	30
	160	13	14	16	18	20
	140	8	9	10	12	13
	120	5	6	6	7	8
45−49	180	16	18	20	22	25
	160	10	11	13	14	16
	140	6	7	8	9	10
	120	4	4	5	5	6
40−44	180	12	13	15	17	19
	160	7	8	9	10	11
	140	4	5	5	6	7
	120	2	3	3	3	4

Diabetic, Non−smoker

Age	SBP	3	4	5	6	7
70−74	180	36	39	43	47	51
	160	27	30	33	37	40
	140	20	23	25	28	31
	120	15	17	19	21	23
65−69	180	32	35	39	43	47
	160	24	26	29	32	36
	140	17	19	21	24	26
	120	12	14	15	17	19
60−64	180	31	34	38	41	45
	160	22	25	27	30	33
	140	15	17	19	22	24
	120	11	12	13	15	17
55−59	180	30	33	36	40	43
	160	20	23	25	28	31
	140	14	15	17	19	21
	120	9	10	11	13	14
50−54	180	26	29	32	36	39
	160	18	20	22	24	27
	140	11	13	14	16	18
	120	7	8	9	10	12
45−49	180	22	24	27	30	33
	160	14	16	18	20	22
	140	9	10	11	12	14
	120	5	6	7	8	9
40−44	180	16	18	20	23	25
	160	10	11	12	14	16
	140	6	7	7	8	9
	120	3	4	4	5	6

Diabetic, Smoker

Age	SBP	3	4	5	6	7
70−74	180	46	50	54	58	63
	160	36	39	43	47	51
	140	27	30	33	37	40
	120	20	23	25	28	31
65−69	180	42	46	50	54	58
	160	31	35	38	42	46
	140	23	26	28	31	35
	120	17	19	21	23	26
60−64	180	40	44	48	52	57
	160	29	33	36	39	43
	140	21	23	26	29	32
	120	14	16	18	20	23
55−59	180	39	42	46	50	55
	160	27	30	33	37	40
	140	19	21	23	26	29
	120	12	14	16	18	20
50−54	180	35	38	42	46	50
	160	24	26	29	32	35
	140	16	17	19	22	24
	120	10	11	13	14	16
45−49	180	29	32	36	39	43
	160	19	21	24	26	29
	140	12	13	15	17	19
	120	7	8	9	11	12
40−44	180	22	24	27	30	33
	160	14	15	17	19	21
	140	8	9	10	12	13
	120	5	5	6	7	8

Barbados

Systolic Blood Pressur (mmHg) by Total cholteserol (mmol/L)

Women

Age	Systolic Blood Pressur (mmHg)	Non−diabetic Non−smoker					Non−diabetic Smoker					Diabetic Non−smoker					Diabetic Smoker				
	Total cholteserol (mmol/L)	3	4	5	6	7	3	4	5	6	7	3	4	5	6	7	3	4	5	6	7
70−74	180	15	17	19	21	24	23	26	29	32	35	30	33	37	40	44	44	48	52	57	61
	160	11	12	14	16	18	17	19	22	24	27	23	25	28	31	34	34	38	41	45	49
	140	8	9	10	12	13	13	14	16	18	20	17	19	21	24	26	26	29	32	35	39
	120	6	7	7	8	9	9	11	12	13	15	13	14	16	18	20	20	22	24	27	30
65−69	180	11	12	14	16	17	17	19	22	24	27	23	25	28	31	34	34	37	41	45	49
	160	8	9	10	11	12	12	14	15	17	19	16	18	20	23	25	25	28	31	34	38
	140	5	6	7	8	9	9	10	11	12	14	12	13	15	16	18	18	20	23	25	28
	120	4	4	5	5	6	6	7	8	9	10	8	9	10	12	13	13	15	16	18	20
60−64	180	9	10	12	13	15	15	16	18	20	23	19	22	24	27	30	29	32	36	39	43
	160	6	7	8	9	10	10	11	13	14	16	13	15	17	19	21	21	23	26	29	32
	140	4	5	5	6	7	7	8	9	10	11	9	10	12	13	15	14	16	18	20	23
	120	3	3	3	4	4	4	5	6	7	7	6	7	8	9	10	10	11	13	14	16
55−59	180	7	8	9	10	11	11	12	14	15	17	15	16	18	20	23	23	25	28	31	34
	160	4	5	6	6	7	7	8	9	10	12	10	11	12	14	15	15	17	19	21	24
	140	3	3	4	4	5	5	5	6	7	8	6	7	8	9	10	10	11	13	14	16
	120	2	2	2	3	3	3	3	4	4	5	4	5	5	6	7	7	7	8	10	11
50−54	180	5	5	6	7	8	8	9	10	11	12	10	12	13	15	17	16	18	21	23	25
	160	3	3	4	4	5	5	5	6	7	8	7	7	8	9	11	11	12	13	15	17
	140	2	2	2	3	3	3	3	4	4	5	4	5	5	6	7	7	8	9	10	11
	120	1	1	1	1	2	2	2	2	3	3	2	3	3	4	4	4	5	5	6	7
45−49	180	3	3	4	4	5	5	5	6	7	8	6	7	8	9	10	10	12	13	15	16
	160	1	2	2	2	3	3	3	4	4	5	4	4	5	6	6	6	7	8	9	10
	140	1	1	1	1	1	1	2	2	2	3	2	2	3	3	4	4	4	5	5	6
	120	0	0	0	1	1	1	1	1	1	1	1	1	2	2	2	2	2	3	3	4
40−44	180	2	2	2	2	3	3	3	4	4	5	4	4	5	6	7	6	7	8	9	11
	160	1	1	1	1	1	1	2	2	2	3	2	2	3	3	4	4	4	5	5	6
	140	0	0	0	1	1	1	1	1	1	1	1	1	1	2	2	2	2	3	3	4
	120	0	0	0	0	0	0	0	0	0	1	0	1	1	1	1	1	1	1	2	2

Men

Age	Systolic Blood Pressur (mmHg)	Non−diabetic Non−smoker					Non−diabetic Smoker					Diabetic Non−smoker					Diabetic Smoker				
	Total cholteserol (mmol/L)	3	4	5	6	7	3	4	5	6	7	3	4	5	6	7	3	4	5	6	7
70−74	180	22	25	28	31	34	30	33	36	40	44	39	43	47	51	56	50	54	59	63	68
	160	17	19	21	23	26	22	25	28	31	34	30	33	37	40	44	39	43	47	51	56
	140	12	14	15	17	19	17	19	21	23	26	23	25	28	31	34	30	34	37	41	44
	120	9	10	11	13	14	12	14	15	17	19	17	19	21	24	26	23	25	28	31	35
65−69	180	19	22	24	27	30	26	29	32	35	39	35	38	42	46	50	45	49	53	57	62
	160	14	16	17	19	22	19	21	23	26	29	26	28	32	35	38	34	37	41	45	49
	140	10	11	12	14	16	13	15	17	19	21	19	21	23	26	29	25	28	31	34	37
	120	7	8	9	10	11	10	11	12	14	15	13	15	17	19	21	18	20	23	25	28
60−64	180	16	18	20	22	24	21	24	26	29	32	29	32	35	39	42	38	41	45	49	53
	160	11	12	14	15	17	15	17	19	21	23	20	23	25	28	31	27	30	33	37	40
	140	7	8	9	10	12	10	11	13	14	16	14	16	18	20	22	19	21	24	26	29
	120	5	5	6	7	8	7	8	9	10	11	10	11	12	14	15	13	15	17	19	21
55−59	180	12	13	15	17	19	16	18	20	23	25	22	25	28	31	34	30	33	36	40	44
	160	8	9	10	11	13	11	12	14	15	17	15	17	19	21	24	20	23	25	28	31
	140	5	6	6	7	8	7	8	9	10	11	10	11	13	14	16	14	15	17	19	22
	120	3	4	4	5	5	5	5	6	7	8	7	7	8	9	11	9	10	12	13	15
50−54	180	10	12	13	15	17	14	16	18	20	22	20	22	24	27	30	26	29	32	36	39
	160	7	7	8	9	11	9	10	12	13	15	13	14	16	18	20	17	19	22	24	27
	140	4	5	5	6	7	6	6	7	8	9	8	9	10	12	13	11	13	14	16	18
	120	2	3	3	4	4	3	4	5	5	6	5	6	6	7	8	7	8	9	10	11
45−49	180	9	10	12	13	15	13	14	16	18	20	17	19	22	24	27	23	26	29	32	35
	160	6	6	7	8	9	8	9	10	11	13	11	12	14	15	17	15	17	19	21	23
	140	3	4	4	5	5	5	5	6	7	8	7	8	9	10	11	9	10	12	13	15
	120	2	2	2	3	3	3	3	4	4	5	4	5	5	6	7	6	6	7	8	9
40−44	180	5	6	6	7	8	7	8	9	10	11	10	11	12	14	16	13	15	17	19	21
	160	3	3	4	4	5	4	5	5	6	7	6	7	7	8	9	8	9	10	12	13
	140	1	2	2	2	3	2	3	3	3	4	3	4	4	5	6	5	5	6	7	8
	120	1	1	1	1	1	1	1	2	2	2	2	2	2	3	3	3	3	4	4	5

Belize

Systolic Blood Pressur (mmHg) by Total cholteserol (mmol/L)

Women

Non−diabetic

Age	Systolic BP	Non−smoker 3	4	5	6	7	Smoker 3	4	5	6	7
70−74	180	22	24	27	30	33	33	36	40	44	48
	160	16	18	20	23	25	25	28	31	34	38
	140	12	13	15	17	19	19	21	23	26	29
	120	9	10	11	12	14	14	16	17	19	22
65−69	180	16	18	20	23	25	25	28	31	34	38
	160	12	13	15	16	18	18	20	23	25	28
	140	8	9	10	12	13	13	14	16	18	20
	120	6	6	7	8	9	9	10	12	13	15
60−64	180	13	14	16	18	20	20	22	25	28	31
	160	9	10	11	12	14	14	16	17	20	22
	140	6	7	7	8	10	9	11	12	14	15
	120	4	4	5	6	6	6	7	8	9	10
55−59	180	11	12	14	16	18	17	19	22	24	27
	160	7	8	9	10	12	12	13	15	16	18
	140	5	5	6	7	8	8	9	10	11	12
	120	3	3	4	4	5	5	6	6	7	8
50−54	180	10	11	12	14	16	15	17	19	22	24
	160	6	7	8	9	10	10	11	13	14	16
	140	4	4	5	6	6	6	7	8	9	10
	120	2	3	3	3	4	4	4	5	6	6
45−49	180	9	10	11	12	14	14	15	17	19	22
	160	5	6	7	8	9	9	10	11	12	14
	140	3	3	4	5	5	5	6	7	7	8
	120	2	2	2	3	3	3	3	4	4	5
40−44	180	6	7	8	9	10	10	11	13	14	16
	160	4	4	5	5	6	6	7	8	9	10
	140	2	2	3	3	3	3	4	4	5	6
	120	1	1	1	2	2	2	2	2	3	3

Diabetic

Age	Systolic BP	Non−smoker 3	4	5	6	7	Smoker 3	4	5	6	7
70−74	180	42	46	50	54	59	58	63	67	71	76
	160	32	36	39	43	47	47	51	55	60	64
	140	25	27	30	33	37	37	40	44	48	52
	120	18	20	23	25	28	28	31	34	38	41
65−69	180	32	36	39	43	47	47	51	55	60	64
	160	24	26	29	32	36	35	39	43	47	51
	140	17	19	21	24	26	26	29	32	36	39
	120	12	14	15	17	19	19	21	24	26	29
60−64	180	26	29	32	35	39	39	43	47	51	55
	160	18	21	23	25	28	28	31	34	38	42
	140	13	14	16	18	20	20	22	25	27	30
	120	9	10	11	12	14	14	15	17	19	22
55−59	180	23	25	28	31	34	34	38	41	45	49
	160	15	17	19	22	24	24	26	29	32	36
	140	10	12	13	15	16	16	18	20	22	25
	120	7	8	9	10	11	11	12	14	15	17
50−54	180	20	23	25	28	31	31	34	37	41	45
	160	13	15	17	19	21	21	23	26	28	31
	140	8	9	11	12	14	13	15	17	19	21
	120	5	6	7	8	9	9	10	11	12	14
45−49	180	18	20	23	25	28	28	31	34	38	41
	160	11	13	14	16	18	18	20	22	25	28
	140	7	8	9	10	11	11	13	14	16	18
	120	4	5	5	6	7	7	8	9	10	11
40−44	180	14	15	17	19	21	21	24	26	29	32
	160	8	9	10	12	13	13	15	16	18	20
	140	5	5	6	7	8	8	9	10	11	13
	120	3	3	3	4	5	5	5	6	7	7

Men

Non−diabetic

Age	Systolic BP	Non−smoker 3	4	5	6	7	Smoker 3	4	5	6	7
70−74	180	33	37	40	44	48	43	47	51	55	60
	160	25	28	31	34	38	33	37	40	44	48
	140	19	21	23	26	29	25	28	31	34	38
	120	14	16	17	19	22	19	21	23	26	29
65−69	180	29	32	35	39	42	38	41	45	49	54
	160	21	23	26	29	32	28	31	34	38	41
	140	15	17	19	21	23	20	23	25	28	31
	120	11	12	13	15	17	15	16	18	20	23
60−64	180	26	29	32	35	39	34	38	42	46	50
	160	18	21	23	26	28	25	27	30	34	37
	140	13	14	16	18	20	17	19	22	24	27
	120	9	10	11	12	14	12	13	15	17	19
55−59	180	21	23	26	28	31	28	31	34	37	41
	160	14	16	18	20	22	19	21	24	26	29
	140	9	10	12	13	15	13	14	16	18	20
	120	6	7	8	9	10	8	9	11	12	13
50−54	180	16	18	20	22	25	22	24	27	30	33
	160	10	12	13	15	16	14	16	18	20	22
	140	6	7	8	9	11	9	10	11	13	14
	120	4	5	5	6	7	6	6	7	8	9
45−49	180	12	13	15	16	18	16	18	20	22	25
	160	7	8	9	10	12	10	11	13	14	16
	140	4	5	5	6	7	6	7	8	9	10
	120	2	3	3	4	4	4	4	5	5	6
40−44	180	7	8	9	10	11	9	10	12	13	15
	160	4	4	5	6	6	5	6	7	8	9
	140	2	2	3	3	4	3	4	4	5	5
	120	1	1	1	2	2	2	2	2	3	3

Diabetic

Age	Systolic BP	Non−smoker 3	4	5	6	7	Smoker 3	4	5	6	7
70−74	180	55	59	64	68	72	67	71	75	79	83
	160	44	48	52	56	60	55	59	64	68	72
	140	34	37	41	45	49	44	48	52	56	61
	120	26	28	32	35	38	34	37	41	45	49
65−69	180	49	53	57	62	66	60	65	69	73	78
	160	37	41	45	49	53	48	52	56	60	65
	140	28	31	34	37	41	36	40	44	48	52
	120	20	22	25	28	31	27	30	33	36	40
60−64	180	45	49	53	58	62	56	61	65	70	74
	160	33	37	40	44	48	43	47	51	55	60
	140	24	26	29	32	36	31	35	38	42	46
	120	17	19	21	23	26	22	25	28	31	34
55−59	180	37	40	44	48	52	47	51	55	60	64
	160	26	29	32	35	38	34	37	41	45	49
	140	18	20	22	24	27	24	26	29	32	36
	120	12	13	15	17	19	16	18	20	22	25
50−54	180	29	32	36	39	43	38	42	46	50	54
	160	20	22	24	27	30	26	29	32	35	39
	140	13	14	16	18	20	17	19	22	24	27
	120	8	9	10	12	13	11	13	14	16	18
45−49	180	22	24	27	30	33	29	32	35	39	43
	160	14	16	17	19	22	19	21	23	26	29
	140	9	10	11	12	14	12	13	15	17	19
	120	5	6	7	8	8	7	8	9	10	12
40−44	180	13	15	16	18	20	18	20	22	25	27
	160	8	9	10	11	12	11	12	14	15	17
	140	4	5	6	7	7	6	7	8	9	10
	120	2	3	3	4	4	4	4	5	5	6

Total cholteserol (mmol/L)

Bolivia

Systolic Blood Pressur (mmHg) by Total cholteserol (mmol/L)

Women

Age	Systolic Blood Pressur (mmHg)	Non−diabetic Non−smoker					Non−diabetic Smoker					Diabetic Non−smoker					Diabetic Smoker				
		3	4	5	6	7	3	4	5	6	7	3	4	5	6	7	3	4	5	6	7
70−74	180	22	25	28	31	34	34	37	41	44	48	42	46	50	55	59	59	63	68	72	76
	160	17	19	21	23	26	25	28	31	35	38	33	36	40	44	48	47	52	56	60	65
	140	12	14	15	17	19	19	21	24	26	29	25	28	31	34	37	37	41	45	49	53
	120	9	10	11	13	14	14	16	18	20	22	19	21	23	26	29	28	31	35	38	42
65−69	180	19	21	23	26	29	28	31	35	38	42	36	40	44	48	52	52	56	60	65	69
	160	13	15	17	19	21	21	23	26	28	31	27	30	33	36	40	40	44	48	52	56
	140	9	11	12	13	15	15	17	19	21	23	20	22	24	27	30	30	33	36	40	44
	120	6	7	8	9	11	10	12	13	15	17	14	16	18	20	22	22	24	27	30	33
60−64	180	18	20	23	25	28	28	31	34	37	41	36	39	43	47	51	51	55	59	64	68
	160	13	14	16	18	20	20	22	24	27	30	26	28	31	35	38	38	42	45	50	54
	140	8	10	11	12	14	14	15	17	19	21	18	20	22	25	28	27	30	33	37	41
	120	6	6	7	8	9	9	10	12	13	15	12	14	16	17	19	19	22	24	27	29
55−59	180	19	21	23	26	28	28	31	35	38	42	36	40	44	48	52	52	56	60	65	69
	160	12	14	16	17	20	19	22	24	27	30	25	28	31	34	38	38	41	45	49	54
	140	8	9	10	12	13	13	15	16	18	20	17	19	22	24	27	26	29	32	36	39
	120	5	6	7	8	9	9	10	11	12	14	12	13	15	16	18	18	20	22	25	28
50−54	180	19	21	23	26	29	29	32	35	38	42	37	40	44	48	52	52	56	61	65	69
	160	12	14	15	17	19	19	21	24	26	29	25	28	31	34	37	37	41	45	49	53
	140	8	9	10	11	12	12	14	16	17	19	16	18	20	23	25	25	28	31	34	38
	120	5	5	6	7	8	8	9	10	11	13	11	12	13	15	17	17	19	21	23	26
45−49	180	18	20	22	24	27	27	30	33	36	40	35	38	42	46	50	49	54	58	62	67
	160	11	12	14	16	17	17	19	22	24	27	23	25	28	31	34	34	38	41	45	49
	140	7	8	9	10	11	11	12	14	15	17	14	16	18	20	23	22	25	28	31	34
	120	4	5	5	6	7	7	7	8	10	11	9	10	11	13	14	14	16	18	20	22
40−44	180	14	16	18	20	22	22	25	27	30	34	29	32	35	39	42	42	46	50	55	59
	160	9	10	11	12	14	14	15	17	19	21	18	20	23	25	28	28	31	34	37	41
	140	5	6	6	7	8	8	9	10	12	13	11	12	14	16	17	17	19	22	24	27
	120	3	3	4	4	5	5	5	6	7	8	7	7	8	9	11	11	12	13	15	17

Men

Age	Systolic Blood Pressur (mmHg)	Non−diabetic Non−smoker					Non−diabetic Smoker					Diabetic Non−smoker					Diabetic Smoker				
		3	4	5	6	7	3	4	5	6	7	3	4	5	6	7	3	4	5	6	7
70−74	180	23	26	29	32	35	31	34	37	41	45	41	44	48	53	57	51	56	60	64	69
	160	17	19	22	24	27	23	26	29	32	35	31	34	38	42	46	41	44	48	53	57
	140	13	14	16	18	20	17	19	22	24	27	24	26	29	32	36	31	35	38	42	46
	120	9	10	12	13	15	13	14	16	18	20	18	20	22	24	27	24	26	29	32	36
65−69	180	20	22	25	28	31	27	30	33	36	40	36	39	43	47	51	46	50	54	59	63
	160	14	16	18	20	23	20	22	24	27	30	27	29	33	36	39	35	38	42	46	50
	140	10	11	13	14	16	14	16	18	20	22	19	22	24	27	30	26	29	32	35	39
	120	7	8	9	10	11	10	11	13	14	16	14	15	17	19	22	19	21	23	26	29
60−64	180	20	22	24	27	30	26	29	32	35	39	35	38	42	46	50	45	49	53	58	62
	160	14	15	17	19	21	18	21	23	25	28	25	28	31	34	37	33	36	40	44	48
	140	9	10	12	13	15	13	14	16	18	20	18	20	22	24	27	24	26	29	32	35
	120	6	7	8	9	10	9	10	11	12	14	12	14	15	17	19	16	18	21	23	26
55−59	180	20	22	24	27	30	26	29	32	36	39	35	39	42	46	51	45	49	54	58	62
	160	13	15	17	19	21	18	20	22	25	28	25	27	30	33	37	32	36	39	43	47
	140	9	10	11	12	14	12	13	15	17	19	17	19	21	23	26	22	25	28	31	34
	120	6	6	7	8	9	8	9	10	11	13	11	12	14	16	18	15	17	19	21	24
50−54	180	19	21	24	27	29	26	29	32	35	38	34	38	42	46	50	44	48	53	57	61
	160	13	14	16	18	20	17	19	21	24	26	23	26	29	32	35	31	34	38	41	45
	140	8	9	10	11	13	11	12	14	16	17	15	17	19	21	24	21	23	26	28	32
	120	5	6	6	7	8	7	8	9	10	11	10	11	12	14	16	14	15	17	19	21
45−49	180	17	19	21	24	26	23	25	28	31	34	31	34	37	41	45	40	44	48	52	56
	160	11	12	13	15	17	15	16	18	20	23	20	22	25	28	31	27	30	33	36	40
	140	6	7	8	9	11	9	10	11	13	14	13	14	16	18	20	17	19	21	24	27
	120	4	4	5	6	6	5	6	7	8	9	8	9	10	11	13	11	12	14	15	17
40−44	180	14	16	18	20	22	19	22	24	27	30	26	29	32	35	39	34	38	42	46	50
	160	8	10	11	12	14	12	13	15	17	19	16	18	20	23	25	22	25	27	30	33
	140	5	6	6	7	8	7	8	9	10	11	10	11	12	14	16	14	15	17	19	21
	120	3	3	4	4	5	4	5	5	6	7	6	7	7	8	9	8	9	10	12	13

Total cholteserol (mmol/L)

Brazil

Women

Age	Systolic Blood Pressur (mmHg)	Non−diabetic Non−smoker					Non−diabetic Smoker					Diabetic Non−smoker					Diabetic Smoker				
	Total cholteserol (mmol/L)	3	4	5	6	7	3	4	5	6	7	3	4	5	6	7	3	4	5	6	7
70−74	180	14	15	17	19	21	21	24	26	29	32	28	31	34	37	41	41	44	49	53	57
	160	10	11	13	14	16	16	18	20	22	24	21	23	26	28	32	31	35	38	42	46
	140	7	8	9	10	12	12	13	15	16	18	15	17	19	21	24	24	26	29	32	36
	120	5	6	7	7	8	8	9	11	12	13	11	13	14	16	18	18	20	22	25	27
65−69	180	11	13	14	16	18	18	20	22	25	28	24	26	29	32	35	35	39	42	46	51
	160	8	9	10	11	13	13	14	16	18	20	17	19	21	24	26	26	29	32	35	39
	140	6	6	7	8	9	9	10	11	13	14	12	14	15	17	19	19	21	24	26	29
	120	4	4	5	6	6	6	7	8	9	10	8	10	11	12	14	14	15	17	19	21
60−64	180	10	11	13	14	16	16	18	20	22	24	21	23	26	28	31	31	35	38	42	46
	160	7	8	9	10	11	11	12	14	15	17	14	16	18	20	22	22	25	28	30	34
	140	4	5	6	6	7	7	8	9	10	12	10	11	12	14	16	16	17	19	22	24
	120	3	3	4	4	5	5	5	6	7	8	7	7	8	10	11	11	12	13	15	17
55−59	180	9	10	11	13	14	14	16	18	20	22	19	21	23	26	28	28	31	35	38	42
	160	6	6	7	8	9	9	10	12	13	15	12	14	16	18	20	19	22	24	27	30
	140	4	4	5	5	6	6	7	8	9	10	8	9	10	12	13	13	15	16	18	20
	120	2	3	3	3	4	4	4	5	6	6	5	6	7	8	9	9	10	11	12	14
50−54	180	8	9	11	12	13	13	15	17	19	21	17	20	22	24	27	27	30	33	36	40
	160	5	6	7	7	8	8	9	11	12	13	11	13	14	16	18	18	20	22	25	27
	140	3	4	4	5	5	5	6	7	8	9	7	8	9	10	12	11	13	14	16	18
	120	2	2	2	3	3	3	4	4	5	5	4	5	6	6	7	7	8	9	10	12
45−49	180	7	8	9	10	11	11	13	14	16	18	15	17	19	21	23	23	26	29	32	35
	160	4	5	5	6	7	7	8	9	10	11	9	10	12	13	15	15	17	18	21	23
	140	2	3	3	4	4	4	5	5	6	7	6	6	7	8	9	9	10	12	13	15
	120	1	1	2	2	2	2	3	3	4	4	3	4	4	5	6	6	6	7	8	9
40−44	180	6	7	8	9	10	10	11	12	14	15	13	14	16	18	20	20	22	25	28	31
	160	3	4	4	5	6	6	6	7	8	9	8	9	10	11	12	12	14	15	17	19
	140	2	2	2	3	3	3	4	4	5	5	4	5	6	6	7	7	8	9	10	12
	120	1	1	1	1	2	2	2	2	3	3	2	3	3	4	4	4	5	5	6	7

Men

Age	Systolic Blood Pressur (mmHg)	Non−diabetic Non−smoker					Non−diabetic Smoker					Diabetic Non−smoker					Diabetic Smoker				
	Total cholteserol (mmol/L)	3	4	5	6	7	3	4	5	6	7	3	4	5	6	7	3	4	5	6	7
70−74	180	18	20	22	24	27	24	26	29	32	36	32	35	39	42	46	41	45	49	53	58
	160	13	15	16	18	20	18	20	22	24	27	24	27	30	33	36	32	35	39	42	46
	140	9	11	12	13	15	13	15	16	18	20	18	20	22	25	28	24	27	30	33	36
	120	7	8	9	10	11	9	11	12	13	15	13	15	17	19	21	18	20	22	25	28
65−69	180	16	18	20	22	24	21	24	26	29	32	29	32	35	39	42	38	41	45	49	54
	160	11	13	14	16	18	15	17	19	21	24	21	23	26	29	32	28	31	34	38	41
	140	8	9	10	11	13	11	12	14	15	17	15	17	19	21	23	20	23	25	28	31
	120	5	6	7	8	9	8	9	10	11	12	11	12	13	15	17	15	16	18	20	23
60−64	180	14	16	18	20	23	20	22	24	27	30	27	30	33	36	40	35	39	42	46	50
	160	10	11	13	14	16	14	15	17	19	21	19	21	23	26	29	25	28	31	34	38
	140	7	8	8	10	11	9	10	12	13	15	13	15	16	18	20	18	20	22	24	27
	120	4	5	6	6	7	6	7	8	9	10	9	10	11	13	14	12	14	15	17	19
55−59	180	14	15	17	19	22	19	21	23	26	29	25	28	31	34	38	34	37	41	44	48
	160	9	10	11	13	14	13	14	16	18	20	17	19	22	24	27	23	26	29	32	35
	140	6	7	8	8	10	8	9	10	12	13	12	13	15	16	18	16	18	20	22	24
	120	4	4	5	5	6	5	6	7	8	9	8	9	10	11	12	10	12	13	15	17
50−54	180	13	15	16	18	21	18	20	22	25	27	24	27	30	33	37	32	36	39	43	47
	160	8	9	11	12	13	12	13	15	16	18	16	18	20	22	25	22	24	27	30	33
	140	5	6	7	8	9	7	8	9	10	12	10	12	13	15	16	14	16	18	20	22
	120	3	4	4	5	5	5	5	6	7	7	6	7	8	9	11	9	10	11	13	14
45−49	180	11	13	14	16	18	15	17	19	21	24	21	24	26	29	32	28	31	34	38	42
	160	7	8	9	10	11	10	11	12	14	15	13	15	17	19	21	18	20	23	25	28
	140	4	5	5	6	7	6	7	7	8	9	8	9	10	12	13	11	13	14	16	18
	120	2	3	3	4	4	3	4	4	5	6	5	6	6	7	8	7	8	9	10	11
40−44	180	9	10	11	12	14	12	13	15	17	19	17	19	21	23	26	22	25	28	31	34
	160	5	6	7	7	8	7	8	9	10	12	10	11	13	14	16	14	16	17	19	22
	140	3	3	4	4	5	4	5	5	6	7	6	7	8	9	10	8	9	11	12	13
	120	1	2	2	2	3	2	3	3	3	4	3	4	4	5	6	5	5	6	7	8

Chile

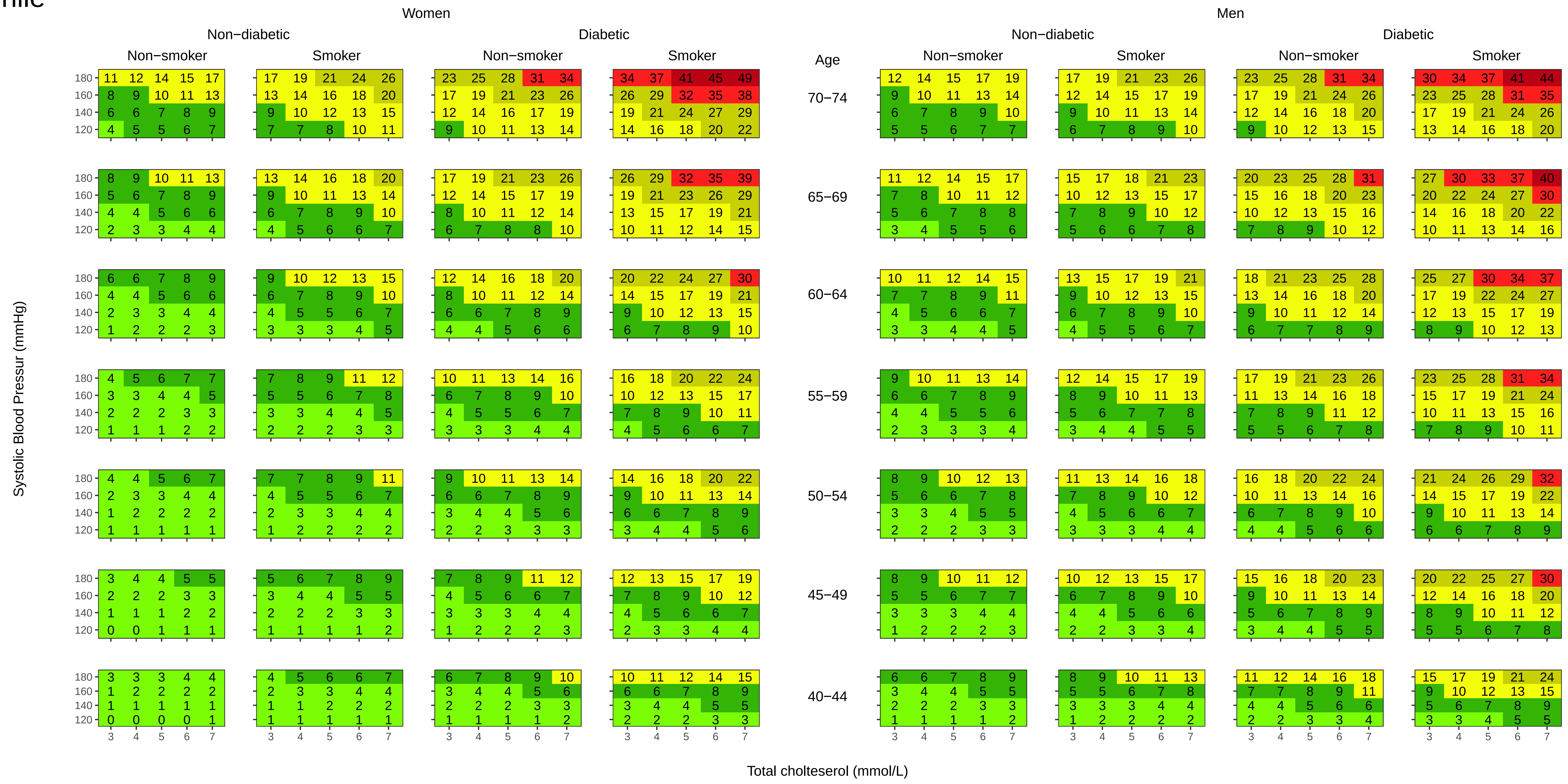
Women
Men
Non−diabetic
Diabetic
Non−smoker
Smoker
Age
70−74
65−69
60−64
55−59
50−54
45−49
40−44
180
160
140
120
3
4
5
6
7
Systolic Blood Pressur (mmHg)
Total cholteserol (mmol/L)

Colombia

Total cholteserol (mmol/L) by Systolic Blood Pressur (mmHg)

Women

Age	Systolic Blood Pressur (mmHg)	Non−diabetic Non−smoker					Non−diabetic Smoker					Diabetic Non−smoker					Diabetic Smoker				
		3	4	5	6	7	3	4	5	6	7	3	4	5	6	7	3	4	5	6	7
70−74	180	23	26	29	32	35	35	38	42	46	50	44	48	52	56	61	60	65	69	74	78
	160	17	19	22	24	27	27	29	33	36	39	34	38	41	45	49	49	53	57	62	66
	140	13	14	16	18	20	20	22	25	27	30	26	29	32	35	39	38	42	46	50	54
	120	9	10	12	13	15	15	16	18	21	23	19	22	24	27	30	30	33	36	40	43
65−69	180	19	22	24	27	30	29	32	36	39	43	38	41	45	49	53	53	57	62	66	71
	160	14	16	17	19	22	22	24	27	30	33	28	31	34	38	41	41	45	49	53	58
	140	10	11	12	14	16	15	17	19	22	24	20	23	25	28	31	31	34	38	41	45
	120	7	8	9	10	11	11	12	14	16	17	15	16	18	21	23	23	25	28	31	34
60−64	180	16	18	20	22	24	24	27	30	33	36	31	34	38	42	46	45	49	54	58	62
	160	11	12	14	15	17	17	19	21	24	26	22	25	28	30	34	33	37	40	44	48
	140	7	8	9	10	12	12	13	15	17	18	16	17	19	22	24	24	27	30	33	36
	120	5	6	6	7	8	8	9	10	11	13	11	12	14	15	17	17	19	21	23	26
55−59	180	13	15	16	18	21	20	23	25	28	31	27	29	33	36	40	39	43	47	51	56
	160	9	10	11	12	14	14	15	17	19	22	18	20	23	25	28	28	31	34	37	41
	140	6	6	7	8	9	9	10	12	13	15	12	14	15	17	19	19	21	24	26	29
	120	4	4	5	5	6	6	7	8	9	10	8	9	10	11	13	13	14	16	18	20
50−54	180	12	13	15	17	19	19	21	23	26	29	25	27	30	33	37	37	40	44	48	52
	160	8	9	10	11	12	12	14	15	17	19	16	18	20	23	25	25	28	31	34	37
	140	5	5	6	7	8	8	9	10	11	12	10	12	13	15	17	16	18	21	23	25
	120	3	3	4	4	5	5	5	6	7	8	7	7	8	9	11	11	12	13	15	17
45−49	180	10	12	13	15	16	16	18	20	23	25	21	24	26	29	32	32	35	39	43	47
	160	6	7	8	9	10	10	11	13	14	16	13	15	17	19	21	21	23	26	29	32
	140	4	4	5	5	6	6	7	8	9	10	8	9	11	12	13	13	15	17	19	21
	120	2	2	3	3	4	4	4	5	5	6	5	6	6	7	8	8	9	10	12	13
40−44	180	8	9	10	11	13	13	14	16	18	20	17	19	21	24	26	26	29	32	35	39
	160	5	5	6	7	8	8	9	10	11	12	10	12	13	15	16	16	18	20	23	25
	140	3	3	3	4	4	4	5	6	7	7	6	7	8	9	10	10	11	12	14	16
	120	1	2	2	2	2	2	3	3	4	4	3	4	5	5	6	6	7	7	8	9

Men

Age	Systolic Blood Pressur (mmHg)	Non−diabetic Non−smoker					Non−diabetic Smoker					Diabetic Non−smoker					Diabetic Smoker				
		3	4	5	6	7	3	4	5	6	7	3	4	5	6	7	3	4	5	6	7
70−74	180	26	29	32	36	39	35	38	42	46	50	45	49	54	58	62	57	61	65	70	74
	160	20	22	25	27	30	26	29	32	36	39	35	39	43	46	51	45	49	54	58	62
	140	15	16	18	20	23	20	22	25	27	30	27	30	33	36	40	35	39	43	47	51
	120	11	12	13	15	17	15	16	18	20	23	20	22	25	28	31	27	30	33	36	40
65−69	180	25	28	31	34	38	33	37	40	44	48	43	47	52	56	60	55	59	63	68	72
	160	18	20	23	25	28	24	27	30	33	37	33	36	40	44	48	42	46	51	55	59
	140	13	15	16	18	20	18	20	22	25	27	24	27	30	33	36	32	35	39	43	46
	120	9	10	12	13	15	13	14	16	18	20	17	20	22	24	27	24	26	29	32	35
60−64	180	23	26	29	32	35	31	34	38	41	45	41	45	49	53	57	52	56	61	65	69
	160	16	18	20	23	25	22	25	27	30	33	30	33	36	40	44	39	43	47	51	55
	140	11	13	14	16	18	15	17	19	22	24	21	24	26	29	32	28	31	35	38	42
	120	8	9	10	11	12	11	12	13	15	17	15	17	18	21	23	20	22	25	28	31
55−59	180	21	24	26	29	32	28	31	35	38	42	38	41	45	49	54	48	52	57	61	66
	160	14	16	18	20	22	20	22	24	27	30	27	29	33	36	39	35	38	42	46	50
	140	10	11	12	14	15	13	15	17	18	21	18	20	23	25	28	24	27	30	33	37
	120	6	7	8	9	10	9	10	11	12	14	12	14	15	17	19	17	19	21	23	26
50−54	180	19	21	24	26	29	26	28	31	35	38	34	38	41	45	49	44	48	52	57	61
	160	12	14	16	18	20	17	19	21	24	26	23	26	29	32	35	31	34	37	41	45
	140	8	9	10	11	13	11	12	14	16	17	15	17	19	21	24	21	23	26	28	31
	120	5	6	6	7	8	7	8	9	10	11	10	11	12	14	16	13	15	17	19	21
45−49	180	16	18	20	22	25	22	24	27	30	33	29	32	36	39	43	38	42	46	50	54
	160	10	11	13	14	16	14	15	17	19	22	19	21	24	26	29	26	28	31	35	38
	140	6	7	8	9	10	9	10	11	12	14	12	13	15	17	19	16	18	20	23	25
	120	4	4	5	5	6	5	6	7	8	8	7	8	9	11	12	10	12	13	15	16
40−44	180	11	13	14	16	18	16	18	20	22	24	22	24	27	30	33	29	32	35	39	42
	160	7	8	9	10	11	9	11	12	14	15	13	15	17	19	21	18	20	23	25	28
	140	4	4	5	6	7	6	6	7	8	9	8	9	10	11	13	11	12	14	16	17
	120	2	2	3	3	4	3	4	4	5	5	5	5	6	7	8	7	7	8	9	11

Costa Rica

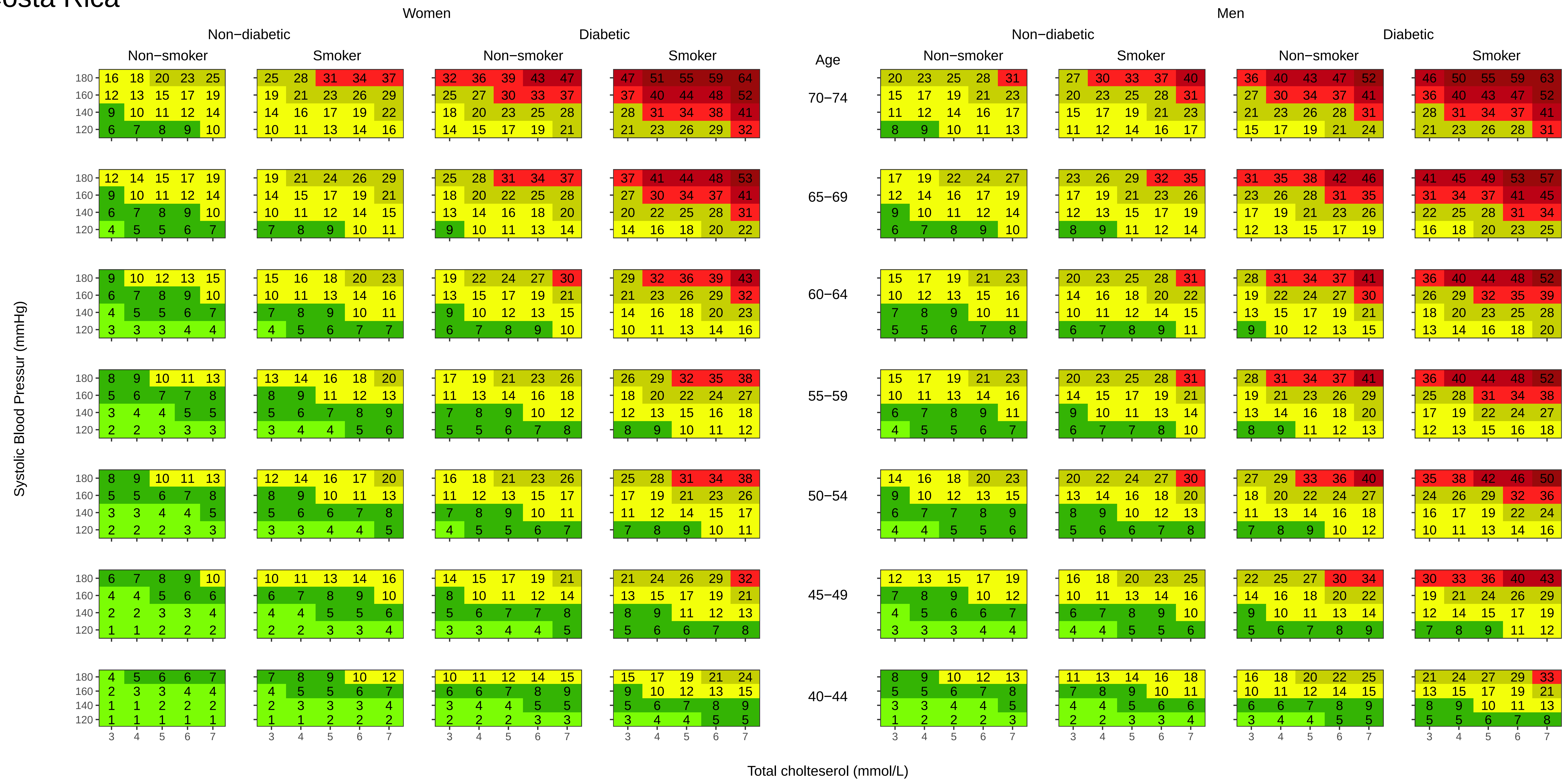
Women
Men
Non−diabetic
Diabetic
Non−smoker
Smoker
Age
70−74
65−69
60−64
55−59
50−54
45−49
40−44
Systolic Blood Pressur (mmHg)
Total cholteserol (mmol/L)

Cuba

Women

Age	Systolic Blood Pressur (mmHg)	Non−diabetic										Diabetic									
		Non−smoker					Smoker					Non−smoker					Smoker				
		3	4	5	6	7	3	4	5	6	7	3	4	5	6	7	3	4	5	6	7
70−74	180	22	25	27	30	33	33	37	40	44	48	42	46	50	54	59	58	63	67	72	76
	160	16	18	20	23	25	25	28	31	34	38	32	36	39	43	47	47	51	55	60	64
	140	12	14	15	17	19	19	21	23	26	29	25	27	30	33	37	37	40	44	48	52
	120	9	10	11	12	14	14	16	17	19	22	18	21	23	25	28	28	31	34	38	41
65−69	180	18	20	23	25	28	28	31	34	37	41	35	39	43	47	51	51	55	59	64	68
	160	13	14	16	18	20	20	22	25	28	31	26	29	32	36	39	39	43	47	51	55
	140	9	10	12	13	15	14	16	18	20	23	19	21	24	26	29	29	32	35	39	43
	120	6	7	8	9	10	10	11	13	15	16	14	15	17	19	21	21	24	26	29	32
60−64	180	16	17	19	22	24	24	27	30	33	36	31	34	38	41	45	45	49	53	58	62
	160	11	12	13	15	17	17	19	21	23	26	22	25	27	30	33	33	37	40	44	48
	140	7	8	9	10	12	12	13	15	16	18	15	17	19	21	24	24	26	29	32	36
	120	5	5	6	7	8	8	9	10	11	13	11	12	13	15	17	17	19	21	23	26
55−59	180	13	14	16	18	20	20	22	24	27	30	26	28	31	35	38	38	42	46	50	54
	160	8	9	10	12	13	13	15	17	19	21	17	20	22	24	27	27	30	33	36	40
	140	5	6	7	8	9	9	10	11	12	14	12	13	15	17	18	18	20	23	25	28
	120	3	4	4	5	6	6	6	7	8	9	8	9	10	11	12	12	14	15	17	19
50−54	180	10	11	12	14	15	15	17	19	21	24	20	23	25	28	31	31	34	37	41	45
	160	6	7	8	9	10	10	11	13	14	16	13	15	17	19	21	21	23	26	28	31
	140	4	4	5	6	6	6	7	8	9	10	8	10	11	12	14	13	15	17	19	21
	120	2	3	3	3	4	4	4	5	6	6	5	6	7	8	9	9	10	11	12	14
45−49	180	7	8	9	10	11	11	13	14	16	18	15	17	19	21	24	23	26	29	32	35
	160	4	5	5	6	7	7	8	9	10	11	9	11	12	13	15	15	17	19	21	23
	140	2	3	3	4	4	4	5	5	6	7	6	6	7	8	9	9	10	12	13	15
	120	1	2	2	2	2	2	3	3	4	4	3	4	4	5	6	6	6	7	8	9
40−44	180	4	5	6	7	7	7	8	9	11	12	10	11	13	14	16	16	18	20	22	24
	160	2	3	3	4	4	4	5	6	6	7	6	7	8	9	10	10	11	12	14	15
	140	1	2	2	2	2	2	3	3	4	4	3	4	4	5	6	6	6	7	8	9
	120	1	1	1	1	1	1	1	2	2	2	2	2	2	3	3	3	4	4	5	5

Men

Age	Systolic Blood Pressur (mmHg)	Non−diabetic										Diabetic									
		Non−smoker					Smoker					Non−smoker					Smoker				
		3	4	5	6	7	3	4	5	6	7	3	4	5	6	7	3	4	5	6	7
70−74	180	26	29	32	35	38	34	37	41	45	49	44	48	53	57	61	56	60	65	69	73
	160	19	22	24	27	30	26	29	32	35	39	34	38	42	46	50	44	48	53	57	61
	140	14	16	18	20	22	19	22	24	27	30	26	29	32	36	39	35	38	42	46	50
	120	10	12	13	15	17	14	16	18	20	22	20	22	24	27	30	26	29	32	36	39
65−69	180	23	26	29	32	35	31	34	37	41	45	41	44	48	53	57	51	56	60	65	69
	160	17	19	21	23	26	23	25	28	31	34	30	34	37	41	45	40	43	47	52	56
	140	12	13	15	17	19	16	18	20	23	25	22	25	28	30	34	30	33	36	40	43
	120	8	9	11	12	13	12	13	15	16	18	16	18	20	22	25	22	24	27	30	33
60−64	180	22	24	27	30	33	29	32	35	39	43	38	42	46	50	54	49	53	58	62	66
	160	15	17	19	21	24	21	23	25	28	31	28	31	34	37	41	36	40	44	48	52
	140	10	12	13	15	17	14	16	18	20	22	20	22	24	27	30	26	29	32	36	39
	120	7	8	9	10	11	10	11	12	14	16	14	15	17	19	21	19	21	23	26	28
55−59	180	20	22	25	27	30	27	29	33	36	40	35	39	43	47	51	46	50	54	58	63
	160	13	15	17	19	21	18	20	23	25	28	25	27	30	34	37	33	36	40	43	47
	140	9	10	11	13	14	12	14	15	17	19	17	19	21	23	26	23	25	28	31	34
	120	6	6	7	8	9	8	9	10	11	13	11	13	14	16	18	15	17	19	22	24
50−54	180	17	19	22	24	27	23	26	29	32	35	31	35	38	42	46	41	45	49	53	57
	160	11	13	14	16	18	15	17	19	21	24	21	23	26	29	32	28	31	34	38	42
	140	7	8	9	10	11	10	11	12	14	16	14	15	17	19	22	19	21	23	26	29
	120	4	5	6	6	7	6	7	8	9	10	9	10	11	13	14	12	14	15	17	19
45−49	180	13	15	17	19	21	18	20	22	25	28	25	27	30	33	37	33	36	39	43	47
	160	8	9	10	12	13	11	13	14	16	18	16	18	20	22	24	21	24	26	29	32
	140	5	6	6	7	8	7	8	9	10	11	10	11	12	14	16	13	15	17	19	21
	120	3	3	4	4	5	4	5	5	6	7	6	7	8	9	10	8	9	11	12	13
40−44	180	9	10	12	13	15	13	14	16	18	20	18	20	22	25	27	24	27	29	33	36
	160	5	6	7	8	9	8	9	10	11	12	11	12	14	15	17	15	17	19	21	23
	140	3	4	4	5	5	4	5	6	6	7	6	7	8	9	10	9	10	11	13	14
	120	2	2	2	3	3	2	3	3	4	4	4	4	5	5	6	5	6	7	8	9

Total cholteserol (mmol/L)

Dominican Republic

Systolic Blood Pressur (mmHg) by Total cholteserol (mmol/L)

Women — Non−diabetic — Non−smoker

Age	Systolic Blood Pressur (mmHg)	3	4	5	6	7
70−74	180	23	25	28	31	34
	160	17	19	21	24	26
	140	12	14	16	18	20
	120	9	10	11	13	14
65−69	180	19	22	24	27	30
	160	14	16	17	19	22
	140	10	11	12	14	16
	120	7	8	9	10	11
60−64	180	17	19	21	23	26
	160	12	13	15	16	18
	140	8	9	10	11	13
	120	5	6	7	8	9
55−59	180	17	19	21	23	26
	160	11	12	14	16	17
	140	7	8	9	10	12
	120	5	5	6	7	8
50−54	180	15	17	19	21	23
	160	9	11	12	13	15
	140	6	7	8	9	10
	120	4	4	5	5	6
45−49	180	12	13	15	17	19
	160	7	8	9	10	12
	140	4	5	6	6	7
	120	2	3	3	4	4
40−44	180	10	11	12	14	15
	160	6	6	7	8	9
	140	3	4	4	5	5
	120	2	2	2	3	3

Women — Non−diabetic — Smoker

Age	Systolic Blood Pressur (mmHg)	3	4	5	6	7
70−74	180	34	38	41	45	49
	160	26	29	32	35	39
	140	19	22	24	27	30
	120	14	16	18	20	22
65−69	180	29	33	36	40	43
	160	22	24	27	30	33
	140	16	17	19	22	24
	120	11	12	14	16	17
60−64	180	26	29	32	35	38
	160	18	20	23	25	28
	140	13	14	16	18	20
	120	8	10	11	12	14
55−59	180	25	28	31	35	38
	160	17	19	22	24	27
	140	12	13	15	16	18
	120	8	9	10	11	12
50−54	180	23	25	28	31	34
	160	15	17	19	21	23
	140	10	11	12	14	15
	120	6	7	8	9	10
45−49	180	18	21	23	26	28
	160	12	13	15	16	18
	140	7	8	9	10	12
	120	4	5	5	6	7
40−44	180	15	17	19	21	24
	160	9	10	12	13	15
	140	5	6	7	8	9
	120	3	3	4	5	5

Women — Diabetic — Non−smoker

Age	Systolic Blood Pressur (mmHg)	3	4	5	6	7
70−74	180	43	47	51	55	60
	160	33	37	40	44	48
	140	25	28	31	34	38
	120	19	21	24	26	29
65−69	180	38	41	45	49	53
	160	28	31	34	38	41
	140	20	23	25	28	31
	120	15	16	18	21	23
60−64	180	33	37	40	44	48
	160	24	26	29	32	36
	140	17	19	21	23	26
	120	11	13	14	16	18
55−59	180	33	36	40	44	48
	160	23	25	28	31	34
	140	15	17	19	22	24
	120	10	12	13	15	16
50−54	180	30	33	36	40	44
	160	20	22	25	27	30
	140	13	15	16	18	20
	120	8	9	10	12	13
45−49	180	24	27	30	33	36
	160	15	17	19	22	24
	140	10	11	12	14	15
	120	6	7	7	8	10
40−44	180	20	22	25	28	31
	160	12	14	16	17	19
	140	7	8	9	11	12
	120	4	5	5	6	7

Women — Diabetic — Smoker

Age	Systolic Blood Pressur (mmHg)	3	4	5	6	7
70−74	180	60	64	68	73	77
	160	48	52	57	61	65
	140	38	41	45	49	54
	120	29	32	35	39	43
65−69	180	53	58	62	66	71
	160	41	45	49	53	58
	140	31	34	38	41	45
	120	23	25	28	31	34
60−64	180	48	52	56	61	65
	160	35	39	43	47	51
	140	26	28	31	35	38
	120	18	20	22	25	28
55−59	180	47	51	56	60	65
	160	34	38	41	45	49
	140	24	26	29	32	36
	120	16	18	20	23	25
50−54	180	43	47	51	56	60
	160	30	33	37	40	44
	140	20	23	25	28	31
	120	13	15	17	19	21
45−49	180	36	40	43	47	52
	160	24	26	29	32	36
	140	15	17	19	21	24
	120	9	11	12	13	15
40−44	180	31	34	37	41	45
	160	19	22	24	27	30
	140	12	13	15	17	19
	120	7	8	9	10	11

Men — Non−diabetic — Non−smoker

Age	Systolic Blood Pressur (mmHg)	3	4	5	6	7
70−74	180	25	28	31	34	37
	160	19	21	23	26	28
	140	14	15	17	19	21
	120	10	11	13	14	16
65−69	180	25	28	31	34	38
	160	18	21	23	25	28
	140	13	15	17	18	21
	120	9	10	12	13	15
60−64	180	25	28	31	34	38
	160	18	20	22	25	27
	140	12	14	15	17	19
	120	8	9	11	12	13
55−59	180	25	28	31	35	38
	160	17	19	22	24	27
	140	12	13	15	16	18
	120	8	9	10	11	12
50−54	180	23	26	28	31	35
	160	15	17	19	21	24
	140	10	11	12	14	16
	120	6	7	8	9	10
45−49	180	19	21	23	26	29
	160	12	13	15	17	19
	140	7	8	9	10	12
	120	4	5	6	6	7
40−44	180	13	14	16	18	20
	160	8	9	10	11	12
	140	4	5	6	7	7
	120	2	3	3	4	4

Men — Non−diabetic — Smoker

Age	Systolic Blood Pressur (mmHg)	3	4	5	6	7
70−74	180	33	36	40	44	48
	160	25	28	31	34	37
	140	19	21	23	26	29
	120	14	15	17	19	21
65−69	180	34	37	41	44	48
	160	25	27	30	34	37
	140	18	20	22	25	28
	120	13	14	16	18	20
60−64	180	33	37	40	44	48
	160	24	27	29	33	36
	140	17	19	21	23	26
	120	12	13	15	16	18
55−59	180	34	37	41	45	49
	160	23	26	29	32	35
	140	16	18	20	22	25
	120	11	12	13	15	17
50−54	180	31	34	37	41	45
	160	21	23	25	28	31
	140	13	15	17	19	21
	120	9	10	11	12	14
45−49	180	25	28	31	34	38
	160	16	18	20	22	25
	140	10	11	13	14	16
	120	6	7	8	9	10
40−44	180	17	20	22	24	27
	160	11	12	13	15	17
	140	6	7	8	9	10
	120	4	4	5	5	6

Men — Diabetic — Non−smoker

Age	Systolic Blood Pressur (mmHg)	3	4	5	6	7
70−74	180	43	47	51	55	60
	160	33	37	40	44	48
	140	25	28	31	34	38
	120	19	21	24	26	29
65−69	180	44	48	52	56	61
	160	33	36	40	44	48
	140	24	27	30	33	37
	120	18	20	22	24	27
60−64	180	44	48	52	56	61
	160	32	35	39	43	47
	140	23	25	28	31	35
	120	16	18	20	22	25
55−59	180	44	48	52	56	61
	160	31	35	38	42	46
	140	22	24	27	30	33
	120	15	16	18	21	23
50−54	180	40	44	48	52	57
	160	28	31	34	37	41
	140	18	21	23	26	28
	120	12	13	15	17	19
45−49	180	34	37	41	44	49
	160	22	25	27	30	33
	140	14	16	18	20	22
	120	9	10	11	12	14
40−44	180	24	27	29	32	36
	160	15	17	19	21	23
	140	9	10	11	13	14
	120	5	6	7	8	9

Men — Diabetic — Smoker

Age	Systolic Blood Pressur (mmHg)	3	4	5	6	7
70−74	180	54	59	63	67	72
	160	43	47	51	55	60
	140	33	37	40	44	48
	120	25	28	31	34	38
65−69	180	55	60	64	68	73
	160	43	47	51	55	60
	140	32	36	39	43	47
	120	24	26	29	32	36
60−64	180	55	59	64	68	72
	160	42	46	50	54	58
	140	30	34	37	41	45
	120	22	24	27	30	33
55−59	180	55	60	64	68	73
	160	41	45	49	53	57
	140	29	32	35	39	43
	120	20	22	25	27	30
50−54	180	51	55	60	64	69
	160	36	40	44	48	52
	140	25	28	31	34	37
	120	16	18	20	23	25
45−49	180	43	47	52	56	60
	160	29	32	36	39	43
	140	19	21	24	26	29
	120	12	14	15	17	19
40−44	180	32	35	38	42	46
	160	20	22	25	28	31
	140	12	14	15	17	19
	120	7	8	9	11	12

Total cholteserol (mmol/L)

Ecuador

Systolic Blood Pressur (mmHg) by Age; Total cholteserol (mmol/L): 3, 4, 5, 6, 7

Women

Age	SBP	Non−diabetic Non−smoker 3	4	5	6	7	Non−diabetic Smoker 3	4	5	6	7	Diabetic Non−smoker 3	4	5	6	7	Diabetic Smoker 3	4	5	6	7
70−74	180	17	19	21	24	27	26	29	32	36	39	34	37	41	45	49	49	53	57	62	66
	160	13	14	16	18	20	20	22	25	27	30	26	29	32	35	39	38	42	46	50	54
	140	9	10	12	13	15	15	16	18	21	23	19	22	24	27	30	29	33	36	40	43
	120	7	8	9	10	11	11	12	14	15	17	14	16	18	20	22	22	25	28	31	34
65−69	180	13	14	16	18	20	20	22	24	27	30	26	28	31	35	38	38	42	45	50	54
	160	9	10	11	13	14	14	16	18	20	22	19	21	23	26	28	28	31	35	38	42
	140	6	7	8	9	10	10	11	13	14	16	13	15	17	19	21	21	23	26	28	31
	120	4	5	5	6	7	7	8	9	10	11	9	11	12	13	15	15	17	19	21	23
60−64	180	10	12	13	15	16	16	18	20	23	25	21	24	26	29	32	32	35	39	43	47
	160	7	8	9	10	11	11	12	14	16	18	15	17	19	21	23	23	25	28	31	35
	140	5	5	6	7	8	8	8	10	11	12	10	11	13	14	16	16	18	20	22	25
	120	3	3	4	4	5	5	6	6	7	8	7	8	9	10	11	11	12	14	16	17
55−59	180	9	10	11	12	14	14	15	17	19	21	18	20	23	25	28	28	31	34	37	41
	160	6	6	7	8	9	9	10	11	13	14	12	14	15	17	19	19	21	24	26	29
	140	4	4	5	5	6	6	7	8	8	10	8	9	10	11	13	13	14	16	18	20
	120	2	2	3	3	4	4	4	5	5	6	5	6	7	7	8	8	9	11	12	13
50−54	180	8	9	11	12	13	13	15	17	19	21	18	20	22	24	27	27	30	33	36	40
	160	5	6	7	8	9	8	10	11	12	14	11	13	14	16	18	18	20	22	25	27
	140	3	4	4	5	5	5	6	7	8	9	7	8	9	10	12	12	13	15	16	18
	120	2	2	2	3	3	3	4	4	5	5	4	5	6	6	7	7	8	9	10	12
45−49	180	7	8	9	10	11	11	13	14	16	18	15	17	19	21	23	23	26	28	31	35
	160	4	5	5	6	7	7	8	9	10	11	9	10	12	13	15	15	16	18	21	23
	140	2	3	3	4	4	4	5	5	6	7	6	6	7	8	9	9	10	12	13	15
	120	1	1	2	2	2	2	3	3	4	4	3	4	4	5	6	5	6	7	8	9
40−44	180	5	6	7	8	9	9	10	11	13	14	12	13	15	17	19	19	21	23	26	29
	160	3	3	4	5	5	5	6	7	7	8	7	8	9	10	11	11	13	14	16	18
	140	2	2	2	3	3	3	3	4	4	5	4	5	5	6	7	7	8	9	10	11
	120	1	1	1	1	2	2	2	2	2	3	2	3	3	3	4	4	4	5	6	6

Men

Age	SBP	Non−diabetic Non−smoker 3	4	5	6	7	Non−diabetic Smoker 3	4	5	6	7	Diabetic Non−smoker 3	4	5	6	7	Diabetic Smoker 3	4	5	6	7
70−74	180	22	25	28	31	34	30	33	36	40	44	39	43	47	51	56	50	54	59	63	68
	160	17	19	21	23	26	23	25	28	31	34	30	34	37	41	44	40	43	47	52	56
	140	12	14	15	17	19	17	19	21	23	26	23	26	28	31	35	30	34	37	41	45
	120	9	10	11	13	14	12	14	16	17	19	17	19	21	24	26	23	26	28	31	35
65−69	180	18	20	23	25	28	25	27	30	33	37	33	36	40	44	48	43	47	51	55	59
	160	13	15	16	18	21	18	20	22	25	27	24	27	30	33	36	32	35	39	43	47
	140	9	10	12	13	15	13	14	16	18	20	18	20	22	24	27	24	26	29	32	36
	120	6	7	8	9	10	9	10	11	13	14	13	14	16	18	20	17	19	21	24	26
60−64	180	17	19	21	24	26	23	26	28	31	35	31	34	38	41	45	40	44	48	52	57
	160	12	13	15	17	18	16	18	20	22	25	22	24	27	30	33	29	32	36	39	43
	140	8	9	10	11	13	11	12	14	16	17	15	17	19	21	24	21	23	26	28	31
	120	5	6	7	8	9	7	8	9	11	12	10	12	13	15	17	14	16	18	20	22
55−59	180	17	19	21	23	26	22	25	28	31	34	30	33	37	40	44	39	43	47	51	56
	160	11	12	14	16	18	15	17	19	21	24	21	23	26	29	32	28	31	34	37	41
	140	7	8	9	10	12	10	11	13	14	16	14	16	18	20	22	19	21	24	26	29
	120	5	5	6	7	8	7	7	8	9	11	9	10	12	13	15	13	14	16	18	20
50−54	180	15	17	19	21	23	20	23	25	28	31	27	30	34	37	41	36	40	43	47	52
	160	10	11	12	14	15	13	15	17	19	21	18	20	23	25	28	25	27	30	33	37
	140	6	7	8	9	10	8	9	11	12	14	12	13	15	17	19	16	18	20	23	25
	120	4	4	5	5	6	5	6	7	8	9	7	8	10	11	12	10	12	13	15	17
45−49	180	12	14	16	17	19	17	19	21	23	26	23	26	28	31	35	31	34	37	41	45
	160	8	9	10	11	12	10	12	13	15	17	15	16	18	20	23	20	22	25	27	30
	140	5	5	6	7	7	6	7	8	9	10	9	10	12	13	15	13	14	16	18	20
	120	3	3	3	4	4	4	4	5	6	6	5	6	7	8	9	8	9	10	11	12
40−44	180	10	11	12	14	15	13	15	17	19	21	18	21	23	25	28	25	27	30	34	37
	160	6	6	7	8	9	8	9	10	11	13	11	13	14	16	18	15	17	19	21	24
	140	3	4	4	5	5	5	5	6	7	8	7	7	8	10	11	9	10	12	13	15
	120	2	2	2	3	3	3	3	3	4	4	4	4	5	6	6	5	6	7	8	9

Total cholteserol (mmol/L)

El Salvador

Systolic Blood Pressur (mmHg) by Total cholteserol (mmol/L)

Women — Non−diabetic

Age	SBP	Non−smoker 3	4	5	6	7	Smoker 3	4	5	6	7
70−74	180	23	26	28	31	35	35	38	42	46	50
	160	17	19	21	24	26	26	29	32	36	39
	140	13	14	16	18	20	20	22	25	27	30
	120	9	10	12	13	15	15	16	18	20	23
65−69	180	17	19	21	24	26	26	29	32	35	39
	160	12	14	15	17	19	19	21	24	26	29
	140	9	10	11	12	14	14	15	17	19	21
	120	6	7	8	9	10	10	11	12	14	15
60−64	180	15	16	18	20	23	22	25	28	31	34
	160	10	11	13	14	16	16	18	20	22	24
	140	7	8	9	10	11	11	12	14	15	17
	120	4	5	6	6	7	7	8	9	10	12
55−59	180	14	16	17	19	22	22	24	27	30	33
	160	9	10	12	13	15	15	16	18	20	23
	140	6	7	8	9	10	10	11	12	14	15
	120	4	4	5	6	6	6	7	8	9	10
50−54	180	13	15	17	19	21	21	23	26	28	32
	160	8	10	11	12	14	14	15	17	19	21
	140	5	6	7	8	9	9	10	11	12	14
	120	3	4	4	5	5	5	6	7	8	9
45−49	180	12	14	15	17	19	19	21	23	26	29
	160	7	8	9	11	12	12	13	15	17	19
	140	4	5	6	6	7	7	8	9	10	12
	120	3	3	3	4	4	4	5	6	6	7
40−44	180	10	12	13	15	16	16	18	20	23	25
	160	6	7	8	9	10	10	11	12	14	16
	140	3	4	4	5	6	6	7	7	8	9
	120	2	2	2	3	3	3	4	4	5	6

Women — Diabetic

Age	SBP	Non−smoker 3	4	5	6	7	Smoker 3	4	5	6	7
70−74	180	44	48	52	56	60	60	65	69	73	77
	160	34	37	41	45	49	49	53	57	62	66
	140	26	29	32	35	38	38	42	46	50	54
	120	19	22	24	27	30	29	32	36	39	43
65−69	180	34	37	41	45	49	48	53	57	61	66
	160	25	28	31	34	37	37	41	44	49	53
	140	18	20	22	25	28	28	30	34	37	41
	120	13	14	16	18	20	20	22	25	28	31
60−64	180	29	32	36	39	43	43	47	51	55	59
	160	21	23	26	28	31	31	34	38	42	46
	140	14	16	18	20	22	22	25	27	30	34
	120	10	11	12	14	16	16	17	19	22	24
55−59	180	28	31	34	38	41	41	45	49	53	58
	160	19	21	24	27	29	29	32	36	39	43
	140	13	14	16	18	20	20	22	25	28	31
	120	8	10	11	12	14	14	15	17	19	21
50−54	180	27	30	33	36	40	40	44	48	52	56
	160	18	20	22	25	28	27	30	34	37	41
	140	12	13	15	16	18	18	20	23	25	28
	120	7	8	9	11	12	12	13	15	17	19
45−49	180	25	27	30	34	37	37	40	44	48	52
	160	16	18	20	22	24	24	27	30	33	36
	140	10	11	12	14	16	16	17	19	22	24
	120	6	7	8	9	10	10	11	12	14	15
40−44	180	21	24	26	29	32	32	35	39	43	47
	160	13	15	16	18	21	20	23	25	28	31
	140	8	9	10	11	13	13	14	16	18	20
	120	5	5	6	7	7	7	8	9	11	12

Men — Non−diabetic

Age	SBP	Non−smoker 3	4	5	6	7	Smoker 3	4	5	6	7
70−74	180	23	25	28	31	34	30	33	36	40	44
	160	17	19	21	23	26	23	25	28	31	34
	140	12	14	16	17	19	17	19	21	23	26
	120	9	10	11	13	14	12	14	16	17	19
65−69	180	20	22	24	27	30	26	29	32	35	39
	160	14	16	18	20	22	19	21	24	26	29
	140	10	11	12	14	16	14	15	17	19	21
	120	7	8	9	10	11	10	11	12	14	15
60−64	180	20	22	25	28	30	27	30	33	36	40
	160	14	16	17	19	22	19	21	23	26	29
	140	9	11	12	13	15	13	15	16	18	20
	120	6	7	8	9	10	9	10	11	13	14
55−59	180	21	23	26	28	31	28	31	34	37	41
	160	14	16	17	19	22	19	21	23	26	29
	140	9	10	12	13	15	13	14	16	18	20
	120	6	7	8	9	10	8	9	11	12	13
50−54	180	20	22	25	27	30	26	29	32	36	39
	160	13	14	16	18	20	18	20	22	24	27
	140	8	9	10	12	13	11	13	14	16	18
	120	5	6	7	7	8	7	8	9	10	12
45−49	180	17	19	22	24	27	23	26	29	32	35
	160	11	12	14	15	17	15	17	19	21	23
	140	7	8	8	10	11	9	10	12	13	15
	120	4	4	5	6	7	6	6	7	8	9
40−44	180	15	16	18	21	23	20	22	25	27	30
	160	9	10	11	13	14	12	14	15	17	19
	140	5	6	7	7	8	7	8	9	10	12
	120	3	3	4	4	5	4	5	5	6	7

Men — Diabetic

Age	SBP	Non−smoker 3	4	5	6	7	Smoker 3	4	5	6	7
70−74	180	40	43	47	51	56	50	55	59	63	68
	160	30	34	37	41	45	40	43	47	52	56
	140	23	26	28	31	35	31	34	37	41	45
	120	17	19	21	24	26	23	26	28	31	35
65−69	180	35	38	42	46	50	45	49	53	58	62
	160	26	29	32	35	39	34	38	41	45	49
	140	19	21	23	26	29	25	28	31	34	38
	120	13	15	17	19	21	18	20	23	25	28
60−64	180	36	39	43	47	51	46	50	54	58	63
	160	26	28	31	35	38	34	37	41	45	49
	140	18	20	22	25	28	24	27	30	33	36
	120	12	14	16	17	20	17	19	21	24	26
55−59	180	37	40	44	48	52	47	51	55	60	64
	160	26	28	32	35	38	34	37	41	45	49
	140	18	20	22	24	27	24	26	29	32	35
	120	12	13	15	17	18	16	18	20	22	25
50−54	180	35	39	43	47	51	45	49	54	58	62
	160	24	27	30	33	36	32	35	39	42	46
	140	16	18	20	22	24	21	24	26	29	32
	120	10	11	13	14	16	14	16	18	20	22
45−49	180	31	35	38	42	46	41	45	49	53	57
	160	21	23	25	28	31	27	30	34	37	41
	140	13	15	16	18	20	18	20	22	24	27
	120	8	9	10	11	13	11	12	14	16	18
40−44	180	27	30	33	36	40	35	39	43	47	51
	160	17	19	21	23	26	23	25	28	31	34
	140	10	11	13	14	16	14	16	18	20	22
	120	6	7	8	9	10	8	9	11	12	13

Grenada

Systolic Blood Pressur (mmHg) by Total cholteserol (mmol/L)

Age 70−74

Women	Non−diabetic Non−smoker					Non−diabetic Smoker					Diabetic Non−smoker					Diabetic Smoker				
SBP \ Chol	3	4	5	6	7	3	4	5	6	7	3	4	5	6	7	3	4	5	6	7
180	24	27	29	33	36	36	39	43	47	51	45	49	53	58	62	62	66	70	75	79
160	18	20	22	25	27	27	30	33	37	40	35	38	42	46	50	50	54	59	63	67
140	13	15	17	18	21	21	23	25	28	31	27	30	33	36	40	39	43	47	51	56
120	10	11	12	14	15	15	17	19	21	24	20	22	25	28	31	30	34	37	41	45

Men	Non−diabetic Non−smoker					Non−diabetic Smoker					Diabetic Non−smoker					Diabetic Smoker				
SBP \ Chol	3	4	5	6	7	3	4	5	6	7	3	4	5	6	7	3	4	5	6	7
180	30	33	36	40	44	39	43	47	51	55	50	54	59	63	67	62	66	71	75	79
160	22	25	28	31	34	30	33	36	40	44	39	43	47	51	56	50	54	59	63	68
140	17	19	21	23	26	23	25	28	31	34	30	33	37	41	44	39	43	47	51	56
120	12	14	15	17	19	17	19	21	23	26	23	25	28	31	35	30	34	37	41	44

Age 65−69

Women	Non−diabetic Non−smoker					Non−diabetic Smoker					Diabetic Non−smoker					Diabetic Smoker				
SBP \ Chol	3	4	5	6	7	3	4	5	6	7	3	4	5	6	7	3	4	5	6	7
180	19	21	24	27	29	29	32	36	39	43	37	41	45	49	53	53	57	62	66	70
160	14	15	17	19	22	21	24	26	29	32	28	31	34	37	41	41	45	49	53	57
140	10	11	12	14	16	15	17	19	22	24	20	23	25	28	31	31	34	37	41	45
120	7	8	9	10	11	11	12	14	15	17	15	16	18	20	23	23	25	28	31	34

Men	Non−diabetic Non−smoker					Non−diabetic Smoker					Diabetic Non−smoker					Diabetic Smoker				
SBP \ Chol	3	4	5	6	7	3	4	5	6	7	3	4	5	6	7	3	4	5	6	7
180	26	29	32	35	39	34	38	42	45	50	45	49	53	57	62	56	61	65	69	74
160	19	21	24	26	29	25	28	31	35	38	34	37	41	45	49	44	48	52	56	61
140	14	15	17	19	21	18	21	23	26	28	25	28	31	34	38	33	37	40	44	48
120	10	11	12	14	15	13	15	17	19	21	18	20	23	25	28	24	27	30	33	37

Age 60−64

Women	Non−diabetic Non−smoker					Non−diabetic Smoker					Diabetic Non−smoker					Diabetic Smoker				
SBP \ Chol	3	4	5	6	7	3	4	5	6	7	3	4	5	6	7	3	4	5	6	7
180	16	18	20	22	25	25	27	30	33	37	32	35	39	42	46	46	50	54	59	63
160	11	12	14	16	17	17	19	22	24	27	23	25	28	31	34	34	37	41	45	49
140	7	8	9	11	12	12	13	15	17	19	16	18	20	22	25	24	27	30	33	37
120	5	6	6	7	8	8	9	10	12	13	11	12	14	15	17	17	19	21	24	26

Men	Non−diabetic Non−smoker					Non−diabetic Smoker					Diabetic Non−smoker					Diabetic Smoker				
SBP \ Chol	3	4	5	6	7	3	4	5	6	7	3	4	5	6	7	3	4	5	6	7
180	24	27	30	33	37	32	36	39	43	47	42	46	50	55	59	53	58	62	67	71
160	17	19	21	24	26	23	26	28	31	35	31	34	38	41	45	40	44	48	52	57
140	12	13	15	17	19	16	18	20	22	25	22	25	27	30	33	29	32	36	39	43
120	8	9	10	11	13	11	12	14	16	18	15	17	19	21	24	21	23	26	29	32

Age 55−59

Women	Non−diabetic Non−smoker					Non−diabetic Smoker					Diabetic Non−smoker					Diabetic Smoker				
SBP \ Chol	3	4	5	6	7	3	4	5	6	7	3	4	5	6	7	3	4	5	6	7
180	14	16	18	20	22	22	24	27	30	33	28	31	35	38	42	42	45	49	54	58
160	9	10	12	13	15	15	16	18	21	23	19	22	24	27	30	29	33	36	40	43
140	6	7	8	9	10	10	11	12	14	16	13	15	16	18	20	20	23	25	28	31
120	4	4	5	6	6	6	7	8	9	10	9	10	11	12	14	14	15	17	19	21

Men	Non−diabetic Non−smoker					Non−diabetic Smoker					Diabetic Non−smoker					Diabetic Smoker				
SBP \ Chol	3	4	5	6	7	3	4	5	6	7	3	4	5	6	7	3	4	5	6	7
180	24	26	29	32	35	31	34	38	42	46	41	45	49	53	58	52	56	61	65	70
160	16	18	20	22	25	22	24	27	30	33	29	32	36	39	43	38	42	46	50	54
140	11	12	13	15	17	15	16	18	20	23	20	22	25	28	31	27	30	33	36	40
120	7	8	9	10	11	10	11	12	14	15	13	15	17	19	21	18	20	23	25	28

Age 50−54

Women	Non−diabetic Non−smoker					Non−diabetic Smoker					Diabetic Non−smoker					Diabetic Smoker				
SBP \ Chol	3	4	5	6	7	3	4	5	6	7	3	4	5	6	7	3	4	5	6	7
180	13	15	16	18	20	20	23	25	28	31	26	29	32	36	39	39	43	47	51	55
160	8	9	11	12	13	13	15	17	19	21	17	20	22	24	27	27	30	33	36	40
140	5	6	7	7	8	8	9	11	12	14	11	13	14	16	18	18	20	22	25	27
120	3	4	4	5	5	5	6	7	8	9	7	8	9	10	12	12	13	15	16	18

Men	Non−diabetic Non−smoker					Non−diabetic Smoker					Diabetic Non−smoker					Diabetic Smoker				
SBP \ Chol	3	4	5	6	7	3	4	5	6	7	3	4	5	6	7	3	4	5	6	7
180	22	25	28	31	34	30	33	36	40	44	39	43	47	51	56	50	54	59	63	67
160	15	16	18	21	23	20	22	25	27	30	27	30	33	36	40	36	39	43	47	51
140	9	11	12	13	15	13	15	16	18	20	18	20	22	25	28	24	27	30	33	36
120	6	7	8	9	10	8	9	11	12	13	12	13	15	16	18	16	18	20	22	25

Age 45−49

Women	Non−diabetic Non−smoker					Non−diabetic Smoker					Diabetic Non−smoker					Diabetic Smoker				
SBP \ Chol	3	4	5	6	7	3	4	5	6	7	3	4	5	6	7	3	4	5	6	7
180	10	11	13	14	16	16	18	20	22	25	21	23	26	29	32	32	35	39	42	46
160	6	7	8	9	10	10	11	13	14	16	13	15	17	19	21	21	23	26	29	32
140	4	4	5	5	6	6	7	8	9	10	8	9	10	12	13	13	15	17	19	21
120	2	2	3	3	4	4	4	5	5	6	5	6	6	7	8	8	9	10	12	13

Men	Non−diabetic Non−smoker					Non−diabetic Smoker					Diabetic Non−smoker					Diabetic Smoker				
SBP \ Chol	3	4	5	6	7	3	4	5	6	7	3	4	5	6	7	3	4	5	6	7
180	17	19	21	24	26	23	26	28	31	35	31	34	38	41	45	40	44	48	52	57
160	11	12	14	15	17	15	16	18	21	23	20	22	25	28	31	27	30	33	36	40
140	7	7	8	9	11	9	10	12	13	15	13	14	16	18	20	17	19	22	24	27
120	4	4	5	6	7	6	6	7	8	9	8	9	10	11	13	11	12	14	16	17

Age 40−44

Women	Non−diabetic Non−smoker					Non−diabetic Smoker					Diabetic Non−smoker					Diabetic Smoker				
SBP \ Chol	3	4	5	6	7	3	4	5	6	7	3	4	5	6	7	3	4	5	6	7
180	8	9	10	11	13	13	14	16	18	20	17	19	21	23	26	26	28	31	35	38
160	5	5	6	7	8	7	8	10	11	12	10	11	13	14	16	16	18	20	22	25
140	3	3	3	4	4	4	5	6	6	7	6	7	8	9	10	10	11	12	14	15
120	1	2	2	2	2	2	3	3	4	4	3	4	4	5	6	6	7	7	8	9

Men	Non−diabetic Non−smoker					Non−diabetic Smoker					Diabetic Non−smoker					Diabetic Smoker				
SBP \ Chol	3	4	5	6	7	3	4	5	6	7	3	4	5	6	7	3	4	5	6	7
180	12	14	15	17	19	17	19	21	23	26	23	25	28	31	34	30	33	37	40	44
160	7	8	9	10	12	10	11	13	14	16	14	16	18	20	22	19	21	24	26	29
140	4	5	5	6	7	6	7	8	9	10	8	9	11	12	14	12	13	15	16	18
120	2	3	3	3	4	3	4	4	5	6	5	6	6	7	8	7	8	9	10	11

Guatemala

Rows: Age / Systolic Blood Pressur (mmHg). Columns: Total cholteserol (mmol/L).

Women – Non−diabetic – Non−smoker

Age	SBP	3	4	5	6	7
70−74	180	18	20	22	25	27
	160	13	15	16	18	21
	140	10	11	12	14	15
	120	7	8	9	10	11
65−69	180	13	15	17	19	21
	160	9	11	12	13	15
	140	7	7	8	9	11
	120	4	5	6	7	7
60−64	180	11	13	14	16	18
	160	8	9	10	11	12
	140	5	6	7	7	8
	120	3	4	4	5	6
55−59	180	10	12	13	15	16
	160	7	8	9	10	11
	140	4	5	6	6	7
	120	3	3	4	4	5
50−54	180	10	12	13	15	16
	160	7	7	8	9	11
	140	4	5	5	6	7
	120	2	3	3	4	4
45−49	180	9	11	12	13	15
	160	6	6	7	8	9
	140	3	4	4	5	6
	120	2	2	3	3	3
40−44	180	7	8	9	10	12
	160	4	5	5	6	7
	140	2	3	3	3	4
	120	1	1	2	2	2

Women – Non−diabetic – Smoker

Age	SBP	3	4	5	6	7
70−74	180	27	30	33	37	40
	160	20	23	25	28	31
	140	15	17	19	21	24
	120	11	13	14	16	18
65−69	180	21	23	26	28	31
	160	15	17	19	21	23
	140	11	12	13	15	17
	120	7	8	9	11	12
60−64	180	18	20	22	25	27
	160	12	14	15	17	19
	140	8	9	11	12	13
	120	6	6	7	8	9
55−59	180	16	18	20	23	25
	160	11	12	14	15	17
	140	7	8	9	10	12
	120	5	5	6	7	8
50−54	180	16	18	20	23	25
	160	11	12	13	15	17
	140	7	8	8	10	11
	120	4	5	5	6	7
45−49	180	15	17	19	21	23
	160	9	10	12	13	15
	140	6	6	7	8	9
	120	3	4	4	5	6
40−44	180	12	13	15	16	18
	160	7	8	9	10	11
	140	4	4	5	6	7
	120	2	3	3	3	4

Women – Diabetic – Non−smoker

Age	SBP	3	4	5	6	7
70−74	180	35	38	42	46	50
	160	27	30	33	36	40
	140	20	22	25	28	31
	120	15	17	19	21	23
65−69	180	27	30	33	36	40
	160	20	22	24	27	30
	140	14	16	18	20	22
	120	10	11	13	14	16
60−64	180	23	26	29	32	35
	160	16	18	20	23	25
	140	11	13	14	16	18
	120	8	9	10	11	12
55−59	180	22	24	27	29	33
	160	15	16	18	20	23
	140	10	11	12	14	15
	120	6	7	8	9	10
50−54	180	22	24	27	30	33
	160	14	16	18	20	22
	140	9	10	11	13	14
	120	6	6	7	8	9
45−49	180	20	22	24	27	30
	160	12	14	16	18	20
	140	8	9	10	11	12
	120	5	5	6	7	8
40−44	180	15	17	19	21	24
	160	9	10	12	13	15
	140	5	6	7	8	9
	120	3	4	4	5	5

Women – Diabetic – Smoker

Age	SBP	3	4	5	6	7
70−74	180	50	54	58	63	67
	160	39	43	47	51	56
	140	30	34	37	41	45
	120	23	26	28	32	35
65−69	180	40	43	47	52	56
	160	30	33	36	40	44
	140	22	24	27	30	33
	120	16	18	20	22	24
60−64	180	35	38	42	46	50
	160	25	28	31	34	38
	140	18	20	22	25	27
	120	12	14	15	17	19
55−59	180	32	36	39	43	47
	160	23	25	28	31	34
	140	15	17	19	21	24
	120	10	11	13	14	16
50−54	180	32	36	39	43	47
	160	22	24	27	30	33
	140	14	16	18	20	22
	120	9	10	12	13	15
45−49	180	30	33	36	40	44
	160	19	22	24	27	30
	140	12	14	15	17	19
	120	8	9	10	11	12
40−44	180	24	26	29	32	36
	160	15	16	18	21	23
	140	9	10	11	13	14
	120	5	6	7	8	9

Men – Non−diabetic – Non−smoker

Age	SBP	3	4	5	6	7
70−74	180	23	26	29	32	35
	160	17	19	22	24	27
	140	13	14	16	18	20
	120	9	11	12	13	15
65−69	180	18	20	22	25	28
	160	13	14	16	18	20
	140	9	10	12	13	15
	120	6	7	8	9	10
60−64	180	15	17	19	21	23
	160	10	11	13	14	16
	140	7	8	9	10	11
	120	5	5	6	7	8
55−59	180	14	16	18	20	22
	160	9	10	12	13	15
	140	6	7	8	9	10
	120	4	4	5	6	6
50−54	180	14	15	17	19	21
	160	9	10	11	12	14
	140	5	6	7	8	9
	120	3	4	4	5	6
45−49	180	12	14	16	17	19
	160	8	9	10	11	12
	140	5	5	6	7	8
	120	3	3	3	4	5
40−44	180	10	12	13	15	16
	160	6	7	8	9	10
	140	3	4	4	5	6
	120	2	2	2	3	3

Men – Non−diabetic – Smoker

Age	SBP	3	4	5	6	7
70−74	180	31	34	38	41	45
	160	23	26	29	32	35
	140	18	20	22	24	27
	120	13	15	16	18	20
65−69	180	24	27	30	33	36
	160	18	20	22	24	27
	140	13	14	16	18	20
	120	9	10	11	13	14
60−64	180	20	22	25	28	31
	160	14	16	18	20	22
	140	10	11	12	14	15
	120	6	7	8	9	10
55−59	180	19	21	24	26	29
	160	13	14	16	18	20
	140	8	9	11	12	14
	120	5	6	7	8	9
50−54	180	18	21	23	25	28
	160	12	13	15	17	19
	140	8	9	10	11	12
	120	5	5	6	7	8
45−49	180	17	19	21	23	26
	160	11	12	13	15	17
	140	6	7	8	9	10
	120	4	4	5	6	6
40−44	180	14	16	18	20	22
	160	8	9	11	12	14
	140	5	6	6	7	8
	120	3	3	4	4	5

Men – Diabetic – Non−smoker

Age	SBP	3	4	5	6	7
70−74	180	41	45	49	53	57
	160	31	35	38	42	46
	140	24	27	29	33	36
	120	18	20	22	25	28
65−69	180	32	36	39	43	47
	160	24	27	29	33	36
	140	17	19	22	24	27
	120	12	14	16	17	20
60−64	180	27	30	33	37	40
	160	19	22	24	27	29
	140	13	15	17	19	21
	120	9	10	12	13	15
55−59	180	26	29	32	35	39
	160	18	20	22	25	27
	140	12	13	15	17	19
	120	8	9	10	11	13
50−54	180	25	28	31	34	37
	160	17	18	21	23	26
	140	11	12	13	15	17
	120	7	8	9	10	11
45−49	180	23	26	28	31	35
	160	15	16	18	21	23
	140	9	10	12	13	15
	120	6	6	7	8	9
40−44	180	19	22	24	27	30
	160	12	13	15	17	19
	140	7	8	9	10	11
	120	4	5	5	6	7

Men – Diabetic – Smoker

Age	SBP	3	4	5	6	7
70−74	180	52	56	60	65	69
	160	41	45	49	53	57
	140	32	35	38	42	46
	120	24	27	30	33	36
65−69	180	42	46	50	54	59
	160	32	35	38	42	46
	140	23	26	29	32	35
	120	17	19	21	24	26
60−64	180	36	39	43	47	51
	160	26	29	32	35	38
	140	18	20	23	25	28
	120	13	14	16	18	20
55−59	180	34	38	41	45	49
	160	24	26	29	32	36
	140	16	18	20	23	25
	120	11	12	14	15	17
50−54	180	33	36	40	44	48
	160	22	25	28	31	34
	140	15	16	18	20	23
	120	9	11	12	13	15
45−49	180	31	34	37	41	45
	160	20	22	25	27	30
	140	13	14	16	18	20
	120	8	9	10	11	13
40−44	180	26	29	32	35	39
	160	16	18	20	23	25
	140	10	11	12	14	16
	120	6	7	7	8	9

Total cholteserol (mmol/L)

Guyana

Women

Age	Systolic Blood Pressur (mmHg)	Non−diabetic Non−smoker 3	4	5	6	7	Non−diabetic Smoker 3	4	5	6	7	Diabetic Non−smoker 3	4	5	6	7	Diabetic Smoker 3	4	5	6	7
70−74	180	40	43	47	52	56	56	60	64	69	73	67	71	75	79	83	83	86	89	92	94
	160	31	34	37	41	45	45	49	53	57	61	55	59	64	68	72	72	76	80	84	87
	140	23	26	29	32	35	35	38	42	46	50	44	48	52	56	61	60	65	69	74	78
	120	17	19	22	24	27	27	29	33	36	39	34	38	41	45	49	49	53	58	62	66
65−69	180	31	35	38	42	46	45	49	54	58	62	56	60	65	69	73	73	77	81	85	88
	160	23	26	28	31	35	35	38	42	46	50	44	48	52	56	60	60	65	69	73	77
	140	17	19	21	23	26	26	28	31	35	38	33	36	40	44	48	48	52	56	60	65
	120	12	13	15	17	19	19	21	23	26	29	24	27	30	33	37	36	40	44	48	52
60−64	180	26	28	31	35	38	38	42	46	50	54	47	52	56	60	65	64	69	73	77	81
	160	18	20	22	25	28	27	30	34	37	41	35	39	43	46	51	50	55	59	63	68
	140	12	14	16	18	20	19	22	24	27	30	25	28	31	34	38	38	41	45	49	54
	120	8	10	11	12	14	14	15	17	19	21	18	20	22	25	28	27	30	33	37	41
55−59	180	23	25	28	31	34	34	37	41	45	49	43	47	51	55	59	59	64	68	72	77
	160	15	17	19	21	24	24	26	29	32	35	31	34	37	41	45	44	48	53	57	61
	140	10	11	13	14	16	16	18	20	22	25	21	24	26	29	32	32	35	39	42	46
	120	7	7	8	10	11	11	12	14	15	17	14	16	18	20	22	22	25	27	30	33
50−54	180	18	20	22	25	27	27	30	33	37	40	35	38	42	46	50	50	54	59	63	67
	160	12	13	15	16	18	18	20	23	25	28	24	26	29	32	36	36	39	43	47	51
	140	7	8	9	11	12	12	13	15	17	19	16	18	20	22	24	24	27	30	33	36
	120	5	5	6	7	8	7	8	10	11	12	10	11	13	14	16	16	18	20	22	25
45−49	180	14	15	17	19	21	21	24	26	29	32	28	31	34	37	41	41	45	49	53	57
	160	8	10	11	12	14	14	15	17	19	21	18	20	22	25	28	27	30	33	37	41
	140	5	6	7	7	8	8	9	11	12	13	11	13	14	16	18	18	20	22	24	27
	120	3	3	4	4	5	5	6	6	7	8	7	8	9	10	11	11	12	14	16	18
40−44	180	11	13	14	16	18	18	20	22	25	27	23	26	29	32	35	35	38	42	46	50
	160	7	8	9	10	11	11	12	14	15	17	14	16	18	20	22	22	25	27	30	34
	140	4	4	5	6	6	6	7	8	9	10	9	10	11	12	14	14	15	17	19	21
	120	2	2	3	3	4	4	4	5	5	6	5	6	6	7	8	8	9	10	12	13

Men

Age	Systolic Blood Pressur (mmHg)	Non−diabetic Non−smoker 3	4	5	6	7	Non−diabetic Smoker 3	4	5	6	7	Diabetic Non−smoker 3	4	5	6	7	Diabetic Smoker 3	4	5	6	7
70−74	180	50	55	59	63	68	62	66	71	75	79	74	78	82	86	89	85	88	91	93	95
	160	40	44	48	52	56	50	55	59	64	68	63	67	72	76	80	75	79	82	86	89
	140	31	34	37	41	45	40	44	48	52	56	51	56	60	64	69	63	68	72	76	80
	120	23	26	29	32	35	31	34	38	41	45	41	44	48	53	57	51	56	60	65	69
65−69	180	41	45	49	53	58	52	56	61	65	70	65	69	73	77	81	76	80	84	87	90
	160	31	34	38	41	45	40	44	48	52	57	52	56	60	65	69	64	68	72	76	80
	140	23	25	28	31	34	30	33	37	40	44	40	44	48	52	56	51	55	59	64	68
	120	16	18	21	23	25	22	25	27	30	34	30	33	36	40	44	39	43	47	51	55
60−64	180	35	38	42	46	50	45	49	53	58	62	57	61	66	70	74	69	73	77	81	85
	160	25	28	31	34	38	33	37	40	44	48	43	47	52	56	60	55	59	63	68	72
	140	18	20	22	25	27	24	26	29	32	36	32	35	39	43	46	41	45	49	54	58
	120	12	14	15	17	19	17	19	21	23	26	23	25	28	31	34	30	33	37	41	44
55−59	180	32	35	39	43	47	42	46	50	54	58	53	58	62	66	71	65	70	74	78	82
	160	22	25	27	30	34	30	33	36	40	43	39	43	47	51	55	50	54	58	63	67
	140	15	17	19	21	23	20	23	25	28	31	28	31	34	37	41	36	40	44	48	52
	120	10	11	13	14	16	14	15	17	19	21	19	21	24	26	29	25	28	31	34	38
50−54	180	28	31	34	38	41	37	40	44	48	52	48	52	56	61	65	59	64	68	72	77
	160	19	21	23	26	29	25	28	31	34	38	34	37	41	44	49	43	47	52	56	60
	140	12	14	15	17	19	17	19	21	23	26	23	25	28	31	34	30	33	37	40	44
	120	8	9	10	11	12	11	12	14	15	17	15	17	19	21	23	20	23	25	28	31
45−49	180	21	24	26	29	32	28	31	35	38	42	37	41	45	49	53	48	52	56	61	65
	160	13	15	17	19	21	18	20	23	25	28	25	28	31	34	37	33	36	40	44	48
	140	8	9	11	12	13	12	13	15	16	18	16	18	20	22	25	22	24	27	30	33
	120	5	6	6	7	8	7	8	9	10	11	10	11	13	14	16	14	15	17	19	22
40−44	180	13	15	17	19	21	18	20	22	25	28	25	27	30	33	37	32	36	39	43	47
	160	8	9	10	11	13	11	12	14	16	17	15	17	19	21	24	21	23	26	28	31
	140	5	5	6	7	8	6	7	8	9	11	9	10	12	13	15	13	14	16	18	20
	120	3	3	3	4	4	4	4	5	5	6	5	6	7	8	9	8	9	10	11	12

Total cholteserol (mmol/L)

Haiti

Systolic Blood Pressur (mmHg) by Total cholteserol (mmol/L)

Women — Non−diabetic — Non−smoker

Age	SBP	3	4	5	6	7
70−74	180	30	34	37	41	45
	160	23	26	28	31	35
	140	17	19	21	24	27
	120	13	14	16	18	20
65−69	180	23	25	28	31	34
	160	16	18	20	23	25
	140	12	13	15	16	18
	120	8	9	10	12	13
60−64	180	17	19	22	24	27
	160	12	13	15	17	19
	140	8	9	10	12	13
	120	5	6	7	8	9
55−59	180	15	16	18	20	23
	160	10	11	12	14	15
	140	6	7	8	9	10
	120	4	5	5	6	7
50−54	180	14	15	17	19	21
	160	9	10	11	12	14
	140	5	6	7	8	9
	120	3	4	4	5	6
45−49	180	13	15	16	18	20
	160	8	9	10	11	13
	140	5	5	6	7	8
	120	3	3	4	4	5
40−44	180	12	14	15	17	19
	160	7	8	9	10	12
	140	4	5	5	6	7
	120	2	3	3	3	4

Women — Non−diabetic — Smoker

Age	SBP	3	4	5	6	7
70−74	180	44	48	52	57	61
	160	34	38	42	46	50
	140	26	29	32	36	39
	120	20	22	25	27	30
65−69	180	34	37	41	45	49
	160	25	28	31	34	37
	140	18	20	23	25	28
	120	13	15	16	18	20
60−64	180	27	30	33	36	40
	160	19	21	23	26	29
	140	13	15	16	18	20
	120	9	10	11	13	14
55−59	180	23	25	28	31	34
	160	15	17	19	21	24
	140	10	11	13	14	16
	120	7	7	8	10	11
50−54	180	21	24	26	29	32
	160	14	16	17	19	22
	140	9	10	11	13	14
	120	6	6	7	8	9
45−49	180	20	23	25	28	31
	160	13	14	16	18	20
	140	8	9	10	11	13
	120	5	5	6	7	8
40−44	180	19	21	24	26	29
	160	12	13	15	16	18
	140	7	8	9	10	11
	120	4	4	5	6	7

Women — Diabetic — Non−smoker

Age	SBP	3	4	5	6	7
70−74	180	55	59	63	68	72
	160	44	48	52	56	60
	140	34	37	41	45	49
	120	26	29	32	35	39
65−69	180	43	47	51	55	60
	160	32	36	39	43	47
	140	24	27	29	33	36
	120	17	19	22	24	27
60−64	180	34	38	41	45	49
	160	25	27	30	33	37
	140	17	19	22	24	27
	120	12	13	15	17	19
55−59	180	29	32	36	39	43
	160	20	22	25	28	31
	140	14	15	17	19	21
	120	9	10	11	13	14
50−54	180	28	30	34	37	41
	160	18	20	23	25	28
	140	12	13	15	17	19
	120	8	8	10	11	12
45−49	180	26	29	32	36	39
	160	17	19	21	24	26
	140	11	12	13	15	17
	120	6	7	8	9	11
40−44	180	25	27	30	34	37
	160	15	17	19	21	24
	140	9	10	12	13	15
	120	5	6	7	8	9

Women — Diabetic — Smoker

Age	SBP	3	4	5	6	7
70−74	180	72	76	80	84	87
	160	60	65	69	73	77
	140	49	53	57	62	66
	120	38	42	46	50	54
65−69	180	59	64	68	72	77
	160	47	51	55	60	64
	140	36	39	43	47	51
	120	27	29	33	36	39
60−64	180	49	53	58	62	66
	160	37	40	44	48	52
	140	27	29	33	36	39
	120	19	21	23	26	29
55−59	180	43	47	51	55	60
	160	31	34	37	41	45
	140	21	24	26	29	32
	120	14	16	18	20	22
50−54	180	41	44	48	53	57
	160	28	31	34	38	41
	140	19	21	23	26	29
	120	12	14	15	17	19
45−49	180	39	43	47	51	55
	160	26	29	32	35	39
	140	17	19	21	23	26
	120	10	12	13	15	17
40−44	180	37	40	44	48	53
	160	24	26	29	32	36
	140	15	16	18	21	23
	120	9	10	11	13	14

Men — Non−diabetic — Non−smoker

Age	SBP	3	4	5	6	7
70−74	180	33	37	40	44	48
	160	25	28	31	34	38
	140	19	21	23	26	29
	120	14	16	18	20	22
65−69	180	27	30	33	36	40
	160	19	22	24	27	30
	140	14	16	18	20	22
	120	10	11	13	14	16
60−64	180	23	26	28	31	35
	160	16	18	20	22	25
	140	11	12	14	16	17
	120	7	8	10	11	12
55−59	180	21	23	26	28	31
	160	14	16	18	20	22
	140	9	10	12	13	15
	120	6	7	8	9	10
50−54	180	18	20	23	25	28
	160	12	13	15	17	19
	140	8	9	10	11	12
	120	5	5	6	7	8
45−49	180	16	18	20	22	24
	160	10	11	12	14	16
	140	6	7	8	9	10
	120	4	4	5	5	6
40−44	180	13	15	16	18	20
	160	8	9	10	11	12
	140	4	5	6	7	7
	120	2	3	3	4	4

Men — Non−diabetic — Smoker

Age	SBP	3	4	5	6	7
70−74	180	43	47	51	55	60
	160	33	37	40	44	48
	140	25	28	31	34	38
	120	19	21	24	26	29
65−69	180	35	39	42	46	51
	160	26	29	32	35	39
	140	19	21	24	26	29
	120	14	15	17	19	21
60−64	180	30	34	37	41	45
	160	22	24	27	30	33
	140	15	17	19	21	24
	120	10	12	13	15	17
55−59	180	28	31	34	37	41
	160	19	21	24	26	29
	140	13	14	16	18	20
	120	8	9	11	12	13
50−54	180	25	27	30	33	37
	160	16	18	20	23	25
	140	10	12	13	15	17
	120	7	7	8	10	11
45−49	180	21	24	26	29	32
	160	13	15	17	19	21
	140	8	9	11	12	13
	120	5	6	6	7	8
40−44	180	18	20	22	24	27
	160	11	12	14	15	17
	140	6	7	8	9	10
	120	4	4	5	5	6

Men — Diabetic — Non−smoker

Age	SBP	3	4	5	6	7
70−74	180	55	59	63	68	72
	160	44	48	52	56	60
	140	34	37	41	45	49
	120	26	29	32	35	38
65−69	180	46	50	54	58	63
	160	35	38	42	46	50
	140	26	29	32	35	38
	120	19	21	23	26	29
60−64	180	40	44	48	52	57
	160	29	32	36	39	43
	140	21	23	26	29	32
	120	14	16	18	20	23
55−59	180	37	40	44	48	52
	160	26	29	32	35	38
	140	18	20	22	24	27
	120	12	13	15	17	19
50−54	180	33	36	40	44	48
	160	22	25	27	30	34
	140	15	16	18	20	23
	120	9	11	12	13	15
45−49	180	29	32	35	39	42
	160	19	21	23	26	28
	140	12	13	15	16	18
	120	7	8	9	10	12
40−44	180	24	27	30	33	36
	160	15	17	19	21	23
	140	9	10	11	13	14
	120	5	6	7	8	9

Men — Diabetic — Smoker

Age	SBP	3	4	5	6	7
70−74	180	67	71	75	79	83
	160	55	59	64	68	72
	140	44	48	52	56	61
	120	34	37	41	45	49
65−69	180	57	62	66	70	75
	160	45	49	53	57	62
	140	34	37	41	45	49
	120	25	28	31	34	38
60−64	180	51	55	60	64	68
	160	38	42	46	50	54
	140	28	31	34	37	41
	120	20	22	24	27	30
55−59	180	47	51	55	60	64
	160	34	37	41	45	49
	140	24	26	29	32	36
	120	16	18	20	22	25
50−54	180	43	47	51	55	59
	160	30	33	36	40	43
	140	20	22	25	27	30
	120	13	14	16	18	20
45−49	180	38	41	45	49	53
	160	25	28	31	34	37
	140	16	18	20	22	25
	120	10	11	13	14	16
40−44	180	32	35	39	42	46
	160	20	23	25	28	31
	140	12	14	16	17	19
	120	7	8	9	11	12

Honduras

Systolic Blood Pressur (mmHg) by Age and Total cholteserol (mmol/L)

Women — Non−diabetic — Non−smoker

Age	Systolic Blood Pressur (mmHg)	3	4	5	6	7
70−74	180	18	20	22	25	28
	160	13	15	17	19	21
	140	10	11	12	14	15
	120	7	8	9	10	11
65−69	180	16	17	20	22	24
	160	11	12	14	16	18
	140	8	9	10	11	13
	120	5	6	7	8	9
60−64	180	13	15	17	19	21
	160	9	10	12	13	15
	140	6	7	8	9	10
	120	4	5	5	6	7
55−59	180	12	14	15	17	19
	160	8	9	10	12	13
	140	5	6	7	8	9
	120	3	4	4	5	6
50−54	180	13	14	16	18	20
	160	8	9	10	12	13
	140	5	6	6	7	8
	120	3	3	4	5	5
45−49	180	13	15	16	18	21
	160	8	9	10	12	13
	140	5	5	6	7	8
	120	3	3	4	4	5
40−44	180	12	14	15	17	19
	160	7	8	9	10	12
	140	4	5	5	6	7
	120	2	3	3	4	4

Women — Non−diabetic — Smoker

Age	Systolic Blood Pressur (mmHg)	3	4	5	6	7
70−74	180	28	31	34	37	41
	160	21	23	26	28	32
	140	15	17	19	21	24
	120	11	13	14	16	18
65−69	180	24	27	30	33	36
	160	17	19	22	24	27
	140	12	14	16	17	20
	120	9	10	11	12	14
60−64	180	21	23	26	29	32
	160	15	16	18	20	23
	140	10	11	13	14	16
	120	7	8	9	10	11
55−59	180	19	21	24	27	29
	160	13	14	16	18	20
	140	8	10	11	12	14
	120	5	6	7	8	9
50−54	180	20	22	25	28	30
	160	13	15	16	18	20
	140	8	9	10	12	13
	120	5	6	7	7	8
45−49	180	20	23	25	28	31
	160	13	14	16	18	20
	140	8	9	10	11	13
	120	5	5	6	7	8
40−44	180	19	21	24	26	29
	160	12	13	15	17	18
	140	7	8	9	10	11
	120	4	5	5	6	7

Women — Diabetic — Non−smoker

Age	Systolic Blood Pressur (mmHg)	3	4	5	6	7
70−74	180	35	39	43	47	51
	160	27	30	33	36	40
	140	20	23	25	28	31
	120	15	17	19	21	23
65−69	180	31	34	38	42	45
	160	23	25	28	31	34
	140	16	18	21	23	26
	120	12	13	15	17	19
60−64	180	27	30	33	37	40
	160	19	21	24	27	29
	140	13	15	17	19	21
	120	9	10	12	13	15
55−59	180	25	28	31	34	38
	160	17	19	21	24	26
	140	11	13	14	16	18
	120	7	8	9	11	12
50−54	180	26	29	32	35	39
	160	17	19	21	24	27
	140	11	12	14	16	18
	120	7	8	9	10	11
45−49	180	27	29	33	36	39
	160	17	19	21	24	26
	140	11	12	13	15	17
	120	6	7	8	9	11
40−44	180	25	28	31	34	37
	160	16	17	19	22	24
	140	9	11	12	13	15
	120	5	6	7	8	9

Women — Diabetic — Smoker

Age	Systolic Blood Pressur (mmHg)	3	4	5	6	7
70−74	180	50	55	59	64	68
	160	40	44	48	52	56
	140	31	34	37	41	45
	120	23	26	29	32	35
65−69	180	45	49	54	58	62
	160	34	38	41	45	49
	140	25	28	31	34	38
	120	18	21	23	25	28
60−64	180	40	44	48	52	56
	160	29	32	36	39	43
	140	21	23	26	28	32
	120	14	16	18	20	23
55−59	180	37	41	45	49	53
	160	26	29	32	35	39
	140	18	20	22	25	28
	120	12	13	15	17	19
50−54	180	39	42	46	50	55
	160	26	29	32	36	39
	140	17	20	22	24	27
	120	11	13	14	16	18
45−49	180	39	43	47	51	55
	160	26	29	32	35	39
	140	17	19	21	23	26
	120	10	12	13	15	17
40−44	180	37	41	45	49	53
	160	24	27	30	33	36
	140	15	17	19	21	23
	120	9	10	11	13	14

Men — Non−diabetic — Non−smoker

Age	Systolic Blood Pressur (mmHg)	3	4	5	6	7
70−74	180	20	22	25	28	31
	160	15	17	19	21	23
	140	11	12	14	15	17
	120	8	9	10	11	13
65−69	180	19	21	24	26	29
	160	14	15	17	19	21
	140	10	11	12	14	15
	120	7	8	9	10	11
60−64	180	19	21	23	26	29
	160	13	14	16	18	20
	140	9	10	11	12	14
	120	6	7	8	8	10
55−59	180	17	19	22	24	27
	160	12	13	15	16	18
	140	8	9	10	11	12
	120	5	5	6	7	8
50−54	180	16	18	20	22	25
	160	10	12	13	15	16
	140	7	7	8	9	11
	120	4	5	5	6	7
45−49	180	13	15	17	18	21
	160	8	9	10	12	13
	140	5	6	6	7	8
	120	3	3	4	4	5
40−44	180	10	11	12	14	15
	160	6	6	7	8	9
	140	3	4	4	5	5
	120	2	2	2	3	3

Men — Non−diabetic — Smoker

Age	Systolic Blood Pressur (mmHg)	3	4	5	6	7
70−74	180	27	30	33	36	40
	160	20	23	25	28	31
	140	15	17	19	21	23
	120	11	12	14	16	17
65−69	180	26	28	31	35	38
	160	19	21	23	26	28
	140	13	15	17	19	21
	120	9	11	12	13	15
60−64	180	25	28	31	34	37
	160	18	20	22	24	27
	140	12	14	15	17	19
	120	8	9	10	12	13
55−59	180	23	26	29	32	35
	160	16	18	20	22	24
	140	10	12	13	15	17
	120	7	8	9	10	11
50−54	180	22	24	27	30	33
	160	14	16	18	20	22
	140	9	10	12	13	15
	120	6	6	7	8	9
45−49	180	18	20	22	25	28
	160	11	13	14	16	18
	140	7	8	9	10	11
	120	4	5	5	6	7
40−44	180	13	15	17	19	21
	160	8	9	10	11	13
	140	5	5	6	7	8
	120	3	3	3	4	4

Men — Diabetic — Non−smoker

Age	Systolic Blood Pressur (mmHg)	3	4	5	6	7
70−74	180	36	39	43	47	51
	160	27	30	34	37	41
	140	21	23	25	28	31
	120	15	17	19	21	24
65−69	180	34	38	41	45	49
	160	25	28	31	34	38
	140	18	20	23	25	28
	120	13	15	16	18	20
60−64	180	33	37	41	44	48
	160	24	27	30	33	36
	140	17	19	21	23	26
	120	12	13	15	16	18
55−59	180	31	35	38	42	46
	160	22	24	27	30	33
	140	15	16	18	20	23
	120	10	11	12	14	15
50−54	180	29	33	36	39	43
	160	20	22	24	27	30
	140	13	14	16	18	20
	120	8	9	10	12	13
45−49	180	24	27	30	33	37
	160	16	17	20	22	24
	140	10	11	12	14	16
	120	6	7	8	9	10
40−44	180	18	21	23	25	28
	160	11	13	14	16	18
	140	7	8	8	10	11
	120	4	4	5	6	6

Men — Diabetic — Smoker

Age	Systolic Blood Pressur (mmHg)	3	4	5	6	7
70−74	180	46	50	54	59	63
	160	36	40	43	47	51
	140	27	30	34	37	41
	120	21	23	26	28	31
65−69	180	44	48	52	57	61
	160	33	37	40	44	48
	140	25	27	30	33	37
	120	18	20	22	25	27
60−64	180	43	47	51	56	60
	160	32	35	39	42	46
	140	23	25	28	31	34
	120	16	18	20	22	24
55−59	180	41	45	49	53	57
	160	29	32	35	39	42
	140	20	22	25	27	30
	120	13	15	17	19	21
50−54	180	38	42	46	50	54
	160	26	29	32	36	39
	140	17	19	22	24	27
	120	11	13	14	16	18
45−49	180	32	36	39	43	47
	160	21	24	26	29	32
	140	13	15	17	19	21
	120	8	9	11	12	13
40−44	180	25	27	30	34	37
	160	15	17	19	21	24
	140	9	10	12	13	15
	120	5	6	7	8	9

Total cholteserol (mmol/L)

Jamaica

Women

Age	Systolic Blood Pressur (mmHg)	Non−diabetic Non−smoker					Non−diabetic Smoker					Diabetic Non−smoker					Diabetic Smoker				
	Total cholteserol (mmol/L)	3	4	5	6	7	3	4	5	6	7	3	4	5	6	7	3	4	5	6	7
70−74	180	18	20	23	25	28	28	31	34	37	41	36	39	43	47	51	51	55	59	64	68
	160	13	15	17	19	21	21	23	26	29	32	27	30	33	37	40	40	44	48	52	57
	140	10	11	12	14	16	16	17	19	22	24	21	23	25	28	31	31	34	38	41	45
	120	7	8	9	10	12	11	13	14	16	18	15	17	19	21	24	24	26	29	32	35
65−69	180	13	14	16	18	20	20	22	25	27	30	26	29	32	35	39	38	42	46	50	54
	160	9	10	11	13	14	14	16	18	20	22	19	21	23	26	29	29	32	35	39	42
	140	6	7	8	9	10	10	11	13	14	16	14	15	17	19	21	21	23	26	29	32
	120	4	5	6	6	7	7	8	9	10	11	10	11	12	14	15	15	17	19	21	24
60−64	180	9	10	12	13	15	15	16	18	20	23	19	22	24	27	29	29	32	36	39	43
	160	6	7	8	9	10	10	11	13	14	16	13	15	17	19	21	21	23	26	29	32
	140	4	5	5	6	7	7	8	9	10	11	9	10	12	13	15	15	16	18	20	23
	120	3	3	3	4	5	4	5	6	7	7	6	7	8	9	10	10	11	13	14	16
55−59	180	7	8	9	10	11	11	12	14	16	17	15	16	18	21	23	23	25	28	31	34
	160	4	5	6	6	7	7	8	9	10	12	10	11	12	14	16	15	17	19	22	24
	140	3	3	4	4	5	5	5	6	7	8	6	7	8	9	10	10	12	13	15	16
	120	2	2	2	3	3	3	3	4	4	5	4	5	5	6	7	7	8	9	10	11
50−54	180	5	6	7	7	8	8	9	11	12	13	11	13	14	16	18	18	20	22	24	27
	160	3	4	4	5	5	5	6	7	8	9	7	8	9	10	11	11	13	14	16	18
	140	2	2	2	3	3	3	4	4	5	5	4	5	6	6	7	7	8	9	10	12
	120	1	1	1	2	2	2	2	2	3	3	3	3	3	4	5	4	5	6	7	7
45−49	180	4	5	6	6	7	7	8	9	10	12	10	11	12	14	16	15	17	19	22	24
	160	3	3	3	4	4	4	5	6	6	7	6	7	8	9	10	10	11	12	14	15
	140	1	2	2	2	2	2	3	3	4	4	3	4	5	5	6	6	7	7	8	10
	120	1	1	1	1	1	1	2	2	2	2	2	2	3	3	3	3	4	4	5	6
40−44	180	4	5	5	6	7	7	8	9	10	11	9	10	12	13	15	14	16	18	20	23
	160	2	3	3	3	4	4	4	5	6	6	5	6	7	8	9	9	10	11	12	14
	140	1	1	2	2	2	2	2	3	3	4	3	3	4	4	5	5	6	7	7	8
	120	0	1	1	1	1	1	1	1	2	2	2	2	2	2	3	3	3	4	4	5

Men

Age	Systolic Blood Pressur (mmHg)	Non−diabetic Non−smoker					Non−diabetic Smoker					Diabetic Non−smoker					Diabetic Smoker				
	Total cholteserol (mmol/L)	3	4	5	6	7	3	4	5	6	7	3	4	5	6	7	3	4	5	6	7
70−74	180	23	25	28	31	34	30	33	37	40	44	40	43	47	52	56	50	55	59	63	68
	160	17	19	21	23	26	23	25	28	31	34	31	34	37	41	45	40	44	48	52	56
	140	12	14	16	18	20	17	19	21	24	26	23	26	29	32	35	31	34	37	41	45
	120	9	10	11	13	14	12	14	16	18	20	17	19	22	24	27	23	26	29	32	35
65−69	180	17	19	21	23	26	23	25	28	31	34	30	34	37	41	45	40	43	47	52	56
	160	12	13	15	17	19	16	18	20	23	25	22	25	28	31	34	30	33	36	40	44
	140	8	9	11	12	13	12	13	15	16	18	16	18	20	22	25	22	24	27	30	33
	120	6	7	7	8	10	8	9	10	12	13	11	13	14	16	18	16	18	20	22	24
60−64	180	13	15	17	19	21	18	20	22	25	28	24	27	30	33	37	32	36	39	43	47
	160	9	10	11	13	14	12	14	16	17	20	17	19	21	24	27	23	26	29	32	35
	140	6	7	8	9	10	8	10	11	12	14	12	13	15	17	19	16	18	20	23	25
	120	4	5	5	6	7	6	6	7	8	9	8	9	10	12	13	11	13	14	16	18
55−59	180	11	13	14	16	18	15	17	19	22	24	21	24	26	29	32	28	31	34	38	42
	160	7	8	9	11	12	10	12	13	15	16	14	16	18	20	22	19	22	24	27	30
	140	5	5	6	7	8	7	8	9	10	11	9	11	12	13	15	13	15	16	18	20
	120	3	3	4	4	5	4	5	6	6	7	6	7	8	9	10	9	10	11	12	14
50−54	180	10	11	12	14	15	13	15	17	18	21	18	20	23	25	28	24	27	30	33	37
	160	6	7	8	9	10	8	9	11	12	13	12	13	15	17	19	16	18	20	22	25
	140	4	4	5	5	6	5	6	7	8	9	7	8	10	11	12	10	12	13	15	17
	120	2	3	3	3	4	3	4	4	5	5	5	5	6	7	8	7	7	8	9	11
45−49	180	8	9	10	12	13	11	13	14	16	18	16	17	19	22	24	21	23	26	29	32
	160	5	6	6	7	8	7	8	9	10	11	10	11	12	14	15	13	15	17	19	21
	140	3	3	4	4	5	4	5	5	6	7	6	7	8	9	10	8	9	10	12	13
	120	2	2	2	2	3	2	3	3	4	4	3	4	5	5	6	5	6	6	7	8
40−44	180	6	7	8	9	10	9	10	11	13	14	12	14	16	17	20	17	19	21	24	26
	160	4	4	5	5	6	5	6	7	8	9	7	8	9	11	12	10	12	13	15	16
	140	2	2	3	3	3	3	3	4	4	5	4	5	6	6	7	6	7	8	9	10
	120	1	1	1	2	2	2	2	2	2	3	2	3	3	4	4	3	4	4	5	6

Total cholteserol (mmol/L)

Mexico

Systolic Blood Pressur (mmHg) by Total cholteserol (mmol/L)

Women — Non−diabetic — Non−smoker

Age	SBP	3	4	5	6	7
70−74	180	20	22	25	27	30
	160	15	16	18	20	23
	140	11	12	14	15	17
	120	8	9	10	11	13
65−69	180	15	17	19	21	23
	160	11	12	13	15	17
	140	7	8	9	11	12
	120	5	6	7	7	8
60−64	180	12	13	15	17	19
	160	8	9	10	11	13
	140	5	6	7	8	9
	120	4	4	5	5	6
55−59	180	9	10	11	13	14
	160	6	7	7	8	10
	140	4	4	5	5	6
	120	2	3	3	3	4
50−54	180	8	9	10	11	12
	160	5	5	6	7	8
	140	3	3	4	4	5
	120	2	2	2	3	3
45−49	180	6	7	7	8	10
	160	3	4	4	5	6
	140	2	2	3	3	3
	120	1	1	1	2	2
40−44	180	4	5	6	7	7
	160	3	3	3	4	4
	140	1	2	2	2	2
	120	1	1	1	1	1

Women — Non−diabetic — Smoker

Age	SBP	3	4	5	6	7
70−74	180	30	33	37	40	44
	160	23	25	28	31	34
	140	17	19	21	23	26
	120	12	14	16	18	20
65−69	180	23	26	29	32	35
	160	17	19	21	23	26
	140	12	13	15	17	19
	120	8	9	11	12	13
60−64	180	18	21	23	26	28
	160	13	14	16	18	20
	140	9	10	11	12	14
	120	6	7	8	8	10
55−59	180	14	16	18	20	22
	160	9	11	12	13	15
	140	6	7	8	9	10
	120	4	4	5	6	7
50−54	180	12	14	15	17	19
	160	8	9	10	11	12
	140	5	5	6	7	8
	120	3	3	4	4	5
45−49	180	9	11	12	13	15
	160	6	7	7	8	9
	140	3	4	4	5	6
	120	2	2	3	3	3
40−44	180	7	8	9	11	12
	160	4	5	6	6	7
	140	2	3	3	4	4
	120	1	1	2	2	2

Women — Diabetic — Non−smoker

Age	SBP	3	4	5	6	7
70−74	180	38	42	46	50	54
	160	29	32	36	39	43
	140	22	25	27	30	34
	120	17	18	21	23	26
65−69	180	30	33	36	40	44
	160	22	24	27	30	33
	140	16	18	20	22	25
	120	11	13	14	16	18
60−64	180	24	27	30	33	36
	160	17	19	21	24	26
	140	12	13	15	17	18
	120	8	9	10	11	13
55−59	180	19	21	23	26	29
	160	13	14	16	18	20
	140	8	9	11	12	13
	120	5	6	7	8	9
50−54	180	16	18	20	22	25
	160	10	12	13	15	16
	140	7	7	8	9	11
	120	4	5	5	6	7
45−49	180	13	14	16	18	20
	160	8	9	10	11	13
	140	5	5	6	7	8
	120	3	3	4	4	5
40−44	180	10	11	13	14	16
	160	6	7	8	9	10
	140	3	4	4	5	6
	120	2	2	2	3	3

Women — Diabetic — Smoker

Age	SBP	3	4	5	6	7
70−74	180	54	58	63	67	71
	160	43	47	51	55	60
	140	33	37	40	44	48
	120	25	28	31	34	38
65−69	180	44	48	52	56	61
	160	33	36	40	44	48
	140	24	27	30	33	37
	120	18	20	22	25	27
60−64	180	36	40	43	47	52
	160	26	29	32	35	39
	140	18	21	23	25	28
	120	13	14	16	18	20
55−59	180	29	32	35	39	42
	160	20	22	25	27	30
	140	13	15	17	19	21
	120	9	10	11	13	14
50−54	180	25	28	30	34	37
	160	16	18	20	23	25
	140	11	12	13	15	17
	120	7	8	9	10	11
45−49	180	20	22	25	27	30
	160	13	14	16	18	20
	140	8	9	10	11	12
	120	5	5	6	7	8
40−44	180	16	18	20	22	25
	160	10	11	12	14	15
	140	6	6	7	8	9
	120	3	4	4	5	5

Men — Non−diabetic — Non−smoker

Age	SBP	3	4	5	6	7
70−74	180	23	26	28	32	35
	160	17	19	21	24	27
	140	13	14	16	18	20
	120	9	10	12	13	15
65−69	180	19	21	23	26	29
	160	13	15	17	19	21
	140	9	11	12	13	15
	120	7	7	8	10	11
60−64	180	16	18	20	22	24
	160	11	12	14	15	17
	140	7	8	9	10	12
	120	5	5	6	7	8
55−59	180	14	16	17	19	22
	160	9	10	12	13	15
	140	6	7	8	9	10
	120	4	4	5	6	6
50−54	180	13	14	16	18	20
	160	8	9	10	12	13
	140	5	6	7	7	8
	120	3	4	4	5	5
45−49	180	11	13	14	16	18
	160	7	8	9	10	11
	140	4	5	5	6	7
	120	2	3	3	4	4
40−44	180	9	10	11	13	14
	160	5	6	7	7	8
	140	3	3	4	4	5
	120	2	2	2	2	3

Men — Non−diabetic — Smoker

Age	SBP	3	4	5	6	7
70−74	180	31	34	37	41	45
	160	23	26	29	32	35
	140	17	19	22	24	27
	120	13	14	16	18	20
65−69	180	25	28	31	34	38
	160	18	20	23	25	28
	140	13	15	16	18	20
	120	9	10	12	13	15
60−64	180	21	24	26	29	32
	160	15	17	18	21	23
	140	10	11	13	14	16
	120	7	8	9	10	11
55−59	180	19	21	23	26	29
	160	13	14	16	18	20
	140	8	9	11	12	13
	120	5	6	7	8	9
50−54	180	18	20	22	24	27
	160	11	13	14	16	18
	140	7	8	9	10	12
	120	4	5	6	6	7
45−49	180	16	17	19	22	24
	160	10	11	12	14	15
	140	6	7	8	9	10
	120	3	4	5	5	6
40−44	180	12	14	15	17	19
	160	7	8	9	10	12
	140	4	5	5	6	7
	120	2	3	3	3	4

Men — Diabetic — Non−smoker

Age	SBP	3	4	5	6	7
70−74	180	40	44	48	52	57
	160	31	34	38	42	45
	140	24	26	29	32	35
	120	18	20	22	24	27
65−69	180	34	37	41	45	49
	160	25	28	31	34	37
	140	18	20	22	25	28
	120	13	14	16	18	20
60−64	180	29	32	35	38	42
	160	20	23	25	28	31
	140	14	16	18	20	22
	120	10	11	12	14	15
55−59	180	26	28	31	35	38
	160	17	19	22	24	27
	140	12	13	15	16	18
	120	8	9	10	11	12
50−54	180	24	27	29	33	36
	160	16	18	20	22	24
	140	10	11	13	14	16
	120	6	7	8	9	10
45−49	180	21	24	26	29	32
	160	14	15	17	19	21
	140	8	9	11	12	13
	120	5	6	6	7	8
40−44	180	17	19	21	23	26
	160	10	11	13	14	16
	140	6	7	8	9	10
	120	3	4	4	5	6

Men — Diabetic — Smoker

Age	SBP	3	4	5	6	7
70−74	180	51	56	60	64	69
	160	40	44	48	53	57
	140	31	35	38	42	46
	120	24	26	29	32	36
65−69	180	43	47	52	56	60
	160	33	36	40	44	48
	140	24	27	30	33	36
	120	18	20	22	24	27
60−64	180	37	41	45	49	53
	160	27	30	33	37	40
	140	19	21	24	26	29
	120	13	15	17	19	21
55−59	180	34	37	41	45	49
	160	23	26	29	32	35
	140	16	18	20	22	25
	120	11	12	13	15	17
50−54	180	32	35	38	42	46
	160	21	24	26	29	32
	140	14	16	17	20	22
	120	9	10	11	13	14
45−49	180	28	32	35	38	42
	160	18	21	23	26	28
	140	12	13	15	16	18
	120	7	8	9	10	12
40−44	180	23	25	28	31	34
	160	14	16	18	20	22
	140	8	9	11	12	13
	120	5	6	6	7	8

Nicaragua

Systolic Blood Pressur (mmHg) by Total cholteserol (mmol/L)

Women

Age	SBP	Non−diabetic Non−smoker 3	4	5	6	7	Non−diabetic Smoker 3	4	5	6	7	Diabetic Non−smoker 3	4	5	6	7	Diabetic Smoker 3	4	5	6	7
70−74	180	23	26	29	32	35	35	38	42	46	50	44	48	52	56	61	60	65	69	74	78
	160	17	19	22	24	27	27	29	33	36	39	34	38	41	45	49	49	53	57	62	66
	140	13	14	16	18	20	20	22	25	27	30	26	29	32	35	39	38	42	46	50	55
	120	9	10	12	13	15	15	17	18	21	23	19	22	24	27	30	30	33	36	40	43
65−69	180	18	21	23	26	28	28	31	34	38	42	36	40	43	47	52	51	56	60	64	69
	160	13	15	17	19	21	21	23	25	28	31	27	30	33	36	40	40	43	47	52	56
	140	9	10	12	13	15	15	17	18	21	23	19	22	24	27	30	30	33	36	40	43
	120	6	7	8	9	11	10	12	13	15	17	14	16	18	20	22	22	24	27	30	33
60−64	180	16	18	21	23	25	25	28	31	34	38	33	36	40	43	47	47	51	56	60	64
	160	11	13	14	16	18	18	20	22	25	27	23	26	29	32	35	35	38	42	46	50
	140	8	9	10	11	12	12	14	15	17	19	16	18	20	23	25	25	28	31	34	37
	120	5	6	7	7	8	8	9	11	12	13	11	13	14	16	18	18	20	22	24	27
55−59	180	14	15	17	19	21	21	24	26	29	32	28	31	34	37	41	41	45	49	53	57
	160	9	10	11	13	14	14	16	18	20	22	19	21	24	26	29	29	32	35	39	43
	140	6	7	8	8	10	10	11	12	14	15	13	14	16	18	20	20	22	25	27	30
	120	4	4	5	5	6	6	7	8	9	10	8	9	11	12	14	13	15	17	19	21
50−54	180	11	12	14	15	17	17	19	21	24	27	23	25	28	31	34	34	37	41	45	49
	160	7	8	9	10	11	11	12	14	16	18	15	17	19	21	23	23	25	28	31	35
	140	4	5	6	6	7	7	8	9	10	11	10	11	12	14	15	15	17	19	21	23
	120	3	3	3	4	4	4	5	6	6	7	6	7	8	9	10	10	11	12	14	15
45−49	180	9	10	12	13	15	15	16	18	21	23	19	22	24	27	30	29	33	36	39	43
	160	6	6	7	8	9	9	10	12	13	15	12	14	15	17	19	19	21	24	26	29
	140	3	4	4	5	6	5	6	7	8	9	7	8	10	11	12	12	13	15	17	19
	120	2	2	2	3	3	3	4	4	5	5	4	5	6	7	7	7	8	9	11	12
40−44	180	8	9	11	12	13	13	15	17	19	21	18	20	22	25	27	27	30	33	37	40
	160	5	6	6	7	8	8	9	10	11	13	11	12	14	15	17	17	19	21	24	26
	140	3	3	4	4	5	5	5	6	7	8	6	7	8	9	10	10	12	13	15	16
	120	1	2	2	2	3	3	3	3	4	4	4	4	5	5	6	6	7	8	9	10

Men

Age	SBP	Non−diabetic Non−smoker 3	4	5	6	7	Non−diabetic Smoker 3	4	5	6	7	Diabetic Non−smoker 3	4	5	6	7	Diabetic Smoker 3	4	5	6	7
70−74	180	26	29	32	35	39	34	38	41	45	49	45	49	53	57	62	56	60	65	69	74
	160	19	22	24	27	30	26	29	32	35	39	35	38	42	46	50	45	49	53	57	62
	140	14	16	18	20	23	20	22	24	27	30	27	29	33	36	39	35	38	42	46	50
	120	11	12	13	15	17	14	16	18	20	23	20	22	25	27	30	27	30	33	36	40
65−69	180	23	25	28	31	34	30	33	36	40	44	40	43	47	51	56	50	55	59	63	68
	160	16	18	20	23	25	22	24	27	30	33	30	33	36	40	43	39	42	46	50	55
	140	12	13	15	16	18	16	18	20	22	24	22	24	27	30	33	29	32	35	39	42
	120	8	9	10	12	13	11	13	14	16	18	16	17	19	22	24	21	23	26	29	32
60−64	180	21	23	26	29	32	28	31	34	37	41	37	40	44	48	52	47	51	56	60	64
	160	14	16	18	20	22	20	22	24	27	30	27	29	33	36	39	35	38	42	46	50
	140	10	11	12	14	16	14	15	17	19	21	19	21	23	26	29	25	28	31	34	37
	120	7	7	8	10	11	9	10	12	13	15	13	14	16	18	20	18	20	22	24	27
55−59	180	18	20	22	25	27	24	27	29	33	36	32	35	39	43	47	42	46	50	54	58
	160	12	13	15	17	19	16	18	20	23	25	22	25	28	30	34	30	33	36	40	43
	140	8	9	10	11	13	11	12	14	15	17	15	17	19	21	23	20	23	25	28	31
	120	5	6	6	7	8	7	8	9	10	11	10	11	13	14	16	14	15	17	19	22
50−54	180	14	16	18	20	23	20	22	24	27	30	27	29	33	36	39	35	38	42	46	50
	160	9	10	12	13	15	13	14	16	18	20	18	20	22	24	27	24	26	29	32	36
	140	6	7	7	8	9	8	9	10	12	13	11	13	14	16	18	16	18	20	22	24
	120	4	4	5	5	6	5	6	7	7	8	7	8	9	10	12	10	11	13	14	16
45−49	180	11	13	15	16	18	16	18	20	22	24	22	24	27	30	33	29	32	35	39	42
	160	7	8	9	10	11	10	11	12	14	16	14	15	17	19	21	19	21	23	26	29
	140	4	5	5	6	7	6	7	8	9	10	8	10	11	12	14	12	13	15	16	18
	120	2	3	3	4	4	3	4	5	5	6	5	6	7	7	8	7	8	9	10	12
40−44	180	10	11	12	14	15	13	15	17	19	21	18	20	23	25	28	25	27	30	33	37
	160	6	6	7	8	9	8	9	10	11	13	11	12	14	16	18	15	17	19	21	24
	140	3	4	4	5	5	5	5	6	7	8	7	7	8	9	11	9	10	12	13	15
	120	2	2	2	3	3	3	3	3	4	4	4	4	5	6	6	5	6	7	8	9

Panama

Women

Age	Systolic Blood Pressur (mmHg)	Non−diabetic Non−smoker					Non−diabetic Smoker					Diabetic Non−smoker					Diabetic Smoker				
	Total cholteserol (mmol/L)	3	4	5	6	7	3	4	5	6	7	3	4	5	6	7	3	4	5	6	7
70−74	180	14	16	18	20	22	22	24	27	30	33	28	31	35	38	42	42	46	50	54	58
	160	10	12	13	15	16	16	18	20	23	25	21	24	26	29	32	32	36	39	43	47
	140	7	8	9	11	12	12	13	15	17	19	16	18	20	22	25	24	27	30	33	37
	120	5	6	7	8	9	9	10	11	12	14	12	13	15	16	18	18	20	23	25	28
65−69	180	11	13	14	16	18	18	20	22	24	27	23	26	29	32	35	35	38	42	46	50
	160	8	9	10	11	13	13	14	16	18	20	17	19	21	23	26	26	28	32	35	38
	140	5	6	7	8	9	9	10	11	13	14	12	13	15	17	19	19	21	23	26	29
	120	4	4	5	5	6	6	7	8	9	10	8	9	11	12	13	13	15	17	19	21
60−64	180	10	11	12	14	16	16	17	20	22	24	21	23	25	28	31	31	34	38	42	45
	160	7	8	8	10	11	11	12	14	15	17	14	16	18	20	22	22	25	27	30	34
	140	4	5	6	6	7	7	8	9	10	12	10	11	12	14	16	15	17	19	22	24
	120	3	3	4	4	5	5	5	6	7	8	7	7	8	9	11	11	12	13	15	17
55−59	180	8	9	10	11	13	12	14	16	17	20	16	18	21	23	26	25	28	31	34	38
	160	5	6	6	7	8	8	9	10	12	13	11	12	14	16	17	17	19	22	24	27
	140	3	4	4	5	5	5	6	7	8	9	7	8	9	10	12	12	13	15	16	18
	120	2	2	3	3	3	3	4	4	5	6	5	5	6	7	8	8	9	10	11	12
50−54	180	7	8	9	11	12	12	13	15	17	19	16	18	20	22	24	24	27	30	33	36
	160	5	5	6	7	8	7	8	10	11	12	10	11	13	14	16	16	18	20	22	25
	140	3	3	4	4	5	5	5	6	7	8	6	7	8	9	10	10	12	13	15	16
	120	2	2	2	2	3	3	3	4	4	5	4	4	5	6	7	6	7	8	9	11
45−49	180	7	8	9	10	11	11	12	14	16	18	15	17	18	21	23	23	25	28	31	34
	160	4	5	5	6	7	7	8	9	10	11	9	10	12	13	15	15	16	18	20	23
	140	2	3	3	4	4	4	5	5	6	7	6	6	7	8	9	9	10	11	13	14
	120	1	1	2	2	2	2	3	3	3	4	3	4	4	5	5	5	6	7	8	9
40−44	180	5	6	7	8	9	8	10	11	12	14	11	13	14	16	18	18	20	22	25	28
	160	3	3	4	4	5	5	6	6	7	8	7	8	9	10	11	11	12	14	15	17
	140	2	2	2	2	3	3	3	4	4	5	4	4	5	6	6	6	7	8	9	10
	120	1	1	1	1	1	1	2	2	2	3	2	2	3	3	4	4	4	5	5	6

Men

Age	Systolic Blood Pressur (mmHg)	Non−diabetic Non−smoker					Non−diabetic Smoker					Diabetic Non−smoker					Diabetic Smoker				
	Total cholteserol (mmol/L)	3	4	5	6	7	3	4	5	6	7	3	4	5	6	7	3	4	5	6	7
70−74	180	20	22	24	27	30	26	29	32	35	39	35	38	42	46	50	45	49	53	58	62
	160	14	16	18	20	23	20	22	24	27	30	27	29	33	36	40	35	39	42	46	50
	140	11	12	13	15	17	14	16	18	20	23	20	22	25	27	30	27	30	33	36	40
	120	8	9	10	11	12	11	12	13	15	17	15	17	18	21	23	20	22	25	28	30
65−69	180	18	20	23	25	28	24	27	30	33	37	33	36	40	43	47	42	46	50	55	59
	160	13	15	16	18	20	18	20	22	24	27	24	27	30	33	36	32	35	39	42	46
	140	9	10	12	13	15	13	14	16	18	20	17	19	22	24	27	23	26	29	32	35
	120	6	7	8	9	10	9	10	11	13	14	12	14	16	17	19	17	19	21	23	26
60−64	180	17	19	21	24	27	23	26	29	32	35	31	34	38	42	45	40	44	48	53	57
	160	12	13	15	17	19	16	18	20	23	25	22	25	27	30	34	29	33	36	40	43
	140	8	9	10	11	13	11	13	14	16	18	15	17	19	22	24	21	23	26	29	32
	120	5	6	7	8	9	8	9	10	11	12	11	12	13	15	17	15	16	18	20	23
55−59	180	17	19	21	23	26	23	25	28	31	34	31	34	37	41	45	40	44	48	52	56
	160	11	13	14	16	18	15	17	19	22	24	21	24	26	29	32	28	31	35	38	42
	140	7	8	9	11	12	10	12	13	15	16	14	16	18	20	22	19	22	24	27	30
	120	5	5	6	7	8	7	8	9	10	11	9	11	12	13	15	13	15	16	18	20
50−54	180	14	16	18	20	23	20	22	24	27	30	27	30	33	36	40	35	39	42	46	50
	160	9	10	12	13	15	13	14	16	18	20	18	20	22	25	27	24	26	29	32	36
	140	6	7	7	8	10	8	9	10	12	13	11	13	14	16	18	16	18	20	22	24
	120	4	4	5	5	6	5	6	7	7	8	7	8	9	10	12	10	11	13	14	16
45−49	180	11	13	14	16	18	16	17	19	22	24	21	24	26	29	32	28	31	35	38	42
	160	7	8	9	10	11	10	11	12	14	15	14	15	17	19	21	18	21	23	25	28
	140	4	5	5	6	7	6	7	8	9	10	8	9	11	12	13	12	13	15	16	18
	120	2	3	3	4	4	3	4	5	5	6	5	6	7	7	8	7	8	9	10	12
40−44	180	8	9	10	11	12	11	12	14	15	17	15	17	19	21	23	20	23	25	28	31
	160	4	5	6	7	7	6	7	8	9	10	9	10	11	13	14	12	14	16	17	20
	140	2	3	3	4	4	4	4	5	5	6	5	6	7	8	9	7	8	9	11	12
	120	1	2	2	2	2	2	2	3	3	3	3	3	4	4	5	4	5	6	6	7

Paraguay

Systolic Blood Pressur (mmHg) by Total cholteserol (mmol/L)

Women

Age 70–74

Systolic Blood Pressur (mmHg)	Non−diabetic Non−smoker 3	4	5	6	7	Non−diabetic Smoker 3	4	5	6	7	Diabetic Non−smoker 3	4	5	6	7	Diabetic Smoker 3	4	5	6	7
180	23	26	29	32	35	35	39	42	46	50	44	48	52	57	61	61	65	70	74	78
160	18	20	22	24	27	27	30	33	36	40	34	38	42	46	50	49	54	58	62	67
140	13	15	16	18	20	20	23	25	28	31	26	29	32	36	39	39	43	47	51	55
120	9	11	12	13	15	15	17	19	21	23	20	22	25	27	30	30	33	37	40	44

Age 65–69

Systolic Blood Pressur (mmHg)	Non−diabetic Non−smoker 3	4	5	6	7	Non−diabetic Smoker 3	4	5	6	7	Diabetic Non−smoker 3	4	5	6	7	Diabetic Smoker 3	4	5	6	7
180	17	20	22	24	27	27	30	33	36	40	34	38	42	45	50	49	54	58	62	67
160	12	14	16	18	20	19	22	24	27	30	25	28	31	34	38	38	41	45	49	54
140	9	10	11	13	14	14	16	17	20	22	18	21	23	26	28	28	31	34	38	42
120	6	7	8	9	10	10	11	12	14	16	13	15	17	19	21	21	23	25	28	31

Age 60–64

Systolic Blood Pressur (mmHg)	Non−diabetic Non−smoker 3	4	5	6	7	Non−diabetic Smoker 3	4	5	6	7	Diabetic Non−smoker 3	4	5	6	7	Diabetic Smoker 3	4	5	6	7
180	16	18	20	23	25	25	28	31	34	37	32	36	39	43	47	47	51	55	59	64
160	11	13	14	16	18	18	20	22	24	27	23	26	28	31	35	35	38	42	46	50
140	8	9	10	11	12	12	14	15	17	19	16	18	20	22	25	25	28	31	34	37
120	5	6	6	7	8	8	9	10	12	13	11	12	14	16	18	17	19	22	24	27

Age 55–59

Systolic Blood Pressur (mmHg)	Non−diabetic Non−smoker 3	4	5	6	7	Non−diabetic Smoker 3	4	5	6	7	Diabetic Non−smoker 3	4	5	6	7	Diabetic Smoker 3	4	5	6	7
180	16	18	20	22	24	24	27	30	33	36	31	35	38	42	46	46	50	54	58	63
160	10	12	13	15	17	17	18	21	23	26	22	24	27	30	33	33	36	40	43	47
140	7	8	9	10	11	11	12	14	16	17	15	16	18	21	23	23	25	28	31	34
120	4	5	6	6	7	7	8	9	10	12	10	11	12	14	15	15	17	19	21	24

Age 50–54

Systolic Blood Pressur (mmHg)	Non−diabetic Non−smoker 3	4	5	6	7	Non−diabetic Smoker 3	4	5	6	7	Diabetic Non−smoker 3	4	5	6	7	Diabetic Smoker 3	4	5	6	7
180	15	17	19	21	24	23	26	29	32	35	30	33	37	40	44	44	48	52	57	61
160	10	11	12	14	15	15	17	19	21	24	20	23	25	28	31	31	34	37	41	45
140	6	7	8	9	10	10	11	12	14	16	13	15	17	19	21	21	23	25	28	31
120	4	4	5	6	6	6	7	8	9	10	8	9	11	12	14	13	15	17	19	21

Age 45–49

Systolic Blood Pressur (mmHg)	Non−diabetic Non−smoker 3	4	5	6	7	Non−diabetic Smoker 3	4	5	6	7	Diabetic Non−smoker 3	4	5	6	7	Diabetic Smoker 3	4	5	6	7
180	13	15	17	19	21	21	23	26	29	32	27	30	33	37	40	40	44	48	52	56
160	8	9	11	12	13	13	15	17	19	21	18	20	22	24	27	27	30	33	36	40
140	5	6	6	7	8	8	9	10	12	13	11	12	14	16	17	17	19	22	24	27
120	3	3	4	4	5	5	6	6	7	8	7	8	9	10	11	11	12	14	15	17

Age 40–44

Systolic Blood Pressur (mmHg)	Non−diabetic Non−smoker 3	4	5	6	7	Non−diabetic Smoker 3	4	5	6	7	Diabetic Non−smoker 3	4	5	6	7	Diabetic Smoker 3	4	5	6	7
180	11	13	14	16	18	18	20	22	24	27	23	26	28	31	35	34	38	42	46	50
160	7	7	8	10	11	11	12	13	15	17	14	16	18	20	22	22	25	27	30	33
140	4	4	5	6	6	6	7	8	9	10	8	10	11	12	14	14	15	17	19	21
120	2	2	3	3	4	4	4	5	5	6	5	6	6	7	8	8	9	10	12	13

Men

Age 70–74

Systolic Blood Pressur (mmHg)	Non−diabetic Non−smoker 3	4	5	6	7	Non−diabetic Smoker 3	4	5	6	7	Diabetic Non−smoker 3	4	5	6	7	Diabetic Smoker 3	4	5	6	7
180	24	27	30	33	36	32	35	39	42	46	42	46	50	54	58	53	57	62	66	70
160	18	20	22	25	28	24	27	30	33	36	32	36	39	43	47	42	46	50	54	59
140	13	15	17	19	21	18	20	23	25	28	25	27	30	33	37	33	36	39	43	47
120	10	11	12	14	15	13	15	17	19	21	18	21	23	26	28	25	28	30	34	37

Age 65–69

Systolic Blood Pressur (mmHg)	Non−diabetic Non−smoker 3	4	5	6	7	Non−diabetic Smoker 3	4	5	6	7	Diabetic Non−smoker 3	4	5	6	7	Diabetic Smoker 3	4	5	6	7
180	20	23	25	28	31	27	30	33	37	41	36	40	44	48	52	47	51	55	59	64
160	15	16	18	21	23	20	22	25	27	30	27	30	33	36	40	35	39	43	47	51
140	10	12	13	15	16	14	16	18	20	22	20	22	24	27	30	26	29	32	36	39
120	7	8	9	10	12	10	11	13	14	16	14	16	18	20	22	19	21	24	26	29

Age 60–64

Systolic Blood Pressur (mmHg)	Non−diabetic Non−smoker 3	4	5	6	7	Non−diabetic Smoker 3	4	5	6	7	Diabetic Non−smoker 3	4	5	6	7	Diabetic Smoker 3	4	5	6	7
180	19	22	24	27	30	26	29	32	35	39	35	38	42	46	50	45	49	53	57	62
160	13	15	17	19	21	18	20	23	25	28	25	28	31	34	37	33	36	40	44	48
140	9	10	12	13	15	13	14	16	18	20	17	19	22	24	27	23	26	29	32	35
120	6	7	8	9	10	9	10	11	12	14	12	13	15	17	19	16	18	20	23	25

Age 55–59

Systolic Blood Pressur (mmHg)	Non−diabetic Non−smoker 3	4	5	6	7	Non−diabetic Smoker 3	4	5	6	7	Diabetic Non−smoker 3	4	5	6	7	Diabetic Smoker 3	4	5	6	7
180	18	20	22	25	28	24	27	30	33	36	32	36	39	43	47	42	46	50	54	59
160	12	13	15	17	19	16	18	20	23	25	22	25	28	31	34	30	33	36	40	44
140	8	9	10	11	13	11	12	14	15	17	15	17	19	21	24	21	23	26	28	31
120	5	6	7	7	8	7	8	9	10	12	10	11	13	14	16	14	16	17	19	22

Age 50–54

Systolic Blood Pressur (mmHg)	Non−diabetic Non−smoker 3	4	5	6	7	Non−diabetic Smoker 3	4	5	6	7	Diabetic Non−smoker 3	4	5	6	7	Diabetic Smoker 3	4	5	6	7
180	16	18	20	22	25	22	24	27	30	33	29	32	36	39	43	38	42	46	50	54
160	10	12	13	15	16	14	16	18	20	22	20	22	24	27	30	26	29	32	35	39
140	7	7	8	9	11	9	10	12	13	15	13	14	16	18	20	17	19	22	24	27
120	4	5	5	6	7	6	6	7	8	9	8	9	10	12	13	11	13	14	16	18

Age 45–49

Systolic Blood Pressur (mmHg)	Non−diabetic Non−smoker 3	4	5	6	7	Non−diabetic Smoker 3	4	5	6	7	Diabetic Non−smoker 3	4	5	6	7	Diabetic Smoker 3	4	5	6	7
180	15	17	19	21	23	20	23	25	28	31	28	31	34	37	41	36	40	44	48	52
160	9	11	12	13	15	13	14	16	18	20	18	20	22	25	27	24	27	29	33	36
140	6	6	7	8	9	8	9	10	11	13	11	13	14	16	18	15	17	19	21	24
120	3	4	4	5	6	5	5	6	7	8	7	8	9	10	11	9	11	12	14	15

Age 40–44

Systolic Blood Pressur (mmHg)	Non−diabetic Non−smoker 3	4	5	6	7	Non−diabetic Smoker 3	4	5	6	7	Diabetic Non−smoker 3	4	5	6	7	Diabetic Smoker 3	4	5	6	7
180	12	14	15	17	19	17	19	21	23	26	23	25	28	31	34	30	33	37	41	44
160	7	8	9	10	12	10	11	13	14	16	14	16	18	20	22	19	21	24	26	29
140	4	5	5	6	7	6	7	8	9	10	8	10	11	12	14	12	13	15	17	18
120	2	3	3	4	4	3	4	4	5	6	5	6	6	7	8	7	8	9	10	11

Total cholteserol (mmol/L)

Peru

Systolic Blood Pressur (mmHg) by Age; Total cholteserol (mmol/L)

Women

Age	SBP	Non−diabetic Non−smoker 3	4	5	6	7	Non−diabetic Smoker 3	4	5	6	7	Diabetic Non−smoker 3	4	5	6	7	Diabetic Smoker 3	4	5	6	7
70−74	180	25	27	30	33	37	37	40	44	48	52	46	50	54	59	63	63	67	72	76	80
	160	18	21	23	25	28	28	31	34	38	42	36	40	43	47	51	51	56	60	64	69
	140	14	15	17	19	21	21	24	26	29	32	28	31	34	37	41	41	44	48	53	57
	120	10	11	13	14	16	16	18	20	22	24	21	23	26	29	32	31	35	38	42	46
65−69	180	18	20	22	25	28	28	31	34	37	41	35	39	43	47	51	50	55	59	64	68
	160	13	14	16	18	20	20	22	25	28	31	26	29	32	36	39	39	43	47	51	55
	140	9	10	12	13	15	14	16	18	20	23	19	21	24	26	29	29	32	35	39	43
	120	6	7	8	9	10	10	12	13	15	16	14	15	17	19	21	21	24	26	29	32
60−64	180	15	17	19	21	23	23	26	28	31	35	30	33	36	40	44	43	47	52	56	60
	160	10	11	13	14	16	16	18	20	22	25	21	24	26	29	32	32	35	39	42	46
	140	7	8	9	10	11	11	12	14	16	17	15	16	18	21	23	23	25	28	31	34
	120	5	5	6	7	8	7	8	10	11	12	10	11	13	14	16	16	18	20	22	25
55−59	180	13	15	17	19	21	21	23	26	29	32	27	30	33	37	40	40	44	48	52	57
	160	9	10	11	13	14	14	16	18	20	22	19	21	23	26	29	28	32	35	38	42
	140	6	7	7	8	9	9	11	12	13	15	13	14	16	18	20	20	22	24	27	30
	120	4	4	5	5	6	6	7	8	9	10	8	9	10	12	13	13	15	17	18	21
50−54	180	13	14	16	18	20	20	22	25	27	30	26	29	32	35	39	39	42	46	50	55
	160	8	9	10	12	13	13	15	16	18	20	17	19	21	24	27	26	29	32	36	39
	140	5	6	7	7	8	8	9	11	12	13	11	12	14	16	18	17	20	22	24	27
	120	3	3	4	5	5	5	6	7	7	8	7	8	9	10	11	11	13	14	16	18
45−49	180	12	13	15	16	18	18	20	23	25	28	24	27	29	32	36	36	39	43	47	51
	160	7	8	9	10	12	11	13	14	16	18	15	17	19	21	24	24	26	29	32	35
	140	4	5	5	6	7	7	8	9	10	11	9	11	12	13	15	15	17	19	21	23
	120	2	3	3	4	4	4	5	5	6	7	6	6	7	8	9	9	10	12	13	15
40−44	180	10	11	12	14	15	15	17	19	21	24	20	22	25	28	31	30	34	37	41	45
	160	6	6	7	8	9	9	10	12	13	15	12	14	15	17	19	19	21	24	27	29
	140	3	4	4	5	5	5	6	7	8	9	7	8	9	11	12	12	13	15	17	19
	120	2	2	2	3	3	3	3	4	5	5	4	5	5	6	7	7	8	9	10	11

Men

Age	SBP	Non−diabetic Non−smoker 3	4	5	6	7	Non−diabetic Smoker 3	4	5	6	7	Diabetic Non−smoker 3	4	5	6	7	Diabetic Smoker 3	4	5	6	7
70−74	180	24	27	30	33	36	32	35	39	43	47	42	46	50	54	59	53	58	62	66	71
	160	18	20	23	25	28	24	27	30	33	37	33	36	40	43	47	42	46	50	55	59
	140	13	15	17	19	21	18	20	23	25	28	25	28	31	34	37	33	36	40	44	48
	120	10	11	12	14	16	13	15	17	19	21	19	21	23	26	29	25	28	31	34	37
65−69	180	21	23	26	28	31	28	31	34	37	41	37	40	44	48	52	47	51	55	60	64
	160	15	17	19	21	23	20	22	25	28	31	27	30	33	37	40	36	39	43	47	51
	140	10	12	13	15	17	14	16	18	20	22	20	22	25	27	30	27	29	33	36	39
	120	7	8	9	11	12	10	11	13	14	16	14	16	18	20	22	19	22	24	27	30
60−64	180	19	21	24	26	29	26	28	31	35	38	34	38	41	45	49	44	48	52	57	61
	160	13	15	17	19	21	18	20	22	25	28	25	27	30	33	37	32	36	39	43	47
	140	9	10	11	13	14	12	14	16	17	20	17	19	21	24	27	23	26	29	32	35
	120	6	7	8	9	10	8	10	11	12	14	12	13	15	17	19	16	18	20	23	25
55−59	180	18	20	22	25	27	24	27	30	33	36	32	36	39	43	47	42	46	50	54	58
	160	12	13	15	17	19	16	18	20	23	25	22	25	28	31	34	30	33	36	40	44
	140	8	9	10	11	13	11	12	14	15	17	15	17	19	21	24	20	23	25	28	31
	120	5	6	6	7	8	7	8	9	10	12	10	11	13	14	16	14	15	17	19	22
50−54	180	16	18	20	23	25	22	25	27	30	33	30	33	36	40	44	39	43	47	51	55
	160	11	12	13	15	17	14	16	18	20	23	20	22	25	27	30	27	29	33	36	40
	140	7	7	8	10	11	9	10	12	13	15	13	15	16	18	20	18	20	22	24	27
	120	4	5	5	6	7	6	7	7	8	9	8	9	10	12	13	11	13	14	16	18
45−49	180	15	17	19	21	23	20	22	25	28	31	27	30	33	37	40	36	39	43	47	51
	160	9	10	12	13	15	13	14	16	18	20	18	20	22	24	27	24	26	29	32	36
	140	6	6	7	8	9	8	9	10	11	13	11	12	14	16	17	15	17	19	21	23
	120	3	4	4	5	6	5	5	6	7	8	7	8	9	10	11	9	11	12	13	15
40−44	180	12	14	16	17	19	17	19	21	23	26	23	26	28	31	35	31	34	37	41	45
	160	7	8	9	11	12	10	11	13	14	16	14	16	18	20	22	19	22	24	27	30
	140	4	5	5	6	7	6	7	8	9	10	9	10	11	12	14	12	13	15	17	19
	120	2	3	3	4	4	3	4	4	5	6	5	6	6	7	8	7	8	9	10	11

Saint Lucia

Systolic Blood Pressur (mmHg) by Total cholteserol (mmol/L)

Women – Non−diabetic – Non−smoker

Age	SBP	3	4	5	6	7
70−74	180	17	19	21	23	26
	160	12	14	16	17	19
	140	9	10	11	13	14
	120	6	7	8	9	11
65−69	180	14	16	18	20	22
	160	10	11	13	14	16
	140	7	8	9	10	11
	120	5	6	6	7	8
60−64	180	13	14	16	18	20
	160	9	10	11	12	14
	140	6	7	7	8	9
	120	4	4	5	6	6
55−59	180	10	12	13	15	17
	160	7	8	9	10	11
	140	4	5	6	6	7
	120	3	3	4	4	5
50−54	180	9	11	12	13	15
	160	6	7	7	8	10
	140	4	4	5	5	6
	120	2	2	3	3	4
45−49	180	9	10	11	13	14
	160	5	6	7	8	9
	140	3	4	4	5	5
	120	2	2	2	3	3
40−44	180	9	10	12	13	15
	160	5	6	7	8	9
	140	3	4	4	5	5
	120	2	2	2	3	3

Women – Non−diabetic – Smoker

Age	SBP	3	4	5	6	7
70−74	180	26	29	32	35	38
	160	19	22	24	27	30
	140	14	16	18	20	22
	120	10	12	13	15	17
65−69	180	22	25	27	30	33
	160	16	18	20	22	25
	140	11	13	14	16	18
	120	8	9	10	11	13
60−64	180	20	22	25	27	30
	160	14	15	17	19	22
	140	9	11	12	13	15
	120	6	7	8	9	10
55−59	180	16	18	21	23	25
	160	11	12	14	16	17
	140	7	8	9	10	12
	120	5	5	6	7	8
50−54	180	15	17	19	21	23
	160	9	11	12	14	15
	140	6	7	8	9	10
	120	4	4	5	5	6
45−49	180	14	16	18	20	22
	160	9	10	11	13	14
	140	5	6	7	8	9
	120	3	4	4	5	5
40−44	180	15	16	18	20	23
	160	9	10	11	13	14
	140	5	6	7	8	9
	120	3	3	4	4	5

Women – Diabetic – Non−smoker

Age	SBP	3	4	5	6	7
70−74	180	33	37	40	44	48
	160	25	28	31	34	38
	140	19	21	24	26	29
	120	14	16	18	20	22
65−69	180	29	32	35	39	42
	160	21	23	26	29	32
	140	15	17	19	21	23
	120	11	12	14	15	17
60−64	180	26	29	32	35	39
	160	18	20	23	25	28
	140	13	14	16	18	20
	120	9	10	11	12	14
55−59	180	22	24	27	30	33
	160	15	16	18	20	23
	140	10	11	12	14	15
	120	6	7	8	9	10
50−54	180	20	22	24	27	30
	160	13	14	16	18	20
	140	8	9	10	12	13
	120	5	6	6	7	8
45−49	180	19	21	23	26	29
	160	12	13	15	17	19
	140	7	8	9	10	12
	120	4	5	6	6	7
40−44	180	19	22	24	27	29
	160	12	13	15	17	19
	140	7	8	9	10	11
	120	4	5	5	6	7

Women – Diabetic – Smoker

Age	SBP	3	4	5	6	7
70−74	180	48	52	56	61	65
	160	38	41	45	49	53
	140	29	32	35	39	42
	120	22	24	27	30	33
65−69	180	42	46	50	54	59
	160	32	35	39	42	46
	140	23	26	29	32	35
	120	17	19	21	23	26
60−64	180	38	42	46	50	54
	160	28	31	34	37	41
	140	20	22	24	27	30
	120	14	15	17	19	21
55−59	180	33	36	39	43	47
	160	23	25	28	31	34
	140	15	17	19	21	24
	120	10	11	13	14	16
50−54	180	30	33	36	40	44
	160	20	22	25	27	30
	140	13	15	16	18	20
	120	8	9	10	12	13
45−49	180	29	32	35	38	42
	160	19	21	23	26	28
	140	12	13	15	17	18
	120	7	8	9	10	12
40−44	180	29	32	36	39	43
	160	19	21	23	26	28
	140	11	13	14	16	18
	120	7	8	9	10	11

Men – Non−diabetic – Non−smoker

Age	SBP	3	4	5	6	7
70−74	180	22	24	27	30	33
	160	16	18	20	23	25
	140	12	13	15	17	19
	120	9	10	11	12	14
65−69	180	19	21	24	27	29
	160	14	15	17	19	22
	140	10	11	12	14	15
	120	7	8	9	10	11
60−64	180	18	20	23	25	28
	160	13	14	16	18	20
	140	9	10	11	12	14
	120	6	7	7	8	9
55−59	180	15	17	19	21	24
	160	10	12	13	15	16
	140	7	8	9	10	11
	120	4	5	6	6	7
50−54	180	11	13	15	16	18
	160	7	8	9	10	12
	140	5	5	6	7	7
	120	3	3	4	4	5
45−49	180	9	10	11	13	14
	160	5	6	7	8	9
	140	3	4	4	5	5
	120	2	2	2	3	3
40−44	180	5	6	7	8	9
	160	3	3	4	4	5
	140	2	2	2	2	3
	120	1	1	1	1	1

Men – Non−diabetic – Smoker

Age	SBP	3	4	5	6	7
70−74	180	29	32	36	39	43
	160	22	24	27	30	33
	140	16	18	20	23	25
	120	12	14	15	17	19
65−69	180	26	29	32	35	38
	160	19	21	23	26	29
	140	13	15	17	19	21
	120	9	11	12	13	15
60−64	180	25	27	30	33	37
	160	17	19	21	24	27
	140	12	13	15	17	19
	120	8	9	10	12	13
55−59	180	21	23	26	29	32
	160	14	16	18	20	22
	140	9	10	12	13	15
	120	6	7	8	9	10
50−54	180	16	18	20	22	24
	160	10	11	13	14	16
	140	6	7	8	9	10
	120	4	4	5	6	7
45−49	180	12	14	16	17	20
	160	8	9	10	11	12
	140	5	5	6	7	8
	120	3	3	4	4	5
40−44	180	7	8	9	10	12
	160	4	5	5	6	7
	140	2	3	3	4	4
	120	1	1	2	2	2

Men – Diabetic – Non−smoker

Age	SBP	3	4	5	6	7
70−74	180	39	42	46	50	55
	160	30	33	36	40	44
	140	22	25	28	31	34
	120	17	19	21	23	26
65−69	180	34	38	42	45	49
	160	25	28	31	34	38
	140	18	21	23	26	28
	120	13	15	17	19	21
60−64	180	33	36	40	44	48
	160	24	26	29	32	35
	140	16	18	21	23	25
	120	11	13	14	16	18
55−59	180	28	31	34	38	42
	160	19	22	24	27	30
	140	13	15	16	18	20
	120	9	10	11	12	14
50−54	180	22	24	27	30	33
	160	14	16	18	20	22
	140	9	10	11	13	14
	120	6	6	7	8	9
45−49	180	17	19	21	24	26
	160	11	12	14	15	17
	140	7	7	8	10	11
	120	4	5	5	6	7
40−44	180	10	12	13	15	16
	160	6	7	8	9	10
	140	3	4	5	5	6
	120	2	2	3	3	3

Men – Diabetic – Smoker

Age	SBP	3	4	5	6	7
70−74	180	49	53	58	62	67
	160	39	42	46	51	55
	140	30	33	36	40	44
	120	23	25	28	31	34
65−69	180	44	48	53	57	61
	160	34	37	41	44	48
	140	25	27	30	34	37
	120	18	20	22	25	28
60−64	180	43	46	51	55	59
	160	31	34	38	42	45
	140	22	25	27	30	34
	120	16	17	19	22	24
55−59	180	37	41	44	48	53
	160	26	29	32	35	39
	140	18	20	22	25	27
	120	12	13	15	17	19
50−54	180	29	32	35	39	42
	160	19	21	24	26	29
	140	12	14	16	18	20
	120	8	9	10	11	13
45−49	180	23	26	29	32	35
	160	15	17	19	21	23
	140	9	10	12	13	15
	120	6	6	7	8	9
40−44	180	14	16	18	20	22
	160	9	10	11	12	14
	140	5	6	6	7	8
	120	3	3	4	4	5

Saint Vincent and the Grenadines

Systolic Blood Pressur (mmHg) by Total cholteserol (mmol/L)

Age 70–74

Women

SBP	Non−diabetic, Non−smoker					Non−diabetic, Smoker					Diabetic, Non−smoker					Diabetic, Smoker				
	3	4	5	6	7	3	4	5	6	7	3	4	5	6	7	3	4	5	6	7
180	33	36	40	44	48	48	52	56	60	65	58	63	67	71	75	75	79	83	86	89
160	25	28	31	34	37	37	41	45	49	53	47	51	55	60	64	64	68	72	77	80
140	19	21	23	26	29	29	32	35	38	42	37	40	44	48	52	52	56	61	65	69
120	14	16	17	19	22	22	24	27	30	33	28	31	34	38	41	41	45	49	53	58

Men

SBP	Non−diabetic, Non−smoker					Non−diabetic, Smoker					Diabetic, Non−smoker					Diabetic, Smoker				
	3	4	5	6	7	3	4	5	6	7	3	4	5	6	7	3	4	5	6	7
180	33	37	40	44	48	43	47	51	55	60	55	59	63	68	72	67	71	75	79	83
160	25	28	31	34	38	33	37	40	44	48	44	48	52	56	60	55	59	64	68	72
140	19	21	23	26	29	25	28	31	34	38	34	37	41	45	49	44	48	52	56	61
120	14	16	17	20	22	19	21	24	26	29	26	29	32	35	38	34	37	41	45	49

Age 65–69

Women

SBP	Non−diabetic, Non−smoker					Non−diabetic, Smoker					Diabetic, Non−smoker					Diabetic, Smoker				
	3	4	5	6	7	3	4	5	6	7	3	4	5	6	7	3	4	5	6	7
180	26	29	32	36	39	39	43	47	51	55	49	53	57	62	66	66	70	75	79	82
160	19	22	24	27	30	29	32	36	39	43	37	41	45	49	53	53	57	62	66	71
140	14	16	17	19	22	22	24	27	30	33	28	31	34	38	41	41	45	49	53	58
120	10	11	12	14	16	16	17	19	22	24	20	23	25	28	31	31	34	38	41	45

Men

SBP	Non−diabetic, Non−smoker					Non−diabetic, Smoker					Diabetic, Non−smoker					Diabetic, Smoker				
	3	4	5	6	7	3	4	5	6	7	3	4	5	6	7	3	4	5	6	7
180	32	35	39	42	46	41	45	49	53	58	53	57	62	66	70	65	69	73	78	81
160	23	26	29	32	35	31	34	38	41	45	41	45	49	53	57	52	56	60	65	69
140	17	19	21	23	26	23	25	28	31	34	31	34	37	41	45	40	44	48	52	56
120	12	14	15	17	19	16	18	21	23	25	23	25	28	31	34	30	33	36	40	44

Age 60–64

Women

SBP	Non−diabetic, Non−smoker					Non−diabetic, Smoker					Diabetic, Non−smoker					Diabetic, Smoker				
	3	4	5	6	7	3	4	5	6	7	3	4	5	6	7	3	4	5	6	7
180	21	24	26	29	32	32	35	39	43	47	41	45	49	53	57	57	61	66	70	74
160	15	17	19	21	23	23	26	28	31	35	30	33	36	40	44	43	47	52	56	60
140	10	11	13	14	16	16	18	20	22	25	21	24	26	29	32	32	35	39	43	47
120	7	8	9	10	11	11	12	14	16	18	15	17	19	21	23	23	25	28	31	35

Men

SBP	Non−diabetic, Non−smoker					Non−diabetic, Smoker					Diabetic, Non−smoker					Diabetic, Smoker				
	3	4	5	6	7	3	4	5	6	7	3	4	5	6	7	3	4	5	6	7
180	27	30	33	36	40	35	39	43	47	51	46	50	54	59	63	58	62	66	71	75
160	19	21	24	26	29	26	28	31	35	38	34	38	41	45	49	44	48	52	57	61
140	13	15	17	19	21	18	20	22	25	28	25	27	30	33	37	32	36	39	43	47
120	9	10	11	13	15	13	14	16	18	20	17	19	22	24	27	23	26	29	32	35

Age 55–59

Women

SBP	Non−diabetic, Non−smoker					Non−diabetic, Smoker					Diabetic, Non−smoker					Diabetic, Smoker				
	3	4	5	6	7	3	4	5	6	7	3	4	5	6	7	3	4	5	6	7
180	18	20	23	25	28	28	31	34	37	41	36	39	43	47	51	51	55	59	64	68
160	12	14	15	17	19	19	21	24	26	29	25	28	31	34	37	37	41	45	49	53
140	8	9	10	11	13	13	14	16	18	20	17	19	21	24	26	26	29	32	35	39
120	5	6	7	8	8	8	9	11	12	14	11	13	14	16	18	18	20	22	25	27

Men

SBP	Non−diabetic, Non−smoker					Non−diabetic, Smoker					Diabetic, Non−smoker					Diabetic, Smoker				
	3	4	5	6	7	3	4	5	6	7	3	4	5	6	7	3	4	5	6	7
180	21	23	26	29	32	28	31	34	38	41	37	41	45	49	53	48	52	56	60	65
160	14	16	18	20	22	19	22	24	27	29	26	29	32	35	39	34	38	42	46	50
140	9	11	12	13	15	13	15	16	18	20	18	20	22	25	28	24	27	30	33	36
120	6	7	8	9	10	9	10	11	12	14	12	14	15	17	19	17	18	21	23	26

Age 50–54

Women

SBP	Non−diabetic, Non−smoker					Non−diabetic, Smoker					Diabetic, Non−smoker					Diabetic, Smoker				
	3	4	5	6	7	3	4	5	6	7	3	4	5	6	7	3	4	5	6	7
180	15	17	19	21	24	23	26	29	32	35	30	34	37	41	44	44	48	52	57	61
160	10	11	12	14	16	15	17	19	22	24	20	23	25	28	31	31	34	37	41	45
140	6	7	8	9	10	10	11	13	14	16	13	15	17	19	21	21	23	26	28	31
120	4	4	5	6	6	6	7	8	9	10	8	10	11	12	14	13	15	17	19	21

Men

SBP	Non−diabetic, Non−smoker					Non−diabetic, Smoker					Diabetic, Non−smoker					Diabetic, Smoker				
	3	4	5	6	7	3	4	5	6	7	3	4	5	6	7	3	4	5	6	7
180	14	16	17	19	22	19	21	23	26	29	26	28	31	35	38	34	37	41	45	49
160	9	10	11	13	14	12	14	15	17	19	17	19	21	24	26	23	25	28	31	34
140	6	6	7	8	9	8	9	10	11	13	11	12	14	16	17	15	17	19	21	23
120	3	4	4	5	6	5	6	6	7	8	7	8	9	10	11	10	11	12	14	15

Age 45–49

Women

SBP	Non−diabetic, Non−smoker					Non−diabetic, Smoker					Diabetic, Non−smoker					Diabetic, Smoker				
	3	4	5	6	7	3	4	5	6	7	3	4	5	6	7	3	4	5	6	7
180	13	15	17	19	21	21	23	25	28	31	27	30	33	36	40	39	43	47	51	56
160	8	9	10	12	13	13	15	16	18	20	17	19	21	24	27	26	29	32	36	39
140	5	6	6	7	8	8	9	10	11	13	11	12	14	15	17	17	19	21	24	26
120	3	3	4	4	5	5	5	6	7	8	7	7	8	9	11	11	12	13	15	17

Men

SBP	Non−diabetic, Non−smoker					Non−diabetic, Smoker					Diabetic, Non−smoker					Diabetic, Smoker				
	3	4	5	6	7	3	4	5	6	7	3	4	5	6	7	3	4	5	6	7
180	12	13	15	17	18	16	18	20	22	25	22	24	27	30	33	29	32	36	39	43
160	7	8	9	10	12	10	11	13	14	16	14	16	17	19	22	19	21	23	26	29
140	4	5	6	6	7	6	7	8	9	10	9	10	11	12	14	12	13	15	17	19
120	2	3	3	4	4	4	4	5	5	6	5	6	7	8	8	7	8	9	10	12

Age 40–44

Women

SBP	Non−diabetic, Non−smoker					Non−diabetic, Smoker					Diabetic, Non−smoker					Diabetic, Smoker				
	3	4	5	6	7	3	4	5	6	7	3	4	5	6	7	3	4	5	6	7
180	11	12	13	15	17	17	19	21	23	26	22	24	27	30	33	33	36	40	44	48
160	6	7	8	9	10	10	11	13	14	16	13	15	17	19	21	21	23	26	29	32
140	4	4	5	5	6	6	7	8	9	10	8	9	10	12	13	13	14	16	18	20
120	2	2	3	3	3	3	4	4	5	6	5	5	6	7	8	8	9	10	11	12

Men

SBP	Non−diabetic, Non−smoker					Non−diabetic, Smoker					Diabetic, Non−smoker					Diabetic, Smoker				
	3	4	5	6	7	3	4	5	6	7	3	4	5	6	7	3	4	5	6	7
180	10	11	13	14	16	14	15	17	19	21	19	21	24	26	29	25	28	31	34	38
160	6	7	8	9	10	8	9	10	12	13	12	13	15	16	18	16	18	20	22	25
140	3	4	4	5	6	5	5	6	7	8	7	8	9	10	11	10	11	12	14	15
120	2	2	2	3	3	3	3	4	4	5	4	4	5	6	7	6	6	7	8	9

Suriname

Systolic Blood Pressur (mmHg) by Total cholteserol (mmol/L)

Age	Sex	Diabetes	Smoking	SBP	3	4	5	6	7
70–74	Women	Non–diabetic	Non–smoker	180	20	22	24	27	30
70–74	Women	Non–diabetic	Non–smoker	160	15	16	18	20	23
70–74	Women	Non–diabetic	Non–smoker	140	11	12	13	15	17
70–74	Women	Non–diabetic	Non–smoker	120	8	9	10	11	12
70–74	Women	Non–diabetic	Smoker	180	30	33	36	40	44
70–74	Women	Non–diabetic	Smoker	160	23	25	28	31	34
70–74	Women	Non–diabetic	Smoker	140	17	19	21	23	26
70–74	Women	Non–diabetic	Smoker	120	12	14	16	17	19
70–74	Women	Diabetic	Non–smoker	180	38	42	46	50	54
70–74	Women	Diabetic	Non–smoker	160	29	32	36	39	43
70–74	Women	Diabetic	Non–smoker	140	22	25	27	30	33
70–74	Women	Diabetic	Non–smoker	120	16	18	20	23	25
70–74	Women	Diabetic	Smoker	180	54	58	62	67	71
70–74	Women	Diabetic	Smoker	160	43	47	51	55	59
70–74	Women	Diabetic	Smoker	140	33	37	40	44	48
70–74	Women	Diabetic	Smoker	120	25	28	31	34	38
70–74	Men	Non–diabetic	Non–smoker	180	27	30	33	36	40
70–74	Men	Non–diabetic	Non–smoker	160	20	22	25	28	31
70–74	Men	Non–diabetic	Non–smoker	140	15	17	19	21	23
70–74	Men	Non–diabetic	Non–smoker	120	11	12	14	15	17
70–74	Men	Non–diabetic	Smoker	180	35	39	42	46	51
70–74	Men	Non–diabetic	Smoker	160	27	30	33	36	40
70–74	Men	Non–diabetic	Smoker	140	20	22	25	28	31
70–74	Men	Non–diabetic	Smoker	120	15	17	19	21	23
70–74	Men	Diabetic	Non–smoker	180	46	50	54	58	63
70–74	Men	Diabetic	Non–smoker	160	36	39	43	47	51
70–74	Men	Diabetic	Non–smoker	140	27	30	33	37	40
70–74	Men	Diabetic	Non–smoker	120	20	23	25	28	31
70–74	Men	Diabetic	Smoker	180	57	62	66	70	75
70–74	Men	Diabetic	Smoker	160	46	50	54	59	63
70–74	Men	Diabetic	Smoker	140	36	39	43	47	51
70–74	Men	Diabetic	Smoker	120	27	30	34	37	41
65–69	Women	Non–diabetic	Non–smoker	180	15	17	19	21	23
65–69	Women	Non–diabetic	Non–smoker	160	11	12	13	15	17
65–69	Women	Non–diabetic	Non–smoker	140	7	8	10	11	12
65–69	Women	Non–diabetic	Non–smoker	120	5	6	7	7	8
65–69	Women	Non–diabetic	Smoker	180	23	26	29	32	35
65–69	Women	Non–diabetic	Smoker	160	17	19	21	23	26
65–69	Women	Non–diabetic	Smoker	140	12	13	15	17	19
65–69	Women	Non–diabetic	Smoker	120	8	9	11	12	13
65–69	Women	Diabetic	Non–smoker	180	30	33	37	40	44
65–69	Women	Diabetic	Non–smoker	160	22	25	27	30	33
65–69	Women	Diabetic	Non–smoker	140	16	18	20	22	25
65–69	Women	Diabetic	Non–smoker	120	11	13	14	16	18
65–69	Women	Diabetic	Smoker	180	44	48	52	56	61
65–69	Women	Diabetic	Smoker	160	33	37	40	44	48
65–69	Women	Diabetic	Smoker	140	25	27	30	33	37
65–69	Women	Diabetic	Smoker	120	18	20	22	25	27
65–69	Men	Non–diabetic	Non–smoker	180	24	27	30	33	37
65–69	Men	Non–diabetic	Non–smoker	160	18	20	22	25	27
65–69	Men	Non–diabetic	Non–smoker	140	13	14	16	18	20
65–69	Men	Non–diabetic	Non–smoker	120	9	10	11	13	14
65–69	Men	Non–diabetic	Smoker	180	32	36	39	43	47
65–69	Men	Non–diabetic	Smoker	160	24	26	29	32	36
65–69	Men	Non–diabetic	Smoker	140	17	19	21	24	27
65–69	Men	Non–diabetic	Smoker	120	12	14	15	17	19
65–69	Men	Diabetic	Non–smoker	180	42	46	50	55	59
65–69	Men	Diabetic	Non–smoker	160	32	35	39	43	46
65–69	Men	Diabetic	Non–smoker	140	23	26	29	32	35
65–69	Men	Diabetic	Non–smoker	120	17	19	21	24	26
65–69	Men	Diabetic	Smoker	180	54	58	62	67	71
65–69	Men	Diabetic	Smoker	160	41	45	49	54	58
65–69	Men	Diabetic	Smoker	140	31	34	38	42	45
65–69	Men	Diabetic	Smoker	120	23	25	28	31	34
60–64	Women	Non–diabetic	Non–smoker	180	12	13	15	17	19
60–64	Women	Non–diabetic	Non–smoker	160	8	9	10	11	13
60–64	Women	Non–diabetic	Non–smoker	140	5	6	7	8	9
60–64	Women	Non–diabetic	Non–smoker	120	3	4	5	5	6
60–64	Women	Non–diabetic	Smoker	180	19	21	23	26	28
60–64	Women	Non–diabetic	Smoker	160	13	14	16	18	20
60–64	Women	Non–diabetic	Smoker	140	9	10	11	12	14
60–64	Women	Non–diabetic	Smoker	120	6	7	7	8	10
60–64	Women	Diabetic	Non–smoker	180	24	27	30	33	36
60–64	Women	Diabetic	Non–smoker	160	17	19	21	24	26
60–64	Women	Diabetic	Non–smoker	140	12	13	15	17	18
60–64	Women	Diabetic	Non–smoker	120	8	9	10	11	13
60–64	Women	Diabetic	Smoker	180	36	40	44	48	52
60–64	Women	Diabetic	Smoker	160	26	29	32	35	39
60–64	Women	Diabetic	Smoker	140	18	20	23	25	28
60–64	Women	Diabetic	Smoker	120	13	14	16	18	20
60–64	Men	Non–diabetic	Non–smoker	180	21	24	26	29	32
60–64	Men	Non–diabetic	Non–smoker	160	15	17	19	21	23
60–64	Men	Non–diabetic	Non–smoker	140	10	11	13	14	16
60–64	Men	Non–diabetic	Non–smoker	120	7	8	9	10	11
60–64	Men	Non–diabetic	Smoker	180	28	31	35	38	42
60–64	Men	Non–diabetic	Smoker	160	20	22	25	28	31
60–64	Men	Non–diabetic	Smoker	140	14	16	18	20	22
60–64	Men	Non–diabetic	Smoker	120	10	11	12	14	15
60–64	Men	Diabetic	Non–smoker	180	38	41	45	49	54
60–64	Men	Diabetic	Non–smoker	160	27	30	33	37	40
60–64	Men	Diabetic	Non–smoker	140	19	22	24	27	29
60–64	Men	Diabetic	Non–smoker	120	13	15	17	19	21
60–64	Men	Diabetic	Smoker	180	48	52	57	61	66
60–64	Men	Diabetic	Smoker	160	36	39	43	47	51
60–64	Men	Diabetic	Smoker	140	26	29	32	35	39
60–64	Men	Diabetic	Smoker	120	18	20	23	25	28
55–59	Women	Non–diabetic	Non–smoker	180	10	11	13	14	16
55–59	Women	Non–diabetic	Non–smoker	160	7	7	8	9	11
55–59	Women	Non–diabetic	Non–smoker	140	4	5	5	6	7
55–59	Women	Non–diabetic	Non–smoker	120	3	3	3	4	4
55–59	Women	Non–diabetic	Smoker	180	16	18	20	22	25
55–59	Women	Non–diabetic	Smoker	160	11	12	13	15	17
55–59	Women	Non–diabetic	Smoker	140	7	8	9	10	11
55–59	Women	Non–diabetic	Smoker	120	4	5	6	6	7
55–59	Women	Diabetic	Non–smoker	180	21	23	26	29	32
55–59	Women	Diabetic	Non–smoker	160	14	16	18	20	22
55–59	Women	Diabetic	Non–smoker	140	9	11	12	13	15
55–59	Women	Diabetic	Non–smoker	120	6	7	8	9	10
55–59	Women	Diabetic	Smoker	180	32	35	39	42	46
55–59	Women	Diabetic	Smoker	160	22	24	27	30	33
55–59	Women	Diabetic	Smoker	140	15	17	19	21	23
55–59	Women	Diabetic	Smoker	120	10	11	12	14	16
55–59	Men	Non–diabetic	Non–smoker	180	20	22	25	28	31
55–59	Men	Non–diabetic	Non–smoker	160	13	15	17	19	21
55–59	Men	Non–diabetic	Non–smoker	140	9	10	11	13	14
55–59	Men	Non–diabetic	Non–smoker	120	6	6	7	8	9
55–59	Men	Non–diabetic	Smoker	180	27	30	33	36	40
55–59	Men	Non–diabetic	Smoker	160	18	20	23	25	28
55–59	Men	Non–diabetic	Smoker	140	12	14	15	17	19
55–59	Men	Non–diabetic	Smoker	120	8	9	10	11	13
55–59	Men	Diabetic	Non–smoker	180	36	39	43	47	51
55–59	Men	Diabetic	Non–smoker	160	25	28	31	34	37
55–59	Men	Diabetic	Non–smoker	140	17	19	21	24	26
55–59	Men	Diabetic	Non–smoker	120	11	13	14	16	18
55–59	Men	Diabetic	Smoker	180	46	50	54	59	63
55–59	Men	Diabetic	Smoker	160	33	36	40	44	48
55–59	Men	Diabetic	Smoker	140	23	25	28	31	34
55–59	Men	Diabetic	Smoker	120	15	17	19	22	24
50–54	Women	Non–diabetic	Non–smoker	180	11	12	13	15	17
50–54	Women	Non–diabetic	Non–smoker	160	7	8	9	10	11
50–54	Women	Non–diabetic	Non–smoker	140	4	5	5	6	7
50–54	Women	Non–diabetic	Non–smoker	120	2	3	3	4	4
50–54	Women	Non–diabetic	Smoker	180	17	19	21	23	26
50–54	Women	Non–diabetic	Smoker	160	11	12	14	15	17
50–54	Women	Non–diabetic	Smoker	140	7	8	9	10	11
50–54	Women	Non–diabetic	Smoker	120	4	5	5	6	7
50–54	Women	Diabetic	Non–smoker	180	22	25	27	30	33
50–54	Women	Diabetic	Non–smoker	160	14	16	18	20	22
50–54	Women	Diabetic	Non–smoker	140	9	10	12	13	15
50–54	Women	Diabetic	Non–smoker	120	6	7	7	8	9
50–54	Women	Diabetic	Smoker	180	33	37	40	44	48
50–54	Women	Diabetic	Smoker	160	22	25	28	31	34
50–54	Women	Diabetic	Smoker	140	15	16	18	20	23
50–54	Women	Diabetic	Smoker	120	9	11	12	13	15
50–54	Men	Non–diabetic	Non–smoker	180	20	22	25	28	31
50–54	Men	Non–diabetic	Non–smoker	160	13	15	16	18	20
50–54	Men	Non–diabetic	Non–smoker	140	8	9	11	12	13
50–54	Men	Non–diabetic	Non–smoker	120	5	6	7	8	8
50–54	Men	Non–diabetic	Smoker	180	27	30	33	36	40
50–54	Men	Non–diabetic	Smoker	160	18	20	22	25	27
50–54	Men	Non–diabetic	Smoker	140	11	13	14	16	18
50–54	Men	Non–diabetic	Smoker	120	7	8	9	10	12
50–54	Men	Diabetic	Non–smoker	180	36	39	43	47	51
50–54	Men	Diabetic	Non–smoker	160	24	27	30	33	36
50–54	Men	Diabetic	Non–smoker	140	16	18	20	22	25
50–54	Men	Diabetic	Non–smoker	120	10	12	13	15	16
50–54	Men	Diabetic	Smoker	180	46	50	54	59	63
50–54	Men	Diabetic	Smoker	160	32	35	39	43	47
50–54	Men	Diabetic	Smoker	140	22	24	27	30	33
50–54	Men	Diabetic	Smoker	120	14	16	18	20	22
45–49	Women	Non–diabetic	Non–smoker	180	10	12	13	15	16
45–49	Women	Non–diabetic	Non–smoker	160	6	7	8	9	10
45–49	Women	Non–diabetic	Non–smoker	140	4	4	5	5	6
45–49	Women	Non–diabetic	Non–smoker	120	2	2	3	3	4
45–49	Women	Non–diabetic	Smoker	180	16	18	20	23	25
45–49	Women	Non–diabetic	Smoker	160	10	11	13	14	16
45–49	Women	Non–diabetic	Smoker	140	6	7	8	9	10
45–49	Women	Non–diabetic	Smoker	120	4	4	5	5	6
45–49	Women	Diabetic	Non–smoker	180	21	24	26	29	32
45–49	Women	Diabetic	Non–smoker	160	13	15	17	19	21
45–49	Women	Diabetic	Non–smoker	140	8	9	11	12	13
45–49	Women	Diabetic	Non–smoker	120	5	6	6	7	8
45–49	Women	Diabetic	Smoker	180	32	35	39	43	47
45–49	Women	Diabetic	Smoker	160	21	23	26	29	32
45–49	Women	Diabetic	Smoker	140	13	15	17	19	21
45–49	Women	Diabetic	Smoker	120	8	9	10	12	13
45–49	Men	Non–diabetic	Non–smoker	180	19	22	24	27	30
45–49	Men	Non–diabetic	Non–smoker	160	12	14	15	17	19
45–49	Men	Non–diabetic	Non–smoker	140	8	8	10	11	12
45–49	Men	Non–diabetic	Non–smoker	120	4	5	6	7	7
45–49	Men	Non–diabetic	Smoker	180	26	29	32	35	39
45–49	Men	Non–diabetic	Smoker	160	17	19	21	23	26
45–49	Men	Non–diabetic	Smoker	140	10	12	13	15	17
45–49	Men	Non–diabetic	Smoker	120	6	7	8	9	10
45–49	Men	Diabetic	Non–smoker	180	35	38	42	46	50
45–49	Men	Diabetic	Non–smoker	160	23	25	28	31	34
45–49	Men	Diabetic	Non–smoker	140	15	16	18	20	23
45–49	Men	Diabetic	Non–smoker	120	9	10	11	13	14
45–49	Men	Diabetic	Smoker	180	45	49	53	57	62
45–49	Men	Diabetic	Smoker	160	30	34	37	41	44
45–49	Men	Diabetic	Smoker	140	20	22	24	27	30
45–49	Men	Diabetic	Smoker	120	12	14	16	18	20
40–44	Women	Non–diabetic	Non–smoker	180	8	9	10	11	13
40–44	Women	Non–diabetic	Non–smoker	160	5	5	6	7	8
40–44	Women	Non–diabetic	Non–smoker	140	3	3	3	4	4
40–44	Women	Non–diabetic	Non–smoker	120	1	2	2	2	2
40–44	Women	Non–diabetic	Smoker	180	13	14	16	18	20
40–44	Women	Non–diabetic	Smoker	160	8	8	10	11	12
40–44	Women	Non–diabetic	Smoker	140	4	5	6	6	7
40–44	Women	Non–diabetic	Smoker	120	2	3	3	4	4
40–44	Women	Diabetic	Non–smoker	180	17	19	21	23	26
40–44	Women	Diabetic	Non–smoker	160	10	11	13	14	16
40–44	Women	Diabetic	Non–smoker	140	6	7	8	9	10
40–44	Women	Diabetic	Non–smoker	120	3	4	4	5	6
40–44	Women	Diabetic	Smoker	180	26	29	32	35	38
40–44	Women	Diabetic	Smoker	160	16	18	20	22	25
40–44	Women	Diabetic	Smoker	140	10	11	12	14	15
40–44	Women	Diabetic	Smoker	120	6	6	7	8	9
40–44	Men	Non–diabetic	Non–smoker	180	14	16	17	19	22
40–44	Men	Non–diabetic	Non–smoker	160	8	9	11	12	13
40–44	Men	Non–diabetic	Non–smoker	140	5	5	6	7	8
40–44	Men	Non–diabetic	Non–smoker	120	3	3	4	4	5
40–44	Men	Non–diabetic	Smoker	180	19	21	23	26	29
40–44	Men	Non–diabetic	Smoker	160	11	13	14	16	18
40–44	Men	Non–diabetic	Smoker	140	7	8	9	10	11
40–44	Men	Non–diabetic	Smoker	120	4	4	5	6	7
40–44	Men	Diabetic	Non–smoker	180	26	28	31	35	38
40–44	Men	Diabetic	Non–smoker	160	16	18	20	22	25
40–44	Men	Diabetic	Non–smoker	140	10	11	12	14	15
40–44	Men	Diabetic	Non–smoker	120	6	6	7	8	9
40–44	Men	Diabetic	Smoker	180	34	37	41	45	49
40–44	Men	Diabetic	Smoker	160	22	24	27	30	33
40–44	Men	Diabetic	Smoker	140	13	15	17	19	21
40–44	Men	Diabetic	Smoker	120	8	9	10	11	13

The Bahamas

Systolic Blood Pressur (mmHg) by Total cholteserol (mmol/L)

Women

Non−diabetic

Non−smoker

Age	Systolic Blood Pressur (mmHg)	3	4	5	6	7
70−74	180	12	13	15	17	19
	160	9	10	11	12	14
	140	6	7	8	9	10
	120	4	5	6	6	7
65−69	180	10	11	12	14	16
	160	7	8	9	10	11
	140	5	5	6	7	8
	120	3	4	4	5	5
60−64	180	8	9	10	12	13
	160	5	6	7	8	9
	140	4	4	5	5	6
	120	2	3	3	3	4
55−59	180	7	8	9	10	12
	160	5	5	6	7	8
	140	3	3	4	4	5
	120	2	2	2	3	3
50−54	180	7	8	9	10	12
	160	4	5	6	7	7
	140	3	3	4	4	5
	120	2	2	2	2	3
45−49	180	6	7	8	9	10
	160	4	4	5	5	6
	140	2	2	3	3	4
	120	1	1	1	2	2
40−44	180	4	5	6	6	7
	160	2	3	3	4	4
	140	1	1	2	2	2
	120	1	1	1	1	1

Smoker

Age	Systolic Blood Pressur (mmHg)	3	4	5	6	7
70−74	180	18	21	23	26	28
	160	14	15	17	19	21
	140	10	11	13	14	16
	120	7	8	9	10	12
65−69	180	15	17	19	22	24
	160	11	12	14	15	17
	140	8	9	10	11	12
	120	5	6	7	8	9
60−64	180	13	14	16	18	20
	160	9	10	11	13	14
	140	6	7	8	9	10
	120	4	4	5	6	6
55−59	180	12	13	15	16	18
	160	8	9	10	11	12
	140	5	5	6	7	8
	120	3	3	4	5	5
50−54	180	12	13	15	16	18
	160	7	8	9	11	12
	140	5	5	6	7	8
	120	3	3	4	4	5
45−49	180	10	11	13	14	16
	160	6	7	8	9	10
	140	4	4	5	5	6
	120	2	2	3	3	4
40−44	180	7	8	9	10	12
	160	4	5	5	6	7
	140	2	3	3	3	4
	120	1	1	2	2	2

Diabetic

Non−smoker

Age	Systolic Blood Pressur (mmHg)	3	4	5	6	7
70−74	180	24	27	30	33	36
	160	18	20	22	25	28
	140	13	15	17	19	21
	120	10	11	12	14	15
65−69	180	20	23	25	28	31
	160	15	16	18	20	23
	140	10	12	13	15	16
	120	7	8	9	10	12
60−64	180	17	19	21	24	26
	160	12	13	15	17	19
	140	8	9	10	11	13
	120	5	6	7	8	9
55−59	180	15	17	19	21	24
	160	10	11	13	14	16
	140	7	7	8	10	11
	120	4	5	5	6	7
50−54	180	15	17	19	22	24
	160	10	11	13	14	16
	140	6	7	8	9	10
	120	4	4	5	6	6
45−49	180	13	15	17	19	21
	160	8	9	10	12	13
	140	5	6	6	7	8
	120	3	3	4	4	5
40−44	180	10	11	12	14	15
	160	6	6	7	8	9
	140	3	4	4	5	5
	120	2	2	2	3	3

Smoker

Age	Systolic Blood Pressur (mmHg)	3	4	5	6	7
70−74	180	36	40	43	47	52
	160	28	31	34	37	41
	140	21	23	26	28	31
	120	15	17	19	21	24
65−69	180	31	34	37	41	45
	160	23	25	28	31	34
	140	16	18	20	23	25
	120	12	13	15	16	18
60−64	180	26	29	32	36	39
	160	19	21	23	26	28
	140	13	14	16	18	20
	120	9	10	11	12	14
55−59	180	24	26	29	32	36
	160	16	18	20	22	25
	140	11	12	13	15	17
	120	7	8	9	10	11
50−54	180	24	27	29	33	36
	160	16	18	20	22	24
	140	10	11	13	14	16
	120	6	7	8	9	10
45−49	180	21	23	26	28	31
	160	13	15	16	18	20
	140	8	9	10	12	13
	120	5	5	6	7	8
40−44	180	15	17	19	21	24
	160	9	10	12	13	15
	140	5	6	7	8	9
	120	3	4	4	5	5

Men

Non−diabetic

Non−smoker

Age	Systolic Blood Pressur (mmHg)	3	4	5	6	7
70−74	180	15	17	19	22	24
	160	11	13	14	16	18
	140	8	9	10	12	13
	120	6	7	8	9	10
65−69	180	13	14	16	18	20
	160	9	10	11	13	14
	140	6	7	8	9	10
	120	4	5	5	6	7
60−64	180	13	14	16	18	20
	160	9	10	11	12	14
	140	6	7	7	8	9
	120	4	4	5	6	6
55−59	180	13	15	17	19	21
	160	9	10	11	12	14
	140	6	6	7	8	9
	120	4	4	5	5	6
50−54	180	13	15	16	18	21
	160	8	9	11	12	13
	140	5	6	7	8	9
	120	3	4	4	5	5
45−49	180	12	14	16	18	20
	160	8	9	10	11	12
	140	5	5	6	7	8
	120	3	3	3	4	5
40−44	180	11	12	14	15	17
	160	6	7	8	9	10
	140	4	4	5	5	6
	120	2	2	3	3	3

Smoker

Age	Systolic Blood Pressur (mmHg)	3	4	5	6	7
70−74	180	21	23	26	29	32
	160	15	17	19	22	24
	140	11	13	14	16	18
	120	8	9	10	12	13
65−69	180	17	19	22	24	27
	160	12	14	16	17	19
	140	9	10	11	12	14
	120	6	7	8	9	10
60−64	180	17	19	22	24	27
	160	12	13	15	17	19
	140	8	9	10	12	13
	120	5	6	7	8	9
55−59	180	18	20	22	25	28
	160	12	13	15	17	19
	140	8	9	10	11	13
	120	5	6	6	7	8
50−54	180	18	20	22	25	27
	160	12	13	15	16	18
	140	7	8	9	11	12
	120	5	5	6	7	7
45−49	180	17	19	21	24	26
	160	11	12	13	15	17
	140	6	7	8	9	11
	120	4	4	5	6	6
40−44	180	15	17	19	21	23
	160	9	10	11	13	14
	140	5	6	7	8	9
	120	3	3	4	4	5

Diabetic

Non−smoker

Age	Systolic Blood Pressur (mmHg)	3	4	5	6	7
70−74	180	28	31	35	38	42
	160	21	24	26	29	32
	140	16	18	20	22	25
	120	12	13	15	16	18
65−69	180	24	26	29	32	36
	160	17	19	21	24	26
	140	12	14	15	17	19
	120	9	10	11	12	14
60−64	180	24	26	29	32	36
	160	17	19	21	23	26
	140	11	13	14	16	18
	120	8	9	10	11	12
55−59	180	24	27	30	33	37
	160	17	19	21	23	26
	140	11	12	14	16	17
	120	7	8	9	10	12
50−54	180	24	27	30	33	37
	160	16	18	20	22	25
	140	10	12	13	15	16
	120	6	7	8	9	11
45−49	180	23	26	29	32	35
	160	15	17	19	21	23
	140	9	10	12	13	15
	120	6	6	7	8	9
40−44	180	20	23	25	28	31
	160	12	14	16	18	20
	140	7	8	9	11	12
	120	4	5	6	6	7

Smoker

Age	Systolic Blood Pressur (mmHg)	3	4	5	6	7
70−74	180	37	41	44	49	53
	160	28	31	35	38	42
	140	21	24	26	29	32
	120	16	18	20	22	25
65−69	180	32	35	38	42	46
	160	23	26	28	32	35
	140	17	19	21	23	26
	120	12	13	15	17	19
60−64	180	31	35	38	42	46
	160	22	25	28	31	34
	140	16	17	20	22	24
	120	11	12	13	15	17
55−59	180	32	36	39	43	47
	160	22	25	28	31	34
	140	15	17	19	21	23
	120	10	11	13	14	16
50−54	180	32	36	39	43	47
	160	22	24	27	30	33
	140	14	16	18	20	22
	120	9	10	11	13	14
45−49	180	31	34	37	41	45
	160	20	22	25	28	31
	140	13	14	16	18	20
	120	8	9	10	11	13
40−44	180	27	30	33	37	40
	160	17	19	21	24	26
	140	10	12	13	15	16
	120	6	7	8	9	10

Total cholteserol (mmol/L)

Trinidad and Tobago

Systolic Blood Pressur (mmHg) by Age; Total cholteserol (mmol/L)

Women — Non−diabetic — Non−smoker

Age	Systolic BP	3	4	5	6	7
70−74	180	23	25	28	31	34
	160	17	19	21	23	26
	140	12	14	16	17	19
	120	9	10	11	13	14
65−69	180	18	20	22	25	27
	160	13	14	16	18	20
	140	9	10	11	13	14
	120	6	7	8	9	10
60−64	180	16	17	19	22	24
	160	11	12	13	15	17
	140	7	8	9	10	12
	120	5	5	6	7	8
55−59	180	14	15	17	19	21
	160	9	10	11	13	14
	140	6	7	7	8	9
	120	4	4	5	5	6
50−54	180	11	13	14	16	18
	160	7	8	9	10	12
	140	4	5	6	6	7
	120	3	3	3	4	5
45−49	180	8	10	11	12	14
	160	5	6	7	7	8
	140	3	3	4	4	5
	120	2	2	2	3	3
40−44	180	6	7	8	9	10
	160	3	4	4	5	6
	140	2	2	2	3	3
	120	1	1	1	2	2

Women — Non−diabetic — Smoker

Age	Systolic BP	3	4	5	6	7
70−74	180	34	37	41	45	49
	160	26	29	32	35	38
	140	19	21	24	27	29
	120	14	16	18	20	22
65−69	180	27	30	33	37	40
	160	20	22	25	27	30
	140	14	16	18	20	22
	120	10	11	13	14	16
60−64	180	24	27	29	33	36
	160	17	19	21	23	26
	140	12	13	15	16	18
	120	8	9	10	11	13
55−59	180	21	23	26	29	32
	160	14	16	18	20	22
	140	9	11	12	13	15
	120	6	7	8	9	10
50−54	180	18	20	22	25	27
	160	11	13	14	16	18
	140	7	8	9	10	12
	120	4	5	6	7	7
45−49	180	13	15	17	19	21
	160	8	9	11	12	13
	140	5	6	6	7	8
	120	3	3	4	4	5
40−44	180	10	11	12	14	15
	160	6	6	7	8	9
	140	3	4	4	5	5
	120	2	2	2	3	3

Women — Diabetic — Non−smoker

Age	Systolic BP	3	4	5	6	7
70−74	180	43	47	51	55	59
	160	33	36	40	44	48
	140	25	28	31	34	38
	120	19	21	23	26	29
65−69	180	35	39	42	46	50
	160	26	29	32	35	39
	140	19	21	23	26	29
	120	13	15	17	19	21
60−64	180	31	34	38	41	45
	160	22	25	27	30	33
	140	15	17	19	21	24
	120	11	12	13	15	17
55−59	180	27	30	34	37	41
	160	19	21	23	26	29
	140	13	14	16	18	20
	120	8	9	11	12	13
50−54	180	23	26	29	32	35
	160	15	17	19	21	24
	140	10	11	12	14	16
	120	6	7	8	9	10
45−49	180	18	20	22	25	27
	160	11	13	14	16	18
	140	7	8	9	10	11
	120	4	5	5	6	7
40−44	180	13	15	16	18	20
	160	8	9	10	11	13
	140	4	5	6	7	7
	120	3	3	3	4	4

Women — Diabetic — Smoker

Age	Systolic BP	3	4	5	6	7
70−74	180	59	64	68	72	77
	160	48	52	56	61	65
	140	37	41	45	49	53
	120	29	32	35	39	42
65−69	180	50	54	59	63	67
	160	38	42	46	50	54
	140	29	32	35	39	42
	120	21	23	26	29	32
60−64	180	45	49	53	58	62
	160	33	37	40	44	48
	140	24	26	29	32	36
	120	17	19	21	23	26
55−59	180	40	44	48	52	57
	160	29	32	35	38	42
	140	20	22	24	27	30
	120	13	15	17	19	21
50−54	180	35	38	42	46	50
	160	24	26	29	32	35
	140	16	17	19	22	24
	120	10	11	13	14	16
45−49	180	27	30	33	37	40
	160	18	20	22	24	27
	140	11	12	14	16	17
	120	7	8	9	10	11
40−44	180	20	23	25	28	31
	160	12	14	16	18	20
	140	7	8	9	11	12
	120	4	5	6	6	7

Men — Non−diabetic — Non−smoker

Age	Systolic BP	3	4	5	6	7
70−74	180	28	31	35	38	42
	160	21	24	26	29	32
	140	16	18	20	22	25
	120	12	13	15	16	18
65−69	180	25	28	31	34	38
	160	18	20	23	25	28
	140	13	15	16	18	20
	120	9	10	12	13	15
60−64	180	23	26	29	32	35
	160	16	18	20	23	25
	140	11	13	14	16	18
	120	8	9	10	11	12
55−59	180	22	25	27	30	33
	160	15	17	19	21	23
	140	10	11	12	14	16
	120	6	7	8	9	10
50−54	180	20	22	24	27	30
	160	13	14	16	18	20
	140	8	9	10	12	13
	120	5	6	7	7	8
45−49	180	17	19	21	23	26
	160	10	12	13	15	17
	140	6	7	8	9	10
	120	4	4	5	6	6
40−44	180	12	14	16	18	20
	160	7	8	9	11	12
	140	4	5	6	6	7
	120	2	3	3	4	4

Men — Non−diabetic — Smoker

Age	Systolic BP	3	4	5	6	7
70−74	180	37	41	45	49	53
	160	29	32	35	38	42
	140	21	24	27	29	33
	120	16	18	20	22	25
65−69	180	33	37	40	44	48
	160	24	27	30	33	37
	140	18	20	22	24	27
	120	13	14	16	18	20
60−64	180	31	34	38	41	45
	160	22	25	27	30	33
	140	15	17	19	21	24
	120	11	12	13	15	17
55−59	180	29	32	36	39	43
	160	20	22	25	28	31
	140	14	15	17	19	21
	120	9	10	11	13	14
50−54	180	26	29	32	36	39
	160	17	19	22	24	27
	140	11	13	14	16	18
	120	7	8	9	10	12
45−49	180	23	25	28	31	34
	160	14	16	18	20	22
	140	9	10	11	13	14
	120	5	6	7	8	9
40−44	180	17	19	21	24	26
	160	10	12	13	15	16
	140	6	7	8	9	10
	120	3	4	5	5	6

Men — Diabetic — Non−smoker

Age	Systolic BP	3	4	5	6	7
70−74	180	48	52	57	61	66
	160	38	42	45	49	54
	140	29	32	35	39	43
	120	22	24	27	30	33
65−69	180	43	47	52	56	60
	160	33	36	40	43	47
	140	24	27	30	33	36
	120	17	19	22	24	27
60−64	180	41	45	49	53	57
	160	30	33	36	40	44
	140	21	24	26	29	32
	120	15	16	18	21	23
55−59	180	39	42	46	51	55
	160	27	30	33	37	40
	140	19	21	23	26	29
	120	13	14	16	18	20
50−54	180	35	39	42	46	50
	160	24	26	29	32	36
	140	16	18	20	22	24
	120	10	11	13	14	16
45−49	180	30	34	37	41	45
	160	20	22	25	27	30
	140	12	14	16	18	20
	120	8	9	10	11	12
40−44	180	23	26	29	32	35
	160	14	16	18	20	22
	140	9	10	11	12	14
	120	5	6	6	7	8

Men — Diabetic — Smoker

Age	Systolic BP	3	4	5	6	7
70−74	180	60	64	69	73	77
	160	48	53	57	61	66
	140	38	42	46	50	54
	120	29	32	36	39	43
65−69	180	55	59	63	68	72
	160	42	46	50	55	59
	140	32	35	39	42	46
	120	23	26	29	32	35
60−64	180	52	56	60	65	69
	160	39	43	47	51	55
	140	28	31	34	38	42
	120	20	22	25	27	30
55−59	180	49	54	58	62	67
	160	36	39	43	47	51
	140	25	28	31	34	37
	120	17	19	21	24	26
50−54	180	45	49	53	58	62
	160	32	35	38	42	46
	140	21	24	26	29	32
	120	14	16	17	19	22
45−49	180	40	43	47	52	56
	160	26	29	32	36	39
	140	17	19	21	24	26
	120	11	12	13	15	17
40−44	180	31	34	37	41	45
	160	19	22	24	27	30
	140	12	13	15	17	19
	120	7	8	9	10	11

Uruguay

Systolic Blood Pressur (mmHg) by Total cholteserol (mmol/L)

Women

Non−diabetic, Non−smoker

Age	Systolic BP	3	4	5	6	7
70−74	180	15	17	19	21	23
	160	11	12	14	15	17
	140	8	9	10	11	13
	120	6	6	7	8	9
65−69	180	12	14	15	17	19
	160	8	10	11	12	14
	140	6	7	7	8	10
	120	4	5	5	6	7
60−64	180	10	11	13	14	16
	160	7	8	9	10	11
	140	4	5	6	7	7
	120	3	3	4	4	5
55−59	180	8	9	10	12	13
	160	5	6	7	8	9
	140	3	4	4	5	6
	120	2	2	3	3	4
50−54	180	7	7	8	10	11
	160	4	5	5	6	7
	140	2	3	3	4	4
	120	1	2	2	2	3
45−49	180	4	5	6	7	7
	160	3	3	3	4	4
	140	1	2	2	2	3
	120	1	1	1	1	1
40−44	180	3	3	4	4	5
	160	2	2	2	3	3
	140	1	1	1	1	2
	120	0	0	0	1	1

Non−diabetic, Smoker

Age	Systolic BP	3	4	5	6	7
70−74	180	23	26	28	31	35
	160	17	19	21	24	26
	140	13	14	16	18	20
	120	9	10	12	13	15
65−69	180	19	21	23	26	29
	160	13	15	17	19	21
	140	9	11	12	14	15
	120	7	7	8	10	11
60−64	180	16	18	20	22	24
	160	11	12	14	15	17
	140	7	8	9	11	12
	120	5	6	6	7	8
55−59	180	13	15	16	18	21
	160	9	10	11	12	14
	140	6	6	7	8	9
	120	4	4	5	5	6
50−54	180	11	12	14	15	17
	160	7	8	9	10	11
	140	4	5	5	6	7
	120	2	3	3	4	4
45−49	180	7	8	9	11	12
	160	4	5	6	6	7
	140	3	3	3	4	4
	120	1	2	2	2	3
40−44	180	5	6	7	7	8
	160	3	3	4	4	5
	140	2	2	2	2	3
	120	1	1	1	1	1

Diabetic, Non−smoker

Age	Systolic BP	3	4	5	6	7
70−74	180	30	33	36	40	44
	160	23	25	28	31	34
	140	17	19	21	23	26
	120	12	14	15	17	19
65−69	180	25	27	30	33	37
	160	18	20	22	25	27
	140	13	14	16	18	20
	120	9	10	11	13	14
60−64	180	21	23	26	29	32
	160	14	16	18	20	23
	140	10	11	13	14	16
	120	7	8	9	10	11
55−59	180	17	19	22	24	27
	160	12	13	15	16	18
	140	8	9	10	11	12
	120	5	6	6	7	8
50−54	180	14	16	18	20	22
	160	9	10	12	13	15
	140	6	7	7	8	9
	120	4	4	5	5	6
45−49	180	10	11	13	14	16
	160	6	7	8	9	10
	140	4	4	5	5	6
	120	2	2	3	3	4
40−44	180	7	8	9	10	11
	160	4	5	5	6	7
	140	2	3	3	3	4
	120	1	1	2	2	2

Diabetic, Smoker

Age	Systolic BP	3	4	5	6	7
70−74	180	44	48	52	56	60
	160	34	37	41	45	49
	140	26	28	32	35	38
	120	19	21	24	27	29
65−69	180	37	40	44	48	52
	160	27	30	33	37	40
	140	20	22	25	27	30
	120	14	16	18	20	22
60−64	180	31	35	38	42	46
	160	22	25	28	31	34
	140	16	18	20	22	24
	120	11	12	14	15	17
55−59	180	27	30	33	36	40
	160	18	20	23	25	28
	140	12	14	15	17	19
	120	8	9	10	12	13
50−54	180	22	25	27	30	33
	160	15	16	18	20	23
	140	9	11	12	13	15
	120	6	7	8	9	10
45−49	180	16	18	20	22	24
	160	10	11	12	14	16
	140	6	7	8	9	10
	120	4	4	5	5	6
40−44	180	11	13	14	16	18
	160	7	7	8	10	11
	140	4	4	5	6	6
	120	2	2	3	3	4

Men

Non−diabetic, Non−smoker

Age	Systolic BP	3	4	5	6	7
70−74	180	21	23	25	28	31
	160	15	17	19	21	24
	140	11	12	14	16	18
	120	8	9	10	12	13
65−69	180	18	20	22	25	28
	160	13	14	16	18	20
	140	9	10	11	13	14
	120	6	7	8	9	10
60−64	180	16	17	20	22	24
	160	11	12	14	15	17
	140	7	8	9	10	12
	120	5	5	6	7	8
55−59	180	14	16	18	20	22
	160	9	10	12	13	15
	140	6	7	8	9	10
	120	4	4	5	6	6
50−54	180	12	14	15	17	19
	160	8	9	10	11	12
	140	5	5	6	7	8
	120	3	3	4	4	5
45−49	180	9	10	12	13	15
	160	6	6	7	8	9
	140	3	4	4	5	6
	120	2	2	2	3	3
40−44	180	6	7	8	9	10
	160	3	4	4	5	6
	140	2	2	2	3	3
	120	1	1	1	2	2

Non−diabetic, Smoker

Age	Systolic BP	3	4	5	6	7
70−74	180	27	30	34	37	41
	160	21	23	25	28	31
	140	15	17	19	21	24
	120	11	13	14	16	18
65−69	180	24	27	30	33	36
	160	17	19	22	24	27
	140	12	14	16	17	19
	120	9	10	11	12	14
60−64	180	21	24	26	29	32
	160	15	16	18	21	23
	140	10	11	13	14	16
	120	7	8	9	10	11
55−59	180	19	21	24	26	29
	160	13	14	16	18	20
	140	8	9	11	12	14
	120	5	6	7	8	9
50−54	180	17	19	21	23	26
	160	11	12	13	15	17
	140	7	8	9	10	11
	120	4	5	5	6	7
45−49	180	13	14	16	18	20
	160	8	9	10	11	13
	140	5	5	6	7	8
	120	3	3	4	4	5
40−44	180	8	9	11	12	13
	160	5	6	6	7	8
	140	3	3	4	4	5
	120	1	2	2	2	3

Diabetic, Non−smoker

Age	Systolic BP	3	4	5	6	7
70−74	180	36	40	44	48	52
	160	28	31	34	38	41
	140	21	23	26	29	32
	120	15	17	19	22	24
65−69	180	32	36	39	43	47
	160	24	26	29	32	36
	140	17	19	21	24	26
	120	12	14	15	17	19
60−64	180	29	32	35	38	42
	160	20	23	25	28	31
	140	14	16	18	20	22
	120	10	11	12	14	15
55−59	180	26	29	32	35	39
	160	18	20	22	25	27
	140	12	13	15	17	19
	120	8	9	10	11	13
50−54	180	23	25	28	31	34
	160	15	17	19	21	23
	140	10	11	12	14	15
	120	6	7	8	9	10
45−49	180	18	20	22	24	27
	160	11	12	14	16	17
	140	7	8	9	10	11
	120	4	5	5	6	7
40−44	180	12	13	15	17	19
	160	7	8	9	10	11
	140	4	5	5	6	7
	120	2	3	3	3	4

Diabetic, Smoker

Age	Systolic BP	3	4	5	6	7
70−74	180	47	51	55	60	64
	160	37	40	44	48	52
	140	28	31	34	38	41
	120	21	23	26	29	32
65−69	180	42	46	50	54	58
	160	31	35	38	42	46
	140	23	26	28	32	35
	120	17	19	21	23	26
60−64	180	37	41	45	49	53
	160	27	30	33	36	40
	140	19	21	24	26	29
	120	13	15	17	19	21
55−59	180	34	38	41	45	49
	160	24	26	29	32	36
	140	16	18	20	23	25
	120	11	12	14	15	17
50−54	180	30	33	37	40	44
	160	20	22	25	28	31
	140	13	15	16	18	21
	120	8	9	11	12	13
45−49	180	24	26	29	32	36
	160	15	17	19	21	24
	140	9	11	12	13	15
	120	6	6	7	8	9
40−44	180	16	18	20	22	25
	160	10	11	12	14	16
	140	6	7	7	8	9
	120	3	4	4	5	6

Total cholteserol (mmol/L)

Venezuela

Systolic Blood Pressur (mmHg) by Age; columns: Total cholteserol (mmol/L)

Women – Non−diabetic – Non−smoker

Age	SBP	3	4	5	6	7
70−74	180	24	26	29	32	36
	160	18	20	22	25	27
	140	13	15	16	18	21
	120	10	11	12	14	15
65−69	180	19	21	23	26	29
	160	14	15	17	19	21
	140	10	11	12	14	15
	120	7	8	9	10	11
60−64	180	15	17	19	21	24
	160	11	12	13	15	17
	140	7	8	9	10	11
	120	5	5	6	7	8
55−59	180	14	16	18	20	22
	160	9	11	12	14	15
	140	6	7	8	9	10
	120	4	4	5	6	7
50−54	180	14	16	18	20	22
	160	9	10	11	13	14
	140	6	6	7	8	9
	120	3	4	4	5	6
45−49	180	12	13	15	16	18
	160	7	8	9	10	11
	140	4	5	5	6	7
	120	2	3	3	4	4
40−44	180	9	10	11	12	14
	160	5	6	6	7	8
	140	3	3	4	4	5
	120	1	2	2	2	3

Women – Non−diabetic – Smoker

Age	SBP	3	4	5	6	7
70−74	180	36	39	43	47	51
	160	27	30	33	37	40
	140	20	23	25	28	31
	120	15	17	19	21	24
65−69	180	29	32	35	39	42
	160	21	23	26	29	32
	140	15	17	19	21	24
	120	11	12	14	15	17
60−64	180	24	26	29	32	36
	160	17	19	21	23	26
	140	11	13	14	16	18
	120	8	9	10	11	12
55−59	180	22	25	28	31	34
	160	15	17	19	21	23
	140	10	11	13	14	16
	120	6	7	8	9	11
50−54	180	22	24	27	30	33
	160	14	16	18	20	22
	140	9	10	12	13	15
	120	6	6	7	8	9
45−49	180	18	20	23	25	28
	160	11	13	14	16	18
	140	7	8	9	10	11
	120	4	5	5	6	7
40−44	180	14	15	17	19	21
	160	8	9	10	12	13
	140	5	5	6	7	8
	120	3	3	3	4	5

Women – Diabetic – Non−smoker

Age	SBP	3	4	5	6	7
70−74	180	45	49	53	57	62
	160	35	38	42	46	50
	140	27	30	33	36	40
	120	20	22	25	28	30
65−69	180	37	40	44	48	52
	160	27	30	33	37	41
	140	20	22	25	27	30
	120	14	16	18	20	22
60−64	180	31	34	37	41	45
	160	22	24	27	30	33
	140	15	17	19	21	24
	120	10	12	13	15	17
55−59	180	29	32	35	39	43
	160	20	22	25	27	30
	140	13	15	17	19	21
	120	9	10	11	13	14
50−54	180	28	31	35	38	42
	160	19	21	23	26	29
	140	12	14	15	17	19
	120	8	9	10	11	13
45−49	180	24	26	29	32	36
	160	15	17	19	21	24
	140	9	11	12	13	15
	120	6	6	7	8	9
40−44	180	18	20	22	25	28
	160	11	12	14	15	17
	140	6	7	8	9	11
	120	4	4	5	5	6

Women – Diabetic – Smoker

Age	SBP	3	4	5	6	7
70−74	180	62	66	70	75	79
	160	50	54	59	63	67
	140	39	43	47	51	56
	120	30	33	37	41	44
65−69	180	52	56	61	65	70
	160	40	44	48	52	57
	140	30	33	37	40	44
	120	22	25	27	30	34
60−64	180	45	49	53	57	62
	160	33	36	40	44	48
	140	23	26	29	32	35
	120	16	18	21	23	25
55−59	180	42	46	50	55	59
	160	30	33	37	40	44
	140	21	23	26	29	32
	120	14	16	18	20	22
50−54	180	42	45	50	54	58
	160	29	32	35	39	42
	140	19	21	24	26	29
	120	12	14	16	18	20
45−49	180	35	39	43	47	51
	160	23	26	29	32	35
	140	15	17	19	21	23
	120	9	10	12	13	15
40−44	180	27	30	34	37	41
	160	17	19	21	24	27
	140	10	12	13	15	17
	120	6	7	8	9	10

Men – Non−diabetic – Non−smoker

Age	SBP	3	4	5	6	7
70−74	180	31	35	38	42	46
	160	24	26	29	32	36
	140	18	20	22	25	27
	120	13	15	16	18	21
65−69	180	25	28	31	34	37
	160	18	20	22	25	28
	140	13	14	16	18	20
	120	9	10	11	13	14
60−64	180	22	24	27	30	33
	160	15	17	19	21	24
	140	10	12	13	15	16
	120	7	8	9	10	11
55−59	180	22	25	28	31	34
	160	15	17	19	21	24
	140	10	11	13	14	16
	120	7	7	8	9	11
50−54	180	21	24	26	29	32
	160	14	16	17	19	22
	140	9	10	11	13	14
	120	6	6	7	8	9
45−49	180	17	19	22	24	27
	160	11	12	14	15	17
	140	7	8	9	10	11
	120	4	5	5	6	7
40−44	180	12	13	15	16	18
	160	7	8	9	10	11
	140	4	5	5	6	7
	120	2	3	3	3	4

Men – Non−diabetic – Smoker

Age	SBP	3	4	5	6	7
70−74	180	41	45	49	53	57
	160	31	35	38	42	46
	140	24	27	29	33	36
	120	18	20	22	25	27
65−69	180	33	36	40	44	48
	160	24	27	30	33	36
	140	18	20	22	24	27
	120	13	14	16	18	20
60−64	180	29	32	35	39	43
	160	20	23	25	28	31
	140	14	16	18	20	22
	120	10	11	12	14	16
55−59	180	30	33	36	40	44
	160	20	23	25	28	31
	140	14	15	17	19	22
	120	9	10	12	13	15
50−54	180	28	31	35	38	42
	160	19	21	23	26	29
	140	12	14	15	17	19
	120	8	9	10	11	13
45−49	180	23	26	29	32	35
	160	15	17	19	21	23
	140	9	10	12	13	15
	120	6	6	7	8	9
40−44	180	16	18	20	22	25
	160	10	11	12	14	15
	140	6	6	7	8	9
	120	3	4	4	5	5

Men – Diabetic – Non−smoker

Age	SBP	3	4	5	6	7
70−74	180	52	57	61	65	70
	160	41	45	49	54	58
	140	32	35	39	43	47
	120	24	27	30	33	36
65−69	180	43	47	51	55	60
	160	32	36	39	43	47
	140	24	27	29	33	36
	120	17	19	22	24	27
60−64	180	38	42	46	50	54
	160	28	31	34	37	41
	140	20	22	24	27	30
	120	14	15	17	19	21
55−59	180	39	43	47	51	55
	160	28	31	34	37	41
	140	19	21	24	26	29
	120	13	14	16	18	20
50−54	180	38	41	45	49	53
	160	26	28	32	35	38
	140	17	19	21	24	26
	120	11	12	14	16	17
45−49	180	31	35	38	42	46
	160	21	23	25	28	31
	140	13	15	16	18	20
	120	8	9	10	12	13
40−44	180	22	24	27	30	33
	160	13	15	17	19	21
	140	8	9	10	12	13
	120	5	5	6	7	8

Men – Diabetic – Smoker

Age	SBP	3	4	5	6	7
70−74	180	64	69	73	77	81
	160	52	57	61	66	70
	140	42	45	50	54	58
	120	32	36	39	43	47
65−69	180	54	58	63	67	72
	160	42	46	50	54	59
	140	32	35	38	42	46
	120	23	26	29	32	35
60−64	180	49	53	57	62	66
	160	36	40	44	48	52
	140	26	29	32	35	39
	120	18	21	23	26	28
55−59	180	50	54	59	63	67
	160	36	40	44	48	52
	140	25	28	31	35	38
	120	17	19	22	24	27
50−54	180	48	52	57	61	65
	160	34	37	41	45	49
	140	23	25	28	31	35
	120	15	17	19	21	23
45−49	180	41	45	49	53	57
	160	27	30	34	37	41
	140	18	20	22	25	27
	120	11	13	14	16	18
40−44	180	29	32	36	39	43
	160	18	20	23	25	28
	140	11	13	14	16	18
	120	7	7	8	10	11

Total cholteserol (mmol/L)
