# Supplementary material for: Derivation, internal validation, and recalibration of a cardiovascular risk score for Latin America and the Caribbean (Globorisk-LAC): A pooled analysis of cohort studies
Source: Lancet Reg Health Am. 2022 Apr 23;9:100258. doi: 10.1016/j.lana.2022.100258 (PMC9107390; doi:10.1016/j.lana.2022.100258)
Supplement: Supplementary file 3 [file mmc3.pdf]

Antigua and Barbuda

Systolic Blood Pressur (mmHg) by Body Mass Index (kg/m2)

Women

Age	Systolic Blood Pressur (mmHg)	Non−smoker 20	Non−smoker 25	Non−smoker 30	Non−smoker 35	Non−smoker 40	Smoker 20	Smoker 25	Smoker 30	Smoker 35	Smoker 40
70−74	180	16	20	25	31	38	25	31	38	46	55
	160	12	15	18	23	29	18	23	29	35	43
	140	8	10	13	17	21	13	17	21	26	32
	120	6	7	9	12	15	9	12	15	19	24
65−69	180	12	15	19	24	30	19	24	29	36	44
	160	8	10	13	17	21	13	17	21	26	32
	140	5	7	9	12	15	9	12	15	19	23
	120	4	5	6	8	10	6	8	10	13	16
60−64	180	10	13	17	21	26	17	21	26	32	39
	160	7	9	11	14	18	11	14	18	22	28
	140	4	6	7	9	12	7	9	12	15	19
	120	3	4	5	6	8	5	6	8	10	13
55−59	180	11	14	17	21	27	17	21	27	33	40
	160	7	9	11	14	18	11	14	18	22	28
	140	4	5	7	9	12	7	9	11	15	18
	120	2	3	4	6	7	4	6	7	9	12
50−54	180	11	14	17	22	27	17	22	27	33	40
	160	7	8	11	14	17	11	14	17	22	27
	140	4	5	7	8	11	7	8	11	14	17
	120	2	3	4	5	7	4	5	7	8	11
45−49	180	11	14	17	22	27	17	22	27	33	41
	160	6	8	11	13	17	11	13	17	21	27
	140	4	5	6	8	10	6	8	10	13	17
	120	2	3	4	5	6	4	5	6	8	10
40−44	180	11	14	18	22	28	18	22	28	34	42
	160	6	8	11	13	17	11	13	17	21	27
	140	4	5	6	8	10	6	8	10	13	16
	120	2	2	3	4	6	3	4	6	7	9

Men

Age	Systolic Blood Pressur (mmHg)	Non−smoker 20	Non−smoker 25	Non−smoker 30	Non−smoker 35	Non−smoker 40	Smoker 20	Smoker 25	Smoker 30	Smoker 35	Smoker 40
70−74	180	25	31	38	45	54	32	39	47	56	65
	160	18	23	29	35	43	24	30	37	45	53
	140	13	17	21	27	33	18	23	28	34	42
	120	10	12	16	20	25	13	17	21	26	32
65−69	180	19	24	30	36	44	25	31	38	46	55
	160	14	17	22	27	33	18	23	28	35	42
	140	9	12	15	19	24	13	16	20	25	31
	120	6	8	11	14	17	9	11	14	18	23
60−64	180	16	20	26	32	39	22	27	33	40	49
	160	11	14	18	22	28	15	19	23	29	36
	140	7	9	12	15	19	10	13	16	20	25
	120	5	6	8	10	13	7	9	11	14	18
55−59	180	15	19	24	30	36	20	25	31	38	46
	160	10	13	16	20	25	13	17	21	26	32
	140	6	8	10	13	17	9	11	14	18	22
	120	4	5	7	9	11	5	7	9	12	15
50−54	180	13	17	21	27	33	18	23	28	34	42
	160	8	11	14	17	22	11	15	18	23	28
	140	5	7	9	11	14	7	9	12	15	19
	120	3	4	5	7	9	4	6	7	9	12
45−49	180	11	14	17	22	27	15	18	23	29	35
	160	6	8	11	14	17	9	11	14	18	23
	140	4	5	6	8	11	5	7	9	11	14
	120	2	3	4	5	6	3	4	5	7	9
40−44	180	7	9	11	14	18	9	12	15	19	24
	160	4	5	6	8	11	5	7	9	11	14
	140	2	3	4	5	6	3	4	5	7	8
	120	1	1	2	3	3	2	2	3	4	5

Body Mass Index (kg/m2)

Argentina

Systolic Blood Pressur (mmHg) by Body Mass Index (kg/m2)

Women

Non−smoker

Age	Systolic Blood Pressur (mmHg)	20	25	30	35	40
70−74	180	23	29	35	43	51
	160	17	21	26	33	40
	140	12	15	19	24	30
	120	9	11	14	18	22
65−69	180	17	21	27	33	40
	160	12	15	19	24	29
	140	8	10	13	17	21
	120	5	7	9	12	15
60−64	180	13	16	20	25	31
	160	8	11	14	17	22
	140	5	7	9	12	15
	120	3	5	6	8	10
55−59	180	10	13	16	21	26
	160	6	8	11	13	17
	140	4	5	7	9	11
	120	2	3	4	5	7
50−54	180	8	10	13	17	21
	160	5	6	8	11	13
	140	3	4	5	6	8
	120	2	2	3	4	5
45−49	180	7	9	11	14	18
	160	4	5	7	9	11
	140	2	3	4	5	6
	120	1	2	2	3	4
40−44	180	6	7	10	12	16
	160	3	4	5	7	9
	140	2	2	3	4	5
	120	1	1	1	2	3

Smoker

Age	Systolic Blood Pressur (mmHg)	20	25	30	35	40
70−74	180	35	43	51	60	69
	160	26	33	40	48	56
	140	19	24	30	37	44
	120	14	18	22	28	34
65−69	180	26	33	40	48	57
	160	19	24	29	36	43
	140	13	17	21	26	32
	120	9	12	15	18	23
60−64	180	20	25	31	38	46
	160	14	17	22	27	33
	140	9	12	15	19	23
	120	6	8	10	12	16
55−59	180	16	20	26	32	39
	160	11	13	17	21	26
	140	7	9	11	14	18
	120	4	5	7	9	11
50−54	180	13	17	21	26	32
	160	8	11	13	17	21
	140	5	6	8	11	14
	120	3	4	5	6	8
45−49	180	11	14	18	23	28
	160	7	9	11	14	18
	140	4	5	6	8	11
	120	2	3	4	5	6
40−44	180	10	12	16	20	24
	160	5	7	9	11	15
	140	3	4	5	7	8
	120	1	2	3	4	5

Men

Non−smoker

Age	Systolic Blood Pressur (mmHg)	20	25	30	35	40
70−74	180	20	25	31	38	45
	160	15	18	23	29	35
	140	11	13	17	21	26
	120	8	10	12	16	20
65−69	180	17	22	27	34	41
	160	12	16	20	24	30
	140	8	11	14	17	22
	120	6	7	10	12	16
60−64	180	17	21	26	32	40
	160	11	14	18	23	28
	140	8	10	12	16	20
	120	5	6	8	11	14
55−59	180	16	20	25	31	37
	160	10	13	17	21	26
	140	7	8	11	14	17
	120	4	5	7	9	11
50−54	180	14	17	22	27	34
	160	9	11	14	18	22
	140	5	7	9	11	14
	120	3	4	5	7	9
45−49	180	11	14	18	23	28
	160	7	9	11	14	18
	140	4	5	7	9	11
	120	2	3	4	5	7
40−44	180	8	10	13	17	21
	160	5	6	8	10	12
	140	2	3	4	6	7
	120	1	2	2	3	4

Smoker

Age	Systolic Blood Pressur (mmHg)	20	25	30	35	40
70−74	180	26	32	39	47	56
	160	19	24	30	37	45
	140	14	18	22	28	34
	120	10	13	17	21	26
65−69	180	23	29	35	43	51
	160	16	21	26	32	39
	140	12	15	18	23	29
	120	8	10	13	16	21
60−64	180	22	28	34	41	50
	160	15	19	24	30	37
	140	10	13	17	21	26
	120	7	9	11	14	18
55−59	180	21	26	32	39	47
	160	14	18	22	27	34
	140	9	12	15	18	23
	120	6	7	10	12	15
50−54	180	18	23	29	35	43
	160	12	15	19	24	29
	140	7	9	12	15	19
	120	4	6	7	10	12
45−49	180	15	19	24	29	36
	160	9	12	15	19	24
	140	5	7	9	12	15
	120	3	4	5	7	9
40−44	180	11	14	17	22	27
	160	6	8	10	13	17
	140	3	5	6	8	10
	120	2	2	3	4	6

Body Mass Index (kg/m2)

Barbados

Systolic Blood Pressur (mmHg) by Body Mass Index (kg/m2)

Women — Non−smoker

Age	SBP	20	25	30	35	40
70−74	180	17	21	26	32	39
	160	12	15	19	24	30
	140	8	11	14	17	22
	120	6	8	10	13	16
65−69	180	12	15	19	24	29
	160	8	10	13	17	21
	140	5	7	9	12	15
	120	4	5	6	8	10
60−64	180	10	12	16	20	25
	160	6	8	10	13	17
	140	4	5	7	9	11
	120	3	3	4	6	7
55−59	180	7	9	11	15	18
	160	4	6	7	9	12
	140	3	3	5	6	8
	120	1	2	3	4	5
50−54	180	5	6	8	10	13
	160	3	4	5	6	8
	140	1	2	3	4	5
	120	1	1	1	2	3
45−49	180	3	3	5	6	8
	160	1	2	2	3	4
	140	1	1	1	2	2
	120	0	0	1	1	1
40−44	180	1	2	3	3	4
	160	1	1	1	2	2
	140	0	0	1	1	1
	120	0	0	0	0	0

Women — Smoker

Age	SBP	20	25	30	35	40
70−74	180	26	32	39	47	56
	160	19	24	30	36	44
	140	14	17	22	27	33
	120	10	13	16	20	25
65−69	180	19	24	29	36	44
	160	13	17	21	26	32
	140	9	12	15	19	23
	120	6	8	10	13	16
60−64	180	16	20	25	31	37
	160	10	13	17	21	26
	140	7	9	11	14	18
	120	4	6	7	10	12
55−59	180	11	15	18	23	28
	160	7	9	12	15	19
	140	5	6	8	10	12
	120	3	4	5	6	8
50−54	180	8	10	13	16	20
	160	5	6	8	10	13
	140	3	4	5	6	8
	120	1	2	3	4	5
45−49	180	4	6	8	10	12
	160	2	3	4	6	7
	140	1	2	2	3	4
	120	1	1	1	2	2
40−44	180	3	3	4	6	7
	160	1	2	2	3	4
	140	1	1	1	2	2
	120	0	0	0	1	1

Men — Non−smoker

Age	SBP	20	25	30	35	40
70−74	180	24	30	36	44	52
	160	18	22	28	34	41
	140	13	16	20	25	31
	120	9	12	15	19	24
65−69	180	20	25	31	38	46
	160	14	18	23	28	35
	140	10	13	16	20	25
	120	7	9	11	14	18
60−64	180	16	21	26	32	39
	160	11	14	18	22	28
	140	7	9	12	15	19
	120	5	6	8	10	13
55−59	180	12	15	19	24	30
	160	8	10	13	16	20
	140	5	6	8	11	14
	120	3	4	5	7	9
50−54	180	10	13	17	21	26
	160	6	8	11	13	17
	140	4	5	7	8	11
	120	2	3	4	5	7
45−49	180	9	11	14	18	23
	160	5	7	9	11	14
	140	3	4	5	7	9
	120	2	2	3	4	5
40−44	180	5	6	8	10	13
	160	3	3	4	6	8
	140	1	2	2	3	4
	120	1	1	1	2	2

Men — Smoker

Age	SBP	20	25	30	35	40
70−74	180	31	38	46	55	64
	160	23	29	36	43	51
	140	17	22	27	33	40
	120	12	16	20	25	31
65−69	180	27	33	40	48	57
	160	19	24	30	37	44
	140	14	17	22	27	33
	120	9	12	15	19	24
60−64	180	22	27	33	41	49
	160	15	19	24	29	36
	140	10	13	16	20	25
	120	7	9	11	14	18
55−59	180	16	21	26	32	39
	160	11	14	17	22	27
	140	7	9	11	14	18
	120	4	6	7	9	12
50−54	180	14	18	22	28	34
	160	9	11	14	18	23
	140	5	7	9	11	15
	120	3	4	5	7	9
45−49	180	12	15	19	24	30
	160	7	9	12	15	19
	140	4	6	7	9	12
	120	2	3	4	5	7
40−44	180	6	8	11	14	17
	160	4	5	6	8	10
	140	2	3	3	5	6
	120	1	1	2	2	3

Belize

Systolic Blood Pressur (mmHg) by Body Mass Index (kg/m2)

Age 70−74

Systolic Blood Pressur (mmHg)	Women Non−smoker 20	25	30	35	40	Women Smoker 20	25	30	35	40	Men Non−smoker 20	25	30	35	40	Men Smoker 20	25	30	35	40
180	24	30	36	44	52	36	44	52	61	70	34	42	50	59	68	44	52	61	70	79
160	17	22	27	33	41	27	33	41	49	58	26	32	39	47	56	33	41	49	58	67
140	13	16	20	25	31	20	25	31	38	45	19	24	30	36	44	25	31	38	46	54
120	9	11	14	18	23	14	18	23	28	35	14	18	22	27	34	19	23	29	35	43

Age 65−69

Systolic Blood Pressur (mmHg)	Women Non−smoker 20	25	30	35	40	Women Smoker 20	25	30	35	40	Men Non−smoker 20	25	30	35	40	Men Smoker 20	25	30	35	40
180	17	22	27	33	40	27	33	40	48	57	29	36	43	52	61	38	45	54	63	72
160	12	15	19	24	29	19	24	29	36	44	21	26	32	39	47	28	34	41	50	58
140	8	10	13	17	21	13	17	21	26	32	15	19	23	29	36	20	25	31	38	45
120	5	7	9	12	15	9	11	15	18	23	10	13	17	21	26	14	18	22	28	34

Age 60−64

Systolic Blood Pressur (mmHg)	Women Non−smoker 20	25	30	35	40	Women Smoker 20	25	30	35	40	Men Non−smoker 20	25	30	35	40	Men Smoker 20	25	30	35	40
180	13	16	21	26	32	21	26	32	39	47	26	32	39	47	56	34	41	49	58	67
160	9	11	14	18	22	14	18	22	27	34	18	23	28	35	42	24	30	36	44	53
140	6	7	9	12	15	9	12	15	19	24	12	16	20	25	30	17	21	26	32	39
120	4	5	6	8	10	6	8	10	13	16	8	11	13	17	21	11	14	18	23	28

Age 55−59

Systolic Blood Pressur (mmHg)	Women Non−smoker 20	25	30	35	40	Women Smoker 20	25	30	35	40	Men Non−smoker 20	25	30	35	40	Men Smoker 20	25	30	35	40
180	11	14	17	22	27	17	22	27	33	40	20	25	31	38	46	27	33	40	48	57
160	7	9	11	14	18	11	14	18	22	28	13	17	21	26	33	18	22	28	34	42
140	4	5	7	9	12	7	9	12	15	19	9	11	14	18	22	12	15	19	24	29
120	3	3	4	6	7	4	6	7	9	12	6	7	9	12	15	8	10	12	16	20

Age 50−54

Systolic Blood Pressur (mmHg)	Women Non−smoker 20	25	30	35	40	Women Smoker 20	25	30	35	40	Men Non−smoker 20	25	30	35	40	Men Smoker 20	25	30	35	40
180	9	11	15	18	23	14	18	23	28	35	15	19	24	30	37	20	25	31	38	46
160	5	7	9	11	15	9	11	15	18	23	10	12	16	20	24	13	16	21	26	32
140	3	4	5	7	9	5	7	9	11	15	6	8	10	12	16	8	10	13	17	21
120	2	2	3	4	5	3	4	5	7	9	4	5	6	8	10	5	6	8	11	13

Age 45−49

Systolic Blood Pressur (mmHg)	Women Non−smoker 20	25	30	35	40	Women Smoker 20	25	30	35	40	Men Non−smoker 20	25	30	35	40	Men Smoker 20	25	30	35	40
180	8	10	13	16	20	13	16	20	25	31	11	14	17	22	27	15	18	23	29	35
160	4	6	7	10	12	7	10	12	15	19	6	8	11	14	17	9	11	14	18	23
140	2	3	4	6	7	4	6	7	9	12	4	5	6	8	11	5	7	9	11	14
120	1	2	2	3	4	2	3	4	5	7	2	3	4	5	6	3	4	5	7	9

Age 40−44

Systolic Blood Pressur (mmHg)	Women Non−smoker 20	25	30	35	40	Women Smoker 20	25	30	35	40	Men Non−smoker 20	25	30	35	40	Men Smoker 20	25	30	35	40
180	5	7	9	11	14	9	11	14	18	22	6	8	10	13	16	8	11	13	17	21
160	3	4	5	6	8	5	6	8	10	13	3	4	6	7	9	5	6	8	10	13
140	1	2	3	4	5	3	3	5	6	8	2	2	3	4	5	3	3	4	6	7
120	1	1	1	2	2	1	2	2	3	4	1	1	2	2	3	1	2	2	3	4

Body Mass Index (kg/m2)

Bolivia

Systolic Blood Pressur (mmHg) by Body Mass Index (kg/m2)

Age	SBP	Women Non−smoker 20	25	30	35	40	Women Smoker 20	25	30	35	40	Men Non−smoker 20	25	30	35	40	Men Smoker 20	25	30	35	40
70−74	180	25	30	37	45	53	37	45	53	62	71	25	31	38	46	55	33	40	48	57	66
	160	18	22	28	34	42	28	34	42	50	59	19	24	29	36	43	25	31	38	45	54
	140	13	16	20	26	32	20	25	31	38	46	14	17	22	27	33	18	23	29	35	43
	120	9	12	15	19	23	15	19	23	29	36	10	13	16	20	25	13	17	21	26	33
65−69	180	20	25	31	38	46	31	38	46	54	63	22	27	33	41	49	29	35	43	51	60
	160	14	18	22	27	34	22	27	34	41	49	16	20	24	30	37	21	26	32	39	47
	140	10	12	15	20	24	15	19	24	30	37	11	14	17	22	27	15	18	23	29	35
	120	6	8	11	14	17	11	14	17	22	27	7	10	12	16	20	10	13	16	21	26
60−64	180	19	24	29	36	44	29	36	44	52	61	21	26	32	39	47	27	34	41	49	58
	160	13	16	20	25	31	20	25	31	38	46	14	18	23	28	35	19	24	30	36	44
	140	8	11	14	17	22	14	17	22	27	33	10	12	16	20	24	13	16	21	26	32
	120	5	7	9	12	15	9	12	15	18	23	6	8	10	13	17	9	11	14	18	22
55−59	180	19	24	29	36	44	29	36	43	52	61	21	26	32	39	47	27	34	41	49	58
	160	12	16	20	24	30	20	24	30	37	45	14	17	22	27	33	18	23	29	35	43
	140	8	10	13	16	20	13	16	20	25	31	9	11	14	18	23	12	15	19	24	30
	120	5	6	8	10	13	8	10	13	17	21	6	7	9	12	15	8	10	13	16	20
50−54	180	18	23	28	35	42	28	35	42	51	59	20	25	31	38	46	26	32	40	48	56
	160	11	15	18	23	29	18	23	28	35	42	13	16	20	25	31	17	21	27	33	40
	140	7	9	12	15	18	11	15	18	23	29	8	10	13	16	21	11	14	17	22	27
	120	4	5	7	9	12	7	9	12	15	18	5	6	8	10	13	7	9	11	14	18
45−49	180	17	21	26	33	40	26	32	40	48	56	17	22	27	33	41	23	29	35	43	51
	160	10	13	16	21	26	16	21	26	32	39	11	14	17	22	27	14	18	23	28	35
	140	6	8	10	13	16	10	13	16	20	25	6	8	11	13	17	9	11	14	18	22
	120	3	4	6	8	10	6	7	10	12	16	4	5	6	8	10	5	7	9	11	14
40−44	180	13	17	21	26	32	21	26	32	39	47	14	18	23	28	35	19	24	30	36	44
	160	8	10	12	16	20	12	16	20	25	31	8	11	14	17	22	11	15	18	23	28
	140	4	6	7	9	12	7	9	12	15	19	5	6	8	10	13	7	9	11	14	18
	120	2	3	4	5	7	4	5	7	9	11	3	3	5	6	8	4	5	6	8	10

Brazil

Systolic Blood Pressur (mmHg) by Body Mass Index (kg/m2)

Women

Non−smoker

Age	Systolic Blood Pressur (mmHg)	20	25	30	35	40
70−74	180	16	20	25	31	37
	160	11	14	18	23	28
	140	8	10	13	16	21
	120	6	7	9	12	15
65−69	180	13	17	21	26	32
	160	9	11	15	18	23
	140	6	8	10	13	16
	120	4	5	7	9	11
60−64	180	11	14	18	22	28
	160	7	9	12	15	19
	140	5	6	8	10	13
	120	3	4	5	7	9
55−59	180	10	12	16	20	25
	160	6	8	10	13	16
	140	4	5	6	8	11
	120	2	3	4	5	7
50−54	180	9	11	14	18	22
	160	5	7	9	11	14
	140	3	4	5	7	9
	120	2	2	3	4	5
45−49	180	7	9	12	15	19
	160	4	6	7	9	12
	140	2	3	4	5	7
	120	1	2	2	3	4
40−44	180	6	8	10	13	16
	160	3	4	6	7	9
	140	2	2	3	4	5
	120	1	1	2	2	3

Smoker

Age	Systolic Blood Pressur (mmHg)	20	25	30	35	40
70−74	180	25	31	37	45	54
	160	18	23	28	34	42
	140	13	16	21	26	32
	120	9	12	15	19	23
65−69	180	21	26	32	39	47
	160	15	18	23	28	35
	140	10	13	16	20	25
	120	7	9	11	14	18
60−64	180	18	22	28	34	42
	160	12	15	19	24	30
	140	8	10	13	16	20
	120	5	7	8	11	14
55−59	180	16	20	25	31	38
	160	10	13	16	21	26
	140	6	8	11	13	17
	120	4	5	7	9	11
50−54	180	14	18	22	28	34
	160	9	11	14	18	23
	140	5	7	9	11	14
	120	3	4	5	7	9
45−49	180	12	15	19	24	30
	160	7	9	12	15	19
	140	4	5	7	9	11
	120	2	3	4	5	7
40−44	180	10	12	16	20	25
	160	6	7	9	12	15
	140	3	4	5	7	9
	120	2	2	3	4	5

Men

Non−smoker

Age	Systolic Blood Pressur (mmHg)	20	25	30	35	40
70−74	180	19	24	30	37	44
	160	14	18	22	28	34
	140	10	13	16	21	26
	120	7	9	12	15	19
65−69	180	17	22	27	33	40
	160	12	15	19	24	30
	140	8	11	14	17	22
	120	6	7	9	12	15
60−64	180	15	19	24	30	37
	160	10	13	17	21	26
	140	7	9	11	14	18
	120	5	6	8	10	12
55−59	180	14	18	23	28	35
	160	9	12	15	19	24
	140	6	8	10	13	16
	120	4	5	6	8	10
50−54	180	13	17	21	27	33
	160	8	11	14	17	22
	140	5	7	9	11	14
	120	3	4	5	7	9
45−49	180	11	14	18	23	28
	160	7	9	11	14	18
	140	4	5	7	9	11
	120	2	3	4	5	7
40−44	180	9	11	14	18	22
	160	5	6	8	11	13
	140	3	4	5	6	8
	120	1	2	3	3	4

Smoker

Age	Systolic Blood Pressur (mmHg)	20	25	30	35	40
70−74	180	26	32	39	46	55
	160	19	24	29	36	44
	140	14	17	22	27	33
	120	10	13	16	20	25
65−69	180	23	28	35	42	50
	160	16	20	25	31	38
	140	11	14	18	23	28
	120	8	10	13	16	20
60−64	180	21	26	32	39	47
	160	14	18	22	28	34
	140	9	12	15	19	24
	120	6	8	10	13	17
55−59	180	19	24	30	37	44
	160	13	16	20	25	31
	140	8	11	13	17	21
	120	5	7	9	11	14
50−54	180	18	23	28	35	42
	160	11	15	18	23	29
	140	7	9	12	15	19
	120	4	6	7	9	12
45−49	180	15	19	24	30	37
	160	9	12	15	19	24
	140	6	7	9	12	15
	120	3	4	5	7	9
40−44	180	12	15	19	23	29
	160	7	9	11	14	18
	140	4	5	6	8	11
	120	2	3	4	5	6

Body Mass Index (kg/m2)

Chile

Systolic Blood Pressur (mmHg) by Body Mass Index (kg/m2)

Age	SBP	Women Non−smoker 20	25	30	35	40	Women Smoker 20	25	30	35	40	Men Non−smoker 20	25	30	35	40	Men Smoker 20	25	30	35	40
70−74	180	12	15	19	23	29	19	23	29	35	43	12	16	20	25	30	17	21	26	32	39
	160	8	11	13	17	21	13	17	21	26	33	9	11	14	18	23	12	15	19	24	30
	140	6	7	10	12	15	9	12	15	19	24	6	8	10	13	17	9	11	14	18	22
	120	4	5	7	9	11	7	9	11	14	18	4	6	7	10	12	6	8	10	13	16
65−69	180	8	11	14	17	22	14	17	22	27	33	11	14	17	22	27	14	18	23	28	35
	160	6	7	9	12	15	9	12	15	19	24	7	9	12	15	19	10	13	16	20	25
	140	4	5	6	8	10	6	8	10	13	17	5	6	8	11	14	7	9	11	14	18
	120	2	3	4	6	7	4	5	7	9	12	3	4	6	7	9	5	6	8	10	13
60−64	180	6	8	10	12	16	10	12	16	20	25	10	12	16	20	25	13	17	21	26	32
	160	4	5	6	8	11	6	8	11	13	17	6	8	11	13	17	9	11	14	18	22
	140	2	3	4	5	7	4	5	7	9	11	4	5	7	9	11	6	7	10	12	15
	120	1	2	3	3	4	3	3	4	6	7	3	3	5	6	8	4	5	6	8	10
55−59	180	5	6	8	10	13	8	10	12	16	20	9	11	14	18	22	12	15	19	24	29
	160	3	4	5	6	8	5	6	8	10	13	5	7	9	12	15	8	10	12	16	20
	140	2	2	3	4	5	3	4	5	6	8	3	4	6	8	10	5	6	8	10	13
	120	1	1	2	2	3	2	2	3	4	5	2	3	4	5	6	3	4	5	7	8
50−54	180	4	5	7	8	11	7	8	11	14	17	8	10	13	16	20	11	14	17	22	27
	160	2	3	4	5	7	4	5	7	8	11	5	6	8	10	13	7	9	11	14	18
	140	1	2	2	3	4	2	3	4	5	7	3	4	5	6	8	4	5	7	9	11
	120	1	1	1	2	2	1	2	2	3	4	2	2	3	4	5	2	3	4	5	7
45−49	180	3	4	5	7	9	5	7	9	11	15	7	9	12	15	19	10	13	16	20	25
	160	2	2	3	4	5	3	4	5	7	9	4	6	7	9	12	6	8	10	12	16
	140	1	1	2	2	3	2	2	3	4	5	2	3	4	5	7	3	4	6	7	10
	120	0	0	1	1	2	1	1	2	2	3	1	2	2	3	4	2	3	3	4	6
40−44	180	2	3	4	5	7	4	5	7	9	11	5	7	9	11	14	7	9	12	15	19
	160	1	2	2	3	4	2	3	4	5	7	3	4	5	7	8	4	5	7	9	11
	140	0	1	1	1	2	1	1	2	3	4	1	2	3	4	5	2	3	4	5	7
	120	0	0	0	1	1	0	1	1	1	2	1	1	1	2	3	1	2	2	3	4

Colombia

Systolic Blood Pressur (mmHg) by Body Mass Index (kg/m2), Women / Men, Non−smoker / Smoker, by Age

Age	Systolic Blood Pressur (mmHg)	Women Non−smoker					Women Smoker					Men Non−smoker					Men Smoker				
	Body Mass Index (kg/m2)	20	25	30	35	40	20	25	30	35	40	20	25	30	35	40	20	25	30	35	40
70−74	180	26	32	39	46	55	38	46	55	64	73	28	35	42	50	59	36	44	53	62	71
	160	19	23	29	36	43	29	35	43	51	60	21	26	32	39	47	28	34	41	49	58
	140	13	17	21	27	33	21	26	33	40	48	15	19	24	30	37	20	26	32	39	46
	120	10	12	15	19	24	15	19	24	30	37	11	14	18	22	28	15	19	24	29	36
65−69	180	21	26	32	39	47	32	39	47	56	65	27	33	40	48	57	35	42	50	59	68
	160	15	19	23	29	35	23	29	35	43	51	19	24	30	36	44	25	31	38	46	55
	140	10	13	16	21	26	16	21	26	32	39	14	17	21	27	33	18	23	28	35	42
	120	7	9	11	14	18	11	14	18	23	28	9	12	15	19	24	13	16	20	25	31
60−64	180	17	21	26	32	40	26	32	39	48	56	25	31	38	45	54	32	39	47	56	65
	160	11	14	18	23	28	18	23	28	34	42	17	22	27	33	40	23	28	35	42	51
	140	7	10	12	15	19	12	15	19	24	30	12	15	19	23	29	16	20	25	31	37
	120	5	6	8	10	13	8	10	13	17	21	8	10	13	16	20	11	14	17	21	27
55−59	180	14	18	22	27	34	22	27	34	41	49	22	28	34	41	50	29	36	43	52	61
	160	9	11	14	18	23	14	18	23	28	35	15	19	23	29	36	20	25	31	37	45
	140	6	7	9	12	15	9	12	15	19	24	10	12	16	20	25	13	17	21	26	32
	120	3	4	6	8	10	6	8	10	12	16	6	8	10	13	16	8	11	14	17	22
50−54	180	12	16	20	25	30	20	25	30	37	45	20	25	31	37	45	26	32	39	47	56
	160	8	10	12	16	20	12	16	20	25	31	13	16	20	25	31	17	21	26	33	40
	140	5	6	8	10	13	8	10	12	16	20	8	10	13	16	21	11	14	17	22	27
	120	3	3	5	6	8	5	6	8	10	13	5	6	8	10	13	7	9	11	14	18
45−49	180	11	13	17	21	27	17	21	27	33	40	16	21	26	32	39	22	27	33	41	49
	160	6	8	10	13	17	10	13	17	21	26	10	13	16	20	25	14	17	22	27	33
	140	4	5	6	8	10	6	8	10	13	16	6	8	10	13	16	8	11	13	17	21
	120	2	3	3	5	6	3	5	6	8	10	3	5	6	8	10	5	6	8	10	13
40−44	180	8	10	13	17	21	13	17	21	26	32	12	15	19	23	29	16	20	24	30	37
	160	4	6	8	10	12	8	10	12	16	20	7	9	11	14	18	9	12	15	19	24
	140	2	3	4	6	7	4	5	7	9	12	4	5	6	8	11	5	7	9	11	14
	120	1	2	2	3	4	2	3	4	5	7	2	3	4	5	6	3	4	5	7	8

Costa Rica

Systolic Blood Pressur (mmHg) by Body Mass Index (kg/m2)

		Women										Men									
		Non−smoker					Smoker					Non−smoker					Smoker				
Age	SBP	20	25	30	35	40	20	25	30	35	40	20	25	30	35	40	20	25	30	35	40
70−74	180	17	22	27	34	41	27	34	41	49	58	21	27	33	40	48	28	34	42	50	59
	160	13	16	20	25	31	20	25	31	38	46	16	20	25	30	37	21	26	32	39	47
	140	9	11	15	18	23	14	18	23	28	35	11	14	18	23	28	15	19	24	30	37
	120	6	8	10	13	17	10	13	17	21	26	8	10	13	17	21	11	14	18	22	28
65−69	180	13	16	20	25	31	20	25	31	38	46	18	23	28	35	42	24	30	36	44	52
	160	9	11	14	18	22	14	18	22	28	34	13	16	20	25	31	17	21	27	33	40
	140	6	8	10	12	16	10	12	16	20	25	9	11	14	18	23	12	15	19	24	30
	120	4	5	7	9	11	7	9	11	14	18	6	8	10	13	16	8	11	14	17	21
60−64	180	9	12	15	19	24	15	19	24	30	37	15	19	24	30	37	21	26	32	39	47
	160	6	8	10	13	16	10	13	16	21	26	10	13	17	21	26	14	18	22	28	34
	140	4	5	7	9	11	7	9	11	14	18	7	9	11	14	18	9	12	15	19	24
	120	2	3	4	6	7	4	6	7	9	12	5	6	8	10	12	6	8	10	13	17
55−59	180	8	10	13	17	21	13	17	21	26	32	15	19	24	30	36	20	25	31	38	46
	160	5	6	8	11	14	8	11	14	17	21	10	13	16	20	25	13	17	21	26	33
	140	3	4	5	7	9	5	7	9	11	14	6	8	10	13	17	9	11	14	18	22
	120	2	2	3	4	5	3	4	5	7	9	4	5	7	9	11	5	7	9	12	15
50−54	180	8	10	12	16	20	12	16	20	25	31	14	18	23	28	35	19	24	30	36	44
	160	5	6	8	10	13	8	10	12	16	20	9	11	15	18	23	12	15	19	24	30
	140	3	3	5	6	8	5	6	8	10	13	5	7	9	12	15	8	10	12	16	20
	120	1	2	3	4	5	3	3	5	6	8	3	4	6	7	9	5	6	8	10	13
45−49	180	6	8	10	13	16	10	13	16	21	26	12	15	18	23	29	16	20	24	30	37
	160	4	5	6	8	10	6	8	10	13	16	7	9	11	14	18	9	12	15	19	24
	140	2	3	3	4	6	3	4	6	8	10	4	5	7	9	11	6	7	9	12	15
	120	1	1	2	3	3	2	2	3	4	6	2	3	4	5	7	3	4	6	7	9
40−44	180	4	5	7	9	12	7	9	12	15	18	8	10	13	16	20	11	14	17	22	27
	160	2	3	4	5	7	4	5	7	8	11	4	6	7	10	12	6	8	10	13	16
	140	1	1	2	3	4	2	3	4	5	6	2	3	4	6	7	3	5	6	8	10
	120	0	1	1	1	2	1	1	2	3	3	1	2	2	3	4	2	2	3	4	6

Cuba

Age	Systolic Blood Pressur (mmHg)	Women										Men									
		Non−smoker					Smoker					Non−smoker					Smoker				
	Body Mass Index (kg/m2)	20	25	30	35	40	20	25	30	35	40	20	25	30	35	40	20	25	30	35	40
70−74	180	25	31	38	46	54	38	46	54	63	72	28	34	41	49	58	36	43	52	60	70
	160	18	23	28	35	42	28	35	42	51	60	20	25	31	38	46	27	33	40	48	57
	140	13	17	21	26	32	21	26	32	39	47	15	19	24	29	36	20	25	31	38	45
	120	9	12	15	19	24	15	19	24	30	36	11	14	17	22	27	15	18	23	29	35
65−69	180	20	25	31	38	46	31	38	46	55	64	24	30	37	45	53	32	39	47	56	65
	160	14	18	23	28	34	22	28	34	42	50	17	22	27	34	41	23	29	35	43	51
	140	10	12	16	20	25	16	20	25	31	38	12	16	20	24	30	16	21	26	32	39
	120	7	9	11	14	18	11	14	18	22	27	8	11	14	17	22	12	15	18	23	29
60−64	180	17	22	27	33	40	27	33	40	48	57	23	28	35	42	50	30	36	44	52	61
	160	12	15	18	23	29	18	23	29	35	43	16	20	24	30	37	21	26	32	39	47
	140	8	10	12	16	20	12	16	20	25	31	11	13	17	21	26	14	18	22	28	34
	120	5	6	8	10	13	8	10	13	17	21	7	9	11	15	18	10	12	15	19	24
55−59	180	14	17	22	27	33	22	27	33	40	48	20	25	31	38	46	27	33	40	48	57
	160	9	11	14	18	23	14	18	22	28	34	13	17	21	26	33	18	22	28	34	42
	140	5	7	9	12	15	9	12	15	19	23	9	11	14	18	22	12	15	19	24	29
	120	3	4	6	7	10	6	7	10	12	15	6	7	9	12	15	8	10	12	16	20
50−54	180	10	13	16	21	26	16	21	26	32	39	17	22	27	33	41	23	28	35	42	51
	160	6	8	10	13	17	10	13	17	21	26	11	14	18	22	28	15	19	23	29	36
	140	4	5	6	8	10	6	8	10	13	17	7	9	11	14	18	9	12	15	19	24
	120	2	3	4	5	6	4	5	6	8	10	4	5	7	9	11	6	7	10	12	15
45−49	180	7	9	12	15	19	12	15	19	24	30	13	17	21	26	32	17	22	27	34	41
	160	4	6	7	9	12	7	9	12	15	19	8	10	13	16	20	11	14	17	22	27
	140	2	3	4	5	7	4	5	7	9	11	5	6	8	10	13	6	8	11	13	17
	120	1	2	2	3	4	2	3	4	5	7	3	3	5	6	8	4	5	6	8	10
40−44	180	5	6	8	10	12	8	10	12	16	20	9	12	15	19	23	12	16	20	24	30
	160	2	3	4	6	7	4	6	7	9	12	5	7	9	11	14	7	9	12	15	19
	140	1	2	2	3	4	2	3	4	5	7	3	4	5	6	8	4	5	7	9	11
	120	0	1	1	2	2	1	2	2	3	4	1	2	3	4	5	2	3	4	5	6

Dominican Republic

Systolic Blood Pressur (mmHg) by Body Mass Index (kg/m2)

Women — Non−smoker

Age	Systolic BP	20	25	30	35	40
70−74	180	25	31	38	46	55
	160	19	23	29	35	43
	140	13	17	21	26	33
	120	10	12	15	19	24
65−69	180	21	27	33	40	48
	160	15	19	24	29	36
	140	10	13	17	21	26
	120	7	9	12	15	19
60−64	180	18	23	28	35	42
	160	12	15	19	24	30
	140	8	10	13	17	21
	120	5	7	9	11	14
55−59	180	18	22	27	34	41
	160	11	14	18	23	28
	140	7	9	12	15	19
	120	4	6	8	10	12
50−54	180	15	19	24	29	36
	160	9	12	15	19	24
	140	6	7	9	12	15
	120	3	4	6	7	9
45−49	180	12	15	19	23	29
	160	7	9	11	14	18
	140	4	5	7	9	11
	120	2	3	4	5	7
40−44	180	9	12	15	19	23
	160	5	7	9	11	14
	140	3	4	5	6	8
	120	1	2	3	3	4

Women — Smoker

Age	Systolic BP	20	25	30	35	40
70−74	180	38	46	55	64	73
	160	29	35	43	51	60
	140	21	26	33	40	48
	120	15	19	24	30	37
65−69	180	33	40	48	57	66
	160	24	29	36	44	52
	140	17	21	26	32	39
	120	12	15	18	23	29
60−64	180	28	35	42	50	59
	160	19	24	30	37	44
	140	13	16	21	26	32
	120	9	11	14	18	22
55−59	180	27	34	41	49	58
	160	18	23	28	35	42
	140	12	15	19	24	29
	120	8	10	12	16	20
50−54	180	23	29	36	43	52
	160	15	19	24	29	36
	140	9	12	15	19	24
	120	6	7	9	12	15
45−49	180	19	23	29	36	43
	160	11	14	18	23	28
	140	7	9	11	14	18
	120	4	5	7	8	11
40−44	180	15	19	23	29	36
	160	9	11	14	18	22
	140	5	6	8	10	13
	120	3	3	4	6	8

Men — Non−smoker

Age	Systolic BP	20	25	30	35	40
70−74	180	27	34	41	49	58
	160	20	25	31	38	46
	140	15	19	23	29	36
	120	11	14	17	22	27
65−69	180	27	34	41	49	58
	160	20	24	30	37	45
	140	14	17	22	27	34
	120	10	12	16	20	24
60−64	180	27	33	40	48	57
	160	18	23	29	35	43
	140	13	16	20	25	31
	120	8	11	14	17	22
55−59	180	26	32	39	47	56
	160	18	22	27	34	41
	140	12	15	18	23	29
	120	7	10	12	15	19
50−54	180	23	29	35	43	51
	160	15	19	23	29	36
	140	9	12	15	19	24
	120	6	7	10	12	16
45−49	180	18	23	29	35	43
	160	11	14	18	23	28
	140	7	9	11	14	18
	120	4	5	7	9	11
40−44	180	12	16	20	25	30
	160	7	9	12	15	19
	140	4	5	7	9	11
	120	2	3	4	5	7

Men — Smoker

Age	Systolic BP	20	25	30	35	40
70−74	180	35	43	51	60	69
	160	27	33	40	48	57
	140	20	25	30	37	45
	120	14	18	23	28	35
65−69	180	35	43	51	60	69
	160	26	32	39	47	55
	140	18	23	29	35	43
	120	13	16	21	26	32
60−64	180	34	42	50	59	68
	160	24	30	37	45	53
	140	17	21	26	33	40
	120	11	15	18	23	29
55−59	180	34	41	49	58	67
	160	23	29	35	43	51
	140	15	20	24	30	37
	120	10	13	16	21	26
50−54	180	30	37	45	53	62
	160	20	25	31	38	45
	140	13	16	20	25	31
	120	8	10	13	16	21
45−49	180	24	30	37	45	53
	160	15	19	24	30	36
	140	9	12	15	19	24
	120	6	7	9	12	15
40−44	180	17	21	26	32	39
	160	10	13	16	20	25
	140	6	7	9	12	15
	120	3	4	5	7	9

Body Mass Index (kg/m2)

Ecuador

Age	Systolic Blood Pressur (mmHg)	Women Non−smoker					Women Smoker					Men Non−smoker					Men Smoker				
	Body Mass Index (kg/m2)	20	25	30	35	40	20	25	30	35	40	20	25	30	35	40	20	25	30	35	40
70−74	180	18	23	29	35	43	28	35	42	51	60	23	29	35	43	51	30	37	45	53	62
	160	13	17	21	26	32	21	26	32	39	47	17	21	26	33	40	22	28	34	42	50
	140	9	12	15	19	24	15	19	24	30	36	12	16	20	25	30	17	21	26	32	39
	120	7	9	11	14	18	11	14	18	22	27	9	11	14	18	23	12	15	19	24	30
65−69	180	13	17	21	26	32	21	26	32	39	47	19	23	29	35	43	24	30	37	45	53
	160	9	11	15	18	23	15	18	23	29	35	13	17	21	26	32	17	22	27	34	41
	140	6	8	10	13	16	10	13	16	20	25	9	12	15	19	23	12	16	20	24	30
	120	4	5	7	9	11	7	9	11	14	18	6	8	10	13	17	9	11	14	18	22
60−64	180	10	13	17	21	26	17	21	26	32	39	17	21	27	33	40	23	28	35	42	50
	160	7	9	11	14	18	11	14	18	23	28	12	15	19	23	29	16	20	24	30	37
	140	4	6	7	9	12	7	9	12	15	19	8	10	13	16	20	10	13	17	21	26
	120	3	4	5	6	8	5	6	8	10	13	5	7	8	11	14	7	9	11	15	18
55−59	180	9	11	14	18	22	14	18	22	27	34	16	21	26	32	39	22	27	33	41	49
	160	5	7	9	11	15	9	11	15	18	23	11	14	17	22	27	14	18	23	28	35
	140	3	4	6	7	9	6	7	9	12	15	7	9	11	14	18	9	12	15	19	24
	120	2	3	3	5	6	3	4	6	8	10	4	6	7	9	12	6	8	10	13	16
50−54	180	8	10	13	17	21	13	16	21	26	32	15	18	23	29	35	19	24	30	37	45
	160	5	6	8	10	13	8	10	13	17	21	9	12	15	19	23	12	16	20	25	31
	140	3	4	5	6	8	5	6	8	10	13	6	7	9	12	15	8	10	13	16	20
	120	2	2	3	4	5	3	4	5	6	8	3	4	6	7	10	5	6	8	10	13
45−49	180	7	9	11	14	18	11	14	18	22	27	12	15	19	24	29	16	20	25	31	38
	160	4	5	6	8	11	6	8	11	13	17	7	9	12	15	19	10	12	16	20	25
	140	2	3	4	5	6	4	5	6	8	10	4	5	7	9	12	6	7	10	12	16
	120	1	1	2	3	4	2	3	4	5	6	2	3	4	5	7	3	4	6	7	9
40−44	180	5	7	8	11	14	8	11	14	17	22	9	12	15	19	24	12	16	20	25	31
	160	3	4	5	6	8	5	6	8	10	13	5	7	9	11	14	7	9	12	15	19
	140	1	2	3	3	4	3	3	4	6	7	3	4	5	6	8	4	5	7	9	11
	120	1	1	1	2	2	1	2	2	3	4	1	2	3	4	5	2	3	4	5	7

El Salvador

Systolic Blood Pressur (mmHg) by Body Mass Index (kg/m2)

Women (Non−smoker, Smoker) · Men (Non−smoker, Smoker)

Age 70−74

SBP	Women Non−smoker 20	25	30	35	40	Women Smoker 20	25	30	35	40	Men Non−smoker 20	25	30	35	40	Men Smoker 20	25	30	35	40
180	25	31	38	46	55	38	46	54	63	72	23	29	36	43	52	31	37	45	54	63
160	18	23	29	35	43	29	35	43	51	60	17	22	27	33	40	23	28	35	42	51
140	13	17	21	26	32	21	26	32	39	47	13	16	20	25	31	17	21	26	32	40
120	9	12	15	19	24	15	19	24	30	37	9	12	15	18	23	12	15	20	24	30

Age 65−69

SBP	Women Non−smoker 20	25	30	35	40	Women Smoker 20	25	30	35	40	Men Non−smoker 20	25	30	35	40	Men Smoker 20	25	30	35	40
180	18	23	28	35	42	28	35	42	51	59	20	25	31	38	46	26	33	40	48	56
160	13	16	20	25	31	20	25	31	38	46	14	18	22	28	34	19	24	29	36	44
140	9	11	14	18	22	14	18	22	28	34	10	13	16	20	25	13	17	21	26	32
120	6	8	10	12	16	10	12	16	20	25	7	9	11	14	18	9	12	15	19	24

Age 60−64

SBP	Women Non−smoker 20	25	30	35	40	Women Smoker 20	25	30	35	40	Men Non−smoker 20	25	30	35	40	Men Smoker 20	25	30	35	40
180	15	19	24	29	36	24	29	36	44	52	20	25	31	38	46	27	33	40	48	57
160	10	13	16	20	25	16	20	25	31	38	14	17	22	27	34	18	23	29	35	43
140	7	8	11	14	17	11	14	17	22	27	9	12	15	19	24	13	16	20	25	31
120	4	5	7	9	12	7	9	12	15	19	6	8	10	13	16	8	11	14	17	22

Age 55−59

SBP	Women Non−smoker 20	25	30	35	40	Women Smoker 20	25	30	35	40	Men Non−smoker 20	25	30	35	40	Men Smoker 20	25	30	35	40
180	14	18	22	28	34	22	27	34	41	49	20	26	32	39	46	27	33	40	49	57
160	9	11	15	18	23	15	18	23	28	35	14	17	22	27	33	18	23	28	35	42
140	6	7	9	12	15	9	12	15	19	24	9	11	14	18	23	12	15	19	24	30
120	3	4	6	8	10	6	8	10	12	16	6	7	9	12	15	8	10	13	16	20

Age 50−54

SBP	Women Non−smoker 20	25	30	35	40	Women Smoker 20	25	30	35	40	Men Non−smoker 20	25	30	35	40	Men Smoker 20	25	30	35	40
180	13	16	20	25	31	20	25	31	38	46	19	24	30	37	44	25	31	38	46	55
160	8	10	13	16	20	13	16	20	25	31	12	16	20	25	30	17	21	26	32	39
140	5	6	8	10	13	8	10	13	16	21	8	10	13	16	20	10	13	17	21	26
120	3	4	5	6	8	5	6	8	10	13	5	6	8	10	13	6	8	11	14	17

Age 45−49

SBP	Women Non−smoker 20	25	30	35	40	Women Smoker 20	25	30	35	40	Men Non−smoker 20	25	30	35	40	Men Smoker 20	25	30	35	40
180	11	14	18	23	28	18	23	28	35	42	17	21	26	32	39	22	28	34	41	49
160	7	9	11	14	18	11	14	18	22	28	10	13	16	21	26	14	17	22	27	33
140	4	5	7	8	11	6	8	11	14	17	6	8	10	13	16	8	11	14	17	22
120	2	3	4	5	6	4	5	6	8	10	4	5	6	8	10	5	6	8	11	13

Age 40−44

SBP	Women Non−smoker 20	25	30	35	40	Women Smoker 20	25	30	35	40	Men Non−smoker 20	25	30	35	40	Men Smoker 20	25	30	35	40
180	9	12	15	19	24	15	19	24	29	36	14	18	22	27	34	19	23	29	35	43
160	5	7	9	11	14	9	11	14	18	22	8	10	13	17	21	11	14	18	22	28
140	3	4	5	6	8	5	6	8	10	13	5	6	8	10	13	6	8	11	13	17
120	1	2	3	3	5	3	3	5	6	8	2	3	4	6	7	4	5	6	8	10

Body Mass Index (kg/m2)

Grenada

Systolic Blood Pressur (mmHg) by Body Mass Index (kg/m2)

Women

Non−smoker

Age	Systolic Blood Pressur (mmHg)	20	25	30	35	40
70−74	180	26	32	39	46	55
	160	19	23	29	36	43
	140	13	17	21	27	33
	120	10	12	16	20	24
65−69	180	20	25	31	38	46
	160	14	18	22	28	34
	140	10	12	16	20	25
	120	7	8	11	14	17
60−64	180	16	21	26	32	39
	160	11	14	18	22	27
	140	7	9	12	15	19
	120	5	6	8	10	13
55−59	180	14	18	22	28	34
	160	9	11	15	18	23
	140	6	7	9	12	15
	120	3	5	6	8	10
50−54	180	12	16	20	25	31
	160	8	10	12	16	20
	140	5	6	8	10	13
	120	3	4	5	6	8
45−49	180	9	12	15	19	24
	160	6	7	9	12	15
	140	3	4	5	7	9
	120	2	2	3	4	5
40−44	180	7	9	11	15	18
	160	4	5	7	8	11
	140	2	3	4	5	6
	120	1	1	2	3	3

Smoker

Age	Systolic Blood Pressur (mmHg)	20	25	30	35	40
70−74	180	38	46	55	64	73
	160	29	36	43	51	60
	140	21	27	33	40	48
	120	15	20	24	30	37
65−69	180	31	38	46	54	63
	160	22	28	34	41	50
	140	16	20	25	30	37
	120	11	14	17	22	27
60−64	180	26	32	39	46	55
	160	17	22	27	34	41
	140	12	15	19	23	29
	120	8	10	13	16	20
55−59	180	22	28	34	41	49
	160	15	18	23	28	35
	140	9	12	15	19	24
	120	6	8	10	12	16
50−54	180	20	25	30	37	45
	160	12	16	20	25	31
	140	8	10	13	16	20
	120	5	6	8	10	13
45−49	180	15	19	24	30	36
	160	9	12	15	19	23
	140	5	7	9	11	15
	120	3	4	5	7	9
40−44	180	11	14	18	23	28
	160	7	8	11	14	17
	140	4	5	6	8	10
	120	2	3	3	4	6

Men

Non−smoker

Age	Systolic Blood Pressur (mmHg)	20	25	30	35	40
70−74	180	33	40	49	57	66
	160	25	31	38	46	54
	140	18	23	29	35	43
	120	13	17	21	27	33
65−69	180	29	35	43	51	60
	160	21	26	32	39	47
	140	15	19	23	29	35
	120	10	13	17	21	26
60−64	180	26	33	40	48	56
	160	18	23	28	35	42
	140	12	16	20	25	31
	120	8	11	14	17	22
55−59	180	25	31	38	45	54
	160	17	21	26	32	39
	140	11	14	18	22	27
	120	7	9	12	15	18
50−54	180	23	29	35	43	51
	160	15	19	24	29	36
	140	9	12	15	19	24
	120	6	8	10	12	16
45−49	180	17	22	27	33	40
	160	11	14	17	21	27
	140	6	8	11	13	17
	120	4	5	6	8	10
40−44	180	12	15	19	24	30
	160	7	9	12	15	18
	140	4	5	7	9	11
	120	2	3	4	5	6

Smoker

Age	Systolic Blood Pressur (mmHg)	20	25	30	35	40
70−74	180	42	51	60	69	77
	160	32	40	48	56	65
	140	24	30	37	45	53
	120	18	23	28	34	42
65−69	180	37	45	53	62	71
	160	27	34	41	49	58
	140	20	24	30	37	45
	120	14	17	22	27	34
60−64	180	34	42	50	59	68
	160	24	30	37	44	53
	140	17	21	26	32	39
	120	11	14	18	23	28
55−59	180	32	39	48	56	65
	160	22	28	34	41	49
	140	15	19	23	29	35
	120	10	12	16	20	24
50−54	180	30	37	45	53	62
	160	20	25	31	38	46
	140	13	16	20	25	31
	120	8	10	13	17	21
45−49	180	23	28	35	42	51
	160	14	18	23	28	35
	140	9	11	14	18	22
	120	5	7	9	11	14
40−44	180	16	20	25	31	38
	160	10	12	16	20	24
	140	5	7	9	12	15
	120	3	4	5	7	9

Body Mass Index (kg/m2)

Guatemala

Systolic Blood Pressur (mmHg) by Body Mass Index (kg/m2)

Women

Non−smoker

Age	SBP	20	25	30	35	40
70−74	180	19	24	30	37	45
	160	14	18	22	28	34
	140	10	13	16	20	26
	120	7	9	12	15	19
65−69	180	14	18	22	28	34
	160	10	12	16	20	25
	140	7	9	11	14	18
	120	4	6	7	10	12
60−64	180	12	15	19	24	29
	160	8	10	13	16	20
	140	5	7	8	11	14
	120	3	4	5	7	9
55−59	180	10	13	17	21	26
	160	7	9	11	14	18
	140	4	5	7	9	11
	120	2	3	4	6	7
50−54	180	10	13	16	20	25
	160	6	8	10	13	16
	140	4	5	6	8	10
	120	2	3	4	5	6
45−49	180	9	11	15	18	23
	160	5	7	9	11	14
	140	3	4	5	7	9
	120	2	2	3	4	5
40−44	180	7	9	11	14	18
	160	4	5	6	8	10
	140	2	3	3	4	6
	120	1	1	2	2	3

Smoker

Age	SBP	20	25	30	35	40
70−74	180	30	37	45	53	62
	160	22	28	34	41	50
	140	16	20	25	31	38
	120	12	15	19	23	29
65−69	180	22	28	34	42	50
	160	16	20	25	31	37
	140	11	14	18	22	27
	120	7	10	12	15	19
60−64	180	19	23	29	36	43
	160	13	16	20	25	31
	140	8	11	14	17	22
	120	5	7	9	12	15
55−59	180	17	21	26	32	40
	160	11	14	18	22	27
	140	7	9	11	14	18
	120	4	6	7	9	12
50−54	180	16	20	25	31	38
	160	10	13	16	20	26
	140	6	8	10	13	16
	120	4	5	6	8	10
45−49	180	15	18	23	29	35
	160	9	11	14	18	22
	140	5	7	9	11	14
	120	3	4	5	6	8
40−44	180	11	14	17	22	27
	160	6	8	10	13	17
	140	3	4	6	8	10
	120	2	2	3	4	5

Men

Non−smoker

Age	SBP	20	25	30	35	40
70−74	180	26	32	39	47	55
	160	19	24	30	36	44
	140	14	18	22	28	34
	120	10	13	16	21	26
65−69	180	19	24	30	37	44
	160	14	17	22	27	33
	140	10	12	15	19	24
	120	7	8	11	14	17
60−64	180	15	19	24	30	37
	160	10	13	17	21	26
	140	7	9	11	15	18
	120	5	6	8	10	13
55−59	180	14	18	22	28	34
	160	9	12	15	19	24
	140	6	8	10	12	16
	120	4	5	6	8	10
50−54	180	13	17	21	26	33
	160	8	11	14	17	22
	140	5	7	9	11	14
	120	3	4	5	7	9
45−49	180	12	15	19	24	29
	160	7	9	12	15	19
	140	4	5	7	9	12
	120	2	3	4	5	7
40−44	180	10	12	16	20	25
	160	6	7	9	12	15
	140	3	4	5	7	9
	120	2	2	3	4	5

Smoker

Age	SBP	20	25	30	35	40
70−74	180	33	41	49	58	67
	160	25	31	38	46	55
	140	19	23	29	36	43
	120	14	17	22	27	33
65−69	180	25	31	38	46	55
	160	18	23	28	35	42
	140	13	16	21	26	32
	120	9	11	15	18	23
60−64	180	21	26	32	39	47
	160	14	18	22	28	34
	140	10	12	15	19	24
	120	6	8	10	13	17
55−59	180	19	24	29	36	44
	160	13	16	20	25	31
	140	8	10	13	17	21
	120	5	7	9	11	14
50−54	180	18	22	28	34	42
	160	11	14	18	23	28
	140	7	9	12	15	19
	120	4	6	7	9	12
45−49	180	16	20	25	31	38
	160	10	12	16	20	25
	140	6	8	10	12	16
	120	3	4	6	7	10
40−44	180	13	17	21	26	32
	160	8	10	12	16	20
	140	4	6	7	9	12
	120	2	3	4	5	7

Body Mass Index (kg/m2)

Guyana

Systolic Blood Pressur (mmHg) by Body Mass Index (kg/m2)

Age	SBP	Women Non−smoker 20	25	30	35	40	Women Smoker 20	25	30	35	40	Men Non−smoker 20	25	30	35	40	Men Smoker 20	25	30	35	40
70−74	180	40	48	57	66	75	57	66	75	83	90	52	61	70	79	86	64	72	81	88	93
	160	30	37	45	54	63	45	53	63	72	80	41	49	58	67	76	51	60	69	78	86
	140	23	28	34	42	50	34	42	50	59	68	32	39	46	55	64	40	49	57	66	75
	120	16	21	26	32	39	26	32	39	47	55	24	29	36	44	52	31	38	46	54	63
65−69	180	31	38	46	55	64	46	55	64	73	81	43	51	60	69	78	53	62	71	80	87
	160	23	28	35	42	50	35	42	50	59	68	32	39	47	55	65	41	49	58	67	76
	140	16	20	25	31	38	25	31	38	46	54	23	29	35	43	51	30	37	45	53	62
	120	11	14	18	22	28	18	22	28	34	41	17	21	26	32	39	22	27	34	41	49
60−64	180	25	31	38	46	55	38	46	55	64	73	36	43	52	61	70	45	54	63	72	80
	160	17	22	27	33	41	27	33	40	49	57	26	32	39	46	55	33	40	48	57	66
	140	12	15	19	23	29	19	23	29	35	43	18	22	28	34	41	23	29	36	43	52
	120	8	10	13	16	20	13	16	20	25	31	12	15	19	24	30	16	20	25	31	38
55−59	180	22	27	34	41	49	34	41	49	58	67	32	40	48	56	65	41	50	59	68	76
	160	14	18	23	28	35	23	28	35	42	50	22	28	34	41	50	29	36	43	52	61
	140	9	12	15	19	24	15	19	24	29	36	15	19	23	29	36	20	25	30	37	45
	120	6	8	10	12	16	10	12	16	20	25	10	12	16	20	24	13	16	21	26	32
50−54	180	17	21	26	32	40	26	32	39	47	56	28	34	42	50	59	36	44	52	61	70
	160	11	13	17	21	26	17	21	26	33	40	18	23	29	35	43	24	30	37	44	53
	140	6	8	11	14	17	11	13	17	21	27	12	15	19	23	29	16	20	25	31	37
	120	4	5	6	8	11	6	8	11	14	17	7	9	12	15	19	10	13	16	20	25
45−49	180	13	16	20	25	31	20	25	31	38	46	21	26	32	39	47	27	34	41	49	58
	160	8	10	12	16	20	12	16	20	25	30	13	16	21	26	32	17	22	27	33	41
	140	4	6	7	9	12	7	9	12	15	19	8	10	13	16	20	11	14	17	22	27
	120	2	3	4	6	7	4	6	7	9	12	5	6	8	10	13	6	8	11	13	17
40−44	180	10	13	16	21	26	16	21	26	32	39	13	16	20	25	31	17	22	27	33	40
	160	6	7	10	12	15	10	12	15	19	24	7	10	12	16	20	10	13	16	21	26
	140	3	4	5	7	9	5	7	9	11	15	4	6	7	9	12	6	8	10	12	16
	120	2	2	3	4	5	3	4	5	7	8	2	3	4	5	7	3	4	6	7	9

Haiti

Systolic Blood Pressur (mmHg) by Body Mass Index (kg/m2)

Age	Systolic BP	Women Non−smoker 20	25	30	35	40	Women Smoker 20	25	30	35	40	Men Non−smoker 20	25	30	35	40	Men Smoker 20	25	30	35	40
70−74	180	37	45	54	63	72	53	62	72	80	87	36	43	52	61	70	45	54	63	72	80
	160	28	34	42	50	59	42	50	59	68	77	27	33	41	49	58	35	43	51	60	69
	140	21	26	32	39	47	32	39	47	55	64	20	25	31	38	46	27	33	40	48	57
	120	15	19	24	29	36	24	29	36	43	52	15	19	23	29	36	20	25	31	37	45
65−69	180	28	34	41	50	59	41	50	58	67	76	29	35	43	51	60	37	45	53	62	71
	160	20	25	31	37	45	30	37	45	54	63	21	26	32	39	47	27	34	41	49	58
	140	14	17	22	27	34	22	27	33	41	49	15	19	23	29	35	20	25	30	37	45
	120	10	12	15	19	24	15	19	24	30	37	10	13	17	21	26	14	18	22	27	34
60−64	180	21	26	32	39	47	32	39	47	56	65	24	30	37	45	53	32	39	47	56	65
	160	14	18	23	28	34	23	28	34	42	50	17	21	26	33	40	22	28	34	42	50
	140	9	12	15	19	24	15	19	24	30	37	11	15	18	23	29	15	19	24	30	37
	120	6	8	10	13	17	10	13	17	21	26	8	10	13	16	20	10	13	17	21	26
55−59	180	17	22	27	33	40	27	33	40	48	57	22	27	33	41	49	28	35	42	51	60
	160	11	14	18	22	28	18	22	28	34	42	14	18	23	28	35	19	24	30	37	44
	140	7	9	12	15	19	12	15	19	23	29	9	12	15	19	24	13	16	20	25	31
	120	4	6	7	9	12	7	9	12	15	19	6	8	10	13	16	8	11	13	17	21
50−54	180	15	19	24	30	37	24	30	37	44	53	19	24	29	36	44	25	31	38	46	54
	160	10	12	15	19	24	15	19	24	30	37	12	15	19	24	30	16	20	25	31	38
	140	6	7	10	12	16	10	12	16	20	24	8	10	12	16	20	10	13	17	21	26
	120	3	4	6	8	10	6	8	10	12	16	5	6	8	10	13	6	8	10	13	17
45−49	180	14	18	23	28	34	23	28	34	42	50	16	20	25	31	38	21	26	33	40	48
	160	9	11	14	18	22	14	18	22	27	34	10	12	16	20	25	13	17	21	26	32
	140	5	6	8	11	14	8	11	14	17	21	6	8	10	12	16	8	10	13	17	21
	120	3	4	5	6	8	5	6	8	10	13	3	4	6	7	10	5	6	8	10	13
40−44	180	13	16	20	25	31	20	25	31	38	46	13	17	21	26	32	18	22	27	34	41
	160	7	9	12	15	19	12	15	19	24	30	8	10	12	16	20	10	13	17	21	26
	140	4	5	7	9	11	7	9	11	14	18	4	6	7	9	12	6	8	10	13	16
	120	2	3	4	5	6	4	5	6	8	11	2	3	4	5	7	3	4	6	7	9

Honduras

Systolic Blood Pressur (mmHg) by Body Mass Index (kg/m2)

Age	Systolic Blood Pressur (mmHg)	Women Non−smoker					Women Smoker					Men Non−smoker					Men Smoker				
		20	25	30	35	40	20	25	30	35	40	20	25	30	35	40	20	25	30	35	40
70−74	180	20	25	31	38	46	31	38	46	54	63	22	27	34	41	49	29	35	43	51	60
	160	15	18	23	29	35	23	28	35	42	51	16	20	25	31	38	21	27	33	40	48
	140	10	13	17	21	26	17	21	26	32	39	12	15	19	23	29	16	20	25	31	37
	120	7	9	12	15	19	12	15	19	24	30	8	11	14	17	22	11	15	18	23	28
65−69	180	17	21	26	33	40	26	32	40	48	56	20	25	31	38	46	27	33	40	48	57
	160	12	15	19	23	29	19	23	29	36	43	14	18	23	28	35	19	24	30	36	44
	140	8	10	13	16	21	13	16	21	26	32	10	13	16	20	25	13	17	21	27	33
	120	5	7	9	11	15	9	11	14	18	23	7	9	11	14	18	9	12	15	19	24
60−64	180	14	18	22	27	34	22	27	34	41	49	19	24	30	37	44	25	31	38	46	55
	160	9	12	15	19	24	15	19	24	29	36	13	17	21	26	32	18	22	28	34	41
	140	6	8	10	13	16	10	13	16	20	25	9	11	14	18	23	12	15	19	24	30
	120	4	5	7	8	11	7	8	11	14	17	6	8	10	12	16	8	10	13	17	21
55−59	180	12	16	20	25	30	20	25	30	37	45	17	22	27	34	41	23	29	35	43	51
	160	8	10	13	16	20	13	16	20	25	31	11	15	18	23	29	15	19	24	30	37
	140	5	6	8	10	13	8	10	13	17	21	7	9	12	15	19	10	13	16	20	25
	120	3	4	5	7	9	5	7	8	11	14	5	6	8	10	13	6	8	11	13	17
50−54	180	12	16	20	24	30	20	24	30	37	45	16	20	25	31	38	21	26	33	40	48
	160	8	10	12	16	20	12	16	20	24	30	10	13	16	20	25	14	17	22	27	33
	140	4	6	8	10	12	8	10	12	16	20	6	8	10	13	17	9	11	14	18	22
	120	3	3	5	6	8	4	6	8	10	12	4	5	6	8	10	5	7	9	11	14
45−49	180	12	16	20	25	30	20	24	30	37	45	13	16	20	25	31	17	21	27	33	40
	160	7	9	12	15	19	12	15	19	24	30	8	10	13	16	20	10	13	17	21	26
	140	4	5	7	9	12	7	9	12	15	19	5	6	8	10	12	6	8	10	13	17
	120	2	3	4	5	7	4	5	7	9	11	3	3	4	6	8	4	5	6	8	10
40−44	180	11	14	18	22	28	18	22	28	34	42	9	12	15	19	24	13	16	20	25	31
	160	6	8	10	13	17	10	13	17	21	26	5	7	9	11	14	7	9	12	15	19
	140	3	5	6	8	10	6	8	10	13	16	3	4	5	7	8	4	5	7	9	11
	120	2	2	3	4	6	3	4	6	7	9	1	2	3	4	5	2	3	4	5	7

Body Mass Index (kg/m2)

Jamaica

Systolic Blood Pressur (mmHg) by Body Mass Index (kg/m2)

Women

Age	SBP	Non−smoker 20	25	30	35	40	Smoker 20	25	30	35	40
70−74	180	20	25	30	37	45	30	37	45	53	62
	160	14	18	22	28	34	22	28	34	42	50
	140	10	13	16	21	26	16	21	26	32	39
	120	7	9	12	15	19	12	15	19	24	29
65−69	180	14	17	22	27	33	21	27	33	40	48
	160	9	12	15	19	24	15	19	24	29	36
	140	6	8	10	13	17	10	13	17	21	26
	120	4	5	7	9	12	7	9	12	15	19
60−64	180	9	12	15	19	24	15	19	24	30	37
	160	6	8	10	13	17	10	13	16	21	26
	140	4	5	7	9	11	7	9	11	14	18
	120	2	3	4	6	7	4	6	7	9	12
55−59	180	7	9	11	14	18	11	14	18	22	28
	160	4	5	7	9	12	7	9	12	15	19
	140	3	3	4	6	7	4	6	7	10	12
	120	1	2	3	4	5	3	4	5	6	8
50−54	180	5	6	8	10	13	8	10	13	16	21
	160	3	4	5	6	8	5	6	8	10	13
	140	2	2	3	4	5	3	4	5	6	8
	120	1	1	2	2	3	2	2	3	4	5
45−49	180	4	5	7	9	11	7	9	11	14	18
	160	2	3	4	5	6	4	5	6	8	11
	140	1	2	2	3	4	2	3	4	5	6
	120	0	1	1	1	2	1	1	2	3	4
40−44	180	3	5	6	8	10	6	8	10	12	16
	160	2	2	3	4	5	3	4	5	7	9
	140	1	1	2	2	3	2	2	3	4	5
	120	0	0	1	1	2	1	1	2	2	3

Men

Age	SBP	Non−smoker 20	25	30	35	40	Smoker 20	25	30	35	40
70−74	180	24	30	37	45	53	32	39	47	56	65
	160	18	23	28	35	42	24	30	36	44	52
	140	13	17	21	26	32	18	22	28	34	41
	120	10	12	15	19	24	13	16	21	26	32
65−69	180	18	22	28	34	42	24	29	36	44	52
	160	13	16	20	25	31	17	21	26	33	40
	140	9	11	14	18	23	12	15	19	24	29
	120	6	8	10	13	16	8	11	13	17	21
60−64	180	14	17	22	27	34	19	23	29	35	43
	160	9	12	15	19	24	13	16	20	25	31
	140	6	8	10	13	16	8	11	14	17	22
	120	4	5	7	9	11	6	7	9	12	15
55−59	180	12	15	19	23	29	16	20	24	30	37
	160	7	10	12	15	19	10	13	16	21	26
	140	5	6	8	10	13	7	8	11	14	17
	120	3	4	5	6	8	4	5	7	9	11
50−54	180	10	12	16	20	25	13	16	21	26	32
	160	6	8	10	13	16	8	10	13	17	21
	140	4	5	6	8	10	5	6	8	11	14
	120	2	3	4	5	6	3	4	5	7	9
45−49	180	8	10	13	16	21	11	14	17	22	27
	160	5	6	8	10	13	6	8	11	14	17
	140	3	4	5	6	8	4	5	6	8	11
	120	1	2	3	3	5	2	3	4	5	6
40−44	180	6	8	10	13	16	8	11	14	17	22
	160	3	4	6	8	10	5	6	8	10	13
	140	2	2	3	4	6	3	3	5	6	8
	120	1	1	2	2	3	1	2	2	3	4

Body Mass Index (kg/m2)

Mexico

Systolic Blood Pressur (mmHg) by Body Mass Index (kg/m2)

Women

Age	Systolic Blood Pressure (mmHg)	Non−smoker BMI 20	25	30	35	40	Smoker BMI 20	25	30	35	40
70−74	180	22	27	34	41	49	34	41	49	58	67
	160	16	20	25	31	38	25	31	38	46	54
	140	11	15	18	23	29	18	23	28	35	42
	120	8	10	13	17	21	13	17	21	26	32
65−69	180	16	21	26	32	39	26	32	39	46	55
	160	11	14	18	23	28	18	23	28	35	42
	140	8	10	13	16	20	13	16	20	25	31
	120	5	7	9	11	14	9	11	14	18	22
60−64	180	13	16	20	25	31	20	25	31	38	45
	160	8	11	14	17	21	13	17	21	27	33
	140	5	7	9	11	15	9	11	15	18	23
	120	3	4	6	8	10	6	8	10	12	16
55−59	180	9	12	15	19	24	15	19	24	29	36
	160	6	8	10	12	16	10	12	16	20	25
	140	4	5	6	8	10	6	8	10	13	16
	120	2	3	4	5	6	4	5	6	8	11
50−54	180	7	10	12	15	19	12	15	19	24	30
	160	4	6	7	10	12	7	10	12	15	20
	140	3	3	4	6	8	4	6	8	10	12
	120	1	2	3	3	5	3	3	5	6	8
45−49	180	6	7	9	12	15	9	12	15	19	24
	160	3	4	5	7	9	5	7	9	12	15
	140	2	2	3	4	5	3	4	5	7	9
	120	1	1	2	2	3	2	2	3	4	5
40−44	180	4	5	7	9	11	7	9	11	14	18
	160	2	3	4	5	6	4	5	6	8	11
	140	1	1	2	3	4	2	3	4	5	6
	120	0	1	1	1	2	1	1	2	3	3

Men

Age	Systolic Blood Pressure (mmHg)	Non−smoker BMI 20	25	30	35	40	Smoker BMI 20	25	30	35	40
70−74	180	24	30	37	44	53	32	39	46	55	64
	160	18	22	28	34	42	24	29	36	44	52
	140	13	17	21	26	32	17	22	27	34	41
	120	9	12	15	19	24	13	16	20	25	31
65−69	180	19	24	30	37	45	26	32	39	47	55
	160	14	17	22	27	33	18	23	29	35	43
	140	10	12	16	20	24	13	16	21	26	32
	120	7	9	11	14	18	9	12	15	19	23
60−64	180	16	20	25	31	38	21	27	33	40	48
	160	11	14	18	22	27	15	19	23	29	36
	140	7	9	12	15	19	10	13	16	20	25
	120	5	6	8	10	13	7	9	11	14	18
55−59	180	14	18	22	28	34	19	23	29	36	43
	160	9	12	15	19	23	12	16	20	25	30
	140	6	7	10	12	16	8	10	13	16	21
	120	4	5	6	8	10	5	7	8	11	14
50−54	180	13	16	20	25	31	17	22	27	33	40
	160	8	10	13	17	21	11	14	17	22	27
	140	5	6	8	10	13	7	9	11	14	18
	120	3	4	5	6	8	4	5	7	9	11
45−49	180	11	14	18	22	28	15	19	24	29	36
	160	7	9	11	14	18	9	12	15	19	23
	140	4	5	7	8	11	5	7	9	11	15
	120	2	3	4	5	6	3	4	5	7	9
40−44	180	8	11	14	17	21	11	14	18	23	28
	160	5	6	8	10	13	7	8	11	14	17
	140	3	3	4	6	8	4	5	6	8	10
	120	1	2	2	3	4	2	3	3	5	6

Body Mass Index (kg/m2)

Nicaragua

Systolic Blood Pressur (mmHg) by Body Mass Index (kg/m2)

Women

Non−smoker

Age	SBP	20	25	30	35	40
70−74	180	25	31	38	46	55
	160	19	23	29	35	43
	140	13	17	21	26	33
	120	10	12	15	19	24
65−69	180	19	24	30	36	44
	160	13	17	21	26	33
	140	9	12	15	19	24
	120	6	8	10	13	17
60−64	180	16	21	26	32	39
	160	11	14	18	22	27
	140	7	9	12	15	19
	120	5	6	8	10	13
55−59	180	13	17	21	26	32
	160	8	11	14	17	22
	140	5	7	9	11	14
	120	3	4	6	7	9
50−54	180	10	13	16	20	26
	160	6	8	10	13	16
	140	4	5	6	8	10
	120	2	3	4	5	6
45−49	180	8	11	14	17	22
	160	5	6	8	10	13
	140	3	4	5	6	8
	120	1	2	3	3	5
40−44	180	7	10	12	15	19
	160	4	5	7	9	11
	140	2	3	4	5	7
	120	1	1	2	3	4

Smoker

Age	SBP	20	25	30	35	40
70−74	180	38	46	55	64	73
	160	29	35	43	51	60
	140	21	26	33	40	48
	120	15	19	24	30	37
65−69	180	30	36	44	53	62
	160	21	26	33	40	48
	140	15	19	23	29	36
	120	10	13	17	21	26
60−64	180	26	32	39	47	55
	160	18	22	27	34	41
	140	12	15	19	24	29
	120	8	10	13	16	20
55−59	180	21	26	32	39	47
	160	14	17	22	27	33
	140	9	11	14	18	23
	120	6	7	9	12	15
50−54	180	16	20	25	31	38
	160	10	13	16	21	26
	140	6	8	10	13	16
	120	4	5	6	8	10
45−49	180	14	17	22	27	33
	160	8	10	13	17	21
	140	5	6	8	10	13
	120	3	3	5	6	8
40−44	180	12	15	19	24	30
	160	7	9	11	14	18
	140	4	5	6	8	11
	120	2	3	4	5	6

Men

Non−smoker

Age	SBP	20	25	30	35	40
70−74	180	27	33	40	48	57
	160	20	25	31	38	45
	140	15	18	23	29	35
	120	11	13	17	21	26
65−69	180	23	28	35	42	51
	160	16	20	26	32	38
	140	11	14	18	23	28
	120	8	10	13	16	20
60−64	180	21	26	32	39	47
	160	14	18	22	28	34
	140	10	12	15	19	24
	120	6	8	10	13	17
55−59	180	17	22	27	34	41
	160	12	15	18	23	29
	140	7	10	12	15	19
	120	5	6	8	10	13
50−54	180	14	18	22	28	34
	160	9	11	14	18	23
	140	5	7	9	11	15
	120	3	4	6	7	9
45−49	180	11	14	18	22	27
	160	7	8	11	14	17
	140	4	5	6	8	11
	120	2	3	4	5	6
40−44	180	9	12	15	19	23
	160	5	7	9	11	14
	140	3	4	5	6	8
	120	1	2	3	4	5

Smoker

Age	SBP	20	25	30	35	40
70−74	180	35	42	50	59	68
	160	26	32	39	47	56
	140	19	24	30	37	44
	120	14	18	22	28	34
65−69	180	30	37	44	53	62
	160	22	27	33	40	48
	140	15	19	24	30	37
	120	11	14	17	22	27
60−64	180	27	34	41	49	58
	160	19	24	29	36	44
	140	13	16	21	26	32
	120	9	11	14	18	22
55−59	180	23	29	35	43	51
	160	15	19	24	30	37
	140	10	13	16	21	26
	120	6	8	11	14	17
50−54	180	19	23	29	36	43
	160	12	15	19	24	30
	140	7	10	12	15	19
	120	5	6	8	10	12
45−49	180	15	19	23	29	36
	160	9	11	15	18	23
	140	5	7	9	11	14
	120	3	4	5	7	9
40−44	180	12	16	20	24	30
	160	7	9	12	15	19
	140	4	5	7	9	11
	120	2	3	4	5	6

Panama

Systolic Blood Pressur (mmHg) by Body Mass Index (kg/m2)

Women

Age	Systolic Blood Pressur (mmHg)	Non−smoker 20	Non−smoker 25	Non−smoker 30	Non−smoker 35	Non−smoker 40	Smoker 20	Smoker 25	Smoker 30	Smoker 35	Smoker 40
70−74	180	16	20	25	31	38	25	31	38	45	54
	160	11	14	18	23	28	18	23	28	35	42
	140	8	10	13	17	21	13	17	21	26	32
	120	6	7	9	12	15	9	12	15	19	24
65−69	180	12	16	20	25	30	20	25	30	37	45
	160	8	11	14	17	22	14	17	22	27	33
	140	6	7	9	12	15	9	12	15	19	24
	120	4	5	6	8	11	6	8	11	13	17
60−64	180	10	13	17	21	26	17	21	26	33	40
	160	7	9	11	14	18	11	14	18	23	28
	140	4	6	7	10	12	7	10	12	15	19
	120	3	4	5	6	8	5	6	8	10	13
55−59	180	8	10	13	17	21	13	17	21	26	32
	160	5	6	8	11	14	8	11	14	17	22
	140	3	4	5	7	9	5	7	9	11	14
	120	2	2	3	4	5	3	4	5	7	9
50−54	180	7	9	12	15	19	12	15	19	24	29
	160	4	6	7	9	12	7	9	12	15	19
	140	2	3	4	6	7	4	6	7	9	12
	120	1	2	2	3	4	2	3	4	6	7
45−49	180	7	9	11	14	18	11	14	18	22	27
	160	4	5	6	8	11	6	8	11	14	17
	140	2	3	4	5	6	4	5	6	8	10
	120	1	1	2	3	4	2	3	4	5	6
40−44	180	5	6	8	10	13	8	10	13	17	21
	160	3	3	4	6	8	4	6	8	10	12
	140	1	2	2	3	4	2	3	4	5	7
	120	1	1	1	2	2	1	2	2	3	4

Men

Age	Systolic Blood Pressur (mmHg)	Non−smoker 20	Non−smoker 25	Non−smoker 30	Non−smoker 35	Non−smoker 40	Smoker 20	Smoker 25	Smoker 30	Smoker 35	Smoker 40
70−74	180	21	26	33	40	48	28	34	42	50	59
	160	16	20	24	30	37	21	26	32	39	47
	140	11	14	18	23	28	15	19	24	30	36
	120	8	10	13	17	21	11	14	18	22	27
65−69	180	19	24	30	37	44	26	32	38	46	55
	160	14	17	22	27	33	18	23	28	35	42
	140	10	12	15	19	24	13	16	20	26	32
	120	7	8	11	14	17	9	11	15	18	23
60−64	180	18	23	28	35	42	24	30	36	44	52
	160	12	16	20	25	30	17	21	26	32	39
	140	8	11	13	17	21	11	14	18	22	28
	120	5	7	9	12	15	7	10	12	15	20
55−59	180	17	22	27	33	41	23	29	35	43	51
	160	11	14	18	23	28	15	19	24	30	37
	140	7	9	12	15	19	10	13	16	20	25
	120	5	6	8	10	13	6	8	11	13	17
50−54	180	15	18	23	29	35	19	24	30	37	45
	160	9	12	15	19	23	12	16	20	25	31
	140	6	7	9	12	15	8	10	13	16	20
	120	3	4	6	7	10	5	6	8	10	13
45−49	180	11	14	18	22	28	15	19	24	29	36
	160	7	9	11	14	18	9	12	15	19	23
	140	4	5	7	9	11	5	7	9	12	15
	120	2	3	4	5	7	3	4	5	7	9
40−44	180	7	9	12	15	19	10	13	16	20	26
	160	4	5	7	9	12	6	8	10	12	16
	140	2	3	4	5	7	3	4	6	7	9
	120	1	2	2	3	4	2	2	3	4	5

Body Mass Index (kg/m2)

Paraguay

Systolic Blood Pressur (mmHg) by Body Mass Index (kg/m2)

Women

Age	Systolic Blood Pressur (mmHg)	Non−smoker 20	Non−smoker 25	Non−smoker 30	Non−smoker 35	Non−smoker 40	Smoker 20	Smoker 25	Smoker 30	Smoker 35	Smoker 40
70−74	180	26	32	40	48	56	39	47	56	65	74
	160	19	24	30	37	44	30	37	44	53	62
	140	14	18	22	28	34	22	27	34	41	49
	120	10	13	16	20	25	16	20	25	31	38
65−69	180	19	24	30	37	44	30	37	44	53	62
	160	13	17	21	27	33	21	27	33	40	48
	140	9	12	15	19	24	15	19	24	29	36
	120	6	8	10	13	17	10	13	17	21	26
60−64	180	18	22	28	34	41	27	34	41	49	58
	160	12	15	19	24	29	19	24	29	36	44
	140	8	10	13	16	20	13	16	20	25	31
	120	5	7	8	11	14	8	11	14	17	22
55−59	180	17	21	26	32	40	26	32	39	47	56
	160	11	14	17	22	27	17	22	27	33	41
	140	7	9	11	14	18	11	14	18	23	28
	120	4	6	7	9	12	7	9	12	15	19
50−54	180	16	20	24	30	37	24	30	37	45	53
	160	10	12	16	20	25	16	20	24	30	37
	140	6	8	10	12	16	10	12	16	20	25
	120	3	5	6	8	10	6	8	10	12	16
45−49	180	14	17	22	27	33	22	27	33	41	49
	160	8	11	13	17	21	13	17	21	26	33
	140	5	6	8	10	13	8	10	13	16	21
	120	3	4	5	6	8	5	6	8	10	13
40−44	180	11	14	18	22	28	18	22	28	34	41
	160	6	8	10	13	17	10	13	17	21	26
	140	3	5	6	8	10	6	8	10	12	16
	120	2	2	3	4	6	3	4	6	7	9

Men

Age	Systolic Blood Pressur (mmHg)	Non−smoker 20	Non−smoker 25	Non−smoker 30	Non−smoker 35	Non−smoker 40	Smoker 20	Smoker 25	Smoker 30	Smoker 35	Smoker 40
70−74	180	25	31	38	46	54	33	40	48	57	66
	160	19	23	29	35	43	25	30	37	45	54
	140	14	17	22	27	33	18	23	28	35	42
	120	10	13	16	20	25	13	17	21	26	32
65−69	180	21	26	32	40	48	28	34	41	50	59
	160	15	19	24	29	36	20	25	31	38	45
	140	10	13	17	21	26	14	18	22	28	34
	120	7	9	12	15	19	10	13	16	20	25
60−64	180	20	25	31	37	45	26	32	39	47	56
	160	13	17	21	27	33	18	23	28	35	42
	140	9	12	15	19	23	12	16	20	24	30
	120	6	8	10	13	16	8	11	13	17	21
55−59	180	18	23	28	34	42	24	30	36	44	52
	160	12	15	19	24	29	16	20	25	31	38
	140	8	10	13	16	20	10	13	17	21	26
	120	5	6	8	10	13	7	9	11	14	18
50−54	180	16	20	25	31	38	21	26	33	40	48
	160	10	13	16	20	26	14	17	22	27	33
	140	6	8	10	13	17	9	11	14	18	22
	120	4	5	6	8	11	5	7	9	11	14
45−49	180	15	19	23	29	36	20	25	30	37	45
	160	9	11	15	18	23	12	15	19	24	30
	140	5	7	9	11	14	7	9	12	15	19
	120	3	4	5	7	9	4	6	7	9	12
40−44	180	12	15	19	24	29	16	20	25	31	38
	160	7	9	11	14	18	9	12	15	19	24
	140	4	5	7	8	11	5	7	9	11	15
	120	2	3	4	5	6	3	4	5	7	9

Body Mass Index (kg/m2)

Peru

Systolic Blood Pressur (mmHg) by Body Mass Index (kg/m2)

Age 70−74

Systolic Blood Pressur (mmHg)	Women Non−smoker 20	25	30	35	40	Women Smoker 20	25	30	35	40	Men Non−smoker 20	25	30	35	40	Men Smoker 20	25	30	35	40
180	27	34	41	49	58	41	49	58	67	76	25	31	38	46	55	33	40	48	57	66
160	20	25	31	38	46	31	38	46	54	63	19	23	29	36	43	25	31	37	45	54
140	15	18	23	29	35	23	28	35	42	51	14	17	22	27	33	18	23	28	35	42
120	10	13	17	21	26	17	21	26	32	39	10	13	16	20	25	13	17	21	26	33

Age 65−69

Systolic Blood Pressur (mmHg)	Women Non−smoker 20	25	30	35	40	Women Smoker 20	25	30	35	40	Men Non−smoker 20	25	30	35	40	Men Smoker 20	25	30	35	40
180	20	25	30	37	45	30	37	45	53	62	21	26	33	40	48	28	34	42	50	59
160	14	17	22	27	33	22	27	33	41	49	15	19	24	29	36	20	25	31	38	46
140	9	12	15	19	24	15	19	24	30	37	11	13	17	21	27	14	18	22	28	34
120	6	8	11	13	17	11	13	17	21	27	7	9	12	15	19	10	13	16	20	25

Age 60−64

Systolic Blood Pressur (mmHg)	Women Non−smoker 20	25	30	35	40	Women Smoker 20	25	30	35	40	Men Non−smoker 20	25	30	35	40	Men Smoker 20	25	30	35	40
180	16	20	25	31	38	25	31	38	45	54	20	24	30	37	45	26	32	39	47	55
160	11	13	17	21	27	17	21	27	33	40	13	17	21	26	33	18	22	28	34	42
140	7	9	11	14	18	11	14	18	23	28	9	11	15	18	23	12	15	19	24	30
120	4	6	7	10	12	7	10	12	16	20	6	8	10	12	16	8	10	13	17	21

Age 55−59

Systolic Blood Pressur (mmHg)	Women Non−smoker 20	25	30	35	40	Women Smoker 20	25	30	35	40	Men Non−smoker 20	25	30	35	40	Men Smoker 20	25	30	35	40
180	14	18	22	28	34	22	28	34	41	49	18	23	28	34	42	24	29	36	44	52
160	9	11	15	18	23	15	18	23	29	35	12	15	19	24	29	16	20	25	31	38
140	6	7	9	12	15	9	12	15	19	24	8	10	12	16	20	10	13	17	21	26
120	3	5	6	8	10	6	8	10	12	16	5	6	8	10	13	7	9	11	14	18

Age 50−54

Systolic Blood Pressur (mmHg)	Women Non−smoker 20	25	30	35	40	Women Smoker 20	25	30	35	40	Men Non−smoker 20	25	30	35	40	Men Smoker 20	25	30	35	40
180	13	16	20	25	31	20	25	31	38	46	16	20	25	31	38	22	27	33	40	48
160	8	10	13	16	20	13	16	20	25	31	10	13	17	21	26	14	18	22	27	34
140	5	6	8	10	13	8	10	13	16	21	6	8	10	13	17	9	11	14	18	22
120	3	4	5	6	8	5	6	8	10	13	4	5	6	8	11	5	7	9	11	14

Age 45−49

Systolic Blood Pressur (mmHg)	Women Non−smoker 20	25	30	35	40	Women Smoker 20	25	30	35	40	Men Non−smoker 20	25	30	35	40	Men Smoker 20	25	30	35	40
180	12	15	18	23	29	18	23	29	35	43	15	18	23	29	35	19	24	30	37	45
160	7	9	11	14	18	11	14	18	22	28	9	11	14	18	23	12	15	19	24	30
140	4	5	7	9	11	7	9	11	14	17	5	7	9	11	14	7	9	12	15	19
120	2	3	4	5	6	4	5	6	8	11	3	4	5	7	9	4	6	7	9	12

Age 40−44

Systolic Blood Pressur (mmHg)	Women Non−smoker 20	25	30	35	40	Women Smoker 20	25	30	35	40	Men Non−smoker 20	25	30	35	40	Men Smoker 20	25	30	35	40
180	9	12	15	19	23	15	19	23	29	36	12	15	19	24	30	16	20	25	31	38
160	5	7	9	11	14	9	11	14	18	22	7	9	11	15	18	10	12	15	19	24
140	3	4	5	6	8	5	6	8	10	13	4	5	7	9	11	5	7	9	12	15
120	1	2	3	3	5	3	3	5	6	8	2	3	4	5	6	3	4	5	7	9

Body Mass Index (kg/m2)

Saint Lucia

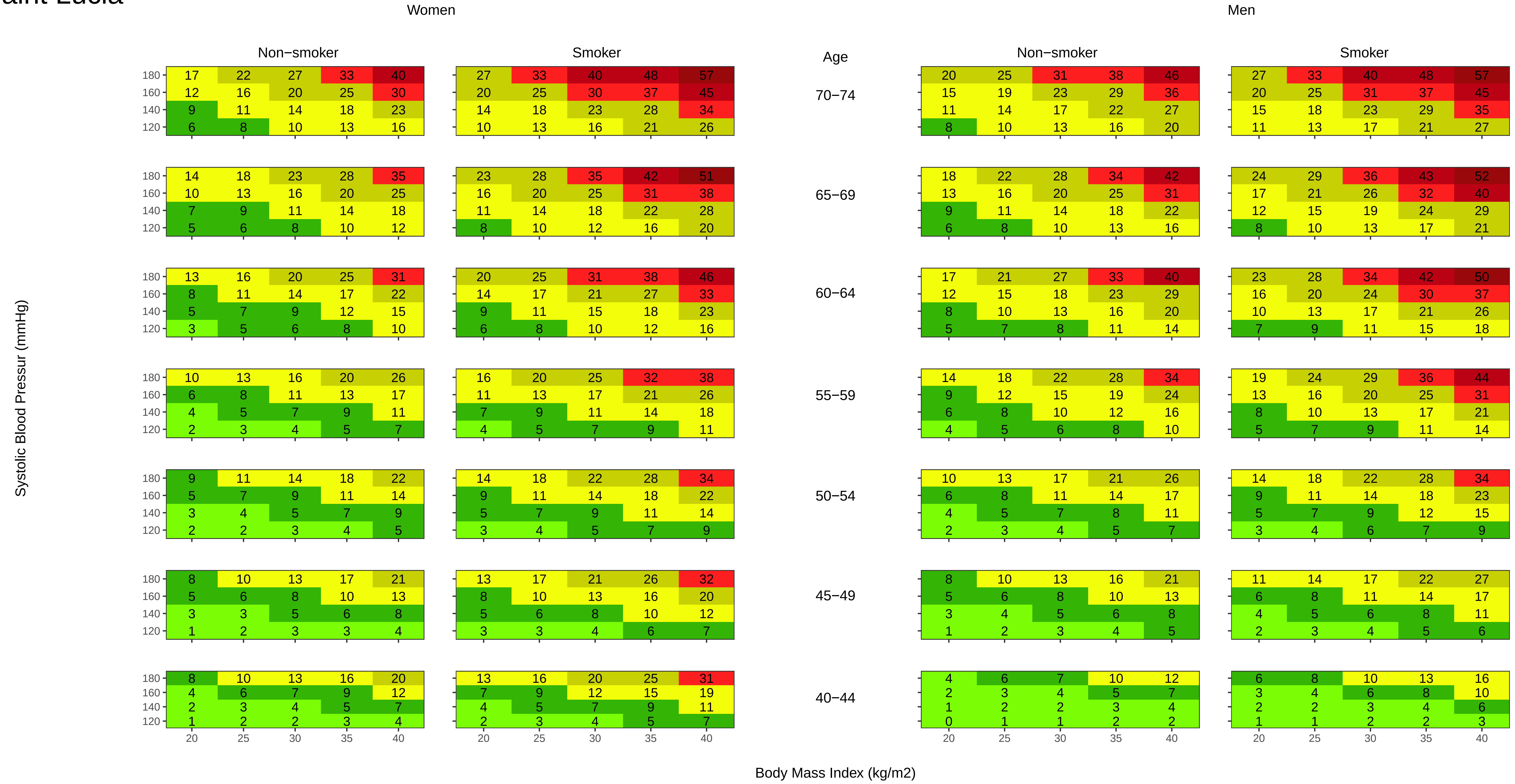

Women
Men
Non−smoker
Smoker
Age
Systolic Blood Pressur (mmHg)
Body Mass Index (kg/m2)
20 25 30 35 40
70−74
Women Non−smoker: 180: 17 22 27 33 40; 160: 12 16 20 25 30; 140: 9 11 14 18 23; 120: 6 8 10 13 16
Women Smoker: 180: 27 33 40 48 57; 160: 20 25 30 37 45; 140: 14 18 23 28 34; 120: 10 13 16 21 26
Men Non−smoker: 180: 20 25 31 38 46; 160: 15 19 23 29 36; 140: 11 14 17 22 27; 120: 8 10 13 16 20
Men Smoker: 180: 27 33 40 48 57; 160: 20 25 31 37 45; 140: 15 18 23 29 35; 120: 11 13 17 21 27
65−69
Women Non−smoker: 180: 14 18 23 28 35; 160: 10 13 16 20 25; 140: 7 9 11 14 18; 120: 5 6 8 10 12
Women Smoker: 180: 23 28 35 42 51; 160: 16 20 25 31 38; 140: 11 14 18 22 28; 120: 8 10 12 16 20
Men Non−smoker: 180: 18 22 28 34 42; 160: 13 16 20 25 31; 140: 9 11 14 18 22; 120: 6 8 10 13 16
Men Smoker: 180: 24 29 36 43 52; 160: 17 21 26 32 40; 140: 12 15 19 24 29; 120: 8 10 13 17 21
60−64
Women Non−smoker: 180: 13 16 20 25 31; 160: 8 11 14 17 22; 140: 5 7 9 12 15; 120: 3 5 6 8 10
Women Smoker: 180: 20 25 31 38 46; 160: 14 17 21 27 33; 140: 9 11 15 18 23; 120: 6 8 10 12 16
Men Non−smoker: 180: 17 21 27 33 40; 160: 12 15 18 23 29; 140: 8 10 13 16 20; 120: 5 7 8 11 14
Men Smoker: 180: 23 28 34 42 50; 160: 16 20 24 30 37; 140: 10 13 17 21 26; 120: 7 9 11 15 18
55−59
Women Non−smoker: 180: 10 13 16 20 26; 160: 6 8 11 13 17; 140: 4 5 7 9 11; 120: 2 3 4 5 7
Women Smoker: 180: 16 20 25 32 38; 160: 11 13 17 21 26; 140: 7 9 11 14 18; 120: 4 5 7 9 11
Men Non−smoker: 180: 14 18 22 28 34; 160: 9 12 15 19 24; 140: 6 8 10 12 16; 120: 4 5 6 8 10
Men Smoker: 180: 19 24 29 36 44; 160: 13 16 20 25 31; 140: 8 10 13 17 21; 120: 5 7 9 11 14
50−54
Women Non−smoker: 180: 9 11 14 18 22; 160: 5 7 9 11 14; 140: 3 4 5 7 9; 120: 2 2 3 4 5
Women Smoker: 180: 14 18 22 28 34; 160: 9 11 14 18 22; 140: 5 7 9 11 14; 120: 3 4 5 7 9
Men Non−smoker: 180: 10 13 17 21 26; 160: 6 8 11 14 17; 140: 4 5 7 8 11; 120: 2 3 4 5 7
Men Smoker: 180: 14 18 22 28 34; 160: 9 11 14 18 23; 140: 5 7 9 12 15; 120: 3 4 6 7 9
45−49
Women Non−smoker: 180: 8 10 13 17 21; 160: 5 6 8 10 13; 140: 3 3 5 6 8; 120: 1 2 3 3 4
Women Smoker: 180: 13 17 21 26 32; 160: 8 10 13 16 20; 140: 5 6 8 10 12; 120: 3 3 4 6 7
Men Non−smoker: 180: 8 10 13 16 21; 160: 5 6 8 10 13; 140: 3 4 5 6 8; 120: 1 2 3 4 5
Men Smoker: 180: 11 14 17 22 27; 160: 6 8 11 14 17; 140: 4 5 6 8 11; 120: 2 3 4 5 6
40−44
Women Non−smoker: 180: 8 10 13 16 20; 160: 4 6 7 9 12; 140: 2 3 4 5 7; 120: 1 2 2 3 4
Women Smoker: 180: 13 16 20 25 31; 160: 7 9 12 15 19; 140: 4 5 7 9 11; 120: 2 3 4 5 7
Men Non−smoker: 180: 4 6 7 10 12; 160: 2 3 4 5 7; 140: 1 2 2 3 4; 120: 0 1 1 2 2
Men Smoker: 180: 6 8 10 13 16; 160: 3 4 6 8 10; 140: 2 2 3 4 6; 120: 1 1 2 2 3

Saint Vincent and the Grenadines

Systolic Blood Pressur (mmHg) by Body Mass Index (kg/m2)

Women — Non−smoker

Age	Systolic Blood Pressur (mmHg)	20	25	30	35	40
70−74	180	35	42	51	60	69
	160	26	32	39	47	56
	140	19	24	30	36	44
	120	14	17	22	27	34
65−69	180	28	34	42	50	59
	160	20	25	31	37	45
	140	14	18	22	27	34
	120	10	12	15	19	24
60−64	180	22	27	33	41	49
	160	15	19	23	29	36
	140	10	13	16	20	25
	120	7	8	11	14	17
55−59	180	18	23	29	35	43
	160	12	15	19	24	30
	140	8	10	12	16	20
	120	5	6	8	10	13
50−54	180	15	19	23	29	36
	160	9	12	15	19	23
	140	6	7	9	12	15
	120	3	4	6	7	9
45−49	180	13	16	20	25	31
	160	8	10	12	16	20
	140	4	6	7	9	12
	120	2	3	4	5	7
40−44	180	10	13	16	20	25
	160	6	7	9	12	15
	140	3	4	5	7	9
	120	2	2	3	4	5

Women — Smoker

Age	Systolic Blood Pressur (mmHg)	20	25	30	35	40
70−74	180	51	60	69	77	85
	160	39	47	56	65	74
	140	30	36	44	52	61
	120	22	27	33	41	49
65−69	180	41	50	58	68	76
	160	31	37	45	54	63
	140	22	27	34	41	49
	120	15	19	24	30	37
60−64	180	33	41	49	58	67
	160	23	29	36	43	52
	140	16	20	25	31	38
	120	11	14	17	22	27
55−59	180	29	35	43	51	60
	160	19	24	30	36	44
	140	12	16	20	25	31
	120	8	10	13	16	21
50−54	180	23	29	35	43	51
	160	15	19	23	29	36
	140	9	12	15	19	24
	120	6	7	9	12	15
45−49	180	20	25	31	38	46
	160	12	16	20	25	30
	140	7	9	12	15	19
	120	4	5	7	9	12
40−44	180	16	20	25	31	38
	160	9	12	15	19	24
	140	5	7	9	11	14
	120	3	4	5	6	8

Men — Non−smoker

Age	Systolic Blood Pressur (mmHg)	20	25	30	35	40
70−74	180	34	41	50	59	68
	160	26	32	39	47	55
	140	19	24	30	36	44
	120	14	18	22	27	34
65−69	180	32	40	48	56	65
	160	24	29	36	43	52
	140	17	21	26	33	40
	120	12	15	19	24	29
60−64	180	27	34	41	49	58
	160	19	24	30	36	44
	140	13	17	21	26	32
	120	9	11	14	18	23
55−59	180	21	26	32	39	47
	160	14	18	22	27	34
	140	9	12	15	19	23
	120	6	8	10	12	16
50−54	180	14	17	22	27	33
	160	9	11	14	18	22
	140	5	7	9	11	14
	120	3	4	5	7	9
45−49	180	11	14	18	23	28
	160	7	9	11	14	18
	140	4	5	7	9	11
	120	2	3	4	5	7
40−44	180	10	12	15	19	24
	160	5	7	9	12	15
	140	3	4	5	7	9
	120	2	2	3	4	5

Men — Smoker

Age	Systolic Blood Pressur (mmHg)	20	25	30	35	40
70−74	180	43	52	61	70	78
	160	33	41	49	58	67
	140	25	31	38	46	54
	120	19	23	29	35	43
65−69	180	41	50	58	68	76
	160	31	38	45	54	63
	140	22	28	34	42	50
	120	16	20	25	31	38
60−64	180	35	43	51	60	69
	160	25	31	38	46	54
	140	17	22	27	34	41
	120	12	15	19	24	30
55−59	180	27	34	41	49	58
	160	19	23	29	35	43
	140	12	16	20	25	30
	120	8	10	13	17	21
50−54	180	18	23	28	35	42
	160	12	15	19	23	29
	140	7	9	12	15	19
	120	4	6	7	10	12
45−49	180	15	19	24	30	37
	160	9	12	15	19	24
	140	5	7	9	12	15
	120	3	4	5	7	9
40−44	180	13	16	21	26	32
	160	8	10	12	16	20
	140	4	6	7	9	12
	120	2	3	4	5	7

Body Mass Index (kg/m2)

Suriname

Systolic Blood Pressur (mmHg) by Body Mass Index (kg/m2)

Age 70–74

Systolic Blood Pressur (mmHg)	Women Non−smoker 20	25	30	35	40	Women Smoker 20	25	30	35	40	Men Non−smoker 20	25	30	35	40	Men Smoker 20	25	30	35	40
180	21	26	32	40	48	32	39	47	56	65	28	34	42	50	59	36	44	52	61	70
160	15	19	24	30	37	24	30	36	44	53	21	26	32	39	47	27	33	41	49	58
140	11	14	18	22	27	18	22	27	34	41	15	19	24	30	36	20	25	31	38	46
120	8	10	13	16	20	13	16	20	25	31	11	14	18	22	28	15	19	23	29	36

Age 65–69

Systolic Blood Pressur (mmHg)	Women Non−smoker 20	25	30	35	40	Women Smoker 20	25	30	35	40	Men Non−smoker 20	25	30	35	40	Men Smoker 20	25	30	35	40
180	16	20	25	31	38	25	31	38	46	54	25	31	38	46	55	33	40	48	57	66
160	11	14	18	22	28	18	22	28	34	41	18	23	28	35	42	24	30	36	44	52
140	8	10	12	16	20	12	16	20	25	30	13	16	20	25	31	17	21	27	33	40
120	5	7	8	11	14	8	11	14	17	22	9	11	14	18	23	12	15	19	24	30

Age 60–64

Systolic Blood Pressur (mmHg)	Women Non−smoker 20	25	30	35	40	Women Smoker 20	25	30	35	40	Men Non−smoker 20	25	30	35	40	Men Smoker 20	25	30	35	40
180	12	16	20	25	30	20	25	30	37	45	22	27	34	41	49	29	35	43	51	60
160	8	10	13	17	21	13	17	21	26	32	15	19	24	29	36	20	25	31	38	46
140	5	7	9	11	14	9	11	14	18	23	10	13	16	21	26	14	17	22	27	33
120	3	4	6	7	9	6	7	9	12	15	7	9	11	14	18	9	12	15	19	24

Age 55–59

Systolic Blood Pressur (mmHg)	Women Non−smoker 20	25	30	35	40	Women Smoker 20	25	30	35	40	Men Non−smoker 20	25	30	35	40	Men Smoker 20	25	30	35	40
180	10	13	17	21	26	17	21	26	32	39	20	25	31	38	46	27	33	40	48	57
160	7	8	11	14	17	11	14	17	22	27	13	17	21	27	33	18	23	28	34	42
140	4	5	7	9	11	7	9	11	14	18	9	11	14	18	22	12	15	19	24	29
120	2	3	4	6	7	4	5	7	9	12	6	7	9	12	15	8	10	12	16	20

Age 50–54

Systolic Blood Pressur (mmHg)	Women Non−smoker 20	25	30	35	40	Women Smoker 20	25	30	35	40	Men Non−smoker 20	25	30	35	40	Men Smoker 20	25	30	35	40
180	11	13	17	21	26	17	21	26	33	40	20	25	31	38	46	26	33	40	48	56
160	6	8	11	13	17	11	13	17	21	27	13	16	20	25	31	17	22	27	33	40
140	4	5	6	8	11	6	8	11	13	17	8	10	13	16	21	11	14	17	22	27
120	2	3	4	5	6	4	5	6	8	11	5	6	8	10	13	7	9	11	14	18

Age 45–49

Systolic Blood Pressur (mmHg)	Women Non−smoker 20	25	30	35	40	Women Smoker 20	25	30	35	40	Men Non−smoker 20	25	30	35	40	Men Smoker 20	25	30	35	40
180	10	13	16	20	25	16	20	25	31	38	19	24	30	36	44	25	31	38	46	55
160	6	8	10	12	16	10	12	16	20	25	12	15	19	24	29	16	20	25	31	38
140	3	4	6	7	9	6	7	9	12	15	7	9	12	15	19	10	12	16	20	25
120	2	2	3	4	5	3	4	5	7	9	4	5	7	9	11	6	7	10	12	15

Age 40–44

Systolic Blood Pressur (mmHg)	Women Non−smoker 20	25	30	35	40	Women Smoker 20	25	30	35	40	Men Non−smoker 20	25	30	35	40	Men Smoker 20	25	30	35	40
180	7	9	12	15	19	12	15	19	24	30	13	17	21	26	33	18	22	28	34	42
160	4	5	7	9	11	7	9	11	14	18	8	10	13	16	20	11	14	17	21	27
140	2	3	4	5	6	4	5	6	8	11	4	6	7	10	12	6	8	10	13	16
120	1	1	2	3	4	2	3	4	5	6	2	3	4	5	7	3	4	6	8	10

Body Mass Index (kg/m2)

The Bahamas

Systolic Blood Pressur (mmHg) by Body Mass Index (kg/m2)

Age 70−74

Systolic BP	Women Non−smoker					Women Smoker					Men Non−smoker					Men Smoker				
BMI	20	25	30	35	40	20	25	30	35	40	20	25	30	35	40	20	25	30	35	40
180	13	16	21	26	32	21	26	32	39	47	16	21	26	32	39	22	27	33	40	49
160	9	12	15	19	23	15	19	23	29	36	12	15	19	24	29	16	20	25	31	38
140	6	8	11	14	17	11	13	17	21	27	9	11	14	18	22	12	15	19	23	29
120	4	6	7	10	12	7	10	12	16	20	6	8	10	13	16	8	11	14	17	22

Age 65−69

Systolic BP	Women Non−smoker					Women Smoker					Men Non−smoker					Men Smoker				
BMI	20	25	30	35	40	20	25	30	35	40	20	25	30	35	40	20	25	30	35	40
180	11	13	17	21	27	17	21	26	33	40	13	17	21	27	33	18	23	28	34	42
160	7	9	12	15	19	12	15	19	23	29	9	12	15	19	24	13	16	20	25	31
140	5	6	8	10	13	8	10	13	17	21	6	8	11	13	17	9	11	14	18	22
120	3	4	5	7	9	5	7	9	11	15	4	6	7	9	12	6	8	10	13	16

Age 60−64

Systolic BP	Women Non−smoker					Women Smoker					Men Non−smoker					Men Smoker				
BMI	20	25	30	35	40	20	25	30	35	40	20	25	30	35	40	20	25	30	35	40
180	9	11	14	18	22	14	18	22	27	34	13	17	21	26	33	18	22	28	34	42
160	6	7	9	12	15	9	12	15	19	24	9	11	15	18	23	12	15	19	24	30
140	4	5	6	8	10	6	8	10	13	16	6	8	10	12	16	8	10	13	17	21
120	2	3	4	5	7	4	5	7	8	11	4	5	6	8	11	5	7	9	11	14

Age 55−59

Systolic BP	Women Non−smoker					Women Smoker					Men Non−smoker					Men Smoker				
BMI	20	25	30	35	40	20	25	30	35	40	20	25	30	35	40	20	25	30	35	40
180	8	10	12	16	20	12	16	20	25	30	14	17	22	27	33	18	23	28	35	42
160	5	6	8	10	13	8	10	13	16	20	9	11	14	18	23	12	15	19	24	30
140	3	4	5	6	8	5	6	8	10	13	6	7	9	12	15	8	10	13	16	20
120	2	2	3	4	5	3	4	5	7	8	3	5	6	8	10	5	6	8	10	13

Age 50−54

Systolic BP	Women Non−smoker					Women Smoker					Men Non−smoker					Men Smoker				
BMI	20	25	30	35	40	20	25	30	35	40	20	25	30	35	40	20	25	30	35	40
180	7	9	12	15	19	12	15	19	24	29	13	17	21	26	33	18	22	28	34	42
160	4	6	7	9	12	7	9	12	15	19	8	11	14	17	22	11	14	18	23	28
140	2	3	4	6	7	4	6	7	9	12	5	7	9	11	14	7	9	12	15	19
120	1	2	2	3	4	2	3	4	6	7	3	4	5	7	9	4	6	7	9	12

Age 45−49

Systolic BP	Women Non−smoker					Women Smoker					Men Non−smoker					Men Smoker				
BMI	20	25	30	35	40	20	25	30	35	40	20	25	30	35	40	20	25	30	35	40
180	6	8	10	13	16	10	13	16	20	25	12	16	20	25	31	17	21	26	32	39
160	3	4	6	8	10	6	8	10	12	16	8	10	12	16	20	10	13	17	21	26
140	2	2	3	4	6	3	4	6	7	9	4	6	7	10	12	6	8	10	13	16
120	1	1	2	2	3	2	2	3	4	5	2	3	4	6	7	4	5	6	8	10

Age 40−44

Systolic BP	Women Non−smoker					Women Smoker					Men Non−smoker					Men Smoker				
BMI	20	25	30	35	40	20	25	30	35	40	20	25	30	35	40	20	25	30	35	40
180	4	5	7	9	11	7	9	11	14	18	11	13	17	21	27	14	18	22	28	34
160	2	3	4	5	6	4	5	6	8	11	6	8	10	13	16	8	11	14	17	22
140	1	1	2	3	4	2	3	4	5	6	3	4	6	7	10	5	6	8	10	13
120	0	1	1	1	2	1	1	2	2	3	2	2	3	4	5	3	3	4	6	8

Body Mass Index (kg/m2)

Trinidad and Tobago

Systolic Blood Pressur (mmHg) by Body Mass Index (kg/m2)

		Women										Men									
		Non−smoker					Smoker					Non−smoker					Smoker				
Age	SBP	20	25	30	35	40	20	25	30	35	40	20	25	30	35	40	20	25	30	35	40
70−74	180	23	29	36	43	52	36	43	51	60	69	28	34	41	50	58	36	43	52	61	70
	160	17	21	27	33	40	27	33	40	48	57	21	26	32	39	47	27	33	41	49	57
	140	12	15	20	24	30	19	24	30	37	45	15	19	24	29	36	20	25	31	38	46
	120	9	11	14	18	22	14	18	22	28	34	11	14	18	22	27	15	19	23	29	35
65−69	180	18	23	28	35	42	28	35	42	50	59	24	30	37	44	53	32	39	46	55	64
	160	13	16	20	25	31	20	25	31	38	46	17	22	27	33	40	23	28	35	42	51
	140	9	11	14	18	22	14	18	22	28	34	12	15	19	24	30	16	20	26	32	39
	120	6	8	10	12	16	10	12	16	20	25	8	11	14	17	22	11	14	18	23	28
60−64	180	15	19	24	30	37	24	30	37	44	53	22	28	34	42	50	29	36	44	52	61
	160	10	13	17	21	26	17	21	26	32	39	15	19	24	30	37	21	26	32	39	47
	140	7	9	11	14	18	11	14	18	22	28	10	13	17	21	26	14	18	22	28	34
	120	4	6	7	9	12	7	9	12	15	19	7	9	11	14	18	9	12	15	19	24
55−59	180	13	17	21	26	32	21	26	32	39	47	21	26	32	39	47	27	34	41	49	58
	160	8	11	14	17	22	14	17	22	27	33	14	17	22	27	34	18	23	29	35	43
	140	5	7	9	11	14	9	11	14	18	23	9	11	15	18	23	12	15	19	24	30
	120	3	4	6	7	9	5	7	9	12	15	6	7	9	12	15	8	10	13	16	20
50−54	180	10	13	17	21	26	17	21	26	32	40	18	23	29	35	43	24	30	37	45	53
	160	6	8	11	13	17	11	13	17	21	27	12	15	19	24	29	16	20	25	31	38
	140	4	5	6	8	11	6	8	11	14	17	7	9	12	15	19	10	13	16	20	25
	120	2	3	4	5	7	4	5	6	8	11	4	6	7	10	12	6	8	10	13	16
45−49	180	8	10	13	16	20	13	16	20	25	31	16	20	24	30	37	21	26	32	39	47
	160	4	6	8	10	12	7	10	12	16	20	9	12	15	19	24	13	16	20	25	31
	140	2	3	4	6	7	4	6	7	9	12	6	7	9	12	15	8	10	13	16	20
	120	1	2	2	3	4	2	3	4	5	7	3	4	6	7	9	5	6	8	10	12
40−44	180	5	7	9	11	14	9	11	14	18	22	11	14	18	23	28	15	19	24	30	37
	160	3	4	5	6	8	5	6	8	11	13	7	8	11	14	17	9	12	15	18	23
	140	1	2	3	4	5	3	3	5	6	8	4	5	6	8	10	5	7	9	11	14
	120	1	1	1	2	2	1	2	2	3	4	2	3	4	5	6	3	4	5	6	8

Uruguay

Women

Age	Systolic Blood Pressur (mmHg)	Non−smoker BMI 20	25	30	35	40	Smoker BMI 20	25	30	35	40
70−74	180	15	19	23	29	36	23	29	35	43	51
	160	11	13	17	21	27	17	21	26	33	40
	140	7	10	12	15	19	12	15	19	24	30
	120	5	7	9	11	14	9	11	14	18	22
65−69	180	12	15	19	24	30	19	24	30	36	44
	160	8	11	13	17	21	13	17	21	26	33
	140	6	7	9	12	15	9	12	15	19	23
	120	4	5	6	8	10	6	8	10	13	17
60−64	180	10	13	16	20	25	16	20	25	31	38
	160	7	8	11	14	17	11	14	17	22	27
	140	4	5	7	9	12	7	9	12	15	19
	120	3	3	5	6	8	5	6	8	10	13
55−59	180	8	11	13	17	21	13	17	21	26	33
	160	5	7	9	11	14	9	11	14	18	22
	140	3	4	5	7	9	5	7	9	11	15
	120	2	2	3	4	6	3	4	6	7	9
50−54	180	6	8	11	14	17	11	14	17	22	27
	160	4	5	7	8	11	7	8	11	14	17
	140	2	3	4	5	7	4	5	7	9	11
	120	1	2	2	3	4	2	3	4	5	7
45−49	180	4	6	7	10	12	7	10	12	15	19
	160	2	3	4	6	7	4	6	7	9	12
	140	1	2	2	3	4	2	3	4	5	7
	120	1	1	1	2	2	1	2	2	3	4
40−44	180	3	4	5	7	8	5	6	8	11	14
	160	1	2	3	4	5	3	4	5	6	8
	140	1	1	1	2	3	1	2	3	3	4
	120	0	0	1	1	1	1	1	1	2	2

Men

Age	Systolic Blood Pressur (mmHg)	Non−smoker BMI 20	25	30	35	40	Smoker BMI 20	25	30	35	40
70−74	180	21	26	32	39	47	27	33	41	49	58
	160	15	19	24	29	36	20	25	31	38	46
	140	11	14	18	22	27	15	19	23	29	35
	120	8	10	13	16	20	11	14	17	21	27
65−69	180	18	22	28	34	42	24	29	36	43	52
	160	13	16	20	25	31	17	21	26	32	40
	140	9	11	14	18	22	12	15	19	24	29
	120	6	8	10	13	16	8	10	13	17	21
60−64	180	16	20	25	30	37	21	26	32	39	47
	160	11	13	17	21	26	14	18	22	28	34
	140	7	9	11	15	18	10	12	15	19	24
	120	5	6	8	10	13	6	8	10	13	17
55−59	180	14	18	22	27	34	19	23	29	35	43
	160	9	12	15	18	23	12	15	20	24	30
	140	6	7	10	12	15	8	10	13	16	20
	120	4	5	6	8	10	5	6	8	11	14
50−54	180	12	15	19	24	29	16	20	25	31	38
	160	7	9	12	15	19	10	13	16	20	25
	140	4	6	8	10	12	6	8	10	13	17
	120	3	3	5	6	8	4	5	6	8	10
45−49	180	9	11	15	18	23	12	15	19	24	30
	160	5	7	9	11	14	7	9	12	15	19
	140	3	4	5	7	9	4	6	7	9	12
	120	2	2	3	4	5	2	3	4	5	7
40−44	180	6	7	10	12	15	8	10	13	16	21
	160	3	4	5	7	9	4	6	8	10	12
	140	2	2	3	4	5	2	3	4	6	7
	120	1	1	2	2	3	1	2	2	3	4

Body Mass Index (kg/m2)

Venezuela

Systolic Blood Pressur (mmHg) by Body Mass Index (kg/m2)

Women

Age	SBP	Non−smoker 20	25	30	35	40	Smoker 20	25	30	35	40
70−74	180	27	33	40	48	57	40	48	57	66	75
	160	20	25	30	37	45	30	37	45	53	62
	140	14	18	22	28	34	22	28	34	42	50
	120	10	13	16	21	26	16	21	26	32	39
65−69	180	21	26	32	39	47	32	39	47	56	65
	160	15	19	23	29	35	23	29	35	43	51
	140	10	13	16	21	26	16	21	26	32	39
	120	7	9	11	14	18	11	14	18	23	28
60−64	180	17	21	26	32	39	26	32	39	47	56
	160	11	14	18	22	28	18	22	28	34	41
	140	7	9	12	15	19	12	15	19	24	30
	120	5	6	8	10	13	8	10	13	16	20
55−59	180	15	19	24	30	36	24	30	36	44	53
	160	10	13	16	20	25	16	20	25	31	38
	140	6	8	10	13	16	10	13	16	21	26
	120	4	5	6	8	11	6	8	11	13	17
50−54	180	14	18	22	28	34	22	28	34	42	50
	160	9	11	14	18	23	14	18	22	28	34
	140	5	7	9	11	14	9	11	14	18	23
	120	3	4	5	7	9	5	7	9	11	14
45−49	180	12	15	19	23	29	18	23	29	35	43
	160	7	9	11	14	18	11	14	18	23	28
	140	4	5	7	9	11	7	9	11	14	18
	120	2	3	4	5	6	4	5	6	8	11
40−44	180	8	10	13	17	21	13	17	21	26	32
	160	5	6	8	10	12	8	10	12	16	20
	140	2	3	4	6	7	4	6	7	9	12
	120	1	2	2	3	4	2	3	4	5	7

Men

Age	SBP	Non−smoker 20	25	30	35	40	Smoker 20	25	30	35	40
70−74	180	33	40	48	57	66	42	50	59	68	77
	160	25	31	38	45	54	32	39	47	56	65
	140	18	23	29	35	43	24	30	37	45	53
	120	13	17	21	27	33	18	23	28	34	42
65−69	180	26	32	39	47	55	33	41	49	58	67
	160	18	23	29	35	43	24	30	37	45	53
	140	13	17	21	26	32	17	22	27	34	41
	120	9	12	15	19	23	12	16	20	25	30
60−64	180	22	27	34	41	49	29	35	43	51	60
	160	15	19	24	30	36	20	25	31	38	46
	140	10	13	16	21	26	14	17	22	27	34
	120	7	9	11	14	18	9	12	15	19	24
55−59	180	22	28	34	41	49	29	36	43	52	61
	160	15	19	23	29	35	20	25	30	37	45
	140	10	12	16	20	24	13	16	21	26	32
	120	6	8	10	13	16	8	11	14	17	22
50−54	180	20	26	32	39	46	27	33	40	49	57
	160	13	17	21	26	32	18	22	27	34	41
	140	8	11	13	17	21	11	14	18	22	28
	120	5	7	8	11	14	7	9	11	14	18
45−49	180	16	21	26	32	39	22	27	33	41	49
	160	10	13	16	20	25	14	17	21	27	33
	140	6	8	10	13	16	8	11	13	17	21
	120	3	5	6	8	10	5	6	8	10	13
40−44	180	11	14	17	21	27	14	18	23	28	35
	160	6	8	10	13	16	8	11	14	17	22
	140	3	4	6	8	10	5	6	8	10	13
	120	2	2	3	4	6	3	3	5	6	8

Body Mass Index (kg/m2)
